# ALKBH3 m1A Demethylase Deficiency Reduces Alzheimer's Amyloid‐β Pathology

**DOI:** 10.1002/advs.202522572

**Published:** 2026-03-12

**Authors:** Yueyang Li, Sifei Yu, Kaidong Lu, Yujie Zhang, Mingjie Dong, Yan Peng, Liang Xue, Waleed Alam, Yuxuan Shui, Yi Zhou, Wuyunhan Ma, Meng Bao, Peiming Li, Peiyi Luo, Tiezhan Lu, Jiajia Li, Kang Zhang, Yuying Wang, Shuchen Yang, Nuoya Yin, Francesco Faiola, Zilong Gao, Jingfeng Zhou, Fei Zhao, Yali He, Magdalena J. Koziol

**Affiliations:** ^1^ Peking University Beijing China; ^2^ Beijing Institute for Brain Research Chinese Academy of Medical Sciences & Peking Union Medical College Beijing China; ^3^ Chinese Institute for Brain Research, Beijing Beijing China; ^4^ School of Basic Medical Sciences Capital Medical University Beijing China; ^5^ College of Biological Sciences China Agricultural University Beijing China; ^6^ Research Unit of Medical Neurobiology Chinese Academy of Medical Sciences Beijing China; ^7^ Department of Neurology Beijing Tiantan Hospital Capital Medical University Beijing China; ^8^ Department of Bioengineering University of Pennsylvania Philadelphia Pennsylvania USA; ^9^ State Key Laboratory of Environmental Chemistry and Ecotoxicology Research Center for Eco‐Environmental Sciences Chinese Academy of Sciences Beijing China; ^10^ College of Resources and Environment University of Chinese Academy of Sciences Beijing China; ^11^ State Key Laboratory of Cognitive Neuroscience and Learning Beijing Normal University Beijing China

**Keywords:** ALKBH3, Alzheimer's disease (AD), Amyloid‐β, m1A, mitochondrial, mitophagy

## Abstract

Amyloid‐beta (Aβ) aggregation, mitochondrial dysfunction, and cognitive decline are hallmarks of Alzheimer's disease (AD), but its initiating molecular events remain unknown. Given that RNA modifications regulate neurodevelopment and neurodegeneration, we explore their functional role in 5xFAD mice, an Aβ AD model. We discover that N1‐methyladenosine (m1A) is the most altered RNA modification, and that its regulator demethylase, ALKBH3 is upregulated. Strikingly, *Alkbh3* reduction decreases Aβ plaques and restores cognition. Conversely, elevated ALKBH3 levels, observed in AD patients, compromise neuronal morphology and mitochondrial function by impairing mitophagy (degradation of dysfunctional mitochondria), a known driver of neuronal dysfunction. Mechanistically, we reveal that ALKBH3 removes m1A from PINK1 mRNA, the mitophagy master regulator. Given that ALKBH3 is elevated in human AD, causally linked to mitophagy impairment, and confers neuroprotection when depleted, we present ALKBH3 as a mechanistically validated therapeutic target in AD.

## Graphical abstract

This study identifies that ALKBH3‐driven m1A demethylation orchestrates Alzheimer's disease progression by disrupting mitochondrial and synaptic homeostasis. This epitranscriptomic mechanism suppresses PINK1‐mediated mitophagy via m1A erasure, leading to mitochondrial dysfunction, oxidative stress, elevated Aβ production, and impaired microglial phagocytic clearance—culminating in pathogenic Aβ accumulation.

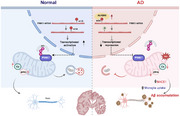

## 1 Introduction

AD remains a defining and unsolved challenge in neurobiology, characterized by the progressive accumulation of Aβ plaques and profound mitochondrial dysfunction [1, 2, 3]. The recent approval of anti‐amyloid therapies, while a landmark, provides only modest symptomatic benefit [4, 5], underscoring a critical gap in our understanding: the molecular triggers that initiate and connect these hallmark pathologies remain elusive. Identifying these upstream drivers is essential for developing transformative, next‐generation therapeutics that can halt or prevent disease progression. In recent years, therapeutics targeting the epitranscriptome have provided clinically meaningful disease‐modifying outcomes in systemic disorders [6, 7].

The epitranscriptome—a dynamic layer of post‐transcriptional RNA modifications—has emerged as a fundamental regulator of gene expression, with demonstrated roles in neurodevelopment and cellular stress responses [8, 9, 10, 11]. Modifications such as N6‐methyladenosine (m6A) fine‐tune neuronal transcriptomes, influencing RNA stability, localization, and translation [12, 13]. However, the functional significance of the vast landscape of non‐m6A RNA modifications in chronic neurodegeneration, particularly in AD, is virtually uncharted [14, 15, 16]. Among these, N1‐methyladenosine (m1A) is a reversible mRNA modification that potently influences translational efficiency [17, 18, 19], yet its role in the brain and its potential dysregulation in disease are unknown. This gap represents a missed opportunity to understand a potentially pivotal layer of gene regulation underlying AD pathogenesis.

Here, we systematically profiled the RNA modification landscape in the AD brain and identified m1A as the most significantly altered modification. We pinpoint the dysregulation of the m1A demethylase ALKBH3 as the causal driver of this epitranscriptomic deficit. We demonstrate that ALKBH3 ablation in a 5xFAD mouse model not only rescues Aβ pathology and cognitive function but also restores mitochondrial integrity by rescuing mitophagy. Mechanistically, we establish that ALKBH3 directly targets PINK1 mRNA, erasing its m1A marks and thereby destabilizing a master regulator of mitochondrial quality control [20, 21]. Our work defines a previously unrecognized ALKBH3‐m1A‐PINK1 axis that directly links epitranscriptomic dysregulation to the core proteotoxic and metabolic failures in AD, positioning ALKBH3 as a mechanistically validated and therapeutically tractable target.

## 2 Results

### 2.1 ALKBH3 Upregulation and m1A Loss in the AD Hippocampus

**FIGURE 1:**
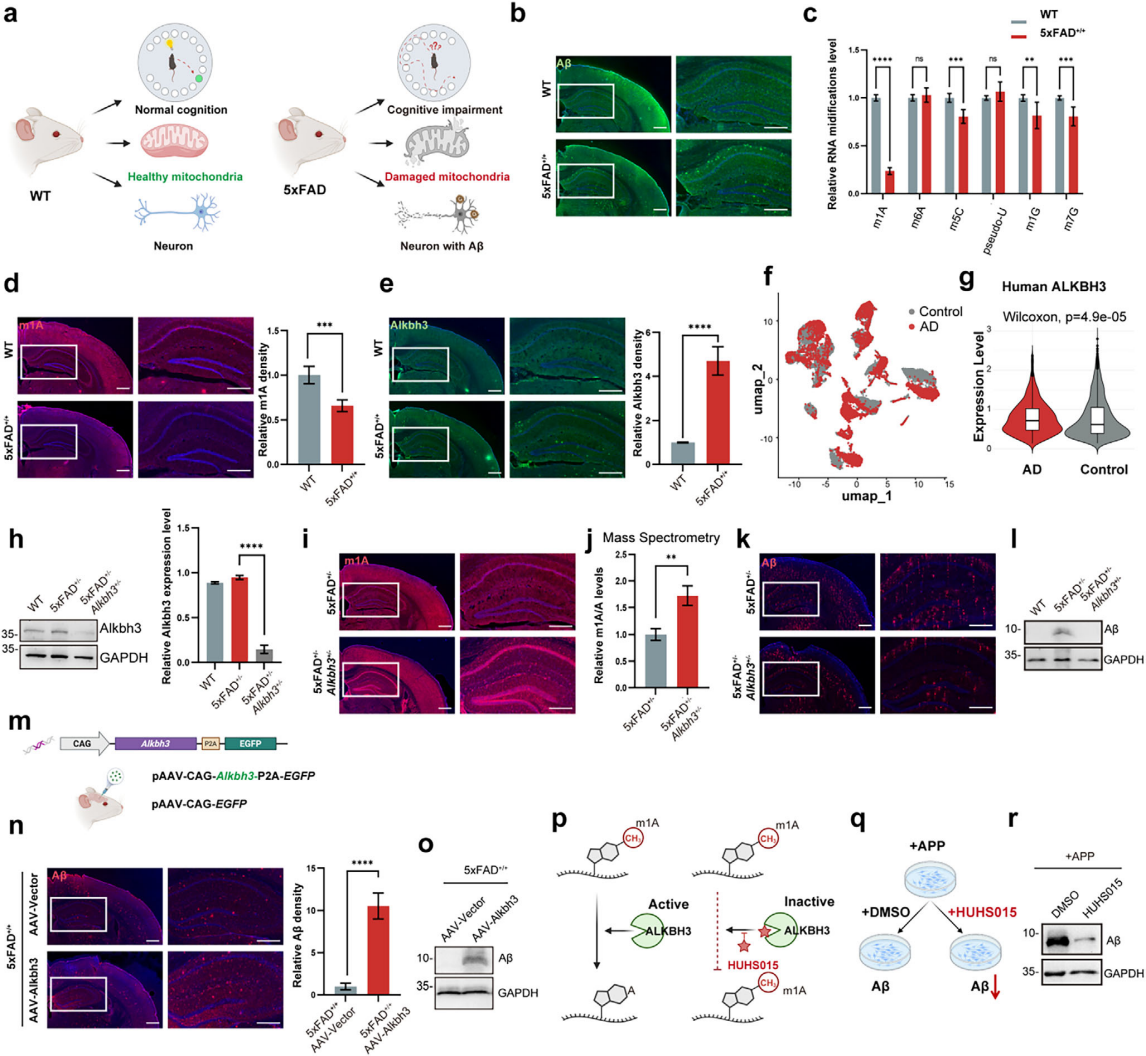
Alkbh3 modulates m1A and Aβ in 5xFAD mice hippocampi. (a) Key neuropathological and behavioral features of Alzheimer's disease modeled in 5xFAD mice; (b) immunofluorescence (IF) images of Aβ deposition (green) in 6‐month‐old WT vs. 5xFAD^+/+^;(c) m1A reduction in 5xFAD^+/+^ and WT hippocampi vs. other RNA modifications (LC‐MS/MS; n = 6/group); (d) m1A levels (red, IF) in 6‐month‐old WT versus 5xFAD^+/+^ hippocampi; (e) Alkbh3 levels (green, IF) in 6‐month‐old WT vs. 5xFAD^+/+^ hippocampi; (f) UMAP visualizations of single‐cell RNA‐seq data from human AD (red) and control (grey) hippocampal cortical samples; (g) Violin plots showing ALKBH3 expression across all cell types in AD versus Control samples (Wilcoxon rank‐sum test, p = 4.9 × 10^−5^). (h) Alkbh3 protein levels by Western Blot (WB), with GAPDH as loading control; (i) m1A levels in 8‐month‐old 5xFAD^+/−^ *Alkbh3^+/−^* versus 5xFAD^+/−^ hippocampi (red, IF); (j) m1A levels (LC‐MS/MS); (k) Aβ reduction in 8‐month‐old 5xFAD^+/−^ *Alkbh3^+/−^* versus 5xFAD^+/−^ (red, IF); (l) Aβ reduction (WB); (m) AAV‐mediated gene overexpression in mouse hippocampi. Schematic of AAV plasmid design (top) and hippocampal injection strategy (bottom). (n) AAV‐Alkbh3 exacerbates Aβ in 8‐month‐old 5xFAD^+/+^ hippocampi versus AAV‐Vector (red, IF); IF and corresponding quantification is shown; (o) Aβ protein levels in AAV‐mediated ALKBH3 overexpression in mouse hippocampi by WB, with GAPDH as loading control; (p) Model of HUHS015‐mediated ALKBH3 inhibition. Diagram depicts HUHS015 (red star) binding to ALKBH3 (green), blocking its catalytic m1A demethylase activity; (q) Diagram depicting SH‐SY5Y cells transfected with APP to evaluate Aβ deposition without (left) or with HUHS015 (right); (r) Aβ modulation by HUHS015 in SH‐SY5Y cells (WB, with GAPDH as loading control); representative images are shown for IF (blue for DAPI), and WB; two‐tailed unpaired *t*‐test; data: mean ± SEM; *n* = 3 biological replicates unless noted; scale bar: 0.5 mm; *p < 0.05, **p < 0.01, ***p < 0.001, ****p < 0.0001.

We first quantified the RNA modification landscape in the hippocampus, a region central to cognitive decline [3, 22], from homozygous 6‐month‐old 5xFAD mice (5xFAD^+/+^). These mice carry familial AD mutations in human *APP* and *PSEN1*, leading to early and aggressive amyloid pathology (Figure 1a,b) [23]. Through Liquid Chromatography coupled with Tandem Mass Spectrometry (LC‐MS/MS), we revealed a selective and striking >3‐fold reduction in m1A in 5xFAD^+/+^ hippocampi, while there were no significant changes or substantially smaller alterations in other modifications (Figure 1c). This pronounced m1A decrease, confirmed by immunofluorescence (Figure 1d), pointed to a specific role for m1A dysregulation in AD pathogenesis.

Since RNA modifications are enzymatically controlled by methyltransferases (writers) and demethylases (erasers) enzymes [9, 24, 25, 26, 27, 28], we hypothesized that the observed m1A loss stemmed from an imbalance in their expression. Upon systematic profiling of hippocampal mRNA from 5xFAD^+/+^ mice, we identified a >2‐fold upregulation of the m1A demethylase *Alkbh3*, while other known writers and erasers were unaltered (Figure S1). This increase was confirmed at the protein level by immunofluorescence (IF) (Figure 1e). As ALKBH3 is a known eraser of m1A modifications on mRNA targets [27, 29], its upregulation provides a direct mechanistic explanation for the m1A reduction observed in 5xFAD^+/+^ mice.

To evaluate the translational relevance of these findings, we analyzed scRNA‐seq data from human AD and control brain samples containing cells of the hippocampus and cortex [30]. Uniform Manifold Approximation and Projection (UMAP) visualization revealed distinct cell populations and elevated ALKBH3 expression in AD samples compared to controls (Figure 1f, S2a). Quantitative analysis confirmed a significant upregulation of ALKBH3 expression in AD samples overall (Wilcoxon rank‐sum test, p = 4.9 × 10^−5^; Figure 1g), and across multiple cell types (Figure S2b). The conservation of ALKBH3 dysregulation between 5xFAD mice and human AD patients underscores the clinical relevance of our findings and highlights the utility of the 5xFAD hippocampus for investigating this epitranscriptomic pathway.

Given the medical relevance of our findings and the established role of Aβ deposition in AD pathogenesis, we next sought to determine whether ALKBH3 changes directly contribute to AD phenotypes in 5xFAD mice.

### 2.2 Increase in Alkbh3 Drives Aβ Deposition

To investigate Alkbh3's role in the hippocampus, we generated *Alkbh3* knockout mice (*Alkbh3^−^* ^/−^) and crossed them with 5xFAD mice (Western blotting: WB, Figure 1h). All following experiments used heterozygotes for 5xFAD and for *Alkbh3* knockout cohorts (hereafter 5xFAD^+/−^ *Alkbh3* ^+/−^) and their heterozygous 5xFAD littermate controls (hereafter 5xFAD^+/−^) (Figure S3b–f). To determine whether *Alkbh3* deletion affects m1A levels in 5xFAD mice, we assessed m1A levels using both immunofluorescence and LC‐MS/MS. Both methods consistently showed increased m1A levels in 5xFAD^+/−^ *Alkbh3* ^±^ mice compared to 5xFAD^+/−^ controls: immunofluorescence confirmed elevated m1A signal in hippocampal tissue (Figure 1i), and quantitative LC‐MS/MS analysis confirmed a significant increase in the global m1A/A ratio (Figure 1j). This indicates that ALKBH3 depletion can partially reverse the m1A reduction characteristic of 5xFAD mice.

Given Aβ’s established role in AD pathogenesis [5, 23], we asked whether *Alkbh3* depletion affects Aβ deposition. In 8‐month‐old 5xFAD^+/−^ *Alkbh3* ^±^ mice, we detected a pronounced reduction in Aβ plaque burden compared to age‐matched 5xFAD^+/−^ controls, as assessed by IF and WB (Figure 1k,l, Figure S4a). To determine whether Alkbh3 elevation drives Aβ deposition, we also developed an AAV‐mediated overexpression system. We constructed AAV vectors expressing *Alkbh3* (pAAV‐CAG‐*Alkbh3*‐P2A‐*EGFP;* hereafter AAV‐Alkbh3) and a control (pAAV‐CAG‐P2A‐*EGFP*; AAV‐Vector). These vectors were injected into the hippocampus of 5xFAD^+/+^ littermates, with Alkbh3 overexpression effects evaluated two weeks post‐injection (Figure 1m). Mirroring our genetic depletion results, AAV‐mediated Alkbh3 overexpression significantly reduced m1A levels in 5xFAD hippocampi (Figure S4b,c). Notably, just 2 weeks of Alkbh3 overexpression was sufficient to substantially increase Aβ deposition compared to control‐injected animals (Figure 1n,o, Figure S4d).

Given the potent role of ALKBH3 in driving Aβ deposition, we investigated whether its pharmacological inhibition could mitigate Aβ accumulation. Using the ALKBH3 inhibitor HUHS015 (Figure 1p, Figure S4e) [31, 32], we validated its target engagement through reduced ALKBH3 protein (Figure S4f). Crucially, we established its functional efficacy by demonstrating a >4‐fold increase in m1A methylation (Figure S4g). To assess therapeutic potential in a disease‐relevant context, we overexpressed APP in HEK293T cells to drive Aβ production and treated them with or without HUHS015 (Figure 1q). Strikingly, ALKBH3 inhibition markedly reduced Aβ levels (Figure 1r, Figure S4h), establishing pharmacological ALKBH3 blockade as a viable strategy to counteract Aβ pathology.

### 2.3 Alkbh3 Loss Rescues 5xFAD Cognition and Restores Hippocampal Calcium Dynamics

**FIGURE 2:**
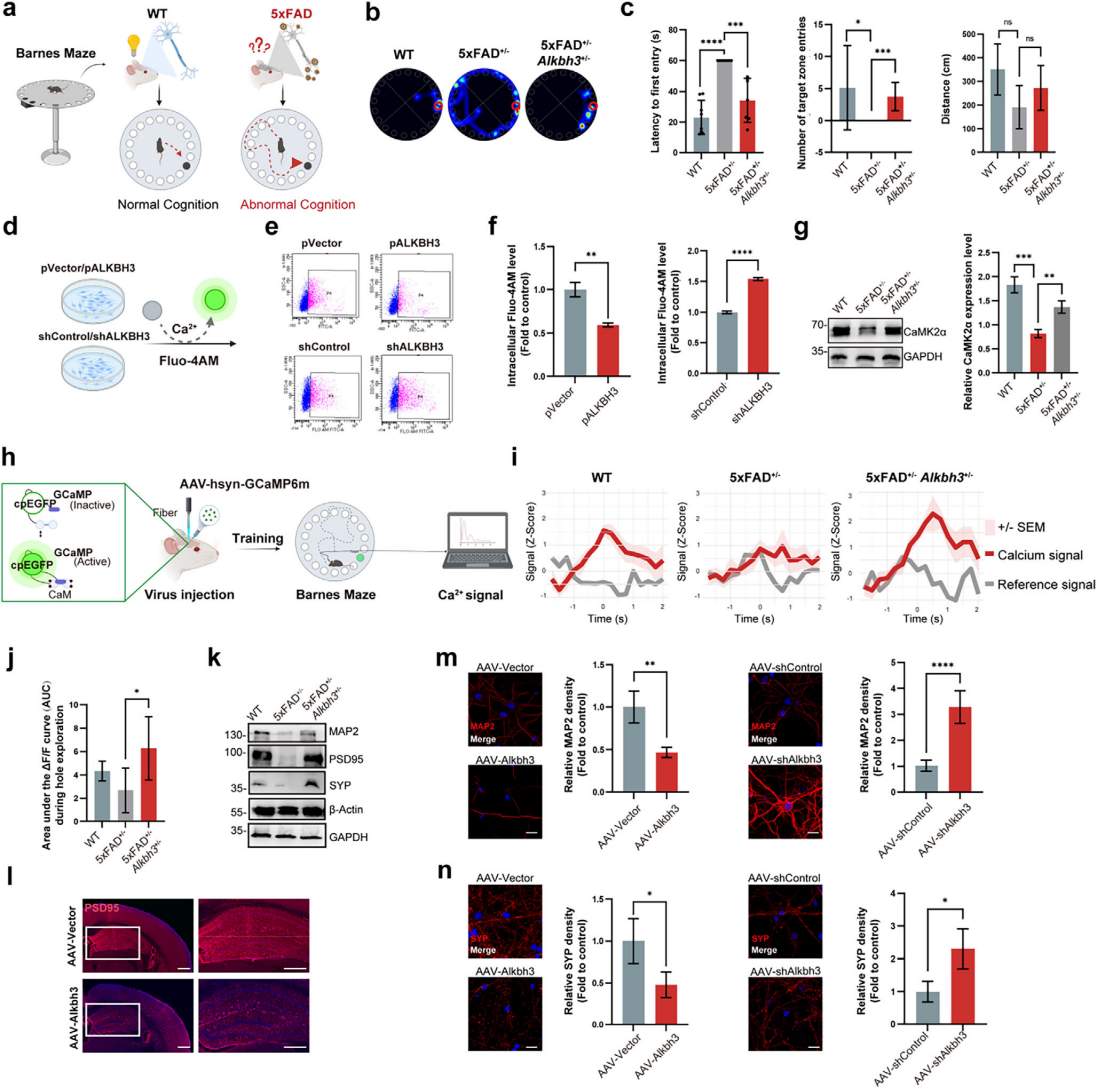
ALKBH3 depletion rescues spatial memory and calcium dysregulation in 5xFAD mice. (a) Barnes maze paradigm for assessing AD‐related cognitive deficits; (b) representative search path heatmaps of WT, 5xFAD^+/−^, and 5xFAD^+/−^ *Alkbh3* ^+/−^ mice (8 months; n = 7–8/group); (c) latency to first entry, number of target zone entries and distance of WT, 5xFAD^+/−^, and 5xFAD^+/−^ *Alkbh3* ^+/−^ mice (8 months; *n* = 7–8/group); (d) fluo‐4AM‐labeled primary neuron activation assay; (e) flow cytometry (FACS) density plots of calcium flux (488 nm) in ALKBH3‐modulated neurons; (f) quantified Fluo‐4AM intensity (*n* = 10 000 events/replicate); (g) CaMK2α versus Gapdh levels in 5xFAD^+/−^ *Alkbh3^+/−^* versus 5xFAD^+/−^ and WT controls (WB); (h) hippocampal GCaMP6m fiber photometry setup during maze navigation; (i) quantification of peak ΔF/F values during hole exploration; (j) area under the curve (AUC) analysis of fluorescence signals during hole exploration, providing a quantitative measure of ΔF/F activity; (k) synaptic protein levels in 5xFAD^+/−^ *Alkbh3^+/−^* versus control hippocampi (WB; Gapdh/β‐Actin loading controls); (l) PSD95 (red) in AAV‐Alkbh3 versus AAV‐Vector hippocampi (IF, 6 months; scale bar: 0.5 mm); (m) IF images (top) and quantifications (bottom) of MAP2 in Alkbh3‐modulated neurons (scale bar: 20 µm); (n) IF images (top) and quantifications (bottom) of SYP in Alkbh3‐modulated neurons (scale bar: 20 µm); representative images shown for IF (blue for DAPI) and WB; WB images were analyzed by ImageJ software (v1.53); two‐tailed unpaired t‐test; data: mean ± SEM; *n* = 3 biological replicates unless noted; *p < 0.05, **p < 0.01, ***p < 0.001, ****p < 0.0001.

Having established that ALKBH3 inhibition reduces Aβ pathology, we next tested whether this intervention could also ameliorate cognitive deficits, a core AD feature linked to Aβ accumulation [5]. We employed the Barnes maze, a validated spatial memory test for AD mouse models, where mice locate a hidden escape box among 20 possible holes on a brightly lit circular platform using distal visual cues (Figure 2a) [33, 34]. Performance was quantified by target zone entries and dwell time during 1‐minute trials, with impaired spatial learning serving as a hallmark of 5xFAD cognitive deficits [33]. 8‐month‐old 5xFAD^+/−^ mice displayed dispersed exploration, whereas age‐matched WT and 5xFAD^+/−^ *Alkbh3* ^+/−^ mice oriented directly toward the target hole (Figure 2b, Figure S5a). 5xFAD^+/−^ mice showed impaired spatial memory (increased latency, reduced target exploration) without locomotor deficits (unchanged path length; Figure 2c, Figure S5b). Genetic reduction of *Alkbh3* (5xFAD^+/−^ *Alkbh3* ^+/−^) fully rescued these deficits, restoring latency and exploration to WT levels without affecting locomotion (Figure 2c, Figure S5b). These findings demonstrate that reduced Alkbh3 expression rescues the spatial memory deficits in 5xFAD mice (Figure 2b,c, Figure S5a,b).

Having observed the rescue of hippocampus‐dependent memory, we sought to investigate the underlying cellular physiology. Synaptic plasticity, the cellular substrate of learning and memory, is fundamentally regulated by intracellular calcium signaling [35, 36, 37]. Furthermore, calcium dysregulation is a well‐documented early and pronounced phenotype in the 5xFAD model, manifesting as elevated baseline calcium and impaired neuronal excitability prior to overt cell death [35]. We therefore hypothesized that the cognitive rescue afforded by ALKBH3 reduction might involve the normalization of neuronal calcium homeostasis.

To first determine whether ALKBH3 can directly modulate neuronal calcium dynamics, we examined its effects in SH‐SY5Y neuroblastoma cells. Overexpression (pALKBH3) or knockdown (shALKBH3) of ALKBH3 in SH‐SY5Y neuroblastoma cells (vs. controls pVector, shControl), constructs validated to modulate ALKBH3 and m1A levels (WB, LC‐MS/MS, Figure S5c,d), altered intracellular calcium dynamics upon activation, as measured by Fluo‐4AM (Figure 2d–f) [38, 39]. FACS analysis confirmed that elevated ALKBH3 dampened calcium responses (Figures 2e,f). This effect was pharmacologically recapitulated using the ALKBH3 inhibitor HUHS015 [31, 40], which enhanced calcium signaling in both undifferentiated and differentiated SH‐SY5Y cells (Figure S5e), demonstrating cell‐state‐independent regulation. Together, these findings establish ALKBH3 as a direct modulator of calcium dynamics.

To assess calcium dynamics in the intact cognitive circuit of living mice, we first noted that CaMK2α, a calcium/calmodulin‐dependent kinase critical for synaptic plasticity and memory formation [41, 42, 43], was elevated in 5xFAD^+/−^ *Alkbh3* ^+/−^ (Figure 2g), suggesting compensatory potentiation of calcium signaling. We therefore monitored hippocampal activity during Barnes maze navigation. We delivered AAV‐GCaMP6m, a calcium‐sensitive biosensor whose fluorescence intensity rises upon calcium binding [44, 45], to the dorsal hippocampus for in vivo imaging (Figure 2h). Using fiber photometry, we then recorded and quantified calcium‐dependent fluorescence in the hippocampus of WT, 5xFAD^+/−^, and 5xFAD^+/−^ *Alkbh3* ^+/−^ mice as they performed hole‐exploration tasks in the Barnes maze (Figure 2h, Figure S5f). Our analysis revealed a severe impairment in hippocampal calcium signaling in 5xFAD^+/−^mice. Z‐score analysis showed these calcium transients were severely blunted, exhibiting no significant deviation from baseline (Figure 2i). This deficit was quantified as a marked attenuation in ΔF/F responses (2.68 ± 1.92) compared to WT controls (4.35 ± 0.85), consistent with reduced spatial memory (Figure 2b,c,j). Strikingly, genetic ablation of *Alkbh3* fully rescued this dysfunction. The calcium dynamics of 5xFAD^+/−^ *Alkbh3* ^+/−^ mice were indistinguishable from WT mice, with robust, well‐defined transients visible in both z‐score traces and a significantly restored ΔF/F magnitude (6.28 ± 2.70; *p* < 0.05 vs. 5xFAD^+/−^) (Figure 2i,j). The complete restoration of calcium signaling dovetailed with recovered cognitive performance. Together, these findings demonstrate that *Alkbh3* deficiency in 5xFAD^+/−^ mice normalizes hippocampal calcium dynamics and rescues cognitive function, establishing ALKBH3 as a critical regulator of synaptic activity and memory.

### 2.4 ALKBH3 Regulates Synaptic and Dendritic Integrity

We next assessed whether the functional rescue in 5xFAD^+/−^ *Alkbh3* ^+/−^ mice correlated with ALKBH3‐dependent structural remodeling. WB analysis revealed significant increases in key neuronal markers in 5xFAD^+/−^ *Alkbh3* ^+/−^ compared to 5xFAD^+/−^ controls (Figure 2k), including: MAP2 (microtubule‐stabilizing protein essential for dendritic branching) [46], synaptophysin (SYP, presynaptic vesicle protein reflecting synaptic vesicle pools) [22, 47], and PSD95 (postsynaptic scaffold critical for excitatory synapse stability) [48, 49]. Similar directional changes in these markers were also noted in *Alkbh3*‐overexpressing and knockout mice by IF, with a concomitant shift in the m1A/A ratio as confirmed by LC‐MS/MS (Figure 2l, Figure S6a,b). Taken together, these data suggest conserved ALKBH3‐mediated regulation of neuronal structure independent of Aβ pathology.

To investigate these effects further, we employed complementary cellular models. In primary neurons, AAV‐mediated ALKBH3 overexpression (AAV‐ALKBH3) reduced both MAP2^+^ dendritic complexity and SYP^+^ puncta density, while *ALKBH3* knockdown (AAV‐shALKBH3) increased these structural parameters versus control (AAV‐Vector, AAV‐shControl) (Figure 2m,n, Figure S6c)—changes accompanied by corresponding alterations in m1A levels (LC‐MS/MS) (Figure S6d). These bidirectional effects were replicated in SH‐SY5Y neuroblastoma cells, with parallel morphological and m1A modifications (Figures S5d and S6e,f). The conserved structural regulation across neuronal cell types and model systems, establishes ALKBH3 as a key coordinator of neuronal morphology, likely operating through regulating fundamental cellular pathways.

### 2.5 ALKBH3 is Associated With Mitochondria and Associates With Outer Membrane Protein

**FIGURE 3:**
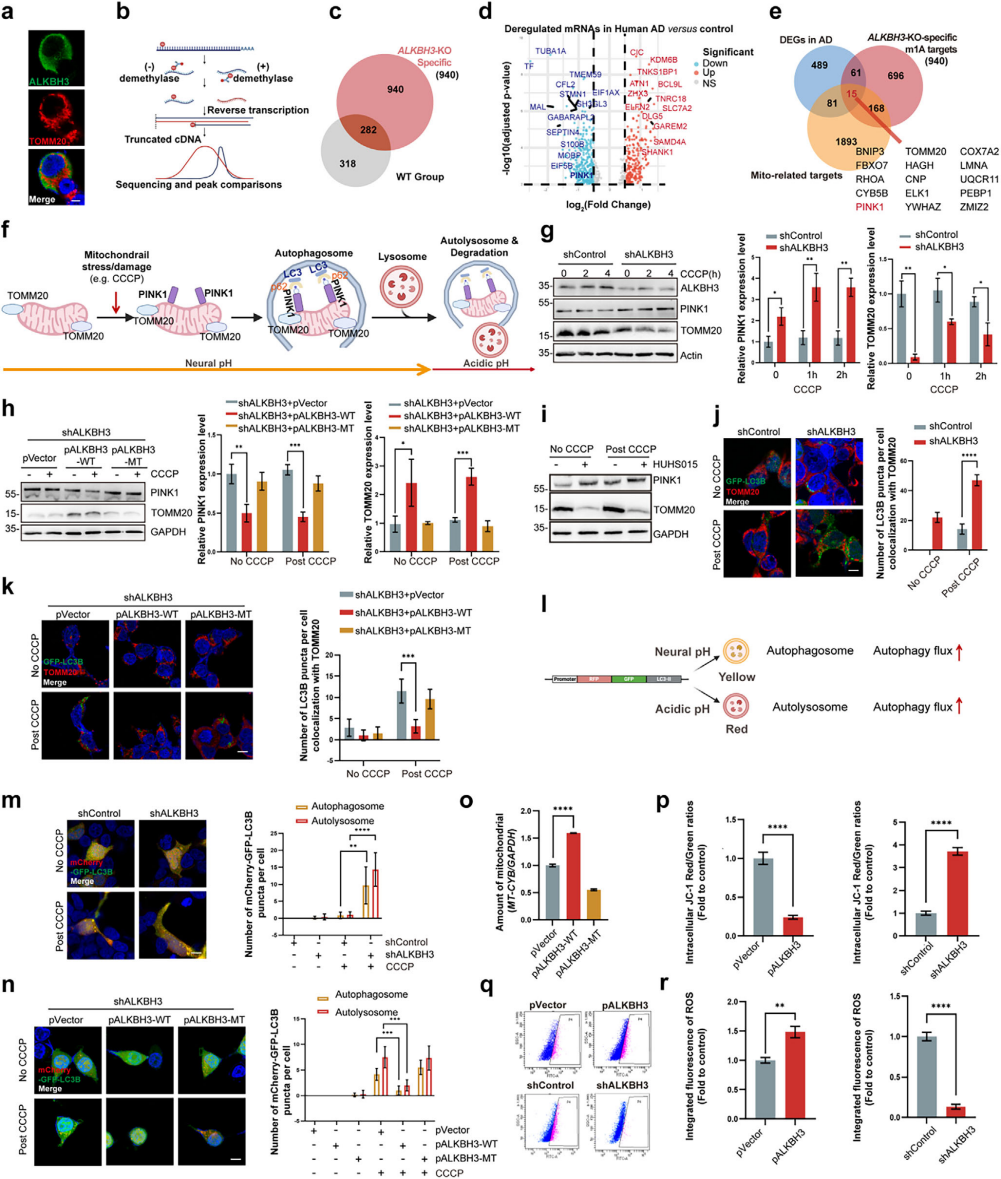
ALKBH3 regulates m1A‐dependent mitophagy to maintain mitochondrial homeostasis. (a) ALKBH3 (green) mitochondrial colocalization in HEK293T cells (TOMM20, red; IF; scale bar: 10 µm); (b) m1A‐ID‐seq workflow for transcriptome‐wide m1A mapping [27]; (c) identification of m1A targets regulated by ALKBH3 (*ALKBH3*‐KO‐specific m1A targets); (d) Volcano plot of differentially expressed genes (DEGs) in human AD patients versus control synaptosomes; (e) Venn diagram of DEGs in AD, *ALKBH3*‐KO‐specific m1A targets and mito‐related targets; mito‐related targets: mitochondria‐associated genes that were identified by retrieving gene ontology terms containing the keywords ‘mitochondrion’ or ‘mitochondria’; (f) PINK1‐dependent mitophagy pathway. (i) basal conditions: PINK1 is constitutively degraded in healthy mitochondria (TOMM20 marker). (ii) Mitochondrial stress (e.g., CCCP mitophagy inducer): PINK1 accumulates on the outer membrane, initiating quality control. (iii) PINK1 recruits protein adaptors (p62, LC3B), forming autophagosomes. (iv) Autophagosome‐lysosome fusion generates acidic autolysosomes, degrading mitochondria; (g) mitophagy in shALKBH3: representative images and quantification of PINK1 and TOMM20 expression by WB in shControl versus shALKBH3 HEK293T cells (± CCCP); (h) mitophagy ALKBH3 rescue: representative images and quantification of PINK1 and TOMM20 expression by WB in shALKBH3 cells transfected with vector, WT (pALKBH3‐WT) or mutant (pALKBH3‐MT) HEK293T cells (± CCCP); (i) PINK1 and TOMM20 in cells treated with HUHS015 or DMSO control (WB; ± CCCP); (j) (i) GFP‐LC3B (green) and TOMM20 (red) in shControl versus shALKBH3. (IF images; ± CCCP; scale bar: 10 µm); (ii) quantifications of GFP‐LC3B and TOMM20 colocalized puncta/cell (n = 6/group); (k) (i) GFP‐LC3B (green) and TOMM20 (red) in rescue experiments with pALKBH3‐WT/MT in shALKBH3 (IF images; ± CCCP; scale bar: 10 µm); (ii) Quantifications of GFP‐LC3B and TOMM20 colocalized puncta/cell (n = 6/group); (l) dual‐color reporter mCherry‐GFP‐LC3B construct with pH‐sensitive GFP permits stage discrimination. Fluorescence readouts: autophagosomes (GFP+mCherry+, yellow) and autolysosomes (mCherry+GFP‐, red); (m) (i) mCherry‐GFP‐LC3B puncta in shControl versus shALKBH3 (IF images; ± CCCP; scale bar: 10 µm); (ii) quantifications of mCherry‐GFP‐LC3B puncta/cell (*n* = 6/group); (n) (i) rescue experiments with pALKBH3‐WT/MT in shALKBH3 (IF images; ± CCCP; scale bar: 10 µm); (ii) Quantifications of mCherry‐GFP‐LC3B puncta/cell (n = 6/group); (o) Mitochondrial DNA abundance quantified through mitochondrial cytochrome b (*MT‐CYB*)/nuclear DNA (*GAPDH*) (qPCR); (p) JC‐1 assay measuring mitochondrial membrane potential (see methods) (10 000 events/replicate) in ALKBH3‐modulated SH‐SY5Y cells; (q) FACS results of MitoSOX Green fluorescence (488 nm) in ALKBH3‐modulated SH‐SY5Y cells; (r) quantification of mitochondrial reactive oxygen species (ROS) measured through MitoSOX (10 000 events/replicate); representative images shown for IF (blue for DAPI) and WB; two‐tailed unpaired *t*‐test; data: mean ± SEM; *n* = 3 biological replicates unless noted; *p < 0.05, **p < 0.01, ***p < 0.001, ****p < 0.0001.

To identify ALKBH3's regulatory pathway, we performed targeted interactome analysis using Flag‐ALKBH3 affinity purification mass spectrometry (Figure S7a). Gene Ontology analysis revealed striking enrichment of mitochondrial‐associated proteins among ALKBH3 interactors (Figure S7b and Table S1), prompting us to investigate a potential mitochondrial role. IF imaging confirmed partial co‐localization of ALKBH3 with mitochondria—marked by TOMM20, a canonical outer mitochondrial membrane protein—(Figure 3a) [50]. To biochemically validate this association, we performed subcellular fractionation, which revealed ALKBH3 enrichment in cytoplasmic and mitochondrial fractions (Figure S7c,d). Super‐resolution microscopy confirmed the close apposition of ALKBH3 with TOMM20 at the mitochondrial surface (Figure S7e). Furthermore, co‐immunoprecipitation experiments using FLAG‐tagged ALKBH3 demonstrated a direct interaction with endogenous TOMM20 (Figure S7f). To determine ALKBH3's submitochondrial topology, we performed a digitonin fractionation assay on purified mitochondria. Digitonin selectively solubilizes the cholesterol‐rich outer mitochondrial membrane, releasing only outer membrane associated proteins [51, 52]. Following digitonin treatment, ALKBH3 and the outer membrane marker TOMM20 were reduced from the mitochondrial pellet, while the matrix protein HSP60 remained intact (Figure S7g). Collectively, these data indicate that a considerable fraction of ALKBH3 is physically associated with the mitochondrial outer membrane.

Given ALKBH3's reported activity as a DNA repair enzyme targeting 1‐methyladenine (m1dA) [53], we tested its potential interaction with DNA. Using our sensitive LC‐MS/MS capable of detecting low‐abundance DNA modifications such as N6‐methyl‐2'‐deoxyadenosine (m6dA), we could not detect m1dA in DNA from cells under our physiological culture conditions (Figure S7h). Consistent with the absence of this substrate, FLAG‐tagged ALKBH3 did not enrich mtDNA in pull‐down assays (Figure S7i). These data indicate that the mitochondrial phenotypes we observe are unlikely to stem from ALKBH3's DNA demethylase activity.

We therefore asked whether ALKBH3 regulates mitochondrial processes through its canonical m1A‐dependent RNA demethylase activity [54]. m1A‐ID‐seq analysis of WT HEK293T versus *ALKBH3* knockout cells (a transcriptome‐wide, single‐nucleotide resolution m1A mapping method) identified 940 m1A sites that are removed by ALKBH3 (Figure 3b,c, Table S1) [27]. These ALKBH3‐specific m1A targets were enriched for mitochondrial pathways (Figure S7j, Table S1), also suggesting a link between ALKBH3 epitranscriptomic function and mitochondrial regulation.

Before probing ALKBH3's mitochondrial mechanisms, we first decided to test its disease relevance by analyzing differential gene expression in synaptosome transcriptome datasets obtained from unaffected individuals and AD patients [55]. Our analysis identified dysregulated genes in AD (log_2_FC > |0.5|, *p* < 0.01; Figure 3d, Table S2). To focus on mitochondrial pathways, we intersected three gene sets: human AD DEGs differentially expressed genes in AD (Table S2), MITO‐related genes (mitochondrial pathways, Table S1), and our *ALKBH3*‐KO‐specific m1A targets (from HEK293T m1A‐ID‐seq, Table S1). This triple intersection yielded 15 transcripts (Figure 3e). We prioritized PINK1, the master regulator of mitophagy, based on its established central role in neuronal mitochondrial quality control [20, 50, 56], its significant downregulation in human AD transcriptomes (Figure S7k), and its identification as a high‐confidence m1A target in an independent transcriptome‐wide mapping study (see Methods) [57].

Given that impaired mitochondrial clearance in neurons leads to synaptic dysfunction and neurodegeneration [2, 20, 58], and that defective mitophagy is a well‐established cause driving neuronal toxicity through mitochondrial dysfunction [20, 59], we reasoned that ALKBH3 regulation of PINK1 could be a mechanism linked to amyloid AD pathogenesis. Supporting this connection, PINK1 expression is reduced in AD patient samples (Figure 3d), and we identified it as a direct m1A target of ALKBH3 (Figure 3e, Table S1) [55, 60]. We therefore hypothesized that ALKBH3 modulates mitophagy, potentially through PINK1. To establish a mechanistic foundation, we next leveraged cellular models to determine whether ALKBH3 regulates mitophagy.

### 2.6 ALKBH3 Regulates Mitophagy via m1A Demethylase Activity

Mitophagy is initiated when damaged mitochondria (e.g., carbonyl cyanide 3‐chlorophenylhydrazone [CCCP] treated [50]) accumulate PINK1, which recruits downstream effectors (p62, LC3B‐II) to target these organelles for degradation (Figure 3f) [61]. WB analysis revealed that ALKBH3 constitutively suppresses this quality control pathway. LC‐MS/MS analysis confirmed alterations in m1A/A levels in HEK293T cells following both *ALKBH3* knockdown (shALKBH3) and overexpression (pALKBH3‐WT) (Figure S8a). In CCCP‐treated HEK293T cells, *ALKBH3* knockdown (shALKBH3) enhanced PINK1 accumulation and reduced TOMM20 levels, indicative of accelerated mitochondrial clearance, while concurrently increasing LC3B‐II lipidation and p62 degradation—hallmarks of autophagosome formation and lysosomal flux (Figure 3g, Figure S8b). Conversely, ALKBH3 overexpression (pALKBH3‐WT) suppressed these mitophagy markers, whereas the catalytically inactive mutant ALKBH3‐D193A (hereafter pALKBH3‐MT, validated by LC‐MS/MS in Figure S8a) had no effect [19, 62], establishing the essential role of m1A demethylation in mitophagy (Figure S8c). Genetic rescue experiments confirmed this specific mechanism, as only pALKBH3‐WT reversed the enhanced mitophagy phenotype in shALKBH3 cells (Figure 3h, Figure S8d). Furthermore, pharmacological inhibition of ALKBH3 with HUHS015 phenocopied genetic knockdown, increasing PINK1 levels and reducing TOMM20 even in basal conditions (Figure 3i, Figure S8e). Together, these results demonstrate that ALKBH3's m1A demethylase activity serves as a physiological brake on mitophagy, while its inhibition unleashes PINK1‐dependent mitochondrial quality control.

To corroborate our findings, we also combined imaging with quantitative assays. When applying the mitochondrial marker TOMM20 and a GFP‐LC3B reporter in IF experiments, we found that ALKBH3 knockdown (shALKBH3) led to increased numbers of GFP‐LC3B^+^ puncta co‐localizing with depolarized mitochondria (CCCP‐treated), indicating enhanced mitophagosome recruitment to damaged organelles (Figure 3j). Conversely, WT ALKBH3 overexpression (pALKBH3‐WT) reduced GFP‐LC3B/TOMM20 co‐localization, demonstrating that ALKBH3 actively suppresses mitophagy initiation (Figure S8f). We further discovered mechanistic m1A‐dependency in rescue experiments: only pALKBH3‐WT reversed the hyperactivated mitophagy phenotype of shALKBH3 cells, while the catalytically inactive mutant (pALKBH3‐MT) had no effect, implicating m1A demethylation in this regulation (Figure 3k).

To resolve ALKBH3's impact on later stages of mitophagy, we employed the mCherry‐GFP‐LC3B reporter system, a gold‐standard assay for tracking autophagy flux [63]. This system exploits pH‐sensitive GFP quenching, enabling detection of neutral autophagosomes with dual mCherry^+^GFP^+^ yellow signals and acidified autolysosomes as mCherry^+^GFP^−^ red puncta marks (Figure 3l) [63, 64]. Strikingly, CCCP‐treated ALKBH3‐deficient cells exhibited an 11‐fold increase in autophagosomes and a 14.3‐fold increase in autolysosomes compared to controls (Figure 3m), indicating enhanced initiation and completion of mitophagy. Conversely, pALKBH3‐WT suppressed both (Figure S8g). In rescue experiments, we confirmed that this regulation required catalytic activity: pALKBH3‐WT normalized autophagic flux in shALKBH3 cells, while the pALKBH3‐MT failed to inhibit either autophagosome formation or lysosomal fusion (Figure 3n).

Together, our findings demonstrate that ALKBH3 serves as a critical brake on mitophagy, operating through two distinct mechanisms: (1) suppression of autophagosome biogenesis and (2) inhibition of lysosomal targeting—both dependent on its m1A demethylase activity.

### 2.7 ALKBH3 Drives Mitochondrial Dysfunction Through Impaired Mitophagy

Next, we investigated ALKBH3s broader impact on mitochondrial homeostasis. qPCR analysis of mitochondrial DNA (mtDNA) revealed that overexpression of pALKBH3‐WT (but not pALKBH3‐MT) increased copy numbers of the mitochondrial mass marker cytochrome b (*MT‐CYB*) (Figure 3o) [65], reflecting defective mitochondrial clearance and accumulation of compromised mitochondria [66]. JC‐1 staining revealed that *ALKBH3* knockdown increased mitochondrial membrane potential (ΔΨm), as evidenced by an increased red/green fluorescence ratio, whereas *ALKBH3* overexpression decreased ΔΨm, reflected by a reduced red/green fluorescence ratio (Figure 3p). This depolarization was accompanied by elevated reactive oxygen species (ROS) in ALKBH3 overexpressing cells, as measured by MitoSOX, while shALKBH3 attenuated oxidative stress (Figure 3q,r). Additionally, oxygen consumption rate (OCR) measurements by Seahorse assay revealed that ALKBH3 overexpression enhanced basal respiration, maximal respiration, ATP production, and proton leak (Figure S8h), all of which are indicative of abnormal cellular respiration. Together, these data establish that ALKBH3 impairment triggers a cascade of mitochondrial pathologies: accumulation of depolarized organelles, oxidative damage, and metabolic inefficiency. This triad of dysfunction mirrors the bioenergetic failure observed in neurodegenerative disease [3, 67, 68].

### 2.8 ALKBH3 Suppression Rescues Mitophagy Defects

**FIGURE 4:**
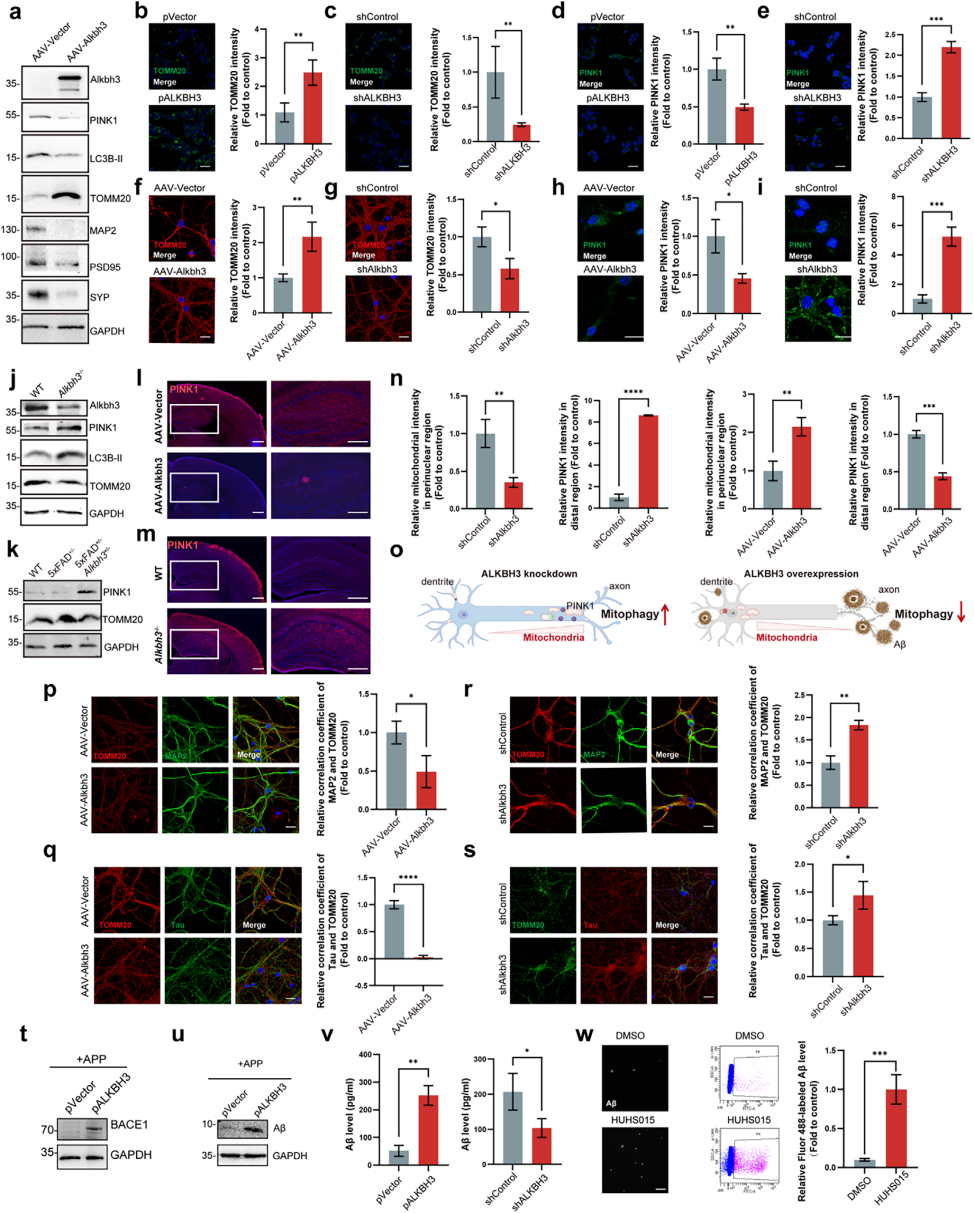
ALKBH3 regulates mitochondrial trafficking and PINK1 distribution in neurons. (a) AAV‐Alkbh3 expression in primary hippocampal neurons versus AAV‐Vector controls (WB; GAPDH loading control); (b) TOMM20 (mitochondria, green; *n* = 5/group) in pVector versus pALKBH3 in SH‐SY5Y cells (left: IF images, scale bar: 20 µm; right: quantification); (c) TOMM20 (mitochondria, green; *n* = 5/group) in shControl versus shALKBH3 in SH‐SY5Y cells (left: IF images, scale bar: 20 µm; right: quantification); (d) PINK1 (green; *n* = 5/group) in pVector versus pALKBH3 in SH‐SY5Y cells (left: IF images, scale bar: 20 µm; right: quantification); (e) PINK1 (green; *n* = 5/group) in shControl versus shALKBH3 in SH‐SY5Y cells (left: IF images, scale bar: 20 µm; right: quantification); (f) TOMM20 (mitochondria, red) in AAV‐Vector versus AAV‐ALKBH3 in primary hippocampal neurons (left: IF images, scale bar: 20 µm; right: quantification); (g) TOMM20 (mitochondria, red) in shControl versus shALKBH3 in primary hippocampal neurons (left: IF images, scale bar: 20 µm; right: quantification); h) (PINK1 (green) in AAV‐Vector versus AAV‐ALKBH3 in primary hippocampal neurons (left: IF images, scale bar: 20 µm; right: quantification); (i) PINK1 (green) in shControl versus shALKBH3 in primary hippocampal neurons (left: IF images, scale bar: 20 µm; right: quantification); (j) PINK1 and TOMM20 levels in WT versus *Alkbh3* ^−/−^ mice (WB); (k) PINK1 and TOMM20 levels in WT, 5xFAD^±^ and 5xFAD^+/−^ *Alkbh3^+/−^* mice (WB); (l) PINK1 expression (red, IF) in 6‐month‐old hippocampi injected with AAV‐Vector or AAV‐Alkbh3 (scale bar: 0.5 mm); (m) PINK1 expression (red, IF) in 6‐month‐old WT versus *Alkbh3* ^−/−^ hippocampi (scale bar: 0.5 mm); (n) quantification of mitochondria (TOMM20) and PINK1 abundance in perinuclear and distal regions, respectively (see methods); (o) schematic of ALKBH3 regulating neuronal mitochondria‐PINK1 distribution; (p) mitochondrial‐neurite co‐localization in AAV‐Vector versus AAV‐ALKBH3 in primary hippocampal neurons: left: IF images of TOMM20 (red)/MAP2 (green) (scale bar: 20 µm); right: Correlation coefficients (see methods); (q) Mitochondrial‐neurite co‐localization in AAV‐Vector versus AAV‐ALKBH3 in primary hippocampal neurons: left: IF images of TOMM20 (red)/Tau (green) (scale bar: 20 µm); right: correlation coefficients (see methods); (r) mitochondrial‐neurite co‐localization in shControl versus shALKBH3 in primary hippocampal neurons: left: IF images of TOMM20 (red)/MAP2 (green) (scale bar: 20 µm); right: correlation coefficients (see methods); (s) mitochondrial‐neurite co‐localization in shControl versus shALKBH3 in primary hippocampal neurons: left: IF images of TOMM20 (green)/Tau (red) (scale bar: 20 µm); right: correlation coefficients (see methods); (t) BACE1 protein levels in pVector versus pALKBH3 in SH‐SY5Y cells overexpressing APP (WB), with GAPDH as loading control; (u) Aβ protein levels in pVector versus pALKBH3 in SH‐SY5Y cells overexpressing APP (WB), with GAPDH as loading control; (v) ELISA quantification of secreted Aβ levels in pVector versus pALKBH3 in SH‐SY5Y cells overexpressing APP; (w) fluor 488‐labeled Aβ in DMSO versus HUHS015 in BV‐2 cells: (i) microscope image (scale bar: 60 µm); (ii) FACS sorting image; (iii) quantification of FACS sorting; representative images shown for IF (blue for DAPI) and WB; IF images were analyzed by ImageJ software (v1.53); two‐tailed unpaired *t*‐test; data: mean ± SEM; *n* = 3 biological replicates unless noted; *p < 0.05, **p < 0.01, ***p < 0.001, ****p < 0.0001.

To define ALKBH3's role in neuronal mitophagy, we examined key regulators of mitochondrial quality control in both primary hippocampal neurons and SH‐SY5Y cells. While ALKBH3 overexpression suppressed mitophagy (reducing levels of PINK1 and autophagosome marker LC3B‐II while increasing levels of mitochondrial protein TOMM20), *ALKBH3* knockdown enhanced these processes (Figure 4a–i, Figure S9a,b). When we then inhibited proteasomal degradation with MG132 [69], we found that PINK1 protein levels remained low in ALKBH3‐overexpressing cells and high in *ALKBH3*‐knockdown cells despite MG132 treatment (Figure S9c, d), demonstrating that ALKBH3‐mediated regulation of PINK1 is independent of protein degradation.

The effect of ALKBH3 on mitophagy markers was conserved in 5xFAD mice: *Alkbh3* ^−/−^ hippocampi showed robust mitophagy activation, and critically, this same rescue of mitochondrial clearance occurred in 5xFAD^+/−^ *Alkbh3* ^+/−^ mice compared to 5xFAD^+/−^ controls (Figure 4j–m). Together, these results demonstrate that ALKBH3 acts as a master brake on neuronal mitophagy, and that its inhibition restores the PINK1‐dependent clearance of damaged mitochondria that is defective in AD pathology.

### 2.9 ALKBH3 Disrupts Mitochondrial Trafficking

Next, we investigated how ALKBH3 impacts mitochondrial trafficking, a process functionally linked to degradation [70, 71]. In neurons, a compartmentalized quality control system ensures that healthy mitochondria are preferentially transported to synapses to meet local energy demands, and that damaged organelles are transported retrogradely to the soma for degradation [70, 71, 72]. We found that increased ALKBH3 seemed to trap mitochondria: levels of perinuclear (near nucleus) TOMM20^+^ mitochondria increased in IF assays (see methods) (Figure 4n,o), reflecting impaired clearance and accumulation of dysfunctional organelles. Conversely, *ALKBH3* knockdown enhanced distal PINK1^+^ puncta (Figure 4n,o), indicating improved synaptic mitophagy, consistent with PINK1's role in maintaining functional mitochondria at synapses [73, 74]. In addition, ALKBH3 overexpression reduced colocalization of TOMM20/MAP2 and TOMM20/Tau (Figure 4p,q), demonstrating that mitochondria accumulate in cell bodies rather than synapses, while *ALKBH3* knockdown increased mitochondrial distribution to axons and dendrites (Figure 4r,s), indicative of improved synaptic energy supply.

These findings pinpoint ALKBH3 as a master regulator of mitochondrial homeostasis, whose overexpression starves synapses of functional mitochondria while accumulating damaged organelles. <mark>This failure in mitochondrial quality control provides a direct mechanistic basis for investigating its downstream consequences on core AD pathologies, including Aβ metabolism.

### 2.10 ALKBH3‐Driven Mitochondrial Dysfunction Exacerbates Aβ Pathology via BACE1 Upregulation and Impaired Microglial Clearance

Having established that ALKBH3 impairs mitophagy and causes mitochondrial dysfunction (Figure 3, 4), we next sought to connect this defect to the increased Aβ deposition observed in our models (Figure 1). Mitochondrial dysfunction promotes Aβ accumulation through two well‐established pathways. First, mitochondrial‐derived reactive oxygen species (ROS) induce oxidative stress that activates the BACE1 (β‐site APP‐cleaving enzyme 1) enzyme [75], which upregulates this rate‐limiting enzyme in amyloidogenic APP processing and shifts APP cleavage toward Aβ production [76, 77]. Second, impairment of microglial phagocytic clearance reduces Aβ removal [78]. To determine whether ALKBH3 influences these downstream pathways, we first measured BACE1 levels in ALKBH3‐modulated SH‐SY5Y cells. ALKBH3 overexpression elevated BACE1 protein (Figure 4t), while HUHS015‐mediated inhibition of ALKBH3 or mitophagy induction (CCCP) reduced it (Figure S10a), indicating that ALKBH3 regulates Aβ production via BACE1‐dependent processing. In APP‐overexpressing cells, ALKBH3 overexpression increased Aβ levels, as identified by WB, ELISA, and IF. (Figure 4u,v, Figure S10b), further confirming its role in amyloidogenic pathway activation.

To also assess Aβ clearance, we used BV‐2 microglial cells, a well‐established microglia cell model [79]. Cells were incubated with oligomeric Aβ42 labeled with HiLyte Fluor 488, a validated probe for assessing microglial Aβ uptake, where fluorescence intensity directly correlates with internalized Aβ load [80]. Microscopic analysis and quantification revealed that ALKBH3 inhibition significantly increased the number of fluorescent Aβ42 puncta per cell, indicating enhanced uptake of pre‐aggregated, HiLyte Fluor 488‐labeled Aβ42 by microglia (Figure 4w). These results demonstrate that inhibition of ALKBH3 promotes both Aβ42 internalization and, consequently, its removal by microglial cells.

Together, these data show that ALKBH3 influences both Aβ production and clearance, linking ALKBH3‐driven mitochondrial impairment to both arms of Aβ dysregulation in AD: enhancing Aβ production via BACE1 and impairing its microglial clearance.

Given the established synergy between Aβ and hyperphosphorylated Tau (p‐Tau) pathologies in AD [81], we also tested whether ALKBH3 could influence p‐Tau. We distinguished between physiological, non‐toxic microtubule‐stabilizing Tau and its pathological, hyperphosphorylated form (p‐Tau), which is prone to aggregation and is a core neurotoxic hallmark of AD [82]. To specifically assess p‐Tau, we expressed the P301L Tau mutant in SH‐SY5Y cells; this mutation accelerates Tau hyperphosphorylation and recapitulates key features of Tau pathology independent of Aβ [83]. In this model, ALKBH3 knockdown reduced p‐Tau levels, while ALKBH3 overexpression increased them (Figure S10c). These findings indicate that ALKBH3 also promotes Tau hyperphosphorylation independently of its effects on Aβ —forming an “Aβ‐Tau cascade” that jointly drives disease progression, positioning it as a regulator of multiple key pathways in AD progression [81].

### 2.11 ALKBH3 Regulates PINK1 Through m1A‐Dependent mRNA Decay

**FIGURE 5:**
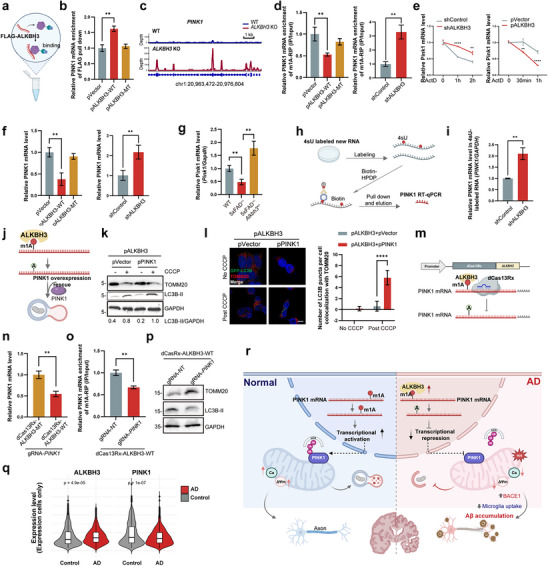
ALKBH3 regulates PINK1 through m1A‐dependent mRNA modification. (a) FLAG‐IP schematic for ALKBH3‐PINK1 mRNA interaction; (b) PINK1 mRNA binding by ALKBH3‐WT versus ‐MT (both FLAG‐tagged); (c) m1A peak distribution across PINK1 mRNA in WT versus *ALKBH3* knockout HEK293T cells; (d) m1A‐RIP‐RT‐qPCR showing PINK1 mRNA enrichment with ALKBH3‐WT versus ALKBH3‐MT and shALKBH3 versus shControl, *n* = 3/group; (e) quantitative RT‐PCR of PINK1 mRNA in pVector versus pALKBH3 and in shControl versus shALKBH3 in HEK293T cells treated with actinomycin D (ActD) at the indicated times; (f) PINK1 transcript levels after m1A RNA immunoprecipitation (m1A‐RIP and RT‐qPCR); (g) Pink1 transcript levels in WT, 5xFAD^±^ and 5xFAD^+/−^ *Alkbh3^+/−^* mice by RT‐qPCR; (h) 4sU labeling strategy of nascent RNA recovery for ALKBH3‐dependent PINK1 mRNA transcription analysis; (i) newly transcribed PINK1 mRNA levels after shALKBH3 versus shControl (RT‐qPCR); (j) PINK1 rescue strategy in ALKBH3 overexpressing cells; (k) LC3B‐II and TOMM20 levels with and without PINK1 rescue in ALKBH3 overexpressing HEK293T cells (WB); (l) GFP‐LC3B (green)/TOMM20 (red) colocalization with and without PINK1 rescue in HEK293T cells (left: IF images; ± CCCP; scale bar: 10 µm; right: quantifications; *n* = 6); (m) CRISPR‐dCas13Rx‐ALKBH3 system for targeted m1A demethylation [62]; n) PINK1 mRNA levels after dCas13Rx‐ALKBH3‐WT versus‐MT with gRNA‐PINK1 targeting PINK1 mRNA; (o) PINK1 mRNA enrichment following m1A‐RIP after dCas13Rx‐ALKBH3‐WT targeted demethylation with gRNA‐PINK1 versus gRNA‐NT control (RT‐qPCR); (p) LC3B‐II and TOMM20 levels in dCas13Rx‐ALKBH3‐WT and CCCP treated HEK293T cells (WB); (q) violin plots quantifying expression levels of ALKBH3 and PINK1 of single‐cell RNA‐seq data from human cortical hippocampi with and without AD pathology (two‐sided Wilcoxon rank‐sum test, *p* = 4.9 × 10^−5^ and p = 1 × 10^−7^, respectively); (r) ALKBH3‐m1A‐PINK1‐mitophagy model of AD pathogenesis. Left: normal state: balanced ALKBH3 activity maintains m1A methylation (red dots) and PINK1 transcript levels, supporting mitophagy and ROS clearance. Right: pathogenic ALKBH3 overexpression: excessive m1A demethylation reduces PINK1 transcription, causing mitophagy impairment leading to damaged mitochondria accumulation, ROS overload, and increased Aβ plaque accumulation and neuronal damage; Representative images are shown for IF (blue for DAPI) and WB; WB images were analyzed by ImageJ software (v1.53); two‐tailed unpaired *t*‐test; data: mean ± SEM; *n* = 3 biological replicates unless noted; *p < 0.05, **p < 0.01, ***p < 0.001, ****p < 0.0001.

Having delineated the downstream consequences of ALKBH3 overexpression on Aβ, we returned to the upstream molecular event: how does ALKBH3 suppress mitophagy? We hypothesized that it directly targets a critical regulator of the pathway, such as PINK1, through its m1A demethylase activity. First, we asked whether ALKBH3 directly interacts with PINK1 mRNA. In RNA immunoprecipitation experiments, we found that FLAG‐tagged ALKBH3‐WT specifically enriched PINK1 mRNA >1.5‐fold compared to controls, while the catalytically inactive ALKBH3‐MT showed no enrichment (Figure 5a,b). These data support that ALBH3 directly interacts with PINK1 mRNA, and that target recognition is dependent on ALKBH3's m1A catalytic domain. To validate ALKBH3 regulation of m1A on PINK1 mRNA, as revealed by our m1A‐ID‐seq analysis (Figures 3c,5c, Table S1), we focused on the m1A peak region that contained the GA‐rich canonical m1A consensus motif [27]. In m1A‐RIP‐RT‐qPCR analysis of this specific region, we found that overexpression of ALKBH3‐WT reduced PINK1 m1A levels by 50%, while the ALKBH3‐MT had no significant effect (Figure 5d). Conversely, ALKBH3 knockdown was associated with increased PINK1 mRNA m1A levels >3‐fold (Figure 5d).  Having established ALKBH3's direct regulation of PINK1 mRNA m1A, we investigated the spatial and mechanistic consequences. Fluorescence in situ hybridization (FISH) combined with IF revealed that PINK1 mRNA puncta co‐localize with ALKBH3 and TOMM20 on the mitochondrial surface (Figure S11a). Subcellular fractionation further demonstrated that ALKBH3 inhibition specifically increased PINK1 mRNA abundance in the mitochondrial fraction (Figure S11b), indicating regulation occurs preferentially at this location.

We next sought to determine the functional consequence of m1A demethylation on PINK1 mRNA. As m1A can influence various aspects of RNA metabolism, including translation and stability [84, 85], we asked whether ALKBH3 affects PINK1 mRNA half‐life. We performed Actinomycin D (ActD) chase assays, which inhibit transcription to allow direct measurement of mRNA decay. ALKBH3 overexpression significantly reduced, while ALKBH3 knockdown increased, the half‐life of PINK1 mRNA (Figure 5e). This demonstrates that m1A demethylation by ALKBH3 destabilizes PINK1 transcripts. Consistent with this post‐transcriptional mechanism, ALKBH3‐WT overexpression reduced steady‐state PINK1 mRNA levels to 38%, while the catalytically inactive ALKBH3‐MT showed no significant effect. Conversely, ALKBH3 knockdown increased PINK1 mRNA 2‐fold (Figure 5f), confirming bidirectional, catalysis‐dependent regulation of PINK1 transcript abundance. Critically, our data support that this regulatory mechanism also operates in vivo and has direct relevance to AD pathology: hippocampal PINK1 mRNA levels were reduced by 50% in 8‐month‐old 5xFAD^+/−^ mice compared to WT controls, while genetic ablation of *Alkbh3* in 5xFAD^+/−^ mice (5xFAD^+/−^ *Alkbh3* ^+/−^) restored PINK1 expression to >3‐fold above 5xFAD^+/−^ levels (Figure 5g). These results establish ALKBH3‐mediated m1A demethylation as a direct regulator of PINK1 transcripts across cellular and animal models, with therapeutic implications for reversing PINK1 deficiency in AD.

Given that the role of m1A in transcriptional regulation is not defined, we also employed 4‐thiouridine (4sU) metabolic labeling—a method that incorporates 4sU into newly synthesized RNA, enabling its isolation and thus providing a snapshot of transcription that is less confounded by steady‐state mRNA stability (Figure 5h) [32, 86]. This approach revealed that *ALKBH3* knockdown increased newly synthesized PINK1 transcripts by ∼2‐fold (Figure 5i). However, while 4sU labeling primarily reflects transcriptional output, the results could potentially also be influenced by the stabilization of nascent transcripts. Consequently, while the 4sU data suggest a role for ALKBH3 in modulating nuclear PINK1 RNA metabolism, the clearly established mechanism is the m1A‐dependent reduction in PINK1 mRNA stability in the cytoplasmic/mitochondrial compartment.

### 2.12 Targeted PINK1 Rescue Reverses ALKBH3‐Mediated Mitophagy Suppression

To establish whether ALKBH3‐mediated inhibition of mitophagy operates specifically through PINK1 suppression, we performed rescue experiments in ALKBH3‐overexpressing cells (Figure 5j–l). We found that PINK1 overexpression reversed the mitophagy blockade caused by ALKBH3, as evidenced by increased LC3B‐II levels and decreased TOMM20 accumulation (Figure 5k). IF further confirmed enhanced LC3B^+^ puncta co‐localizing with mitochondria (Figure 5l), demonstrating that PINK1 restoration in and of itself is sufficient to counteract ALKBH3's inhibitory effects.

To determine whether m1A demethylation of PINK1 mRNA specifically drives mitophagy suppression (as opposed to indirect effects of ALKBH3), we developed a targeted demethylation system [62]. We engineered a dCas13Rx‐ALKBH3 system, fusing catalytically active (ALKBH3‐WT) or inactive (ALKBH3‐MT) domains to dCas13Rx (dCas13Rx‐ALKBH3‐WT and dCas13Rx‐ALKBH3‐MT, respectively) (Figure 5m).

Using m1A‐RIP‐qPCR, we first confirmed site‐specific m1A loss at PINK1 mRNA in cells expressing dCas13Rx‐ALKBH3‐WT with *PINK1*‐targeting gRNAs (gRNA‐*PINK1*) (vs. non‐targeting gRNA control gRNA‐NT; Figure 5n). This experiment demonstrated successful programmable demethylation at the target transcript. Quantitative RT‐qPCR analysis revealed that this targeted m1A erasure reduced PINK1 mRNA levels by nearly 2‐fold in dCas13Rx‐ALKBH3‐WT cells compared to the catalytically inactive dCas13Rx‐ALKBH3‐MT (Figure 5o), demonstrating that ALKBH3's demethylase activity directly suppresses PINK1 transcript abundance. Critically, targeted PINK1 demethylation recapitulated the ALKBH3 overexpression phenotype, reducing LC3B‐II and increasing TOMM20 accumulation relative to gRNA‐NT controls (Figure 5p).

Taken together, these results establish that ALKBH3 control of mitophagy depends overwhelmingly on m1A‐mediated suppression of PINK1. Given that PINK1‐dependent mitophagy prevents accumulation of ROS‐generating dysfunctional mitochondria [87, 88], this mechanism is critically positioned to influence brain health. The pathological relevance of this mechanism is further underscored by our analysis of human AD single‐cell RNA‐seq data [30], which revealed an inverse relationship between ALKBH3 and PINK1 expression. AD samples exhibited significantly elevated ALKBH3 and concomitantly reduced PINK1 levels compared to controls overall (Wilcoxon rank‐sum test, p = 4.9 × 10^−5^ and p = 1 × 10^−7^, respectively; Figures 1g, 5q). Direct correlation analysis demonstrated a strong negative association between ALKBH3 and PINK1 expression across individual cells in both AD (Spearman ρ = −0.739, p < 2.2 × 10^−^^1^ ^6^) and control (Spearman ρ = −0.713, p < 2.2 × 10^−^^1^ ^6^) cohorts (Figure S11c,d). This conservation across species, combined with our mechanistic dissection, positions the ALKBH3‐m1A‐PINK1 mechanism (Figure 5r) as a central pathway connecting epitranscriptomic dysregulation to mitochondrial pathology with relevance to human neurodegeneration.

## 3 Conclusion

Our study reveals that ALKBH3‐driven m1A demethylation is a mechanism that orchestrates progression of AD through coordinated disruption of mitochondrial and synaptic homeostasis. By erasing m1A modifications from PINK1 mRNA, ALKBH3 suppresses mitophagy, leading to accumulation of dysfunctional mitochondria and subsequent oxidative stress (Figure 5r). This phenotype can be rescued by both genetic (*Alkbh3* ablation in 5xFAD mice) and pharmacological (HUHS015 treatment) approaches. Crucially, this pathway is clinically relevant: we observe *PINK1* reduction in human AD brain, highlighting its important role in AD pathogenesis beyond its well‐established function in Parkinson's disease [20]. Our 5xFAD models demonstrate that reducing Alkbh3 levels not only restores PINK1 expression and mitophagy but also reduces Aβ plaques and improves cognitive function. Mechanistically, we find that ALKBH3 upregulation elevates BACE1 to promote Aβ production and concurrently impairs microglial phagocytic function, linking mitochondrial failure to both arms of Aβ dysregulation. It is important to note that although our data establish the ALKBH3‐PINK1‐mitophagy axis as a significant contributor to Aβ accumulation, AD pathogenesis is multifactorial and is likely to involve also ALKBH3‐independent pathways.

Nevertheless, our findings are significant and establish RNA methylation as a previously unrecognized regulatory layer connecting epitranscriptomic changes to canonical AD hallmarks. The observed calcium dysregulation and synaptic failure can be directly explained by PINK1's dual role: beyond initiating mitophagy, PINK1 has been shown to also phosphorylate the mitochondrial calcium transporter LETM1 to regulate mitochondrial calcium buffering [89]. Thus, ALKBH3‐mediated PINK1 suppression creates a dual‐hit—impairing both the clearance of damaged mitochondria and the organelle's ability to handle calcium—which converges to disrupt synaptic activity and cognitive function.

Our data position ALKBH3 as a mitochondrial‐associated RNA demethylase. We found ALKBH3 enriched at the mitochondrial outer membrane, where it interacts with TOMM20. Furthermore, PINK1 mRNA co‐localized with ALKBH3 at this site, and its compartment‐specific stabilization upon ALKBH3 inhibition indicates that the demethylation event likely occurs on the mitochondrial surface. This spatial regulation aligns with studies showing PINK1 mRNA is anchored to mitochondria for local translation during mitophagy [90, 91]. While ALKBH3 can also function as a DNA repair enzyme [53], m1dA was undetectable in our model, and ALKBH3 did not bind mtDNA, arguing that its RNA demethylase activity drives the observed phenotypes.

Our mechanistic focus on PINK1 is justified by a systematic, multi‐tiered prioritization. Our triple‐intersection analysis of ALKBH3 m1A targets, mitochondrial genes, and AD‐dysregulated synaptosomal transcripts initially identified 15 candidates. Among these, PINK1 stood out as the master regulator of the mitophagy pathway, a process with a well‐established and central role in AD pathogenesis. Orthogonal validation further prioritized PINK1: it was the sole candidate bearing a validated m1A site in an independent [57], high‐resolution mapping study [57], and it exhibited consistent and significant downregulation in human AD single‐cell transcriptomes. This specific focus on a single, validated target does not imply ALKBH3 is exclusively mitochondrial or that PINK1 is its sole substrate; indeed, our m1A‐ID‐seq data reveal numerous mitochondrial‐related transcripts as potential ALKBH3 targets. While other candidates such as the mitophagy receptor BNIP3 [47], TOMM20 [92], and the metabolic regulators FBXO7 [93] and HAGH [94] are of biological interest, their direct links to AD epitranscriptomics and their functional validation in this context remain to be explored. The functional rescue experiments demonstrating that PINK1 overexpression alone reverses ALKBH3‐induced mitophagy blockade solidify its key causal role. Therefore, although PINK1 regulation is sufficient to explain the core mitophagy deficit, the m1A‐ID‐seq data suggest ALKBH3 likely coordinates a broader mitochondrial and synaptic RNA regulon. Future studies investigating targets such as BNIP3, TOMM20, and others may reveal additional layers of epitranscriptomic control contributing to the multifaceted mitochondrial and synaptic dysfunction in AD.

Overall, the ALKBH3‐m1A‐PINK1 axis could represent both a diagnostic biomarker for early mitochondrial dysfunction and a promising therapeutic target, but require further validations. Our findings using the inhibitor HUHS015 provide a proof‐of‐concept that pharmacological inhibition of ALKBH3 can mitigate amyloidogenic pathology in our models. However, further studies are essential to evaluate its off‐target effects, pharmacokinetics, and in vivo efficacy before any therapeutic potential can be ascertained. Our preliminary data suggesting ALKBH3 also influences Tau phosphorylation positions it as a potential modulator of the broader AD pathogenic cascade [81], though this also requires dedicated future studies. The conservation of this pathway across neurodegenerative contexts, notably the central role of PINK1 in both AD and Parkinson's [20, 95, 96], suggests that targeting ALKBH3‐mediated epitranscriptomic control could also have broad therapeutic potential. Our CRISPR‐demethylation system and small‐molecule inhibitor provide preclinical proof‐of‐concept for such intervention strategies in AD and pave the way for exploration in other proteinopathies characterized by mitochondrial failure.

## 4 Experimental Section

### 4.1 Cell Lines

HEK293T cells (RRID:CVCL_0063) were purchased from Beijing Xiehe Cell Bank (1101HUM‐PUMC000091) and cultured at 37°C with 5% CO_2_ in DMEM (#11965092, Gibco, USA) containing 10% FBS (# SE100‐B, VisTech, China). Cells were passaged for less than 20 times and have been regularly tested for mycoplasma. The human neuroblastoma cell line SH‐SY5Y (RRID:CVCL_0019) was purchased from Beijing Xiehe Cell Bank (1101HUM‐PUMC000026) and cultured at 37°C with 5% CO_2_ in DMEM/F12 (#11320033, Gibco, USA) containing 10% FBS. Cells were passaged for less than 20 times and have been regularly tested for mycoplasma. BV‐2 cells (RRID: CVCL_0182) were obtained from Vector Core at CIBR and cultured at 37°C with 5% CO2 in DMEM containing 10% FBS. Cells were passaged less than 20 times and have been regularly tested for mycoplasma. All cell lines used were confirmed to be free of contamination. HEK293T cells were chosen for their high transfection efficiency, which was essential for mechanistic studies involving general protein and RNA interactome analysis, m1A mapping, and mitophagy pathway dissection. SH‐SY5Y cells were employed as a neuronal model to investigate ALKBH3's effects on calcium signaling, synaptic protein expression, and amyloidogenic processing (BACE1/Aβ). The murine BV‐2 microglial cell line was chosen to specifically investigate the phagocytic clearance of Aβ, a key function of microglia in AD pathogenesis.

### 4.2 Plasmids

The APP plasmid (#MH00237) was purchased from Mailgene Biotechnology (Beijing, China). The GFP‐LC3B (#D2815) and mCherry‐GFP‐LC3B (#D2816) plasmids were obtained from Beyotime (Shanghai, China). The pPINK1 (HIS‐PINK1; #HH20240131GX‐PC02) plasmid used was from Hanbio Biotechnology (Shanghai, China). The Tau‐P301L (pRK5‐EGFP‐Tau P301L, #46908) plasmid was obtained from Addgene (USA). The WT FLAG‐ALKBH3 plasmid (pALKBH3) was amplified and cloned via PCR from HEK293T cell cDNA and subsequently inserted into the p3 × FLAG CMV10 Vector (pVector) (#VT1070, Youbio, China). The full‐length sequence of *ALKBH3* was confirmed by Sanger sequencing before proceeding with further experiments. The catalytically inactive mutant FLAG‐ALKBH3‐D193A (pALKBH3‐MT) was generated from the WT ALKBH3 (pALKBH3‐WT) plasmid using site‐directed mutagenesis according to the protocol (Table S3) [97]. The mutant sequence was verified by Sanger sequencing prior to subsequent experiments.

dCas13Rx‐ALKBH3‐MT and dCas13Rx‐ALKBH3‐WT, which fused the catalytically active (pALKBH3‐WT) and inactive (pALKBH3‐MT) domains to dCas13Rx, were obtained from Yang Li at Zhejiang University [98]. We designed guide RNAs (gRNAs) targeting the PINK1 transcript using cas13design (https://cas13design.nygenome.org), and gRNA‐*PINK1* was subsequently cloned into the gRNA backbone. The designed gRNAs were subject to MEGABLAST (https://blast.ncbi.nlm.nih.gov/Blast.cgi) to avoid mismatching to unexpected mRNA in human genome. The sequences of gRNAs are listed in Table S3 gRNA‐NT are negative controls that do not have a target. Cells were co‐transfected with dCas13Rx‐ALKBH3‐MT or dCas13Rx‐ALKBH3‐WT and gRNA at a mass ratio of 2:1 using Lipofectamine 3000 (#L300015, Thermo Fisher, USA) according to the manufacturer's instructions.

All to be transfected cell lines were grown and passaged in dishes or six‐well plates at approximately 80% confluency before transfection. All plasmids were transfected to cell lines with Lipofectamine 3000 (#L300015, Thermo Fisher, USA) according to the manufacturer's instructions.

### 4.3 Viral Plasmid Generation and Delivery

Lentiviral short‐hairpin RNA (shRNA) constructs for human *ALKBH3* were obtained according to the pLKO.1‐puro (#8453, Addgene, USA) vector protocol. Oligonucleotides used for the knockdown of human *ALKBH3* were as follows: shALKBH3: CGCACATTTGAGATGAGAAAG. Lentiviral *ALKBH3* expression plasmids were constructed by cloning the full‐length open reading frames (ORFs) of the human *ALKBH3* (NM_139178.4), following the pLKO.1‐puro vector protocol.

Lentiviral particles were produced in HEK293T cells using the second‐generation packaging system. Cells were seeded at 1 × 10^7^ cells/T175 flask and transfected at ∼80% confluency (typically 24 h post‐seeding) with a plasmid mixture containing 15 µg transfer vector, 30 µg psPAX2 (#12260, Addgene, USA), and 30 µg pMD2.G (#12259, Addgene, USA) using Neofect transfection reagent (1:1 DNA: reagent ratio) in Opti‐MEM (#31985070, Thermo Fisher, USA). After 6–8 h, the medium was replaced with serum‐free DMEM supplemented with NEAA/Glutamine. Viral supernatants were harvested 48–72 h post‐transfection, clarified by centrifugation (2500× *g*, 5 min), and concentrated using a commercial kit (#41101ES50, YEASEN, China) to require higher titer. 2 µl of the virus (7 × 10^11^ gc/mL) was added to the 35mm‐dish containing the HEK293T or SH‐SY5Y cells and transfection efficiency was tested at 48 h.

Adeno‐associated plasmid pAAV‐CAG‐*Alkbh3*‐P2A‐WPRE‐SV40 polyA (AAV‐Alkbh3) was constructed by cloning the full‐length ORF of the mouse *Alkbh3* gene (NM_001418679.1). Its control plasmid, pAAV‐CAG‐P2A‐WPRE‐SV40 polyA (AAV‐Vector), was provided by the Vector Core at CIBR. Additionally, the adeno‐associated short‐hairpin RNA (shRNA) pAAV‐U6‐shAlkbh3 (shAlkbh3), targeting *Alkbh3* with the sequence GAAGGAAGCTGACTGGATCTT, along with its control shRNA (shControl), were also provided by the Vector Core at CIBR. To prepare adeno‐associated viruses (AAV) from these constructs, each plasmid was co‐transfected into HEK293T cells with a rep/cap containing plasmid pUCmini‐iCAP‐PHP.eB (#103005, Addgene, USA) and the helper plasmid pAdDeltaF6 (#112867, Addgene, USA), in the presence of polyethylenimine [99]. After packaging, AAV‐shAlkbh3 and AAV‐shControl were purified and subsequently administered to mice via injection; in parallel, cells were transduced with the respective viruses.

Prior to infections, mice were anesthetized with 0.35 g/kg of 2.5% avertin through intraperitoneal injections. AAV stereotaxic injections were performed targeting the hippocampus of adult mice, with X/P 1.94 mm, M/L 1.5 mm from the bregma point, and 2 mm depth for mice. The rate of virus injection was 1 nL/s. The injection interval at each position was 8 min. Hippocampus regions were extracted 2 weeks after viral injections for downstream processing. For the infection of primary neurons, 1 µL of the virus (5 × 10^12^ gc/mL) was added to a 35‐mm dish containing the primary neurons for at least 72 h.

### 4.4 Mice Construction and Genotyping

*Alkbh3* floxed mice were generated by CRISPR/Cas9‐mediated genome editing in C57BL/6J zygotes. Two loxP sites were inserted into the intronic regions flanking exon 3 of the *Alkbh3* gene (chromosome 2, Gene ID: 69113). The presence of loxP sequences enabled conditional deletion of exon 3 upon breeding with CMV‐Cre transgenic mice (B6.C‐Tg (CMV‐Cre)1Cgn/J, JAX stock no. 006054), in which Cre recombinase expression drives recombination between loxP sites. Excision of the 35‐bp exon 3 introduces a frameshift mutation that results in complete loss of *Alkbh3* function, thereby generating *Alkbh3* knockout mice.

To investigate the effects of *Alkbh3* deficiency in the 5xFAD background, *Alkbh3* knockout mice were crossed with 5xFAD transgenic mice (B6SJL‐Tg (APPSwFlLon, PSEN1*M146L*L286V) 6799Vas/Mmjax, JAX stock no. 006554). While homozygous mutants for either 5xFAD or *Alkbh3* knockout can be obtained when bred separately, in the double‐mutant breeding scheme, offspring carrying homozygosity at either the 5xFAD or *Alkbh3* locus were not obtained. Therefore, all experiments involving the double‐mutant background were performed using mice heterozygous for both the 5xFAD and *Alkbh3* alleles (5xFAD^+/−^ *Alkbh3* ^+/−^). Genotyping of 5xFAD mice was performed by PCR using primer sets recommended by The Jackson Laboratory (stock no. 006554; Table S3). Representative results of 5xFAD^+/−^ *Alkbh3* ^±^ mice are shown in Figure S2c,d.

C57BL/6J WT mice were provided by the Laboratory Animal Resource Center (LARC) at CIBR and were originally purchased from the Jackson Laboratory. 6‐ to 8‐month‐old male mice were used for virus injection experiments. Animals were housed in a 12‐hour light/12‐hour cycle, at constant temperature and under enrichment environmental conditions with ad libitum access to sterile food and water. All procedures were performed by trained personnel, in accordance with the AVMA Guidelines for the Euthanasia of Animals: 2020 Edition. All animal procedures were approved by the Institutional Animal Care and Use Committee (IACUC) at CIBR (IACUC approval number: CIBR‐IACUC‐026), and complied with CIBR's institutional ethical standards and the ARRIVE 2.0 guidelines.

Genomic DNA from mouse brains was PCR amplified using the 2 × Taq PCR Mix (#310512, BGI, China). PCR primers are listed in Table S3. Controls were always processed in parallel. Gel electrophoreses were carried in 0.5 × Tris‐acetate‐EDTA buffer. Gels were imaged with the Gel Doc System (Bio‐Rad, USA). DNA fragments molecular weights were compared to the DNA Marker II (#MD102‐02, Tiangen, China).

### 4.5 DNA and RNA Extraction

For the extraction of DNA and RNA from the hippocampi, the tissues were initially homogenized using a glass tissue grinder. Genomic DNA from both tissue and cells was extracted using the DNeasy Blood & Tissue Kit (#69504, Qiagen, Germany) according to the manufacturer's instructions. Total RNA was extracted using 1000 µL of TRIzol reagent (#15596026, Invitrogen, USA) for each brain sample and for cells in a 6‐cm dish. After vortexing to facilitate disintegration, samples or cells were incubated for 5 min before 200 µL of chloroform was added. After mixing and further 5 min incubation, samples were centrifuged at 12 000× *g* for 15 min at 4°C. The upper aqueous phase containing total RNA was recovered and mixed with 0.1 × the volume of sodium acetate (3 M, pH 5.2), 1 × the volume of isopropanol, and 2 µL glycogen (#10901393001, Invitrogen, USA). After at least 50 min incubation at −80°C the RNA was precipitated through centrifuging at 15 000× *g*. After washing the pellet with 70% ethanol for three times and air drying, the RNA was resuspended in nuclease‐free water. DNA and RNA concentrations were measured through Qubit assays (#Q33225, Invitrogen, USA).

### 4.6 LC‐MS/MS

The abundance of m1A and A in RNA was performed on a TSQ Altis triple quadrupole mass spectrometer (Thermo Fisher, USA) coupled to a Vanquish Flex UHPLC system (Thermo Fisher, USA) fitted with an Acquity UPLC HSS T3 column (2.1 × 100 mm, 1.8 µm particle size, Waters). The mobile phase A was 0.5% formic acid in water and mobile phase B was 0.5% formic acid in acetonitrile. The flow rate was 300 µL/min. The mass spectrometer was set in a positive ion mode and operated in selective reaction monitoring. The precursor ions of adenosine (A, Sigma‐Aldrich, USA) (m/z 268.1) and N1‐methyl‐2'‐adenosine (m1A, Sellechchem, USA) (m/z 282.1) were fragmented and the product ions of A (m/z 136.1) and m1A (m/z 150.1) were monitored. 2’‐deoxyadenosine (15N5) (dA‐5) was used as internal standard at 10 nM. m/z 257.1 was precursor ion and m/z 141.2 was production. The peak area ratio of the m1A/dA‐5 and A/dA‐5 was extracted using the XCalibur Quan Browser software (Thermo Fisher, USA). Serial dilutions of synthetic standards for A and m1A were used to generate their calibration curves for quantification. The level of m1A present in the sample was calculated according to the following equation: (%) m1A = 100 × m1A/A.

### 4.7 Measurement of m1dA in DNA by LC‐MS/MS

For assessment of m1dA accumulation in total DNA, DNA was isolated using the Tissue and cell DNA extraction kit (#69504, QIAGEN, German), which included a step for RNase treatment to remove contaminating RNA. The concentration and purity of the isolated DNA were quantified using a NanoDrop 2000 spectrophotometer (Thermo Scientific, USA). Subsequently, 2 µg of purified DNA was enzymatically digested to single nucleotides and subjected to quantitative analysis by liquid chromatography‐tandem mass spectrometry (LC–MS/MS). The standard of m1dA and its retention time was 6.05 min.

### 4.8 RT‐qPCR

RNA was treated with DNase I (#RT411, Tiangen, China) and then reverse transcribed with HiScript III All‐in‐one RT SuperMix following the manufacturers manual (#R333‐01, Vazyme, China). RT‐qPCR was performed with the Abi QuantStudio 3 System (Thermo Fisher, USA), using the SYBR Green JumpStart Taq Ready Mix (#S4438, Sigma‐Aldrich, USA). In every run, negative controls (non‐template) and positive controls were included. RT‐qPCR results from samples were normalized to cDNA levels of the housekeeping gene *GAPDH* and *β‐Actin* to control samples. Gene‐specific primers used for RT‐qPCR are listed in Table S3.

### 4.9 Experimental Design and Statistical Comparisons

For all cellular and in vivo experiments involving modulation of ALKBH3 levels, rigorous internal controls were employed. Each experimental condition (e.g., AAV‐ALKBH3, shALKBH3, pharmacological inhibition) was compared directly and exclusively to its own appropriate control group (e.g., AAV‐Vector, shControl, vehicle‐treated) processed in parallel within the same experimental batch. It is important to note that gain‐of‐function (overexpression) and loss‐of‐function (knockdown) experiments were typically performed in separate experimental series. Therefore, while the AAV‐Vector and AAV‐shControl groups both serve as valid negative controls for their respective manipulations, they are not intended for direct statistical comparison with each other, as they may reflect baseline variations inherent to independent experimental runs. This design ensures that all observed phenotypic changes are attributable to the specific modulation of ALKBH3 activity relative to its matched control.

### 4.10 Immunofluorescence Staining, Visualization, and Analysis

For immunofluorescence staining, mice were perfused with ice‐cold phosphate buffered saline (PBS) and ice‐cold 4% paraformaldehyde (PFA). The whole brain was isolated and fixed overnight in 4% PFA, then incubated on a rolling mixer (Kylin‐Bell, China) for at least 24 h in 30% sucrose at 4°C. The brain was frozen at −80°C, and cryo‐sectioned into sagittal slices with 20 µm thickness. Brain slices were thaw‐mounted on a pre‐cooled, positively charged glass slide (Genview, China). Two consecutive brain slices were mounted next to each other. Slides were subsequently washed in PBS on a horizontal shaker at 4°C overnight. Each brain slice was circled with a liquid blocker PAP pen (#Z672548, Sigma‐Aldrich, USA) to prevent leaking during staining. Next, slices were permeabilized for 1 h with 0.3% Triton‐X100 (#T8787, Sigma‐Aldrich, USA). After three PBS washes, slices were blocked for 1 h in PBS containing 5% non‐fat powdered milk (#A600669, BBI, China) at room temperature. After rinsing with PBS containing 0.1% Tween‐20 (#P1379, Sigma‐Aldrich, USA), one brain slice was stained with a primary antibody. The following primary antibodies were used are listed in Table S3. The Staining was carried out in PBST with 5% bovine serum albumin (#B2064, Sigma‐Aldrich, USA) overnight at 4°C, followed by three PBS washes. Secondary antibodies were applied for 1 h at room temperature. The following secondary antibodies were used on all sections: Alexa‐Fluor 488 Goat Anti‐Rabbit (#ab150077, Abcam, USA, 1:100), Alexa‐Fluor 594 goat anti‐mouse (#ab150116, Abcam, USA, 1:100). Following three PBS washes, all brain slices were sealed with the Antifade Mounting Medium containing 1.5 µg/ml DAPI (#H‐1200, Vectashield, USA). Brain slices were imaged with the Olympus VS120 Virtual Slide Microscope, using the OlyVIA software (DAPI: Excitation 365/10 nm, Emission 440/40 nm; GFP: Excitation 472/30 nm, Emission 520/35 nm; CY5 Excitation 628/40 nm; Emission 692/40 nm).

For cell staining, cells grown on glass coverslips were fixed in 4% paraformaldehyde for 30 min, and then permeabilized in 0.2% Triton X‐100 for 15 min. The cells were then blocked in PBS containing 5% milk for 2 h, before incubating with the primary antibody at 4°C overnight. Following incubation with the secondary antibody in PBST at room temperature for 1 h, nuclei were counter‐stained with DAPI (#H‐1200, Vectorlabs, USA). The coverslips were then mounted onto slides. Images were visualized and collected using a confocal microscope TCS SP8 (Leica, Microsystems, Germany).

Immunofluorescence images were acquired using a confocal microscope, with exposure settings consistently maintained between each experimental group and its corresponding control group. Quantitative analysis of fluorescence intensity was performed using ImageJ software (v1.53; National Institutes of Health, USA). For each image, regions of interest (ROIs) corresponding to individual cells or specific cellular compartments were delineated based on DAPI staining or differential interference contrast (DIC) images. For quantitative analysis of TOMM20 and PINK1 in perinuclear region and the distal region, the cytoplasm was divided into two distinct regions: the perinuclear region, defined as the area within a ≤20 µm radius from the nuclear envelope, and the distal region, defined as the area beyond 20 µm from the nuclear envelope and extending to the plasma membrane.

The mean gray value within each ROI was measured after subtracting the background intensity, which was determined from a cell‐free area of the same image. Data from at least three independent experiments were pooled, and fluorescence intensity was expressed as mean ± SEM after normalization to the control group.

### 4.11 Human Single‐Nucleus RNA‐Seq Analysis

Publicly available human single‐nucleus RNA‐seq (snRNA‐seq) data were obtained from the Gene Expression Omnibus (GEO; GSE175814), comprising post‐mortem brain samples from two AD patients and two age‐matched control individuals. Samples were collected from multiple cortical regions, including the anterior hippocampal cortex, BA41/42, and BA6/8 [30]. Raw count matrices were imported into Seurat (v4.4) [100] using the ReadMtx function and converted into Seurat objects. Sample identities were extracted from cell barcode names and stored as metadata together with disease condition labels (AD or Control). To reduce noise from sparsely detected genes, gene‐level filtering was performed using the raw RNA count matrix. Genes expressed in fewer than 10 cells across the dataset were excluded from further analysis. Single‐nucleus quality control was subsequently applied by computing standard RNA complexity metrics and mitochondrial gene content. Cells were retained if they satisfied the following criteria: total RNA counts greater than 1000, detected gene numbers between 200 and 9000, and mitochondrial read fraction below 15%. Following quality control, disease condition labels were assigned as the active cell identities for downstream comparative analyses. Filtered datasets were log‐normalized and scaled after identification of highly variable features. Dimensionality reduction was performed using principal component analysis (PCA), followed by Uniform Manifold Approximation and Projection (UMAP) for visualization [101]. Transcriptionally distinct cell populations were identified using unsupervised clustering. Cell‐type annotation was performed using a marker gene–driven, cluster‐level approach. Cluster‐specific marker genes were identified using Seurat FindAllMarkers function, restricting the analysis to positively enriched genes (only.pos = TRUE) with a minimum detection rate of 25% of cells per cluster and a minimum log_2_ fold‐change threshold of 0.25. Marker genes were further filtered based on an adjusted *p*‐value <0.05 and average log_2_ fold‐change >0.25, and the top‐ranked markers per cluster were retained. Cell‐type identities were then assigned by mapping cluster labels to predefined cell‐type categories based on established marker gene enrichment patterns encoded directly in the analysis pipeline. These categories included excitatory neuron (ExN) subtypes, inhibitory neuron subtypes (InN), astrocytes, oligodendrocytes, oligodendrocyte precursor cells (OPCs), microglial subpopulations, macrophages, endothelial cells, vascular‐associated cells, and low‐quality or doublet clusters. Annotation was applied programmatically using Seurat RenameIdents function, and annotated cell types were stored as metadata for downstream analyses. Expression levels of ALKBH3 and PINK1 were quantified as log‐normalized counts per cell. Group‐wise comparisons between AD and Control samples were performed using the Wilcoxon rank‐sum test and visualized using violin plots, box plots, and UMAP feature maps. Cell‐type–resolved analyses were conducted to assess expression differences across neuronal and glial populations. To evaluate the relationship between ALKBH3 and PINK1 expression, Spearman's rank correlation analysis was performed separately within AD and Control samples using all cells within each condition. Correlation coefficients and corresponding *p*‐values were calculated and visualized using scatter plots with LOESS‐smoothed trends and 95% confidence intervals. All statistical analyses and visualizations were performed in R (v4.5).

### 4.12 Fluo‐4AM Assay

Calcium imaging in SH‐SY5Y cells was performed using Fluo‐4AM (#HY‐101896, MCE, USA). Prior to experiments, a 1 mM stock solution of Fluo‐4AM was prepared in anhydrous DMSO, aliquoted, and stored at ‐20°C protected from light. For cell loading, the stock solution was diluted to a final concentration of 2 µM in Hanks' Balanced Salt Solution (HBSS) containing 20 mM HEPES (pH 7.4) and 0.02% Pluronic F‐127 (#P3000MP, Thermo Fisher, USA) to facilitate dye uptake. SH‐SY5Y cells were cultured on dishes until reaching 70%–80% confluency, then washed twice with HBSS before incubation with the Fluo‐4AM working solution for 30 min at 37°C in the dark. Following dye loading, cells were washed twice with HBSS. Fluorescence‐activated cell sorting (FACS) was performed using a high‐performance cell sorter BD FACS Aria III (BD Biosciences, USA) equipped with lasers.

### 4.13 Fluorescence‐Activated Cell Sorting (FACS) Analysis

Single‐cell suspensions were prepared and stained with fluorochrome‐conjugated markers as indicated in figures and legends. Cells were resuspended in PBS and filtered through a 70 µm strainer to minimize aggregates. Data acquisition was performed on a BD FACS Aria III system. Voltages were established using unstained controls (Figure S5e). Analysis was conducted with FlowJo (v10.8.1). A sequential gating strategy was implemented: debris exclusion via FSC‐A versus SSC‐A, doublet discrimination via FSC‐A versus FSC‐H, and viability gating using Fixable Viability Dye eFluor 506 (eBioscience). Specific cell populations were identified using fluorescence markers as detailed in corresponding figures. Statistical analysis was performed on ≥10 000 events per sample.

### 4.14 Barnes Maze Trial and Configuration

During the habituation trial, mice were released from the center of the maze, with the escape tunnel positioned at Location 1. In the subsequent acquisition phase (Trials 1–5), the starting position varied pseudo‐randomly across the centers of Quadrants 4, 2, 3, 1, and 4, respectively, while the escape tunnel remained fixed at Location 2 throughout. For the probe trial, the mouse was again placed at the center of the maze, and the escape tunnel was removed to assess spatial memory retention.

Mice were habituated to the maze (Day 1) by free exploration for 5 min with an escape tunnel present. During acquisition training (Days 2–3), mice completed 3 trials (Day 2) and 2 trials (Day 3) with 3 min limits and 1 h intervals, motivated by bright light/white noise (90 dB); the maze was ethanol‐cleaned between trials. On Day 5, memory was assessed in a 1 min probe trial (tunnel removed) while recording hippocampal calcium activity via fiber photometry. Escape latency and quadrant preference were analyzed using tracking software. During the probe trial, the following parameters were recorded to evaluate spatial learning and memory performance: latency and path length to reach the former location of the escape tunnel, proximity to the target hole, total distance traveled, number of visits to the target hole, and number of visits to incorrect holes.

### 4.15 Stereotaxic Viral Injection of GCaMP6m and Optical Fiber Implantation

After deep anesthesia with isoflurane in oxygen, the mice were placed on the stereoscopic positioning instrument (Stoelting Co., USA). Anesthesia remained constant at 1% to 1.5% isoflurane supplied per anesthesia nosepiece. The eyes were coated with erythromycin eye ointment. The scalp was cut open, and the fascia over the skull was removed with 3% hydrogen peroxide in saline. The bregma and lambda points were used to level the mouse head. A small window of 200 to 300 µm in diameter was drilled just above the hippocampus for viral injection and optical fiber implantation (AP = ‐2.0 mm, ML = ‐2.04 mm, DV = ‐1.27 mm, angle = 10 degree). AAV2/9‐hysn‐GCaMP6m_pA178 (AAV‐GCaMP6m) was provided by the Vector Core at CIBR. A total of 300 nL AAV‐GCaMP6m solution (1.0 × 10^13^gc/mL) was slowly injected at 30 nL/min bilaterally for fiber photometry recording with MO10 (Narishige, Japan) coupled with a glass electrode. After injection, the glass electrode was kept in place for 10 min and then slowly withdrawn. An optical fiber (200 µm, 0.37 numerical aperture, Newdoon, China) hold in a ceramic ferrule was slowly inserted into the brain tissue with the tip slightly 100 µm above the viral injection sites. The fiber was sealed to the skull with dental cement. Postoperative care consisted of maintaining normothermia during anesthetic recovery and subcutaneous enrofloxacin prophylaxis (Baytril; 10 mg/kg, twice daily) for 72 h. Following a 14‐day recovery period with daily veterinary monitoring, animals were subjected to the Barnes maze test.

### 4.16 Fiber Photometry Recording and Data Analysis

Fiber photometry recording: Neuronal activities of hippocampus during the Barnes maze test were monitored using the FPS‐405/470/578 photometry system (Developed by CIBR Imaging Core) with the Tri‐photometry software (Developed by CIBR Imaging Core). We used a dual‐wavelength approach: 470 nm LED was used to elicit calcium signal‐dependent fluorescence, while the 405 nm LED reference channel served for motion correction. Light intensity at the fiber tip was calibrated to 10–20 µW. Alternating excitation wavelengths were delivered, and the fluorescence signals were collected using a CMOS camera during dual‐color imaging with 10‐FPS for each channel. We used OBS Studio (https://github.com/obsproject/obs‐studio) to synchronize the behavior video and calcium signal for calcium data analysis.

### 4.17 Calcium Data Analysis

Raw fluorescence signals were exported and analyzed in MATLAB (R2023b, MathWorks, USA) via custom‐written scripts. Photo‐bleaching was first corrected for each channel. The channel 405 signal was used for motion correction of the calcium signal channel. Target hole exploration (E_c_, correct exploration) was scored when the mouse's snout fully entered the target hole while the remainder of its body remained outside the hole. For each correct exploration event (Ec), we extracted fluorescence signals (F) from a 4‐second window centered on exploration onset (2s before to 2s after). These raw fluorescence values were then transformed into ΔF/F and z scores based on the following formulas:

$$\Delta F/F=(F-\mathrm{mean}(F_{\mathrm{pre}})/\mathrm{mean}(F_{\mathrm{pre}})$$

$$\;F_{\mathrm{zscore}}=(F-\mathrm{mean}(F_{\mathrm{pre}})/s.d.(F_{\mathrm{pre}})$$

where F_pre_ was defined as the fluorescence signal 2s before E_c,_ representing the baseline fluorescence. The calcium signal change was calculated by subtracting the peak value of the F_z score_ signal. Neural activity traces were visualized as mean ΔF/F or z‐score trajectories, with shaded regions representing the standard error of the mean (SEM) across all exploration events.

### 4.18 Primary Neuron Culture

To validate key cellular mechanisms in a physiologically relevant, post‐mitotic, neuronal context, we utilized primary hippocampal neurons. This model is essential for studying neuron‐specific processes such as mitochondrial axonal trafficking, dendritic complexity, synaptic protein localization, and the response of neuron activation, which may not be fully recapitulated in immortalized cell lines. Hippocampal neurons from mouse embryos were used in compliance and approval with the Laboratory Animal Welfare and Ethics guidelines at CIBR. The hippocampi of E17 mouse embryos were quickly dissected on ice in a dissection medium after the embryos were sacrificed. The dissection medium consisted of 97.5% HBSS (Ca^2+^ and Mg^2+^ free) (#14175095, Gibco, USA), 11 mg/ml sodium pyruvate (#S104174, Aladdin, China), 0.1% glucose (#520I021, Solarbio, China), and 10 mM HEPES (pH 7.3) (#15630080, Gibco, USA). Cell culture plates were coated with Poly‐L‐ornithine (#P4957, Sigma‐Aldrich, USA) and DMEM/F12 (#11320033, Gibco, USA) for at least 4 h at 37°C before seeding. The dissected tissues were cut into small pieces and digested with 0.125% Trypsin (#2887837, Gibco, USA) at 37°C for 10 min. After terminating the digestion and resuspending the cells, the filtered cell suspension was seeded in 6‐well plates at a density of 7 × 10^5^ cells/well in Neurobasal Medium (#21103049, Gibco, USA) supplemented with 2% B27 (#21103049, Gibco, USA), 1% Glutamate (#35050061, MaxGibco, USA), and 1% Penicillin‐Streptomycin solution (#P7539, Sigma‐Aldrich, USA). The cells were then maintained at 37°C in a humidified atmosphere with 5% CO_2_.

### 4.19 Protein Precipitation and Mass Spectrometry Analysis

HEK293T cells were transfected with the pALKBH3 plasmid that contains a FLAG‐tag. After 48 h the cells were then solubilized in lysis buffer (150 mM NaCl, pH 7.5, 50 mM Tris‐HCl, 5 mM EGTA, 5 mM EDTA, 1% NP‐40 and a protease inhibitor cocktail). The cell lysates were centrifuged at 13500× *g* at 4°C and subsequently incubated with anti‐Flag‐M2 magnetic beads (#M8823, Sigma‐Aldrich, USA) at 4°C for 4 h. After three washes, the beads were boiled at 95°C in 2 × SDS sample buffer, followed by Coomassie Brilliant Blue staining and LC‐MS/MS analysis using a MClass UPLC (Waters, USA) coupled with an Exploris 480 mass spectrometer (Thermo Fisher, USA). Peptide samples were loaded onto an Acclaim PepMap trap column (75 µm × 2 cm, 3 µm, C18, 100 Å, Thermo Scientific) and separated on a custom‐made analytical column (inner diameter, 100 µm) packed with 20 cm of C18 stationary phase (Aqua C18, 1.8 µm, 125 Å, Phenomenex). The eluted proteins were analyzed using gene ontology.

### 4.20 Intracellular Reactive Oxygen Species (ROS) Assay

To assess mitochondrial superoxide production, 1–2 mL of MitoSOX Green reagent (#M36005, Invitrogen, USA) was applied to a working solution at a concentration of 500 nM, ensuring that the reagent adequately covered cells adhering to coverslips in a 35‐mm dish. SH‐SY5Y cells were incubated for 30 min at 37°C in a 5% CO_2_ atmosphere. Following incubation, the cells were gently washed three times with warm Hank's Balanced Salt Solution (HBSS) containing calcium and magnesium, or a suitable buffer. Subsequently, fluorescence‐activated cell sorting (FACS) was performed, and the cells were analyzed based on their absorbance and emission spectra, which were optimally detected at 488 and 510 nm, respectively.

### 4.21 Measurements of Mitochondrial Membrane Potential

For the experiment, 1 mL of cell culture medium was added to each well of a 6‐well plate containing cells. For the positive control, CCCP (50 mM) (#HY‐K0601, MCE, USA) was brought to room temperature, and 1 µL was added to the corresponding wells to achieve a final concentration of 50 µM, followed by a 5 min incubation at 37°C. Subsequently, JC‐1 (200 µM) was also brought to room temperature, and 10 µL was added to each well to reach a final concentration of 2 µM, with a 15–20 min incubation at 37°C. After incubation, the supernatant was aspirated, and the cells were washed twice with PBS. Finally, 500 µL of PBS was added for observation using a fluorescence microscope or laser scanning confocal microscope, with green fluorescence detected at Ex/Em = 510/527 nm and red fluorescence at Ex/Em = 585/590 nm. Flow cytometry analysis was subsequently performed on the stained cells.

### 4.22 Subcellular Fractionation and Protease Protection Assay

Nuclear and cytoplasmic fractions were separated using the Nuclear and Cytoplasmic Protein Extraction Kit (#P0027, Beyotime, China) according to the manufacturer's instructions. Nuclear and mitochondrial fractions were isolated using the Mitochondria Isolation Kit for Cultured Cells (#89874, Thermo Fisher, USA) according to the manufacturer's instructions, using option B Dounce homogenization, with 60 strokes.

Protease protection assays were performed to determine the submitochondrial localization of ALKBH3. Equal amounts of purified mitochondria were divided into three groups, each in a total volume of 15 µl. For the intact mitochondria group, mitochondria were resuspended in 100 µl DPBS (#CC010, Macgene, USA). For complete membrane permeabilization, mitochondria were treated with 100 µL 0.1% Triton X‐100 prepared in the same DPBS buffer. For selective outer mitochondrial membrane permeabilization, mitochondria were treated with 100 µL 0.1% digitonin (#HY‐N4000, MCE, USA) prepared in the same DPBS buffer. All samples were incubated on ice for 10 min. Proteinase K was then added to each sample at a final amount of 1 µL (20 mg/mL; # GE201‐01, TransGen Biotech, China), followed by incubation on ice for 30 min. Protease digestion was terminated by the addition of 1 µL of 100 mM PMSF and a protease inhibitor cocktail prepared from cOmplete Tablets, EDTA‐free, EASYpack (#53002800, Roche; USA). Mitochondria were subsequently lysed, and protein samples were analyzed by WB.

### 4.23 Measurement of Aβ by ELISA

The concentration of Aβ42 in cell culture supernatants was quantified using a commercial *sandwich* ELISA kit (RK05017, Abclonal, China) following the manufacturer's instructions. Briefly, after cells were transfected with the APP plasmid (#MH00237, Mailgene Biotechnology, China) for 48 h, the culture medium was collected and centrifuged to remove cellular debris. Standards and supernatants were added to the antibody‐coated wells and incubated at 37°C for 2 h. Following washing, a biotinylated detection antibody was added and incubated at 37°C for 1 h. After another wash step, streptavidin‐HRP was added and incubated at 37°C for 30 min. Following a final wash, the color reaction was developed by adding TMB substrate and incubating in the dark at room temperature for 15 min, then stopped with the stop solution. Absorbance was immediately measured at 450 nm using a SpectraMax M5 microplate reader (Thermo Fisher, USA). The concentration of Aβ42 in each sample was determined by interpolation from a standard curve generated using a linear logistic fit. All samples were assayed in triplicate.

### 4.24 Aβ Uptake Measurement

Beta‐Amyloid (1–42) HiLyte Fluor 488‐labeled (#AS‐60479‐01, AnaSpec, USA) was used. Aβ42 oligomers were prepared by dissolving the peptide in DMSO, diluting in serum‐free medium to 10 µM, and aggregating overnight at 37°C with agitation. BV‐2 microglial cells were treated with these oligomers at a final concentration of 0.5 µM for 3 h at 37°C. Before measurement, medium was removed and extracellular Aβ42 was quenched with 100 µL 0.2% trypan blue in PBS for 1 min. After aspiration, Oligomer uptake was first visualized by fluorescence microscopy using 488 nm excitation. Cells were then detached, and the percentage of 488 nm‐positive cells was quantified by flow cytometry, with analyses performed in three independent biological replicates.

### 4.25 m1A RNA Immunoprecipitation and Gene‐Specific RT‐qPCR

Total RNAs were extracted from HEK293T cells with TRIzol reagent followed by an additional DNase I (#EN0541, Fermentas, USA) treatment to avoid genomic DNA contamination. For RNA fragmentation, samples were incubated for 4 min at 94°C in a fragmentation buffer (containing 10 mM ZnCl_2_, 10 mM Tris‐HCl, pH 7), followed by standard isopropanol precipitation. 100 µg of total RNAs were then incubated with 1 µg anti‐m1A antibody in RIP buffer (150 mM NaCl, 0.1% NP‐40, 10 mM Tris, pH 7.4), supplemented with RNase inhibitors at 4°C for 2 h. After incubation, 40 µl of Protein A/G beads rinsed with RIP buffer were added to the mixture of RNA and antibody and incubated for an additional 2 h at 4°C. Beads were washed five times with RIP buffer and precipitated RNAs were further purified with TRIzol according to the manufacturer's instructions (Invitrogen, USA). Input and immunoprecipitated RNAs were reverse transcribed into cDNAs (HiScript III RT SuperMix for qPCR (+ gDNA wiper)) and quantified by qPCR. Primers are listed in Table S3.

### 4.26 mRNA Decay Assay

Global mRNA transcription was inhibited by treating HEK293T cells with 5 µg/ml actinomycin D (HY‐17559, MCE, USA). Cells were harvested at the indicated time points, and total RNA was extracted for reverse transcription. The decay kinetics of PINK1 mRNA following transcription inhibition were assessed by quantitative RT‐qPCR.

### 4.27 4sU Assay

SH‐SY5Y cells were cultured to ∼70% confluency and treated with 500 µM 4‐thiouridine (4sU) (#13957‐31‐8, MCE, USA) for 1 h to label newly transcribed RNA. After labeling, cells were immediately washed twice with ice‐cold PBS to remove residual 4sU. Total RNA was extracted using TRIzol Reagent following the manufacturer's protocol. 4sU‐labeled RNA was biotinylated by incubating 50–100 µg of total RNA with 0.2 mg/mL Biotin‐HPDP (#HY‐136769, MCE, USA) in biotinylation buffer (100 mM Tris‐HCl, pH 7.4, 10 mM EDTA, 1% SDS) for 2 h at 25°C with gentle rotation. Biotinylated RNA was incubated with Dynabeads MyOne Streptavidin C1 (#65002, Thermo Fisher, USA) for 1 h at 4°C with rotation. Beads were washed and RNA was eluted by TRIzol.

### 4.28 Analysis of ALKBH3 and PINK1 in Human snRNA‐Seq Dataset

Publicly accessible single‐nucleus RNA‐seq datasets from the NCBl Gene Expression Omnibus (GEO) under the accession number GSE175814 (*n* = 2 AD, *n* = 2 controls) were used for our analyses [30]. The samples were collected from the anterior hippocampal cortex, BA41/42, and BA6/8 [30]. Raw count matrices were imported into Seurat (version 5.3.0) using ReadMtx and transformed into Seurat objects [100], with metadata indicating sample identity and disease status (AD or Control). The samples were merged, underwent log‐normalization, and were scaled after identifying highly variable features. Dimensionality reduction was achieved through principal component analysis (PCA) followed by UMAP embedding. The expression levels of ALKBH3 and PINK1 were measured as normalized counts per cell, with the mean expression and the fraction of expressing cells (counts > 0) computed for each condition. Comparison between the AD and control groups was executed using Wilcoxon rank‐sum tests. Visualization techniques involved violin plots, UMAP feature maps, and box plots of the expressing cell fractions to emphasize differences between groups. All analyses were conducted in R (version 4.5.0).

### 4.29 Identification of m1A Sites in Transcriptome Data

The raw data of m1A‐ID‐seq were obtained from NCBI Gene Expression Omnibus (accession number GSE73941). Reads were trimmed by Trim_galore (version 0.6.7; http://www.bioinformatics.babraham.ac.uk/projects/trim_galore/) and aligned to the human genome (hg19) with the HISAT2 program (version 2.2.1; http://daehwankimlab.github.io/hisat2/). SAM/BAM files were processed using SAMtools (version 1.15.1; http://samtools.sourceforge.net/). Aligned reads from both WT and *ALKBH3* knockout samples were analyzed following the strategy described by Li et al. [27]. Peak calling was performed using MACS2 [102], with ‘(‐) demethylase’ samples compared against corresponding input controls. The effective genome size parameter was set to the calculated transcriptome size (5 × 10^8^). To identify high‐confidence m1A peaks, two criteria were applied: (1) peaks must be present in both biological replicates; and (2) in either replicate, peaks must satisfy q < 1 × 10^−10^ and enrichment fold > 3. Only peaks meeting both requirements were retained for downstream analysis. The intersection of peak sets between replicates was determined using BEDTools [103].

### 4.30 Differential Expression and Volcano Plot

To investigate transcriptional changes associated with AD, we retrieved significantly deregulated mRNAs from Supplementary Table S8 of Zhang et al. [55]. This dataset includes differentially expressed synaptosomal mRNAs identified between AD and control samples, where controls represent synaptosomal mRNAs derived from 14 non‐AD post‐mortem brains. A total of 662 mRNAs were reported as significantly deregulated in AD versus control samples, highlighting synaptic transcriptional dysregulation associated with AD pathology [55]. The log_2_ fold‐change and corresponding adjusted p‐values were extracted and used to construct volcano plots (ggplot2 package (v 3.5.2)) [104], which visually depict the distribution of significantly up‐ and down‐regulated genes (|log2FC| > 0.5, *p*‐value < 0.01).

### 4.31 Gene Ontology (GO) Enrichment Analysis

To investigate the functional annotation of genes, GO enrichment analysis was performed using the clusterProfiler R package (version 4.4.4). The DEGs were first identified through differential expression analysis, and a list of gene symbols or Entrez gene IDs was generated for input into the GO enrichment analysis [105]. For mitochondria‐related gene set, the log2 fold‐change and corresponding adjusted p‐values were extracted and used to construct volcano plots with the ggplot2 package (v 3.5.2) [104].

### 4.32 Venn Diagram Analysis

To visualize the common and specific differentially expressed genes between multiple conditions, a Venn diagram was generated using the VennDiagram R package (version 1.7.1) [106]. The DEGs for each condition were first identified through differential expression analysis. Separate lists of Genes were generated for each condition to capture both the common and condition‐specific genes.

### 4.33 Correlation Coefficients Analysis Based on Pearson's Correlation Coefficient

Colocalization analysis based on Pearson's correlation coefficient (PCC) was performed using the Coloc 2 plugin in ImageJ (v1.53). Dual‐channel immunofluorescence images (red and green channels) were imported as separate stacks or merged images, and regions of interest (ROIs) were selected to exclude background and non‐relevant areas. The Coloc 2 tool was used to assign the two channels and compute PCC as the primary colocalization metric. Unless specified for noise exclusion, no additional threshold was applied; the Costes automatic thresholding method was optionally used to adaptively define thresholds based on signal randomness. The plugin output Pearson's R values (ranging from –1 to + 1, indicating perfect negative to positive correlation, with values near 0 suggesting no correlation), along with scatter plots and regression lines. All measurements were derived from at least three independent biological replicates, and data are presented as mean ± SEM. Analyses were performed under blinded conditions whenever possible.

### 4.34 Statistical Analysis

Statistical analyses were performed to ensure robust interpretation of experimental data. Data pre‐processing, including background correction, normalization to internal standards, and calculation of derived metrics (e.g., m1A/A ratio, ΔF/F), was conducted as detailed in the respective methods subsections for each assay (LC‐MS/MS, qPCR, immunofluorescence, behavioral analysis, etc.). Data are presented as the mean ± standard error of the mean (SEM) from biologically independent replicates (n), with the specific n for each experiment provided in the corresponding figure legend. Sample sizes were not predetermined by power analysis but are consistent with standards in the field. All statistical tests were two‐sided, with *p* < 0.05 considered statistically significant. Comparisons between two groups utilized an unpaired Student's t‐test for data meeting assumptions of normality and equal variance, or the Wilcoxon rank‐sum test otherwise. The correlation between ALKBH3 and PINK1 expression in human snRNA‐seq data was assessed using Spearman's rank correlation coefficient. Specific details for the analysis of calcium transients, image colocalization, and sequencing data are described in their dedicated methods subsections (“Fiber photometry recording and data analysis”, “Correlation coefficients analysis”, and “Analysis of ALKBH3 and PINK1 in human snRNA‐seq dataset”). All statistical analyses were performed using GraphPad Prism (version 8.4).

### 4.35 Data Visualization and Software

Figures were generated using BioRender, Adobe Illustrator (v27.7) and Graphpad (version 8.4).

### 4.36 Manuscript Preparation

During manuscript drafting, AI tools (ChatGPT and DeepSeek) were used solely for text refinement (grammar, syntax, and readability). All AI‐generated content was rigorously verified, edited, and approved by the authors to ensure scientific accuracy.

## Author Contributions

Y.L. and K.L. contributed to investigation, methodology, visualization, writing (original draft), software, data curation, formal analysis, and validation. Y.L., K.L., Y.W., and S.Y. created visualizations and figures. Y.Z., P.L., and T.L. were involved in mice work. M.D., P.Y., L.X., W.A., and Y.S. participated in data analysis, figure preparation, and validation. Y.Z., W.M., M.B., J.L., and P.L contributed to cell culture, methodology, and visualization. K.Z., Y.W., and S.Y. assisted with manuscript preparation. N.Y. and F.F. contributed with advice on cell culture. Y.L. and M.J.K. designed the study and supervised all research. J.Z., Y.H., F.Z., and Z.G. provided important suggestions for the project. Y.L. and M.J.K. wrote and edited the manuscript with input from all authors.

## Funding

This work was supported by CIBR's core grant, the Chinese Academy of Medical Sciences (CAMS) Innovation Fund for Medical Sciences (2019‐I2M‐5‐015), the Beijing Natural Science Foundation (IS23091), and the Beijing Postdoctoral Research Foundation Fellowship (2022‐ZZ‐005 to Y.L., 2020‐YJ‐002 to Y. P.).

## Conflicts of Interest

M.J.K. serves as an Associate Editor for Oxford Open Neuroscience. All other authors declare no competing interests.

## Supporting information




**Supporting File 1**: advs74789‐sup‐0001‐SuppMat.docx.

**Supporting File 2**: advs74789‐sup‐0002‐SupportingTables‐S1–S3.zip.

**Supporting File 3** advs74789‐sup‐0003‐MovieS1.mp4.

**Supporting File 4**: advs74789‐sup‐0004‐MovieS2.mp4.

**Supporting File 5**: advs74789‐sup‐0005‐MovieS3.mp4.

**Supporting File 6**: advs74789‐sup‐0006‐Data.zip.

## Data Availability

Human single‐nucleus RNA‐seq data used for AD versus Control analyses (Figures 1f,g, 3d, 5q, S2, S11b,c) were downloaded from NCBl Gene Expression Omnibus, (GEO) under the accession number GSE175814 (samples A1–A4; raw 10x matrices: matrix.mtx.gz, barcodes.tsv.gz, features.tsv.gz; https://www.ncbi.nlm.nih.gov/geo/query/acc.cgi?acc = GSE175814) [30]. The pipeline for human single‐nucleus RNA‐seq data analyses have been deposited in GitHub (https://github.com/KoziolLaboratory/snRNAseq_analysis_hippocampus). The raw sequencing data generated by m1A‐ID‐seq has been obtained from GEO under the accession number GSE73941. The dataset is publicly available at https://www.ncbi.nlm.nih.gov/geo/query/acc.cgi?acc = GSE73941. This dataset contains transcriptome‐wide mapping data of N1‐methyladenosine (m1A) in human embryonic kidney (HEK293T) cells, including wild‐type, *ALKBH3* knockout, and stress‐induced conditions, and was generated and publicly shared by Li et al. [27]. The significantly deregulated mRNA expression (Figure 3d) data used for comparative analysis in this study were obtained from Supplementary Table S8 part of the published article by Kumar et al. [55]. The dataset is publicly available as part of the supplementary materials of the original publication and was used in accordance with the terms of open‐access data sharing. Our mass spectrometry data related to RNA modifications and proteins has been deposited in FigShare https://doi.org/10.6084/m9.figshare.30000175. All additional software tools are cited in the methods section and are publicly available.
