# Supplementary material for: ALKBH3 m1A Demethylase Deficiency Reduces Alzheimer's Amyloid‐β Pathology
Source: Adv Sci (Weinh). 2026 Mar 12;13(32):e22572. doi: 10.1002/advs.202522572 (PMC13252633; doi:10.1002/advs.202522572)
Supplement: Supplementary file 1 — Supporting File 1: advs74789‐sup‐0001‐SuppMat.docx. [file ADVS-13-e22572-s001.docx]

Supporting Information

Title

ALKBH3 m1A Demethylase Deficiency Reduces Alzheimer’s Amyloid-β Pathology

Yueyang Li^1,2,3^, Sifei Yu^2,3,4^, Kaidong Lu^2,3,5^, Yujie Zhang^2,3^, Mingjie Dong^2,3^, Yan Peng^1,2,3,6^, Liang Xue^2,3,6^, Waleed Alam^2,3^, Yuxuan Shui^1,2,3^, Yi Zhou^2,3,7^, Wuyunhan Ma^2,3^, MengBao^2,3^, Peiming Li^2,3^, Peiyi Luo^2,3^, Tiezhan Lu^2,3^, Jiajia Li^2,3^, Kang Zhang^2,3,7^, Yuying Wang^2,3,5^, Shuchen Yang^2,3,8^, Nuoya Yin^9,10^, Francesco Faiola^9,10^, Zilong Gao^2,3^, Jingfeng Zhou^2,3,11^, Fei Zhao^2,3^, Yali He^2,3^, Magdalena J. Koziol^2,3,6^

**
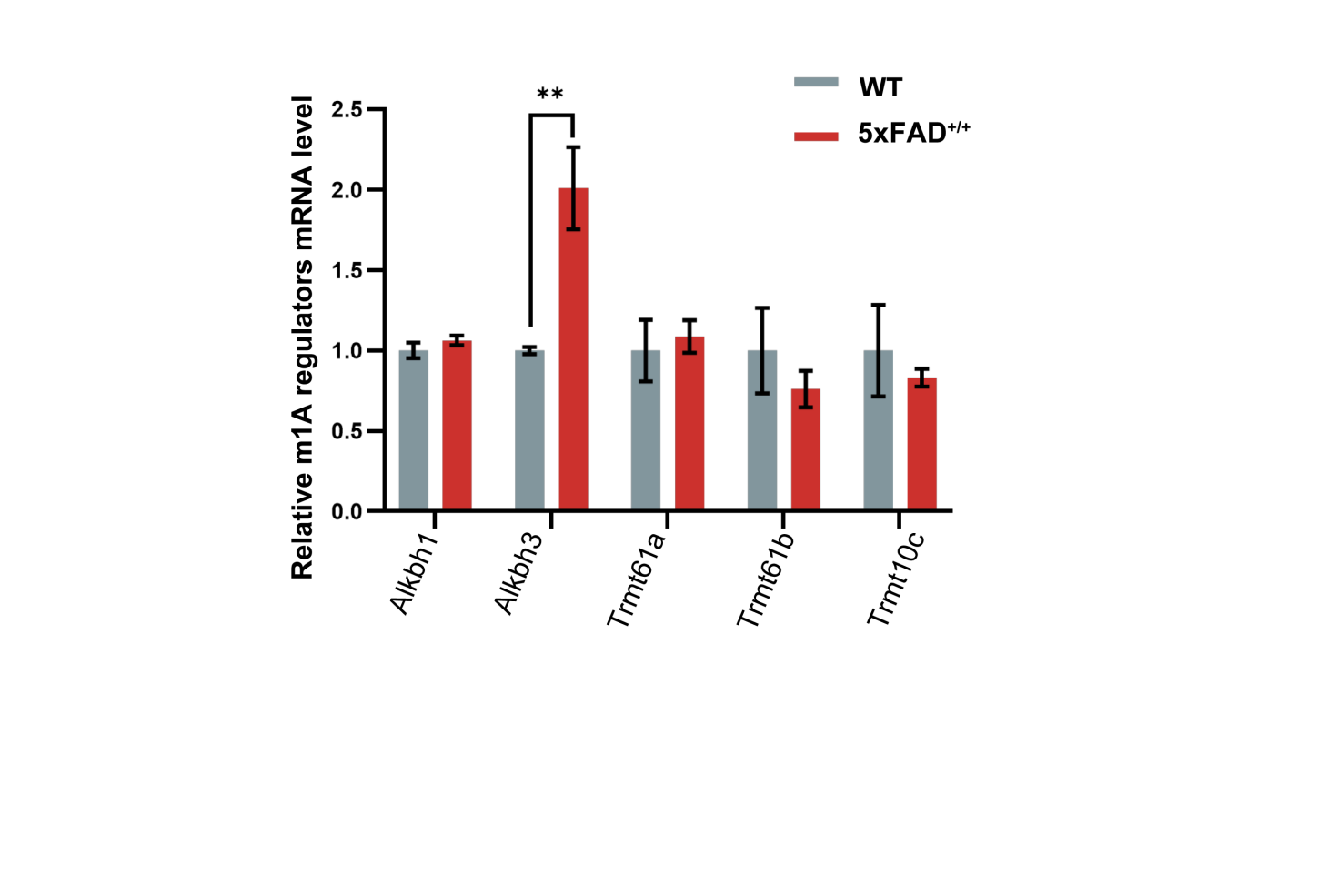
**

**Figure S1.** **m1A-related mRNA Enzymes in Mouse Hippocampi.** RT-qPCR of m1A-related mRNA enzymes in hippocampi;

**
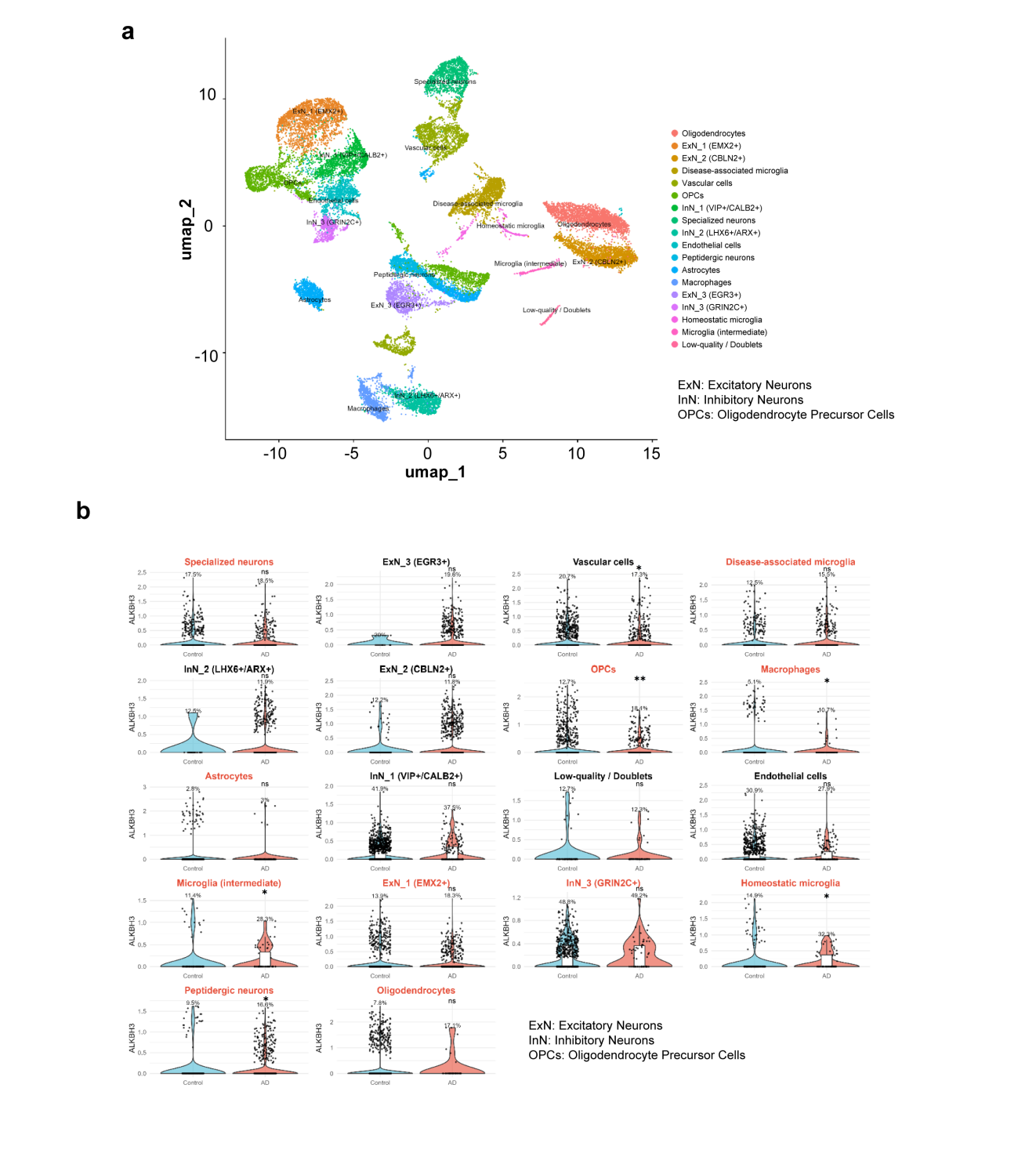
**

**Figure S2.** **ALKBH3 expression levels in hippocampal single-cell transcriptomes**

1. Annotated UMAP representation of human hippocampal single-cell transcriptomes showing major cell populations;
2. Cell-type-specific enrichment of ALKBH3-expressing cells in AD. Violin plots show ALKBH3 expression across major annotated neuronal and glial cell populations in Control and AD samples at single-nucleus resolution. Percentages above each violin indicate the proportion of ALKBH3-expressing cells per cell type and condition. Cell-type labels highlighted in red denote populations with a higher proportion of ALKBH3-expressing cells in AD compared with Control. Statistical significance was assessed using the Wilcoxon rank-sum test, with ns > 0.05 or *p < 0.05, **p < 0.01. ExN: Excitatory Neurons; InN: Inhibitory Neurons; OPCs: Oligodendrocyte Precursor Cells;

**
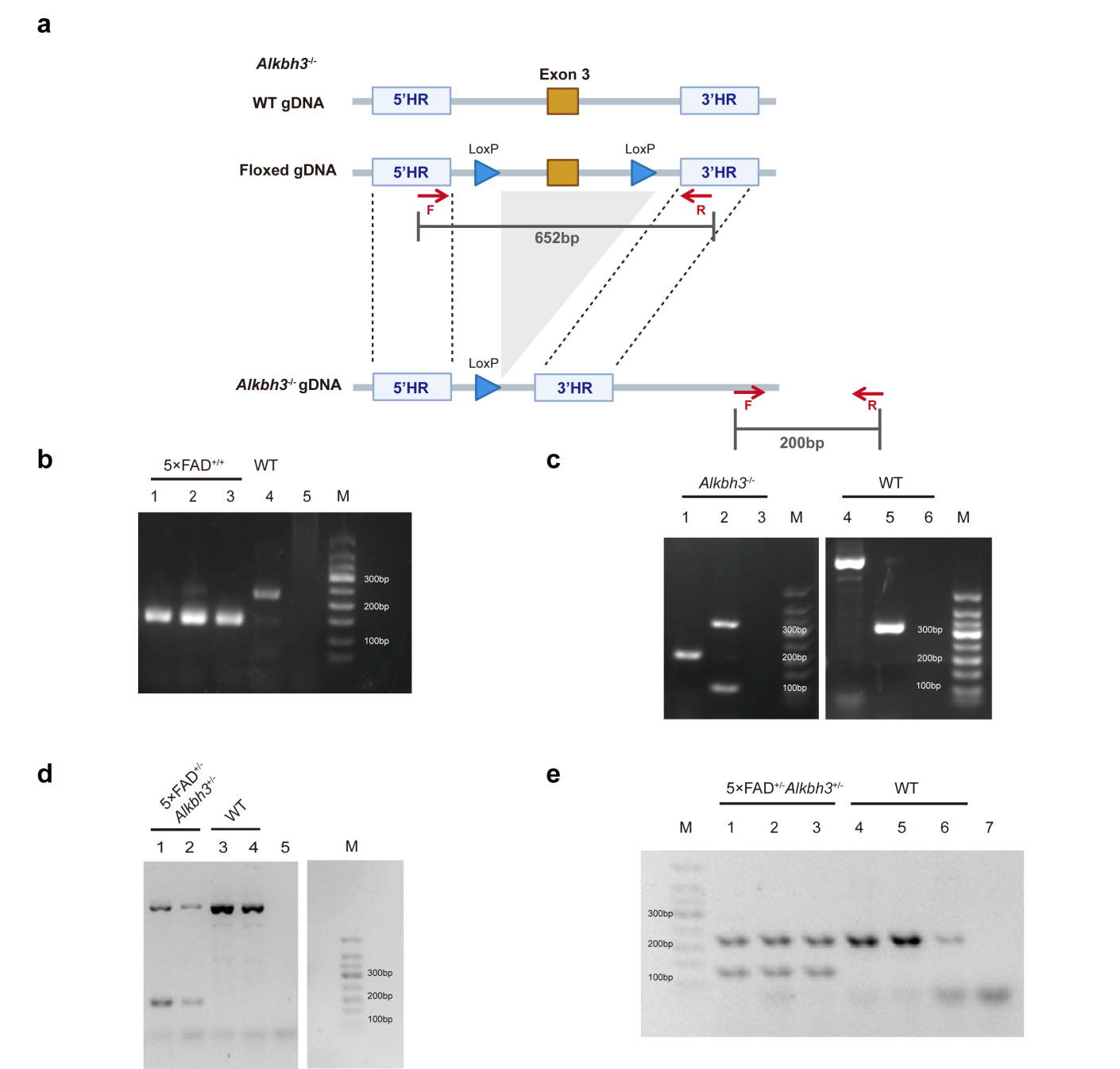
**

**Figure S3.** **Mouse Models and Experimental Design.**

1. Schematic of the *Alkbh3*^fl/-^ and *Alkbh3*^-/-^ alleles. Exon 3 (yellow) is flanked by loxP sites (triangles). Genome coordinates are based on the GRCm38/mm10 assembly: chr2:93,978,624-94,012,824. The genotyping strategy is indicated with primer binding sites (arrows) and expected PCR product sizes. PCR primers (F and R, see Extended Data Table S3) yield expected products of 652 bp for the WT allele and 200 bp for the Alkbh3^-^ allele).
2. Genotyping strategy for FAD^+/+^ *vs.* WT mice using PCR primers FAD-F/R; expected products: WT=216 bp, Mutant=129 bp;
3. Genotyping strategy for *Alkbh3*^-/-^ mice using PCR primers F/R (Extended Data Table S3); expected products: WT=652 bp, KO=200 bp;
4. Genotyping strategy for 5xFAD^+/-^*Alkbh3*^+/-^ mice (evaluating *Alkbh3*) using PCR primers F/R; expected products: WT=652 bp, KO=200 bp;
5. Genotyping strategy for 5xFAD^+/-^*Alkbh3*^+/-^ mice (evaluating 5xFAD) using PCR primers FAD-F/R; expected products: WT=216 bp, Mutant=129 bp;

**
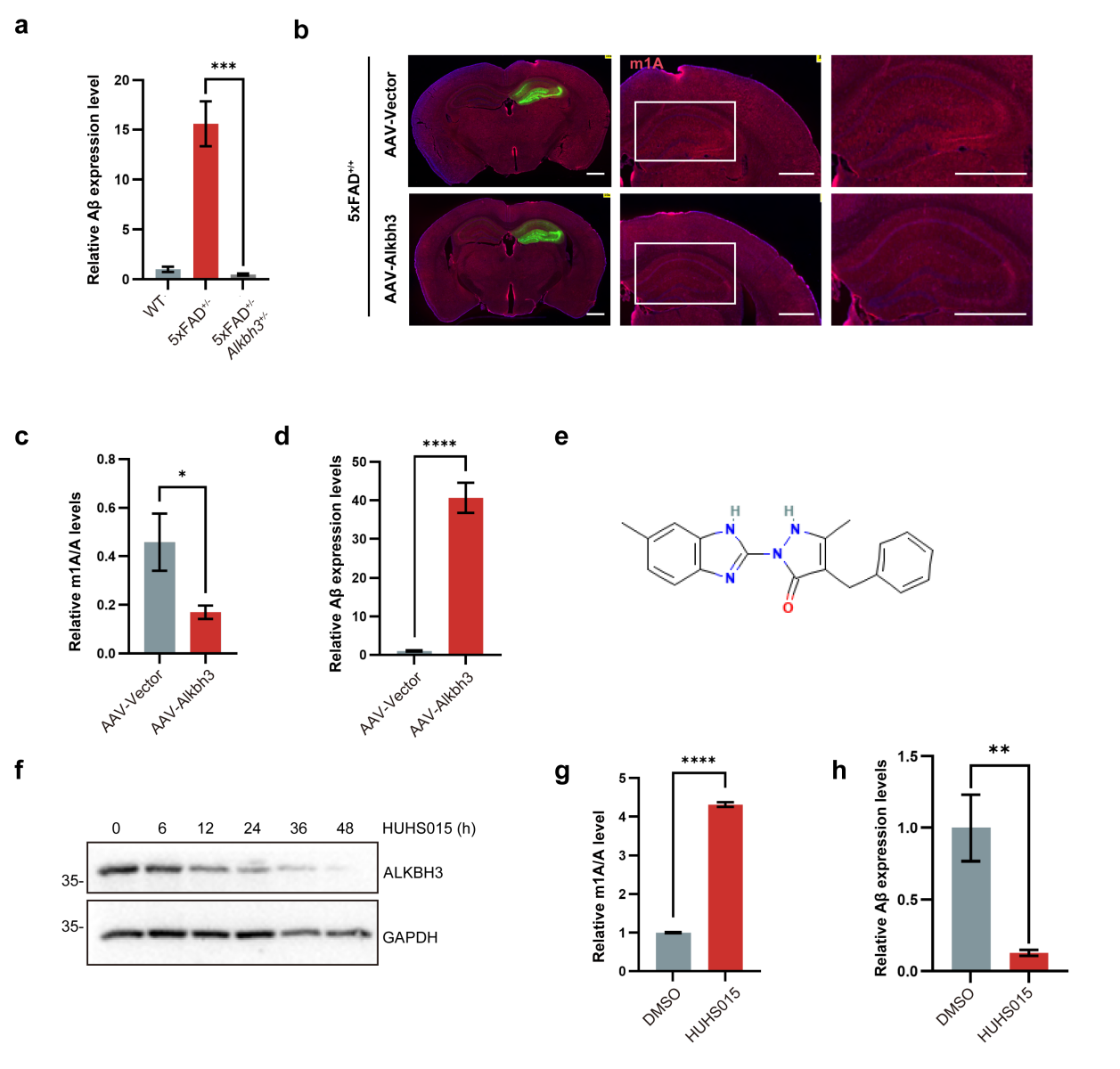
**

**Figure S4. Impact of ALKBH3 Modulation on m1A Levels.**

1. Quantification of Aβ protein levels in 8-month-old 5xFAD^+/-^*Alkbh3^+/-^* *vs.* 5xFAD^+/-^ hippocampi by WB;
2. m1A levels (red, IF) and Alkbh3 (green, IF) in AAV-Vector *vs.* AAV-Alkbh3 hippocampi in 5xFAD^+/+^ mice (scale bar: 0.5 mm);
3. m1A level in AAV-Vector *vs.* AAV-Alkbh3 hippocampi in 5xFAD^+/+^ mice. (LC-MS/MS);
4. Quantification of Aβ protein levels in AAV-mediated ALKBH3 overexpression in mouse hippocampi by WB;
5. Structural schematic of ALKBH3 inhibitor HUHS015;
6. ALKBH3 protein levels under HUHS015 treatment at indicated time points in SH-SY5Y cells (WB; GAPDH as loading control);
7. m1A levels in SH-SY5Y cells treated with DMSO or HUHS015 treated SH-SY5Y cells (LC-MS/MS);
8. Quantification of Aβ protein levels in DMSO *vs.* HUHS015 in SH-SY5Y cells overexpressing APP;

Representative images shown for IF (blue for DAPI) and WB; two-tailed unpaired t-test; data: mean ± SEM; n = 3 biological replicates unless noted; *p < 0.05, **p < 0.01, ***p < 0.001, ****p < 0.0001;

**
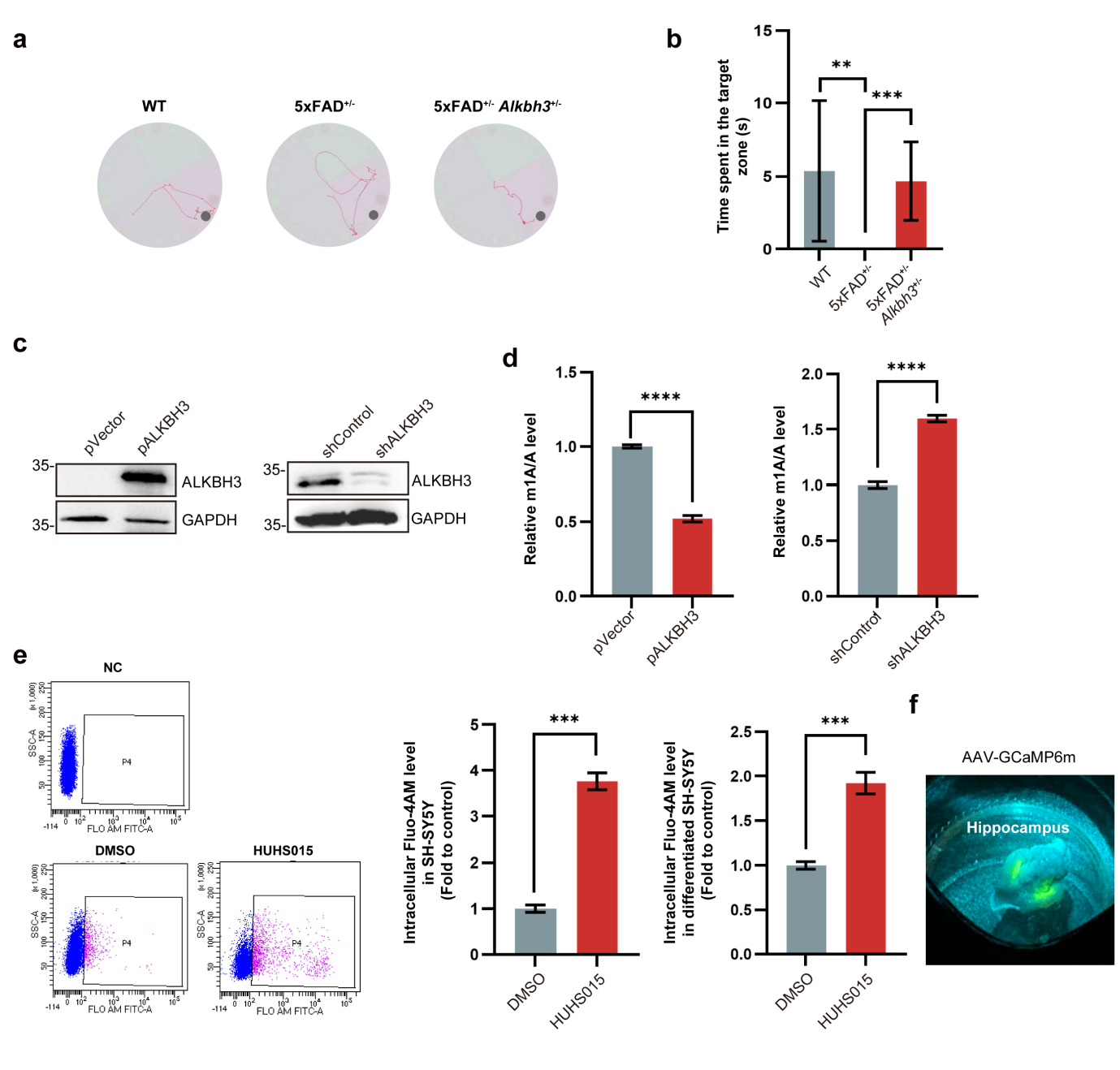
**

**Figure S5. Behavioral, and Cellular Phenotypes of ALKBH3 Modulation.**

1. Representative movement traces of WT, 5xFAD^+/-^ and 5xFAD^+/-^*Alkbh3*^+/-^ mice in Barnes Maze (7-8/group);
2. Time spent in the target or zone (s) (n = 7–8/group);
3. ALKBH3 levels in *ALKBH3* knockdown and ALKBH3 overexpressing SH-SY5Y cells (WB; GAPDH as loading control);
4. m1A levels in *ALKBH3* knockdown and ALKBH3 overexpressing SH-SY5Y cells (LC-MS/MS);
5. Fluo-4AM intensity of SH-SY5Y cells ±HUHS015 quantified by FACS;
6. Representative image of hippocampi injected with AAV-GCaMP6m;

Representative images shown for WB, FACS, and hippocampi; two-tailed unpaired t-test; data: mean ± SEM; n = 3 biological replicates unless noted; **p < 0.01, ***p < 0.001, ****p < 0.0001;

**
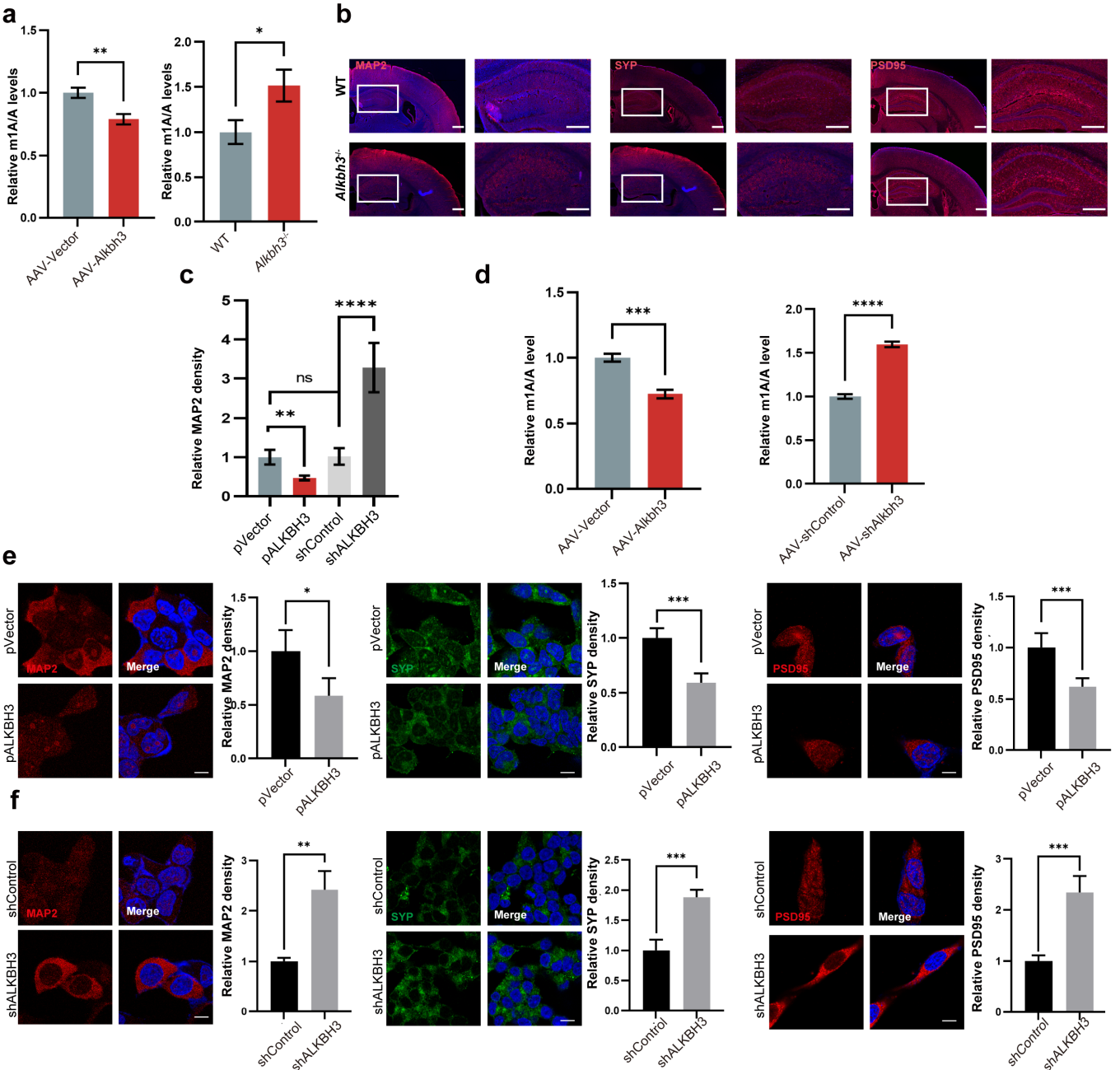
**

**Figure S6. ALKBH3 Induced Brain and Cellular Changes.**

1. m1A levels in AAV-Alkbh3 *vs.* AAV-Vector WT mice and WT vs. *Alkbh3*^-/-^ mice (LC-MS/MS);
2. IF images of MAP2, SYP and PSD95 deposition (red) in 6-month-old WT *vs.* *Alkbh3*^-/-^ mice (scale bar: 0.5 mm);
3. Quantification of MAP2 density (IF) in pVector *vs.* pALKBH3, shControl *vs.* shALKBH3 and pVector *vs.*shALKBH3 in primary neurons;
4. m1A levels in ALKBH3-modulated primary hippocampal neurons (LC-MS/MS);
5. IF images and quantification of MAP2, SYP and PSD95 in pVector *vs.* pALKBH3 in SH-SY5Y cells (scale bar: 10 μm);
6. IF images and quantification of MAP2, SYP and PSD95 in shControl *vs.* shALKBH3 in SH-SY5Y cells (scale bar: 10 μm);

Representative images shown for IF (blue for DAPI) and WB; two-tailed unpaired t-test; data: mean ± SEM; n = 3 biological replicates unless noted; scale bar: 0.5 mm; *p < 0.05, **p < 0.01, ***p < 0.001, ****p < 0.0001;

**
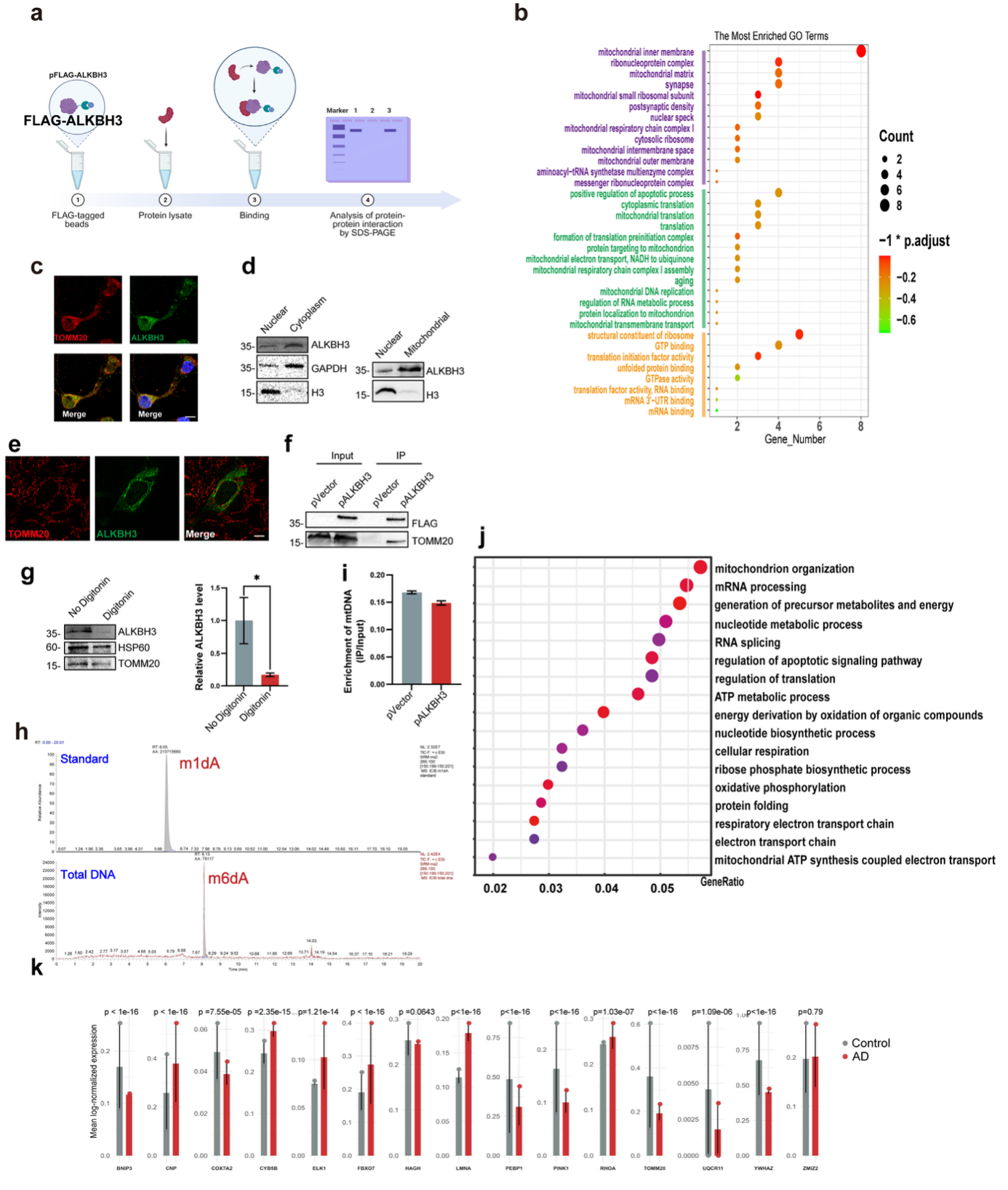
**

**Figure S7. ALKBH3 Interactome and Functional Enrichment Analyses.**

1. Schematic of FLAG-ALKBH3 pull down assay and protein mass spectrometry;
2. Gene Ontology (GO) enrichment of high-confidence ALKBH3-interacting proteins (FDR <0.01). Top categories are shown (Extended Data Table S1);
3. IF of ALKBH3 and TOMM20 in primary neurons (scale bar: 10 μm; IF);
4. WB of nuclear *vs.* cytoplasm and nuclear *vs.* mitochondrial isolated fractions;
5. IF by super‑resolution microscopy (Zeiss Elyra‑7) of ALKBH3 and TOMM20. (scale bar: 7.5 μm; red: TOMM20; green: ALKBH3);
6. WB analysis of co-immunoprecipitation (co-IP) using an anti-FLAG antibody to pull down FLAG-tagged ALKBH3, followed by immunoblotting for TOMM20;
7. WB and quantification of protein distribution in intact mitochondria *vs.* mitochondria with the outer membrane selectively removed through digitonin treatment;
8. LC-MS/MS detection of m1dA in DNA from HEK293T cells. The m1dA standard showed a retention time of 6.05 min. At the corresponding retention time, no m1dA peak was detected in other samples;
9. Enrichment of mtDNA (IP/Input) in pVector vs. pALKBH3;
10. Analysis of 940 m1A-modified transcripts identified by m1A-ID-seq in *ALKBH3* knockout 293T cells. Top pathways are shown (Extended Data Table S1);
11. Differential expression levels of 15 candidate transcripts detected in human Control *vs.* AD samples. p-values are indicated in the figure;

Representative images shown for IF (blue for DAPI) and WB; two-tailed unpaired t-test; data: mean ± SEM; n = 3 biological replicates unless noted; *p < 0.05;

**
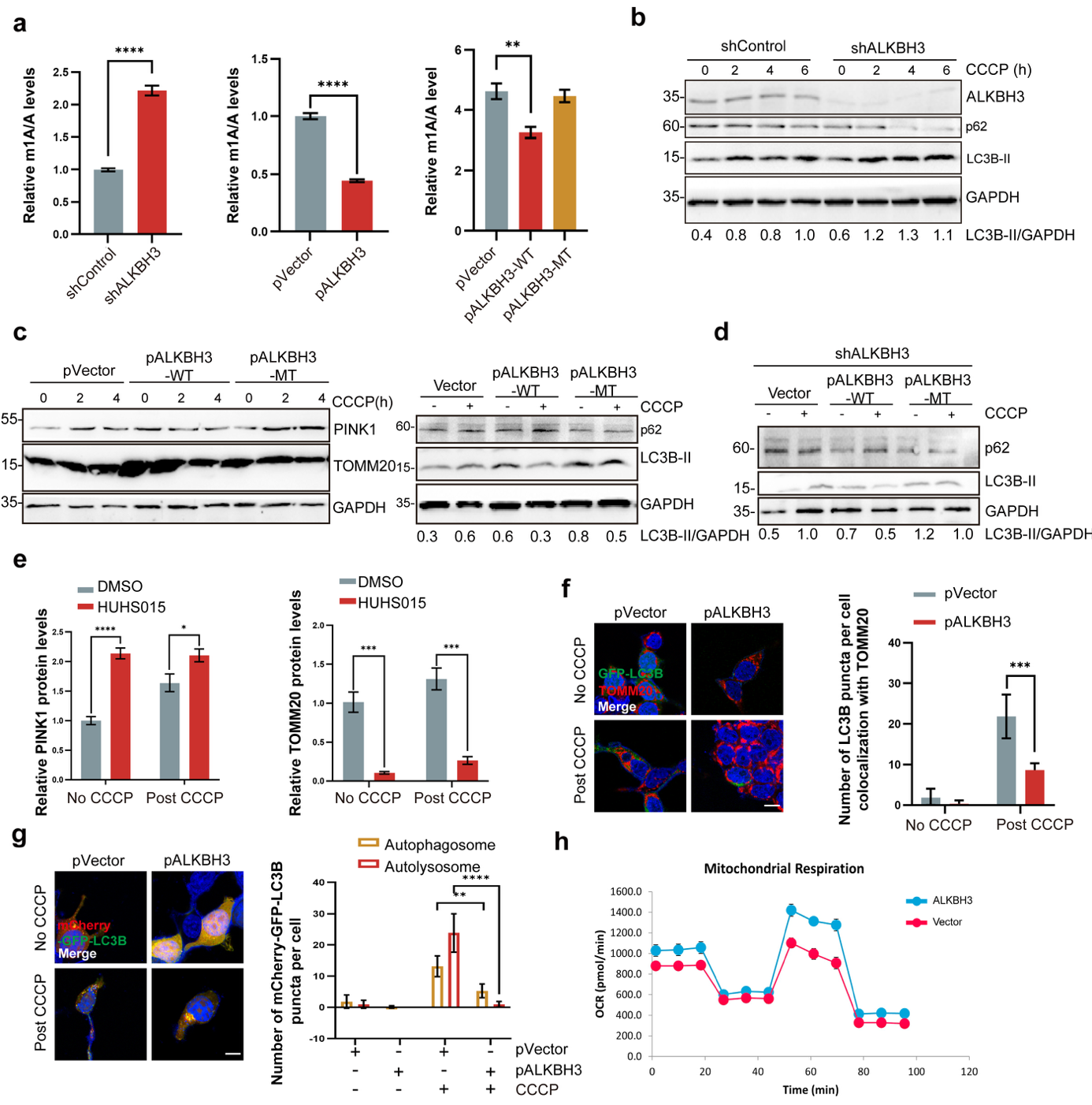
**

**Figure S8. ALKBH3 Regulates Mitophagy and Metabolic Flux in HEK293T Cells.**

1. m1A levels in ALKBH3-modulated HEK293T cells (LC-MS/MS);
2. Autophagy markers (p62/LC3B-II) in shControl *vs.* shALKBH3 (WB; GAPDH as loading control; ± CCCP);
3. Mitophagy markers (PINK1/TOMM20) and autophagy markers (p62/LC3B-II) in pALKBH3-WT/MT (WB; GAPDH as loading control; ± CCCP);
4. Autophagy markers (p62/LC3B-II) in rescue experiments with pALKBH3-WT/MT in shALKBH3 (WB; GAPDH as loading control; ± CCCP);
5. Quantification of PINK1 and TOMM20 protein levels detected by WB (as shown in Fig. 3i.);
6. (i) IF images of GFP-LC3B (green) and TOMM20 (red) in pALKBH3 *vs.* pVector control (scale bar: 10 μm); (ii) Quantification of GFP-LC3B and TOMM20 colocalization in pALKBH3 *vs.* pVector control (n = 6);
7. (i) mCherry-GFP-LC3B puncta in pALKBH3 *vs.* pVector control (scale bar: 10 μm); (ii) Quantification of mCherry-GFP-LC3B puncta in pALKBH3 *vs.* pVector control (n = 6);
8. Oxygen consumption rate (OCR) profiles of pALKBH3 vs. pVector controls assessed by Seahorse XF Analyzer;

Representative images shown for IF (blue for DAPI) and WB; WB images were analyzed by ImageJ software (v1.53); two-tailed unpaired t-test; data: mean ± SEM; n = 3 biological replicates unless noted; **p < 0.01, ***p < 0.001, ****p < 0.0001;

**
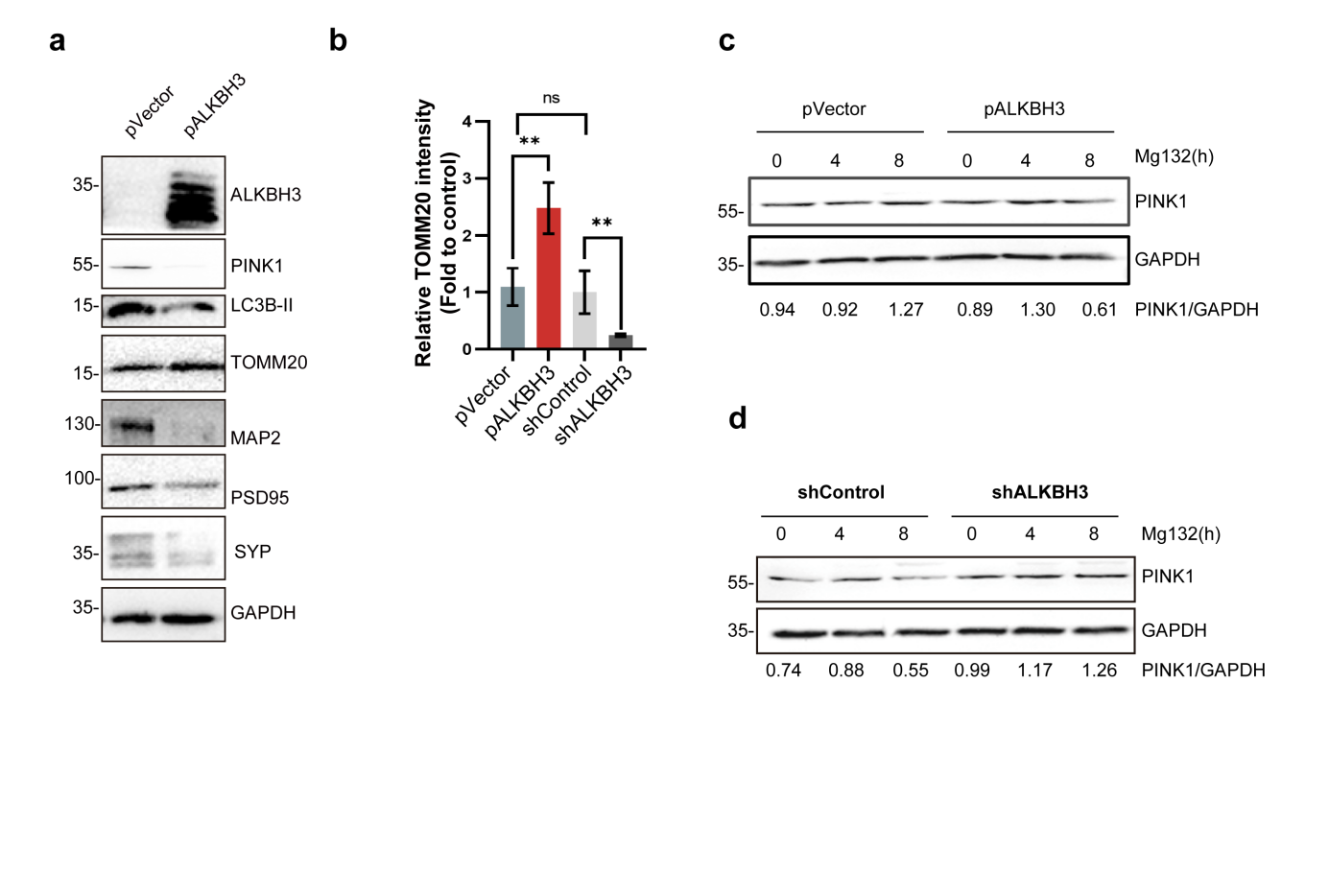
**

**Figure S9. Protein Levels Associated with ALKBH3 Modulation.**

1. WB confirmation of ALKBH3 overexpression in SH-SY5Y cells transfected with pALKBH3 (*vs.* pVector control). GAPDH serves as loading control. Representative images shown; n = 3 biological replicates;
2. Quantification of TOMM20 density (IF) in pVector *vs.* pALKBH3, shControl *vs.* shALKBH3 and pVector *vs.*shALKBH3 in SH-SY5Y cells.
3. Proteasomal degradation of PINK1 in HEK293T cells overexpressing ALKBH3 (pALKBH3) *vs.* vector control. Cells treated with MG132) to block proteasomal degradation. Top: Representative WB for PINK1 and GAPDH. Bottom: band intensity quantification by ImageJ software (v1.53); n = 3 biological replicates;
4. PINK1 stabilization in ALKBH3-depleted cells (shALKBH3) vs. shControl under MG132 treatment. Top: Representative WB for PINK1 and GAPDH. Bottom: band intensity quantification by ImageJ software (v1.53); n = 3 biological replicates;

Representative images shown for WB; WB images were analyzed by ImageJ software (v1.53); two-tailed unpaired t-test; data: mean ± SEM; n = 3 biological replicates unless noted; ns: p > 0.05; **p < 0.001;

**
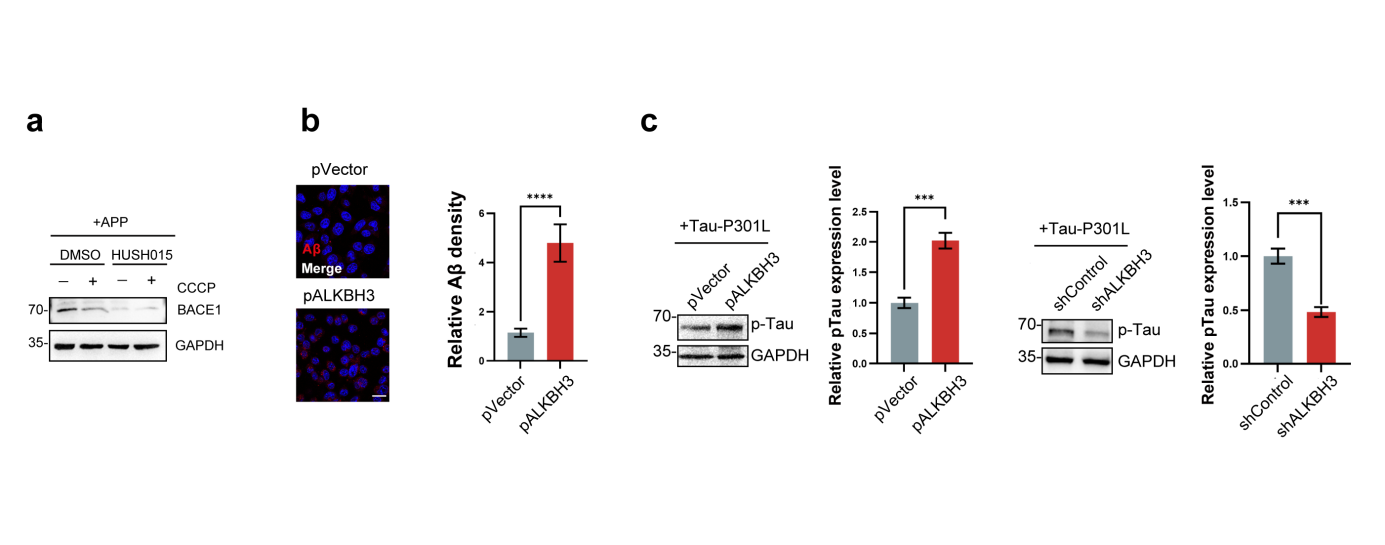
**

**Figure S10. Analysis of Aβ and Tau Pathways.**

1. BACE1 protein levels in response to DMSO *vs.* HUHS015 treatment in SH-SY5Y cells overexpressing APP (WB, ± CCCP), with GAPDH as loading control;
2. IF images and quantifications of Aβ in pVector *vs.* pALKBH3 overexpression in SH-SY5Y cells overexpressing APP (scale bar: 20 μm);
3. Representative images and quantification of pTau expression by WB in pVector *vs.* pALKBH3 and shControl *vs.* shALKBH3 in SH-SY5Y cells;

Representative images shown for IF (blue for DAPI) and WB; WB images were analyzed by ImageJ software (v1.53); two-tailed unpaired t-test; data: mean ± SEM; n = 3 biological replicates unless noted; ***p < 0.001, ****p < 0.0001;


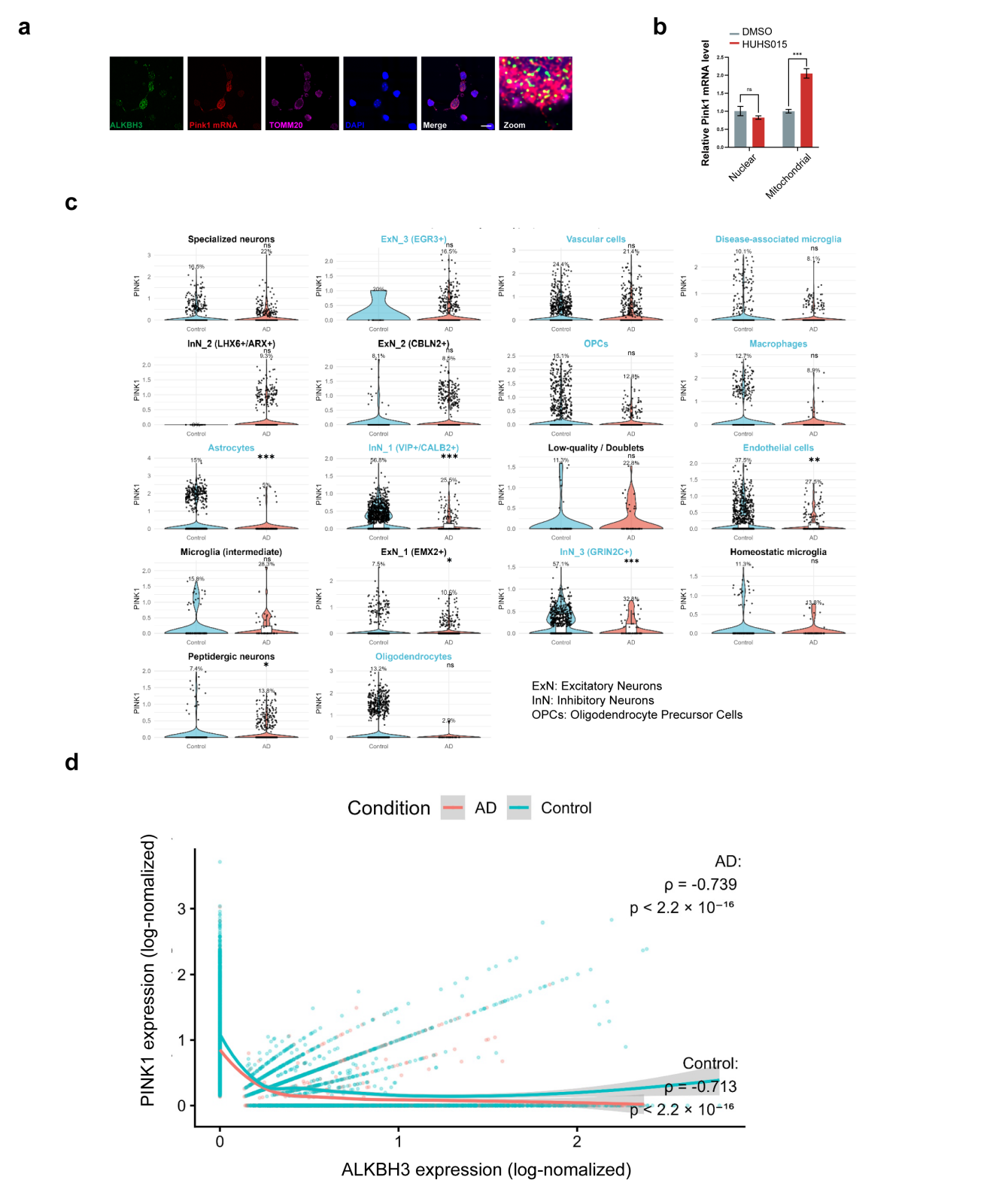


**Figure S11. Relationship between ALKBH3 and PINK1.**

1. IF *in situ* hybridization (FISH) for PINK1 mRNA combined with immunofluorescence for ALKBH3 and the mitochondrial outer‑membrane marker TOMM20 in primary neurons; Representative images are shown (scale bar: 30 μm; n=3 repeats/group);
2. Pink1 mRNA level in nuclei and mitochondria after DMSO *vs.* HUHS015 treatment. Data: mean ± SEM, n = 3 repeats/group. ns: p > 0.05, ***p < 0.001;
3. Cell-type–specific PINK1 expression in human Control versus AD conditions. Violin plots show PINK1 expression across major neuronal and glial cell types in Control and AD samples at single-nucleus resolution. Cell-type labels highlighted in blue denote clusters with a higher proportion of PINK1-expressing cells in Control compared with AD. Percentage indicates proportion of cells. Wilcoxon rank-sum test, with p-values explicitly indicated on the plots. Statistical significance was assessed using the Wilcoxon rank-sum test, with ns > 0.05, *p < 0.05, **p < 0.01, ***p < 0.001. ExN: Excitatory Neurons; InN: Inhibitory Neurons; OPCs: Oligodendrocyte Precursor Cells;
4. Spearman correlation analysis of ALKBH3 and PINK1 expression. AD: Spearman ρ = −0.739, p < 2.2 × 10⁻¹⁶; Control: Spearman ρ = −0.713, p < 2.2 × 10⁻¹⁶;

**Table S1**

ALKBH3 association with mitophagy.

**Table S2**

Differentially expressed genes between Alzheimer’s Disease and control samples.

**Table S3**

List of guide RNAs, specific primers used for qPCR and genotyping and antibodies used for WB and IF.

Movie S1.

WT Barnes maze experiment. Representative videao is shown.

Movie S2.

5×FAD Barnes maze experiment. Representative videao is shown.

Movie S3.

5×FAD Alkbh3 KO Barnes maze experiment. Representative videao is shown.
