# Supplementary material for: ALKBH3 m1A Demethylase Deficiency Reduces Alzheimer's Amyloid‐β Pathology
Source: Adv Sci (Weinh). 2026 Mar 12;13(32):e22572. doi: 10.1002/advs.202522572 (PMC13252633; doi:10.1002/advs.202522572)

**1g**

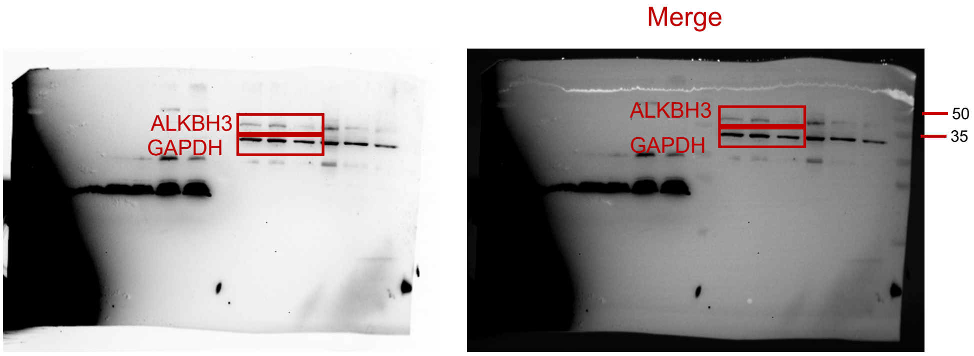
Merge
ALKBH3
GAPDH
ALKBH3
GAPDH
50
35

**1k**

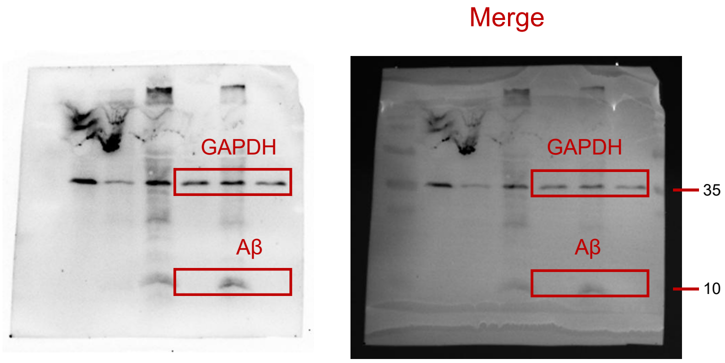
Merge
GAPDH
Aβ
GAPDH
Aβ
35
10

**1m**

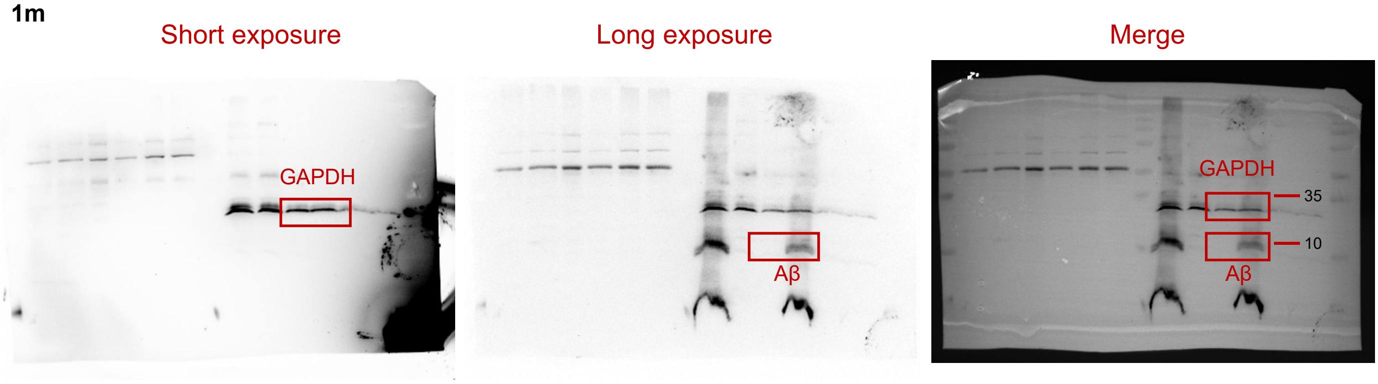
Short exposure
Long exposure
Merge
GAPDH
Aβ
GAPDH
35
10
Aβ

**1p**

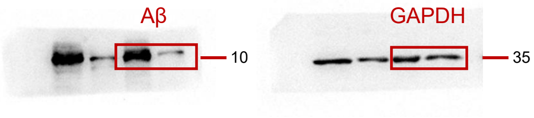
Aβ
10
GAPDH
35

**2g**

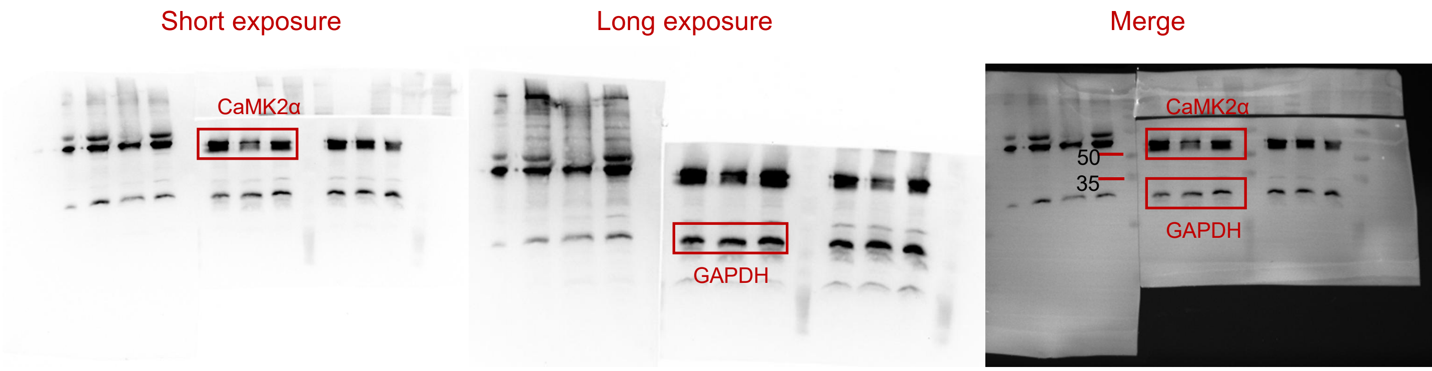

Short exposure
Long exposure
Merge
CaMK2α
GAPDH
CaMK2α
50
35
GAPDH


**2k**

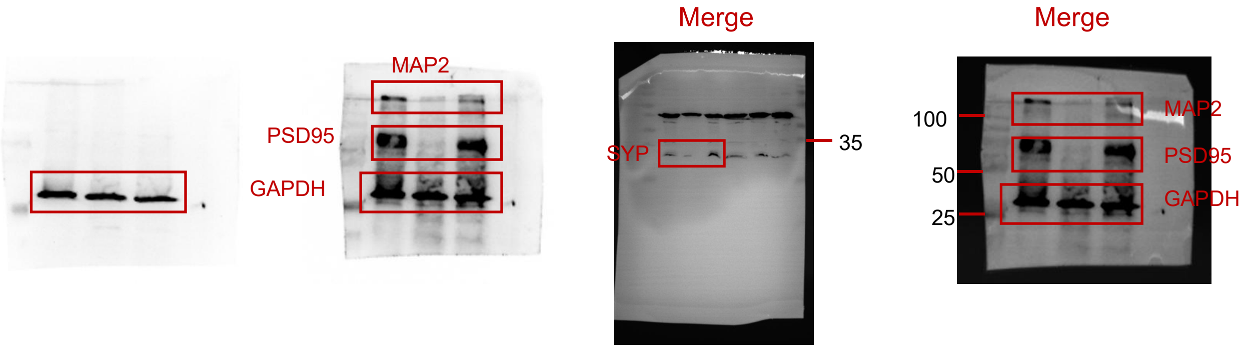

MAP2
PSD95
GAPDH
Merge
SYP
35
Merge
100
50
25
MAP2
PSD95
GAPDH

**3g**

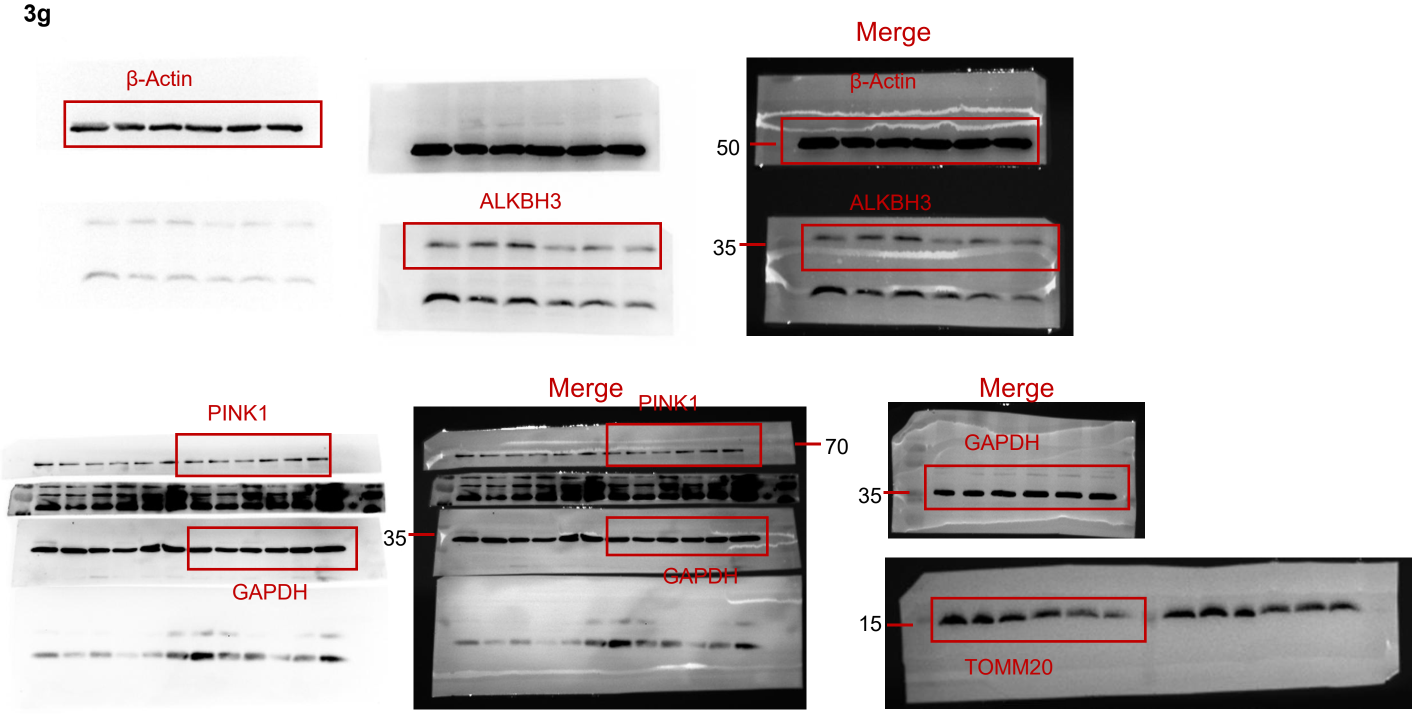
β-Actin
Merge
β-Actin
50
ALKBH3
ALKBH3
35
PINK1
Merge
PINK1
70
Merge
GAPDH
35
35
GAPDH
GAPDH
15
TOMM20

**3h**

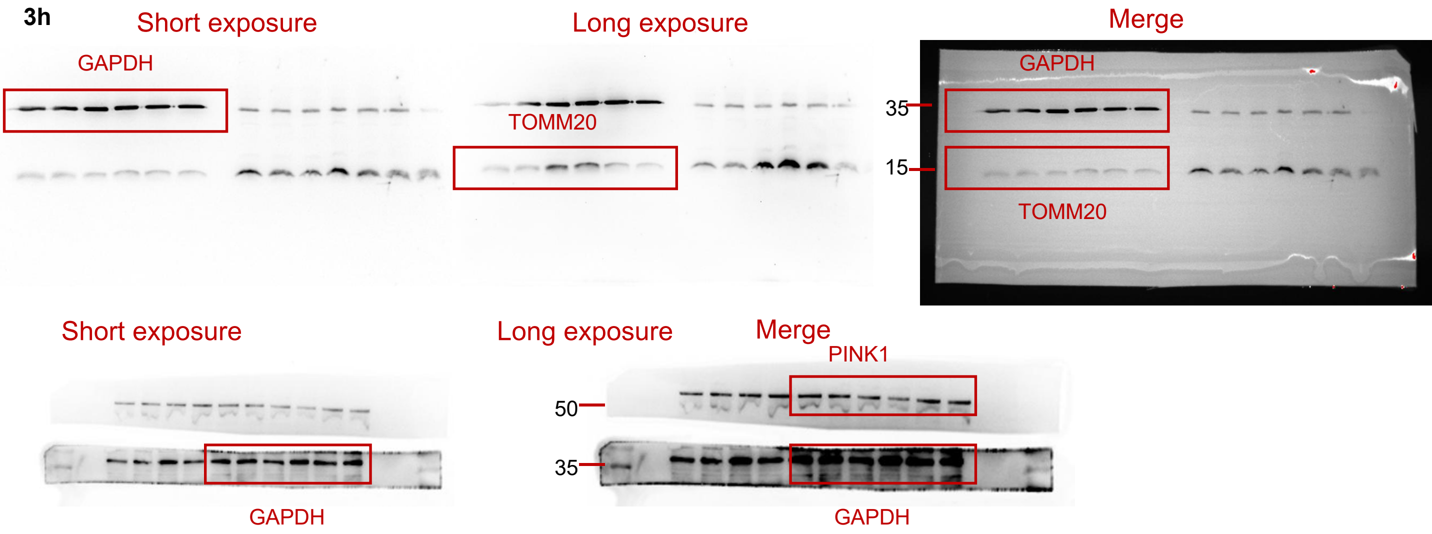
Short exposure
Long exposure
Merge
GAPDH
GAPDH
35
TOMM20
15
TOMM20
Short exposure
Long exposure
Merge
PINK1
50
35
GAPDH
GAPDH

**3i**

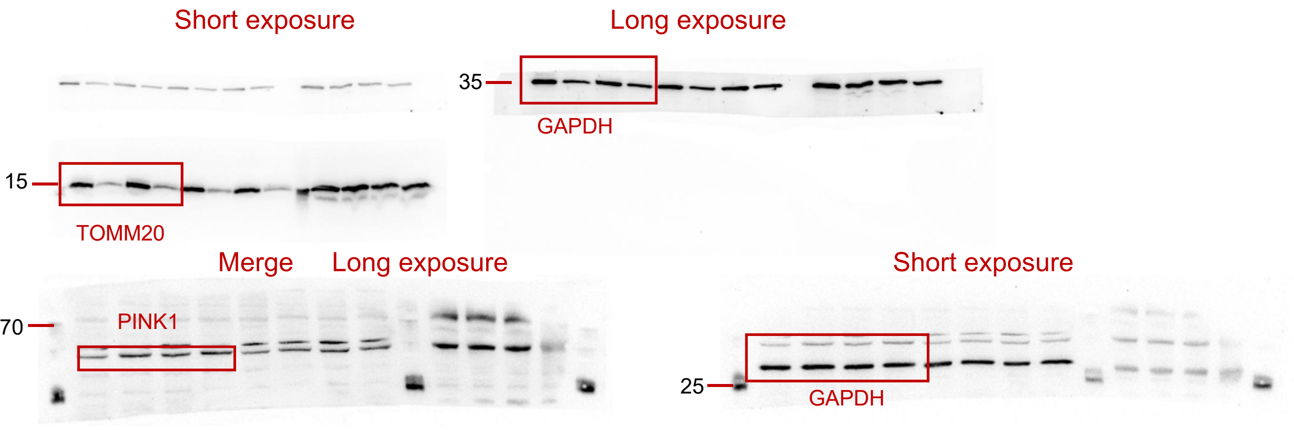
Short exposure
Long exposure
35
GAPDH
15
TOMM20
Merge
Long exposure
Short exposure
70
PINK1
25
GAPDH

4a

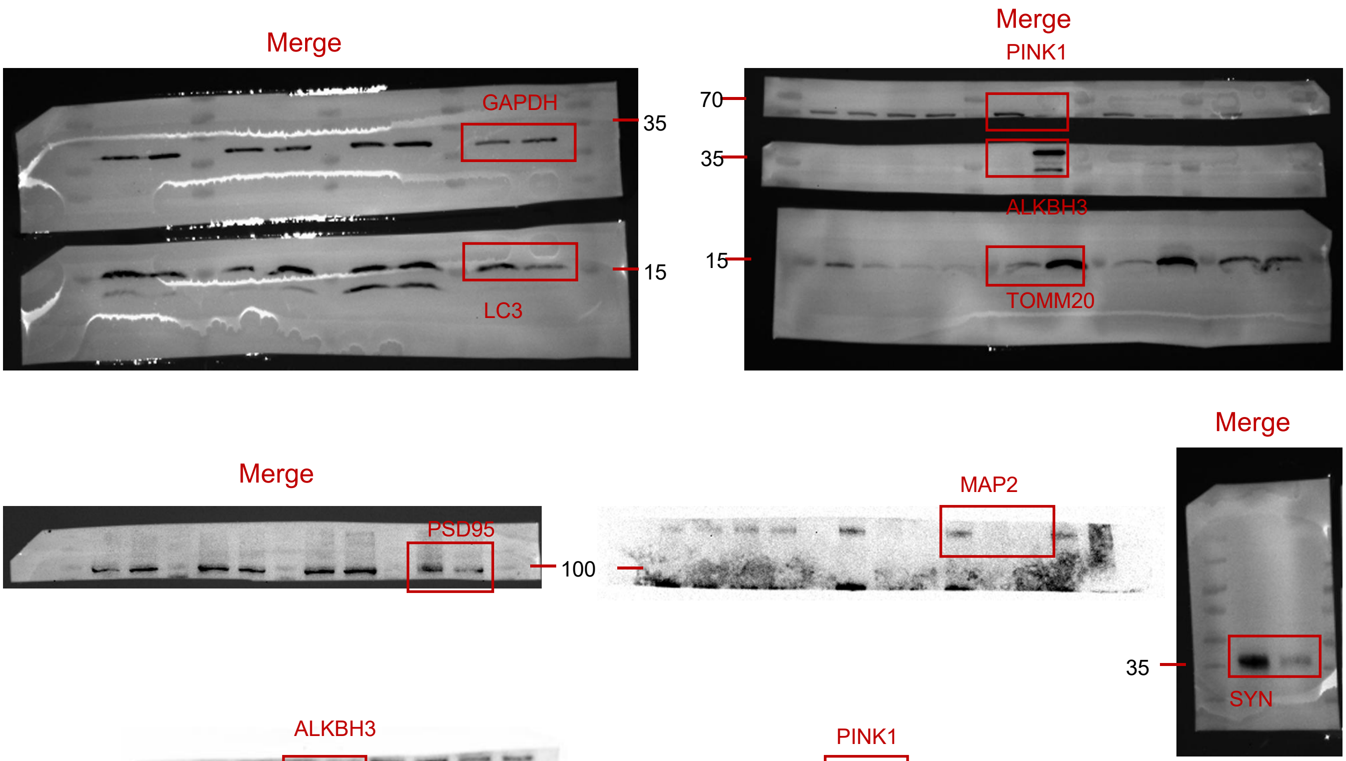
Merge
GAPDH
35
15
LC3
Merge
PINK1
70
35
ALKBH3
15
TOMM20
Merge
PSD95
100
MAP2
Merge
35
SYN

4j

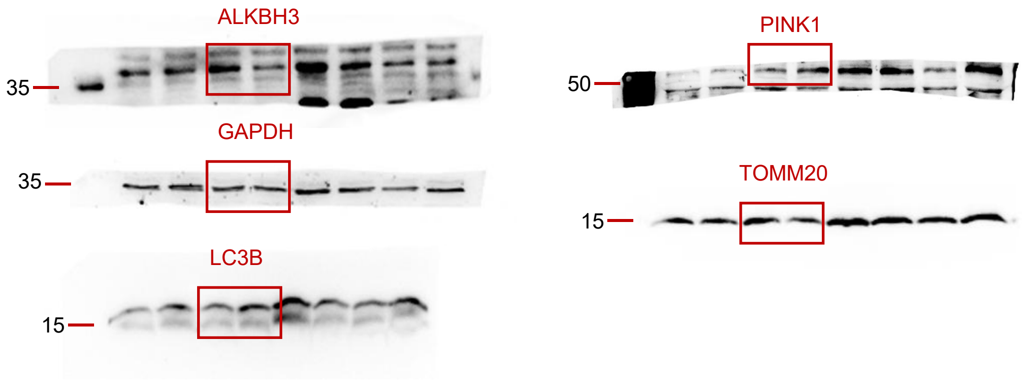
ALKBH3
35
GAPDH
35
LC3B
15
PINK1
50
TOMM20
15

4k

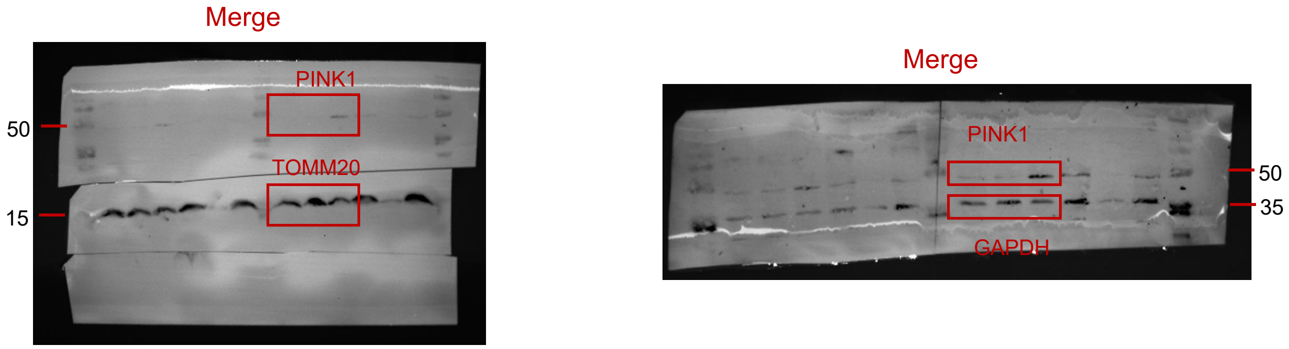
Merge
PINK1
50
TOMM20
15
Merge
PINK1
50
35
GAPDH

**5k**

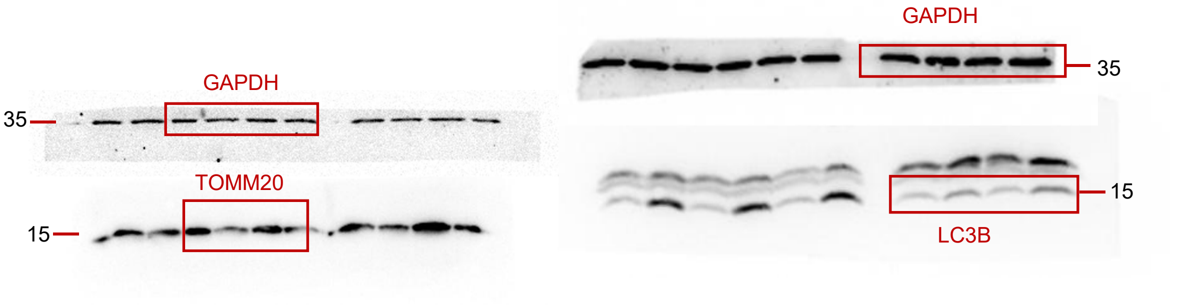
GAPDH
35
TOMM20
15
GAPDH
35
15
LC3B

**5p**

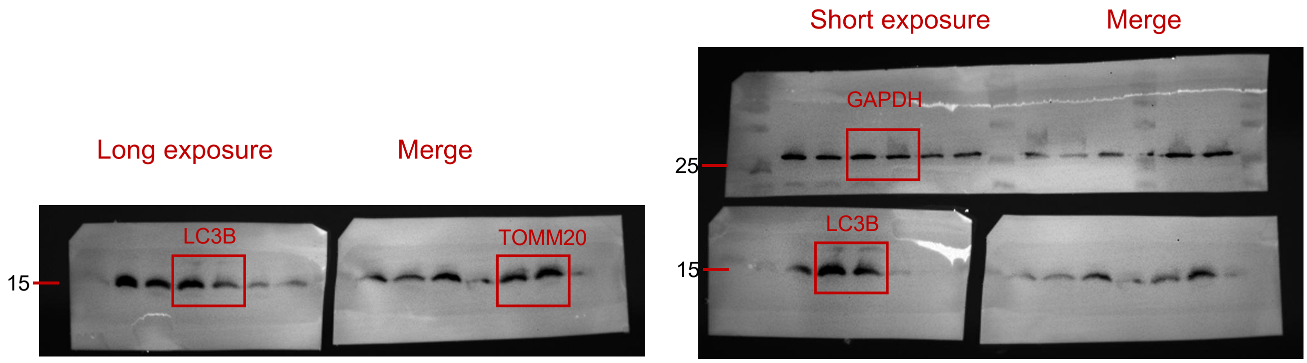
Long exposure
Merge
LC3B
TOMM20
15
Short exposure
Merge
GAPDH
25
LC3B
15

**Supplimental**

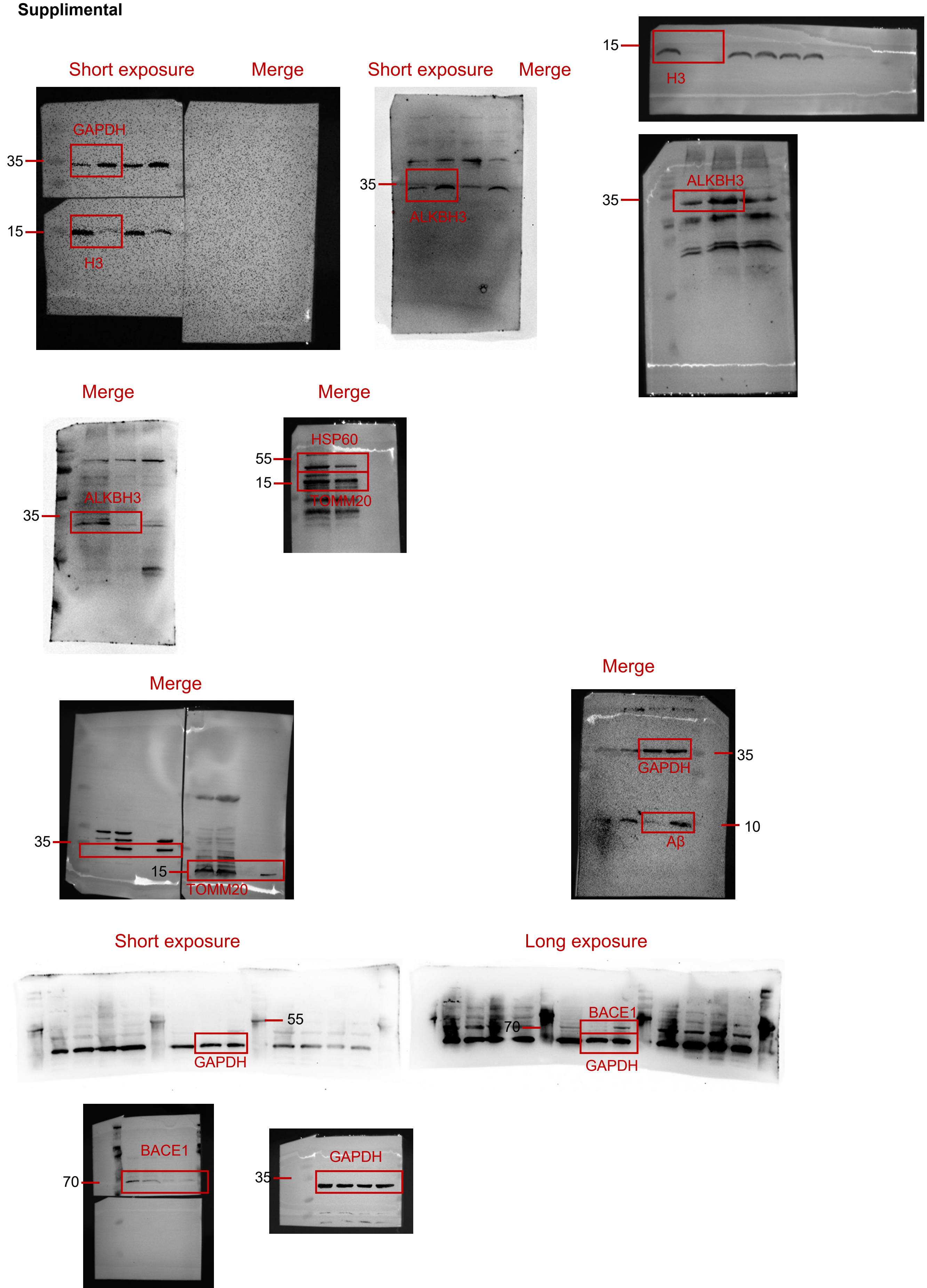

Supplement: Supplementary file 6 — Supporting File 6: advs74789‐sup‐0006‐Data.zip. [file ADVS-13-e22572-s004.zip › raw data WB.pdf]
