# Supplementary figures and images for: ALKBH3 m1A Demethylase Deficiency Reduces Alzheimer's Amyloid‐β Pathology
Source: Adv Sci (Weinh). 2026 Mar 12;13(32):e22572. doi: 10.1002/advs.202522572 (PMC13252633; doi:10.1002/advs.202522572)

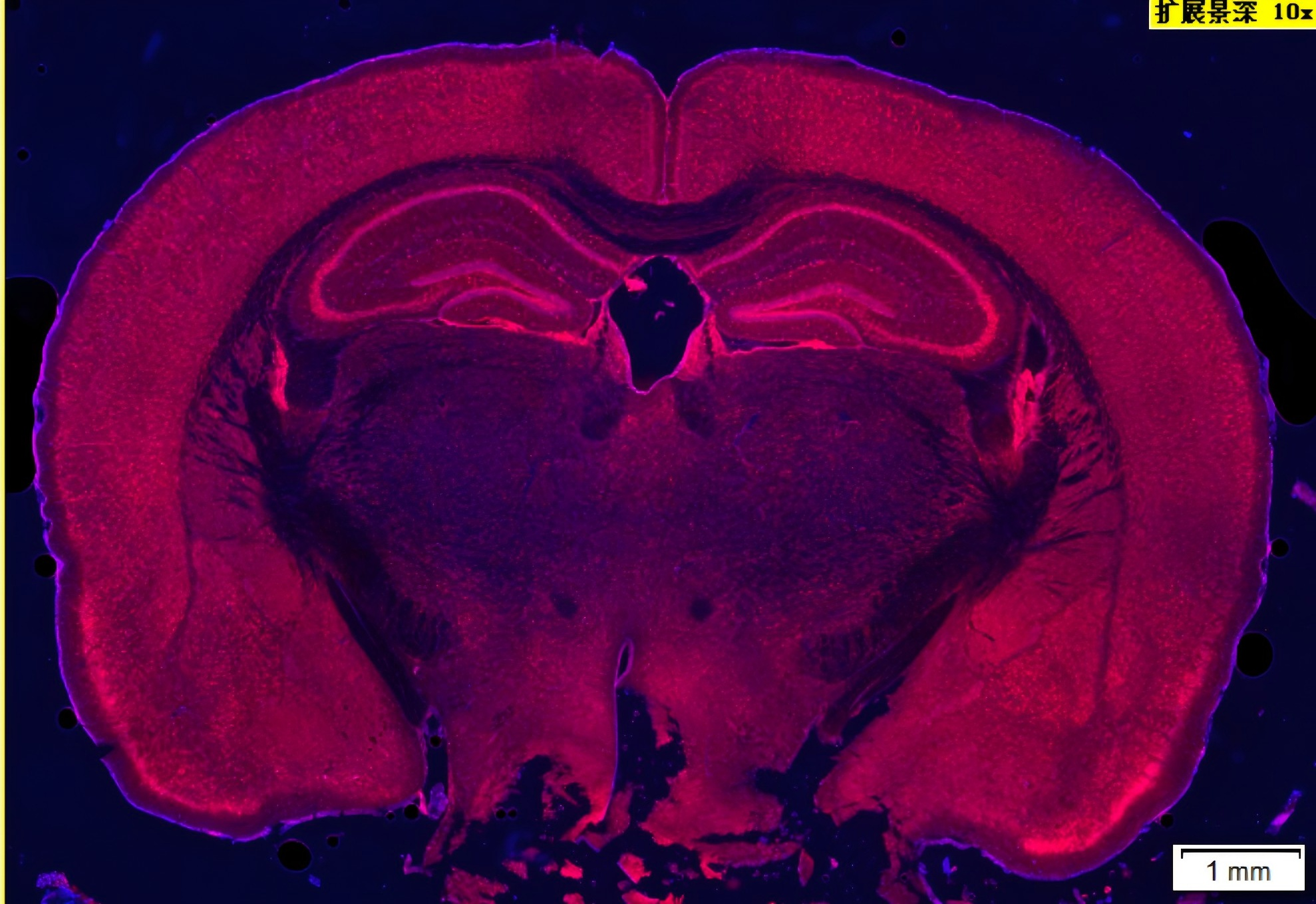

Supplement: Supplementary file 6 — Supporting File 6: advs74789‐sup‐0006‐Data.zip. [file ADVS-13-e22572-s004.zip › Alkbh3-KO_m1A.tif]

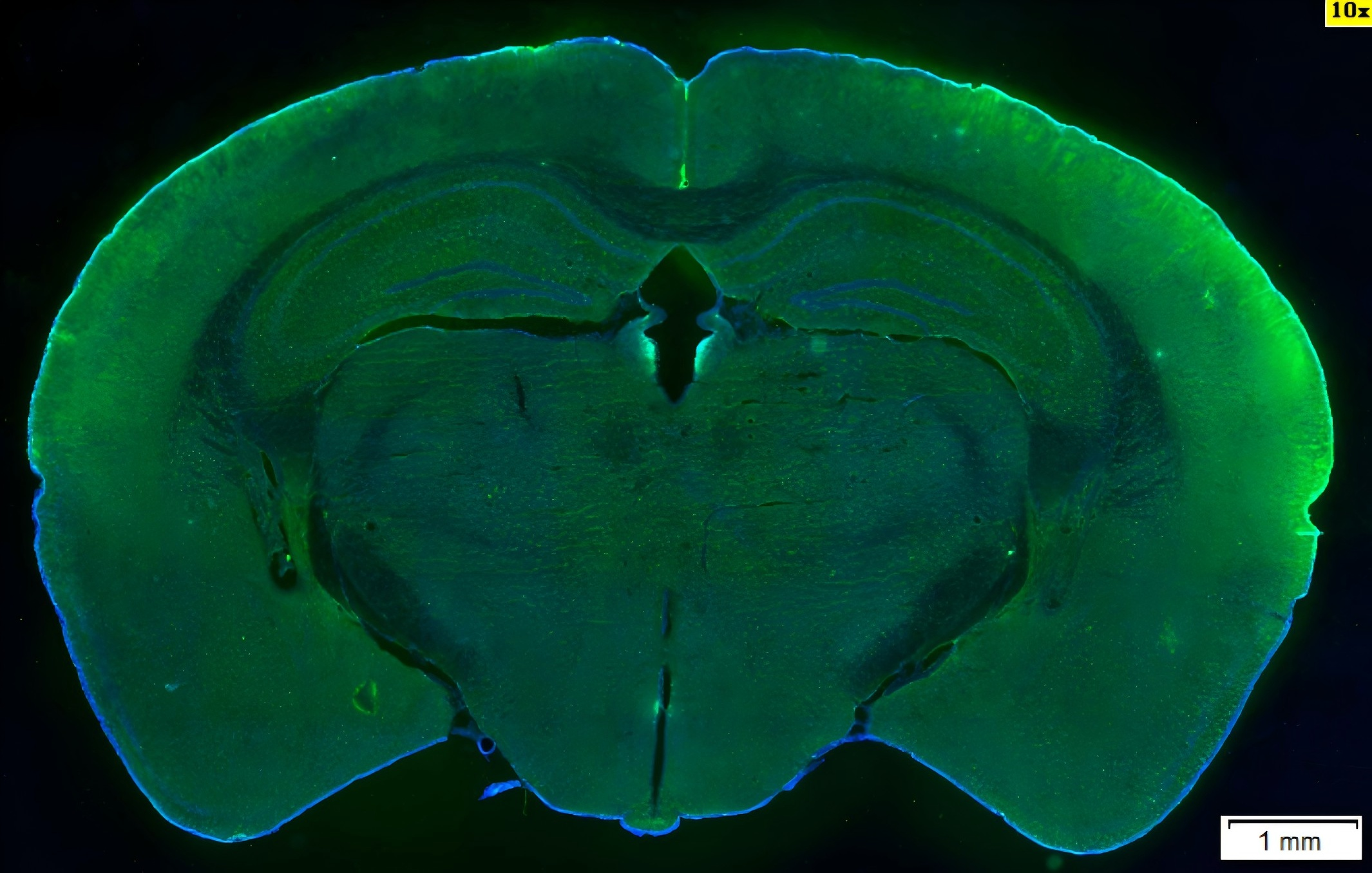

Supplement: Supplementary file 6 — Supporting File 6: advs74789‐sup‐0006‐Data.zip. [file ADVS-13-e22572-s004.zip › renamed_0d60a.tif]

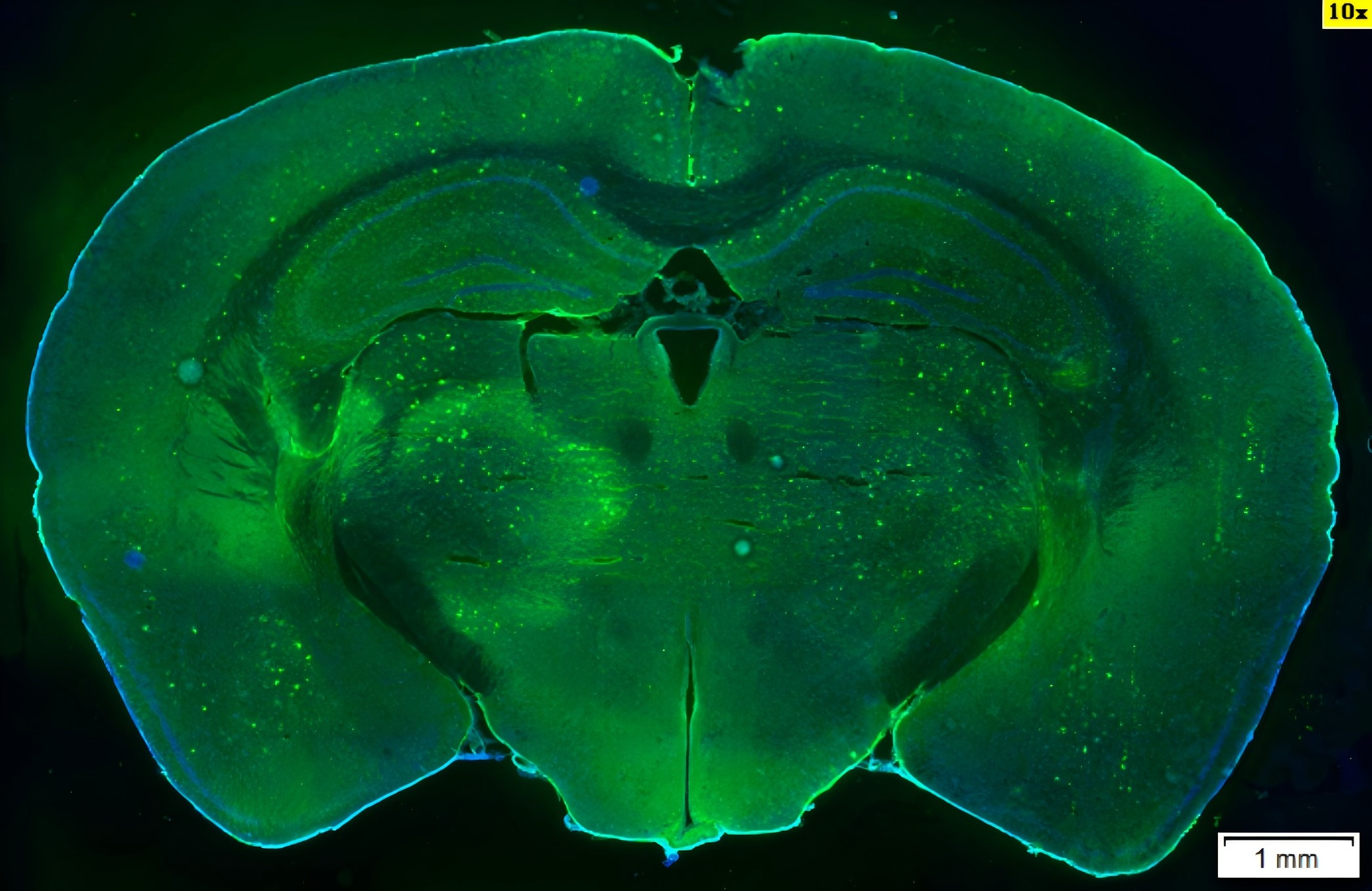

Supplement: Supplementary file 6 — Supporting File 6: advs74789‐sup‐0006‐Data.zip. [file ADVS-13-e22572-s004.zip › renamed_72129.tif]

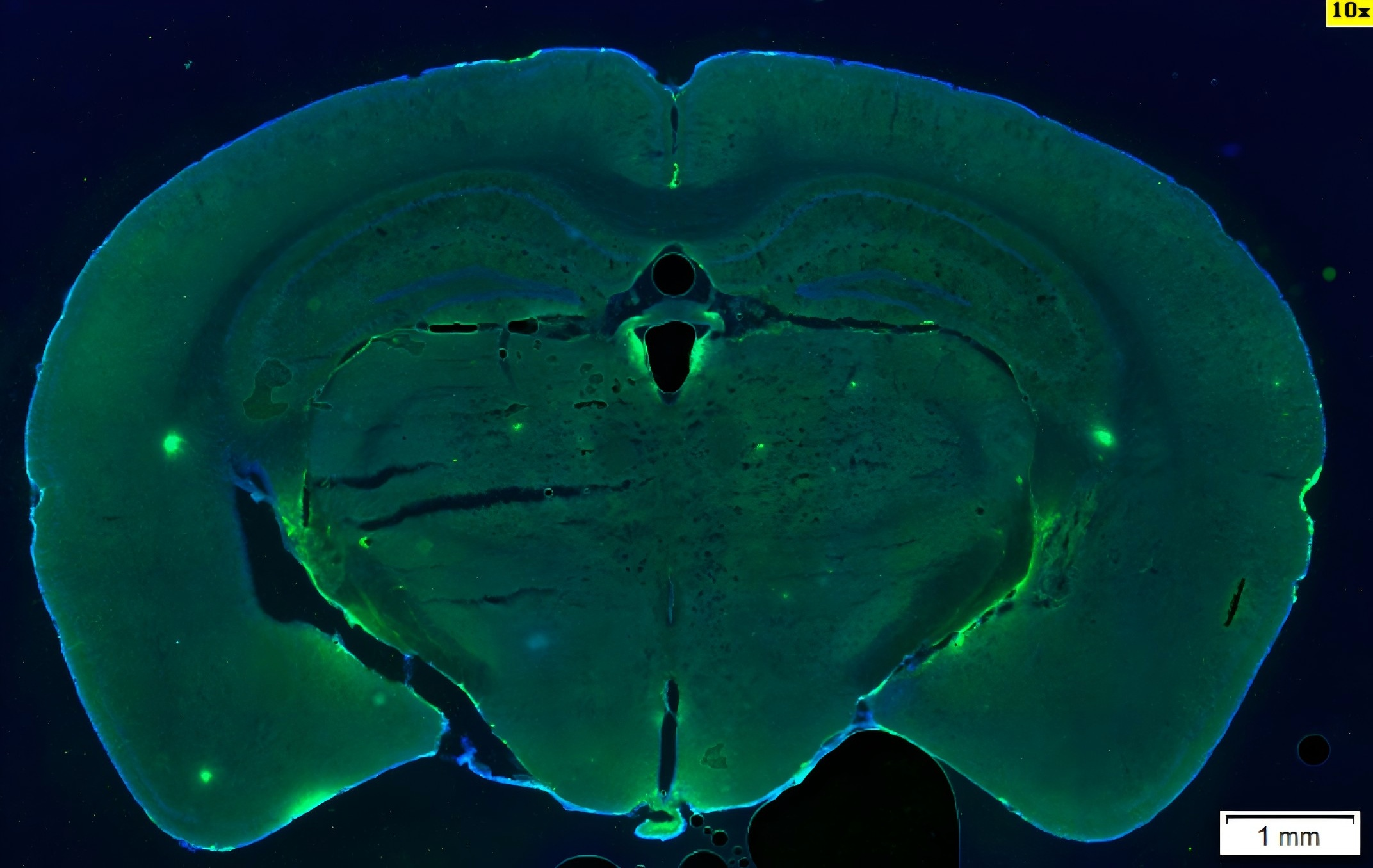

Supplement: Supplementary file 6 — Supporting File 6: advs74789‐sup‐0006‐Data.zip. [file ADVS-13-e22572-s004.zip › WT_ALK.tif]

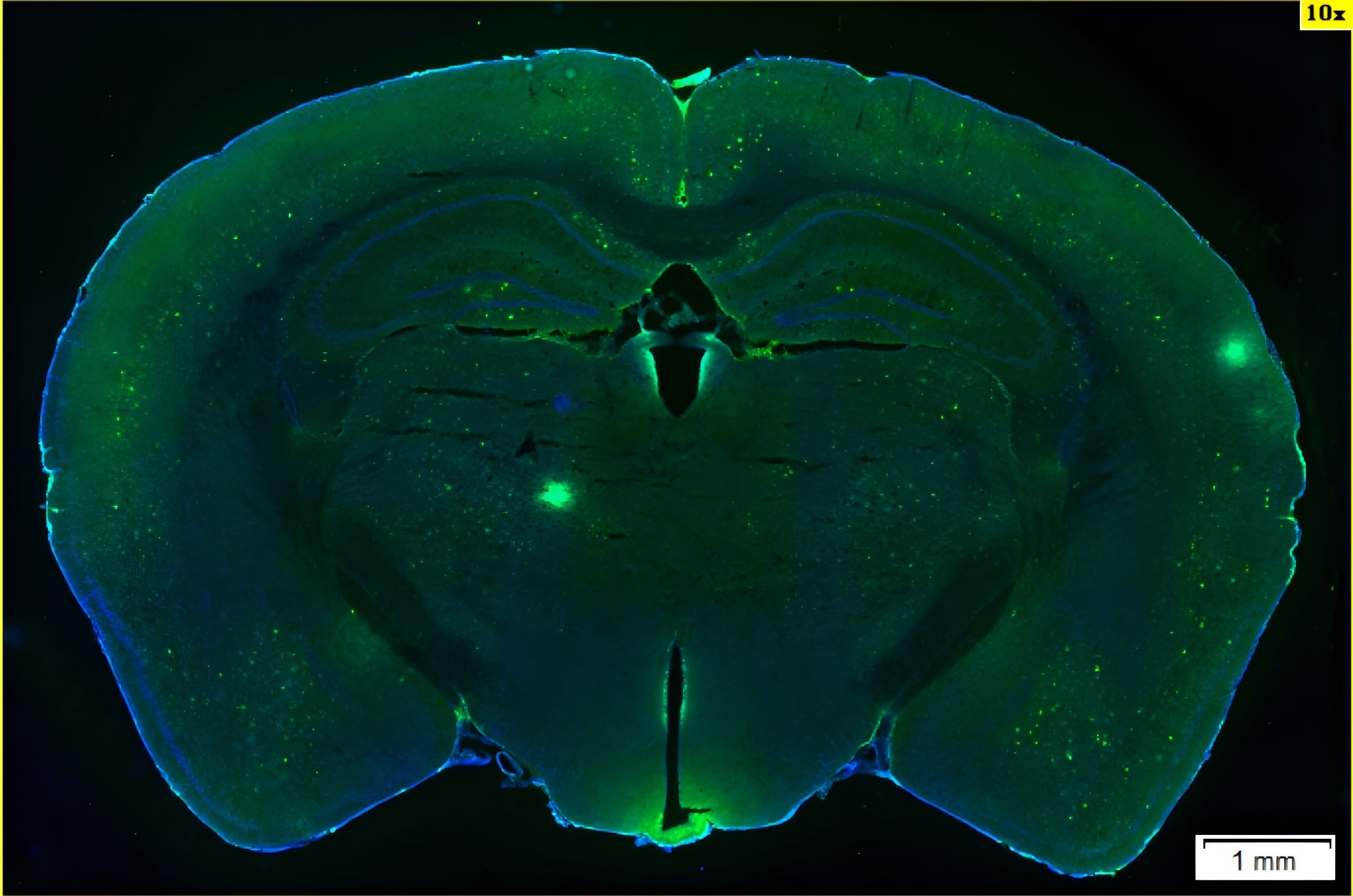

Supplement: Supplementary file 6 — Supporting File 6: advs74789‐sup‐0006‐Data.zip. [file ADVS-13-e22572-s004.zip › FAD_ALK.tif]

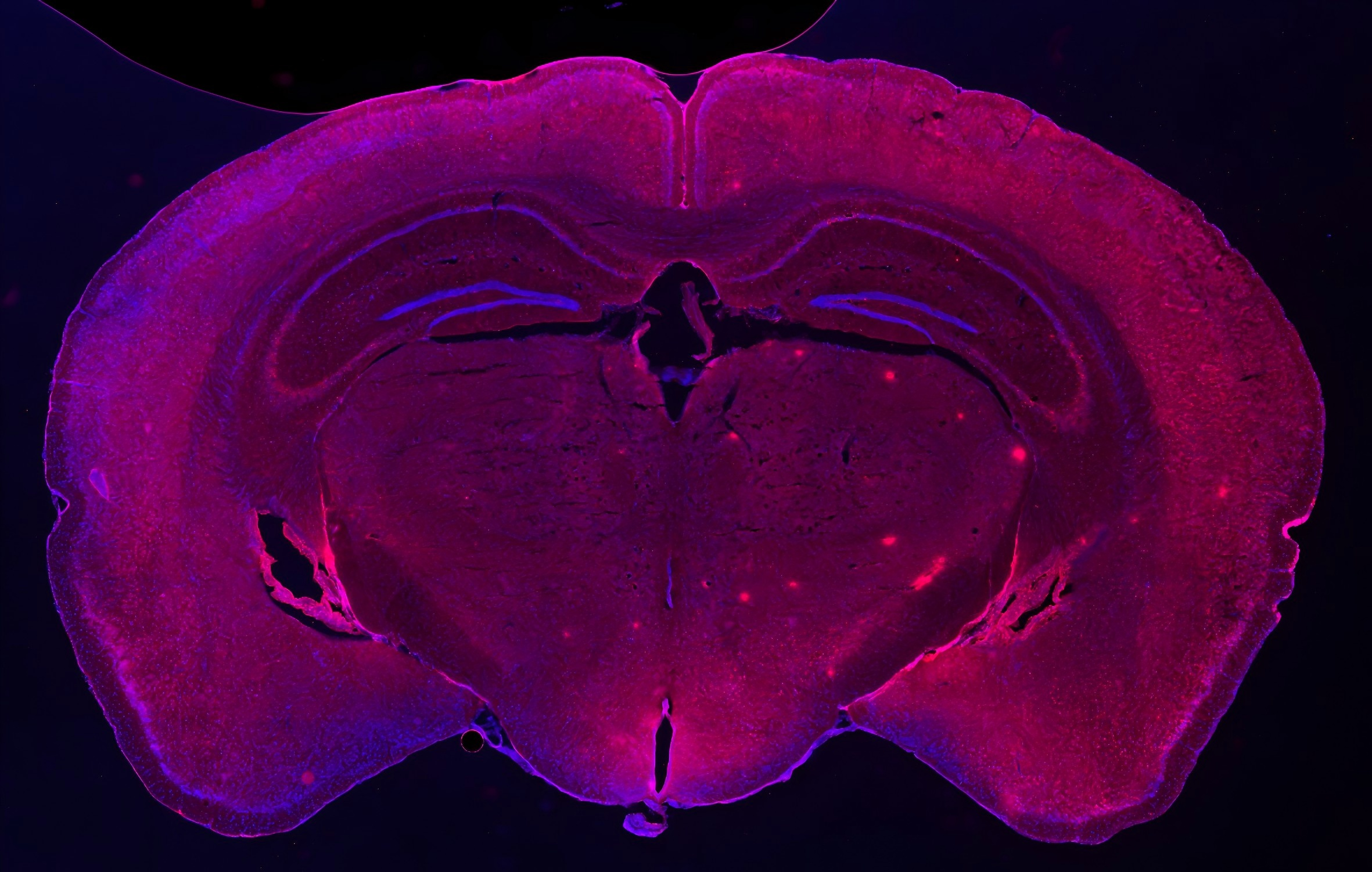

Supplement: Supplementary file 6 — Supporting File 6: advs74789‐sup‐0006‐Data.zip. [file ADVS-13-e22572-s004.zip › WT-m1A.tif]

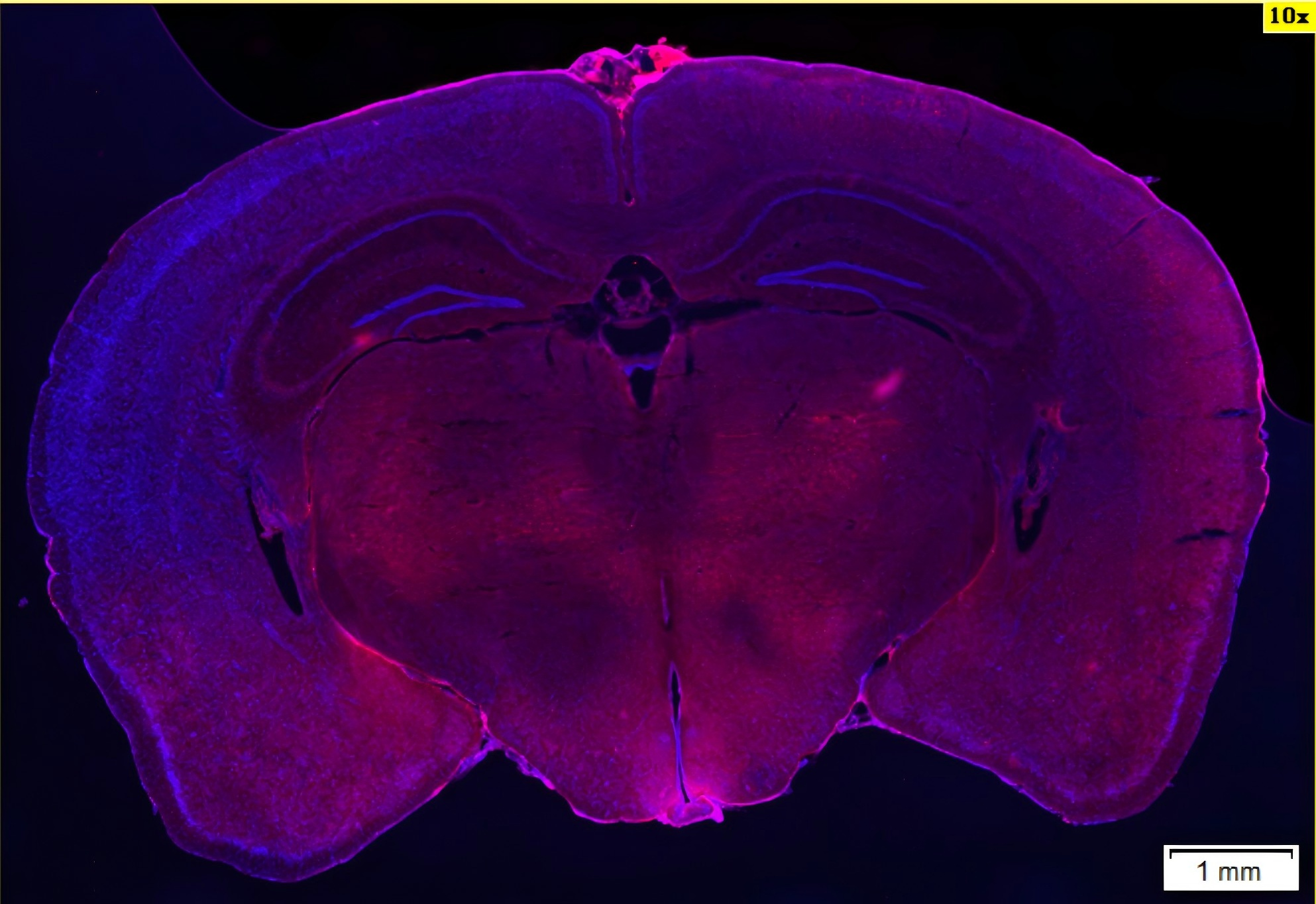

Supplement: Supplementary file 6 — Supporting File 6: advs74789‐sup‐0006‐Data.zip. [file ADVS-13-e22572-s004.zip › FAD-m1A.tif]

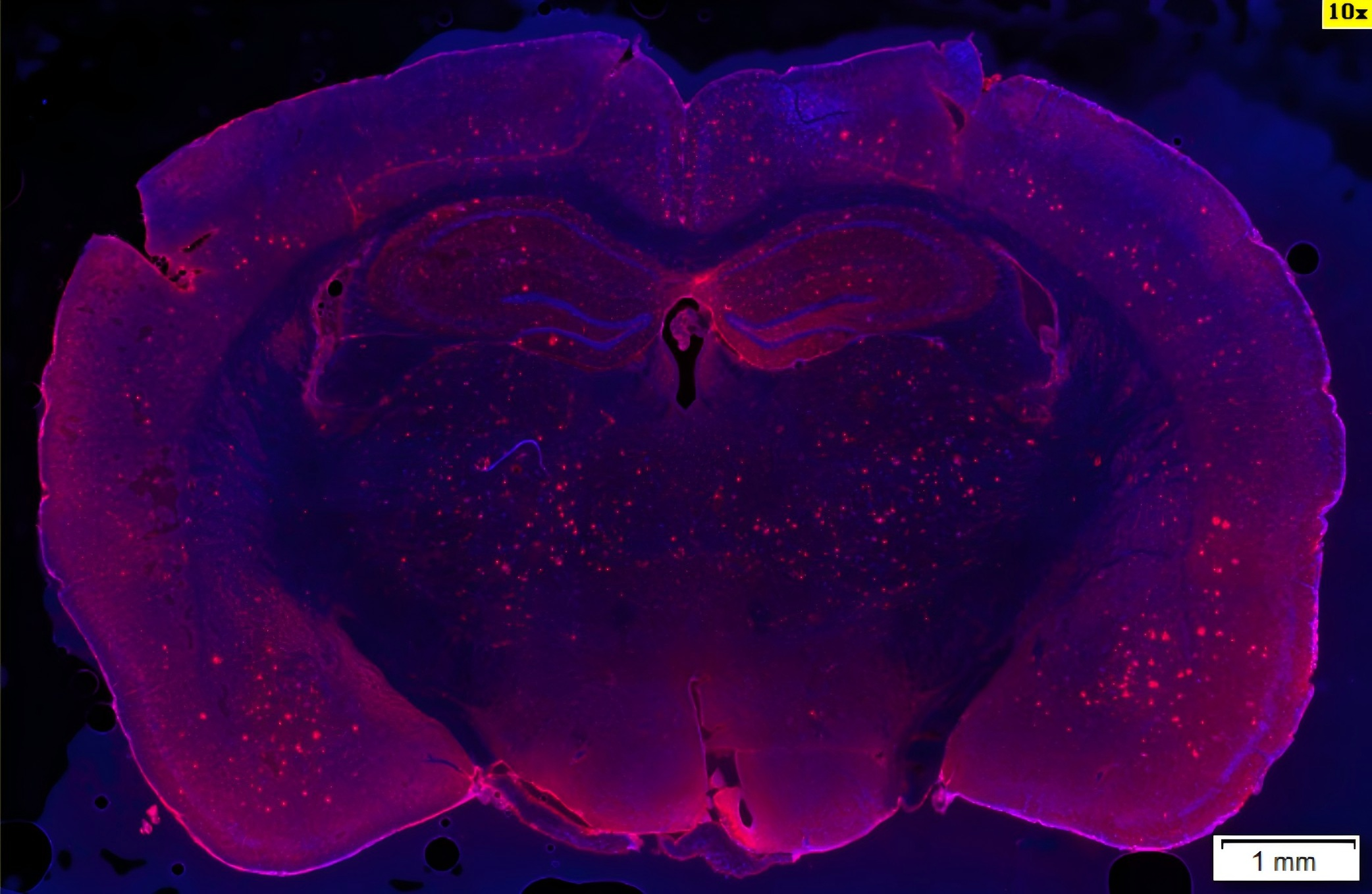

Supplement: Supplementary file 6 — Supporting File 6: advs74789‐sup‐0006‐Data.zip. [file ADVS-13-e22572-s004.zip › renamed_da11c.tif]

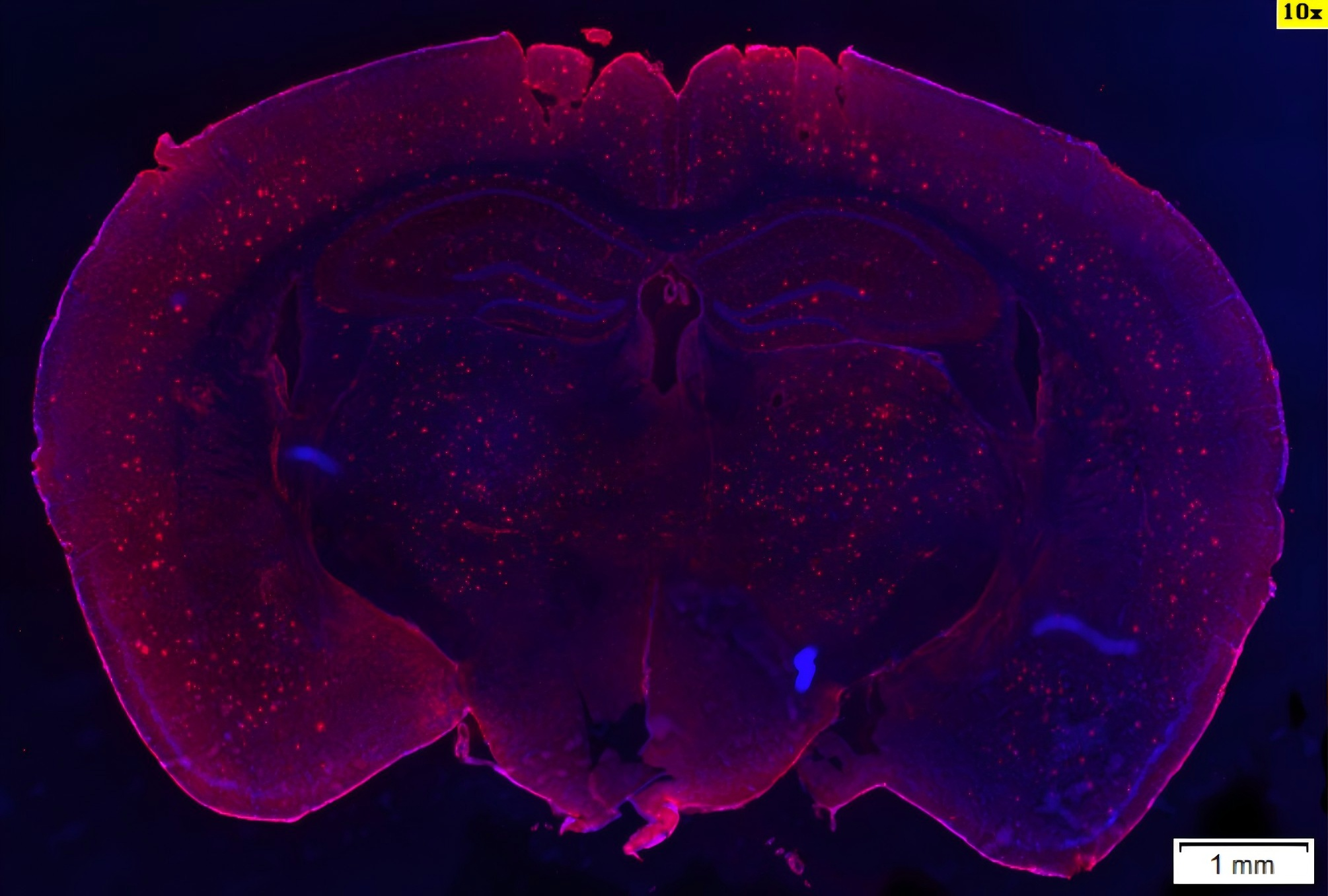

Supplement: Supplementary file 6 — Supporting File 6: advs74789‐sup‐0006‐Data.zip. [file ADVS-13-e22572-s004.zip › renamed_45b32.tif]

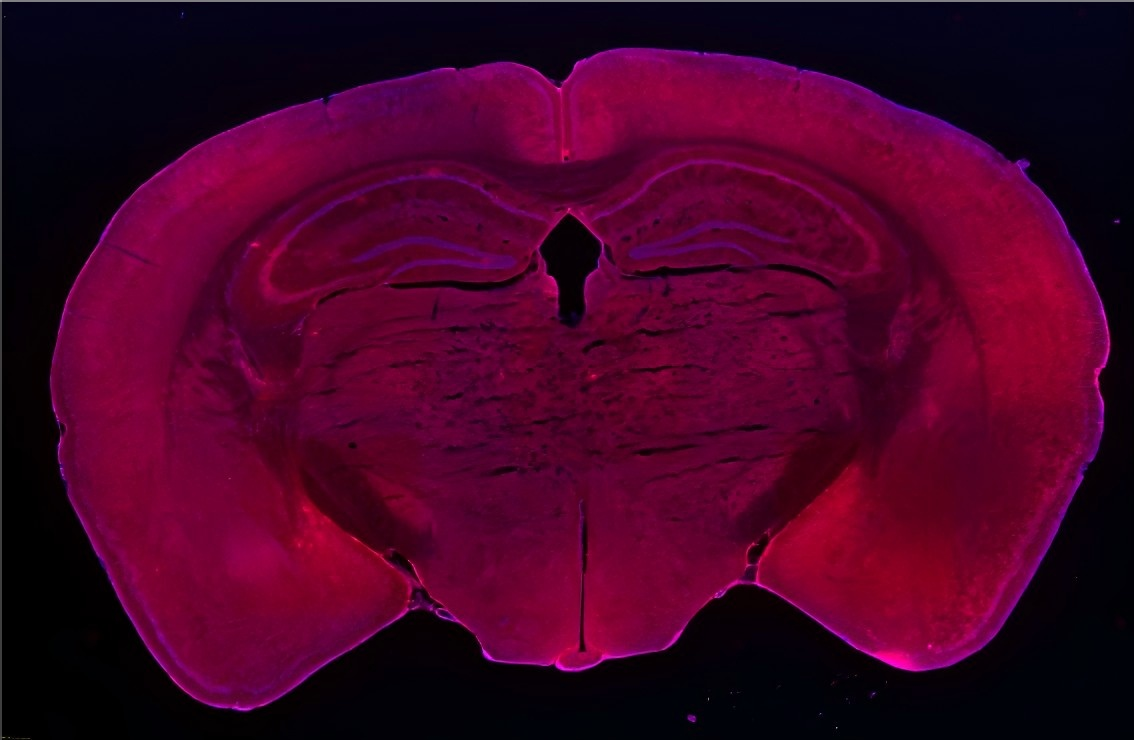

Supplement: Supplementary file 6 — Supporting File 6: advs74789‐sup‐0006‐Data.zip. [file ADVS-13-e22572-s004.zip › fad_m1A.tif]

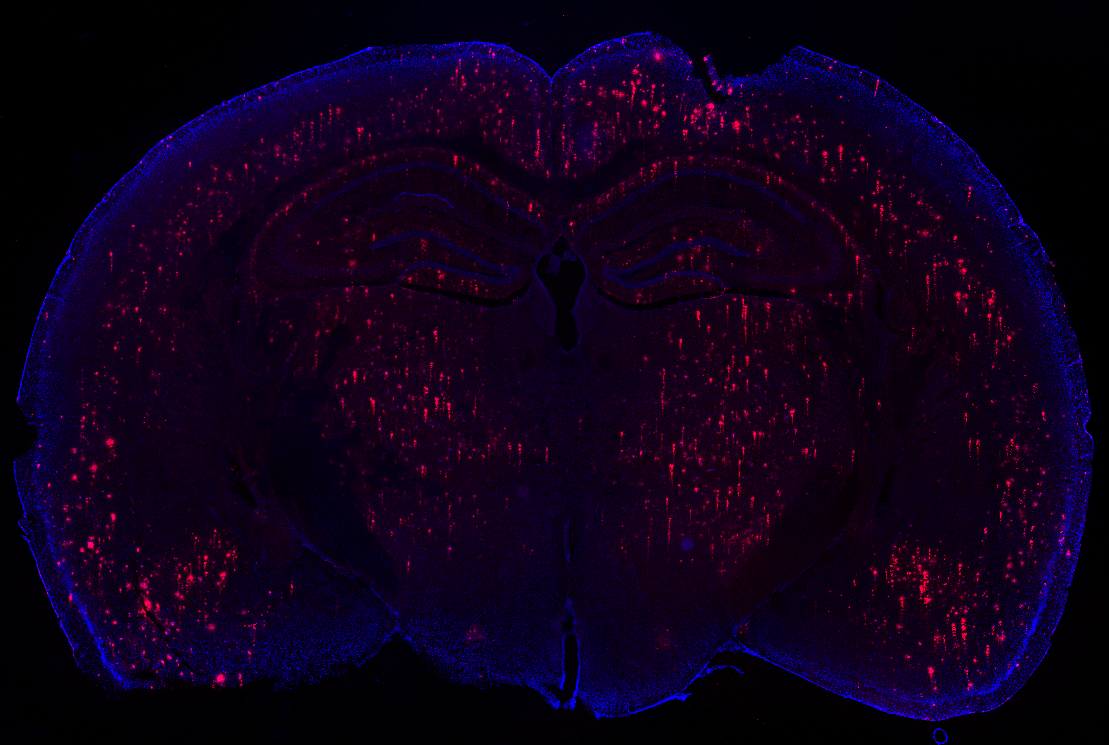

Supplement: Supplementary file 6 — Supporting File 6: advs74789‐sup‐0006‐Data.zip. [file ADVS-13-e22572-s004.zip › renamed_93572.tif]

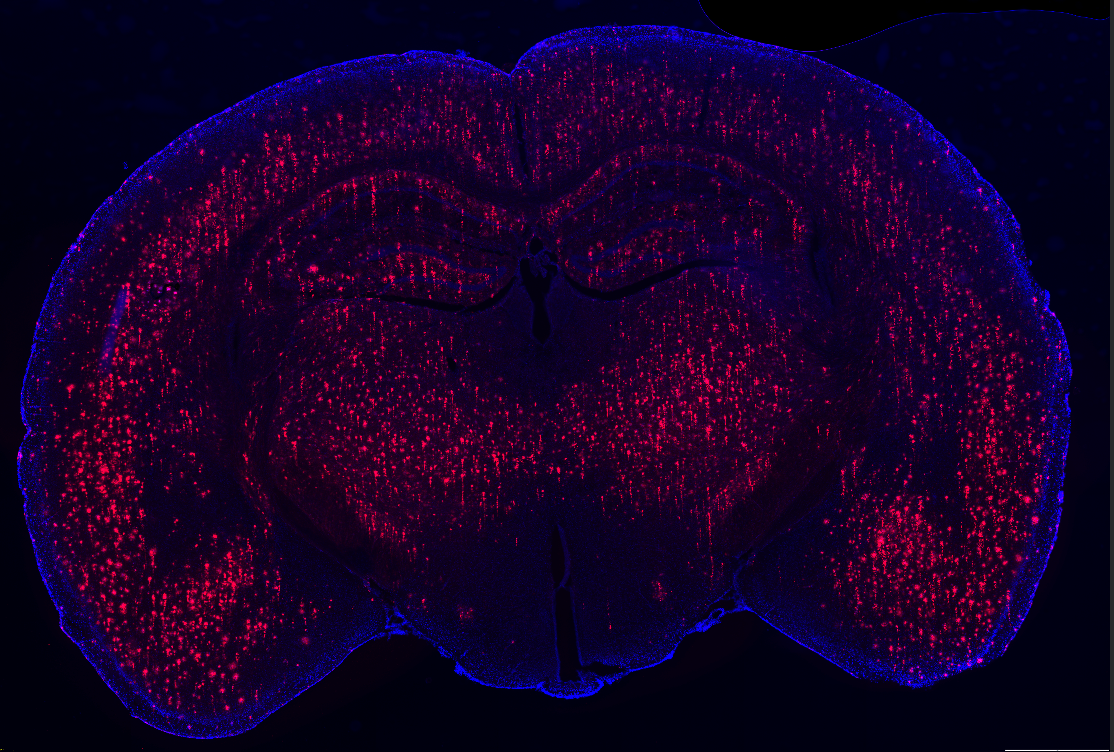

Supplement: Supplementary file 6 — Supporting File 6: advs74789‐sup‐0006‐Data.zip. [file ADVS-13-e22572-s004.zip › renamed_c01b3.tif]

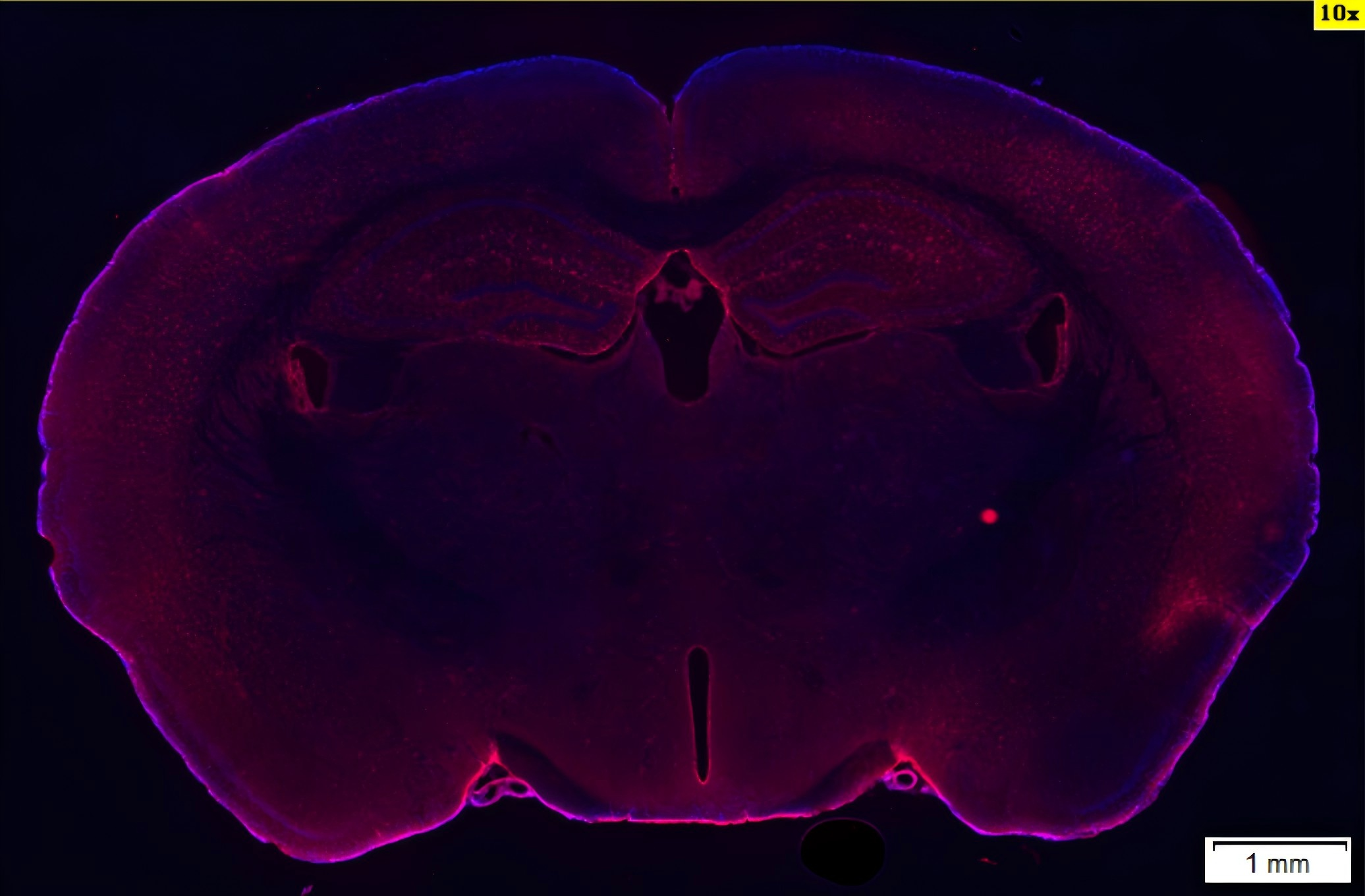

Supplement: Supplementary file 6 — Supporting File 6: advs74789‐sup‐0006‐Data.zip. [file ADVS-13-e22572-s004.zip › AAV-Alk-Psd95.tif]

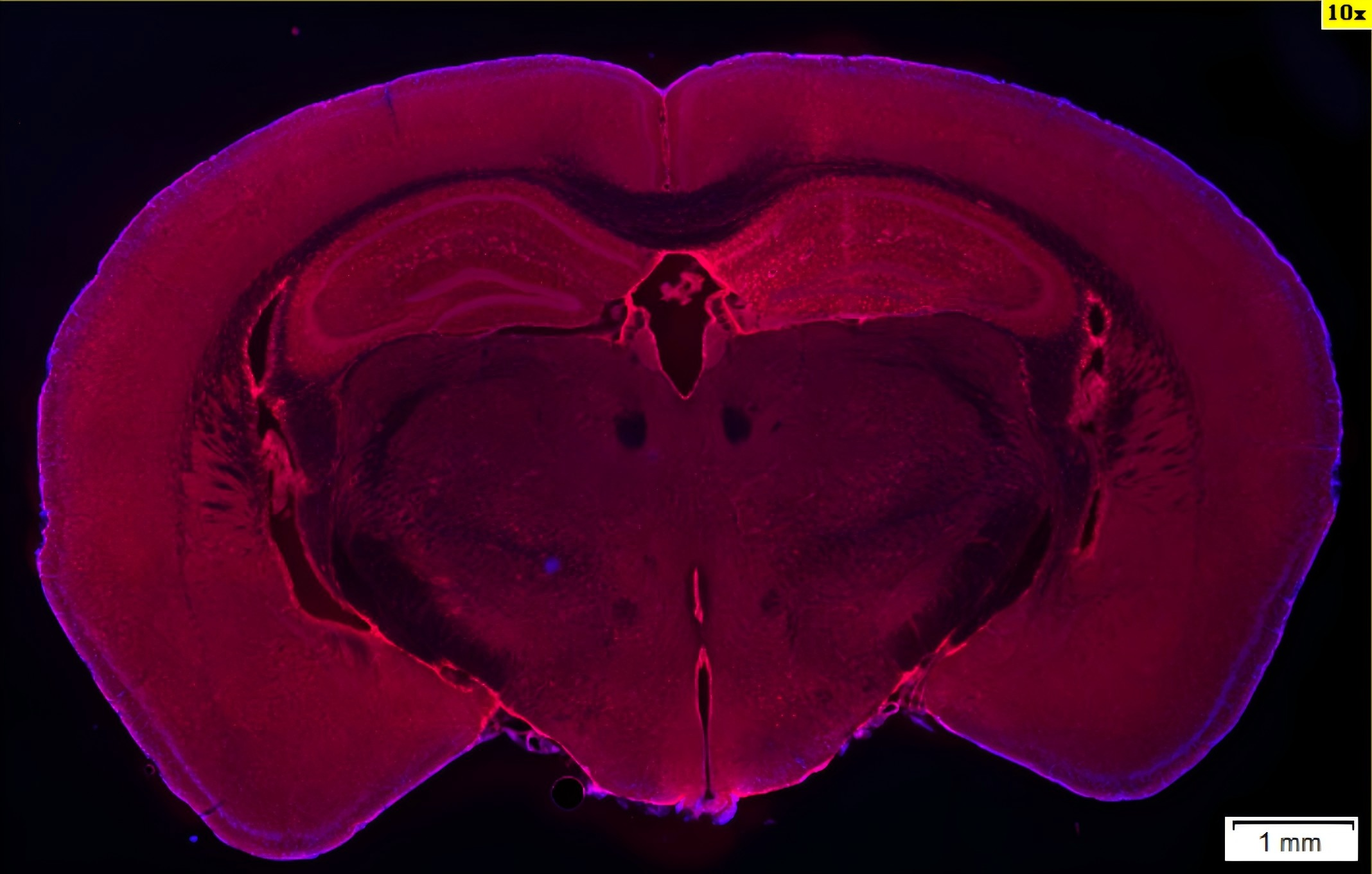

Supplement: Supplementary file 6 — Supporting File 6: advs74789‐sup‐0006‐Data.zip. [file ADVS-13-e22572-s004.zip › AAV-con-Psd95.tif]

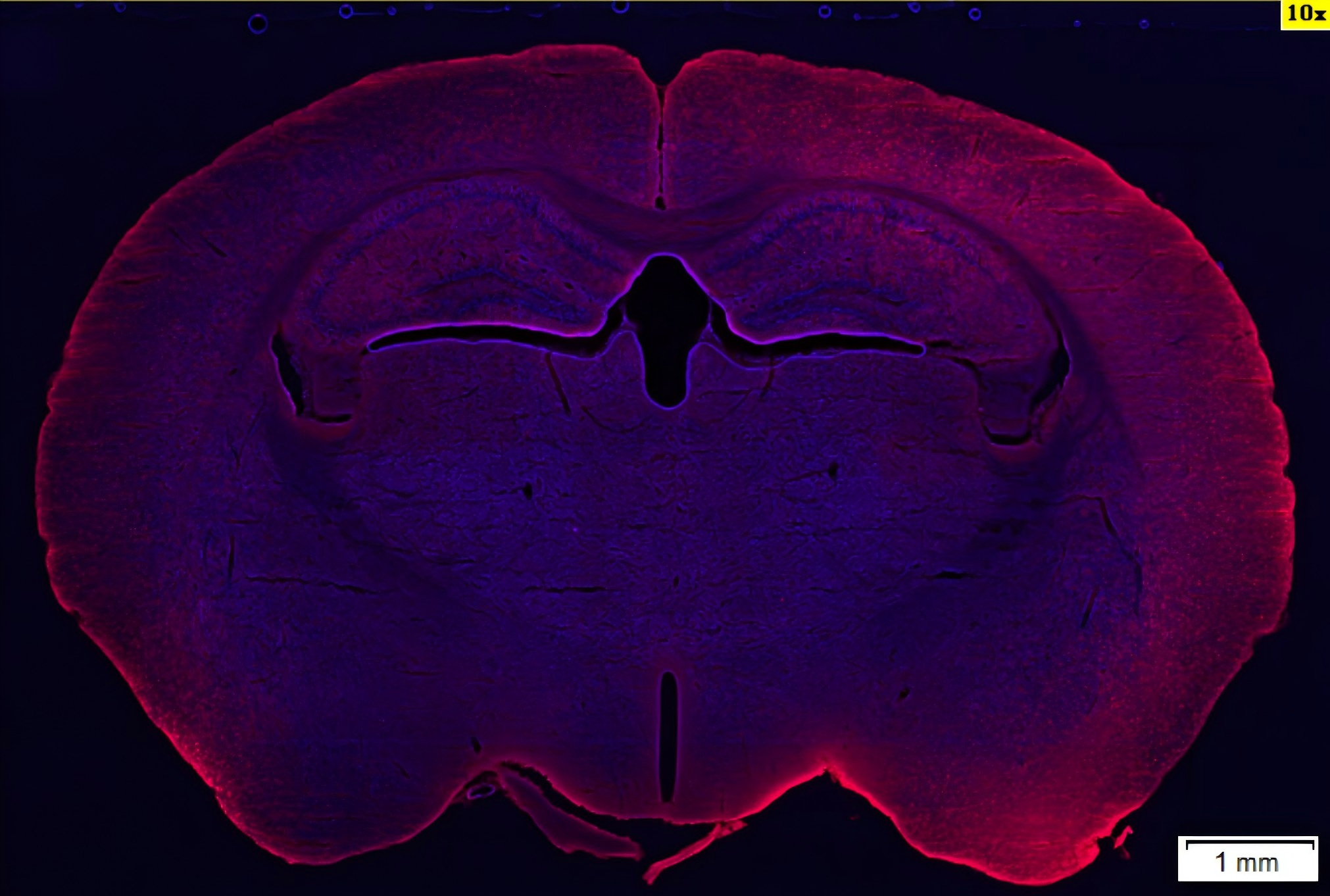

Supplement: Supplementary file 6 — Supporting File 6: advs74789‐sup‐0006‐Data.zip. [file ADVS-13-e22572-s004.zip › Alk-KO_PINK1.tif]

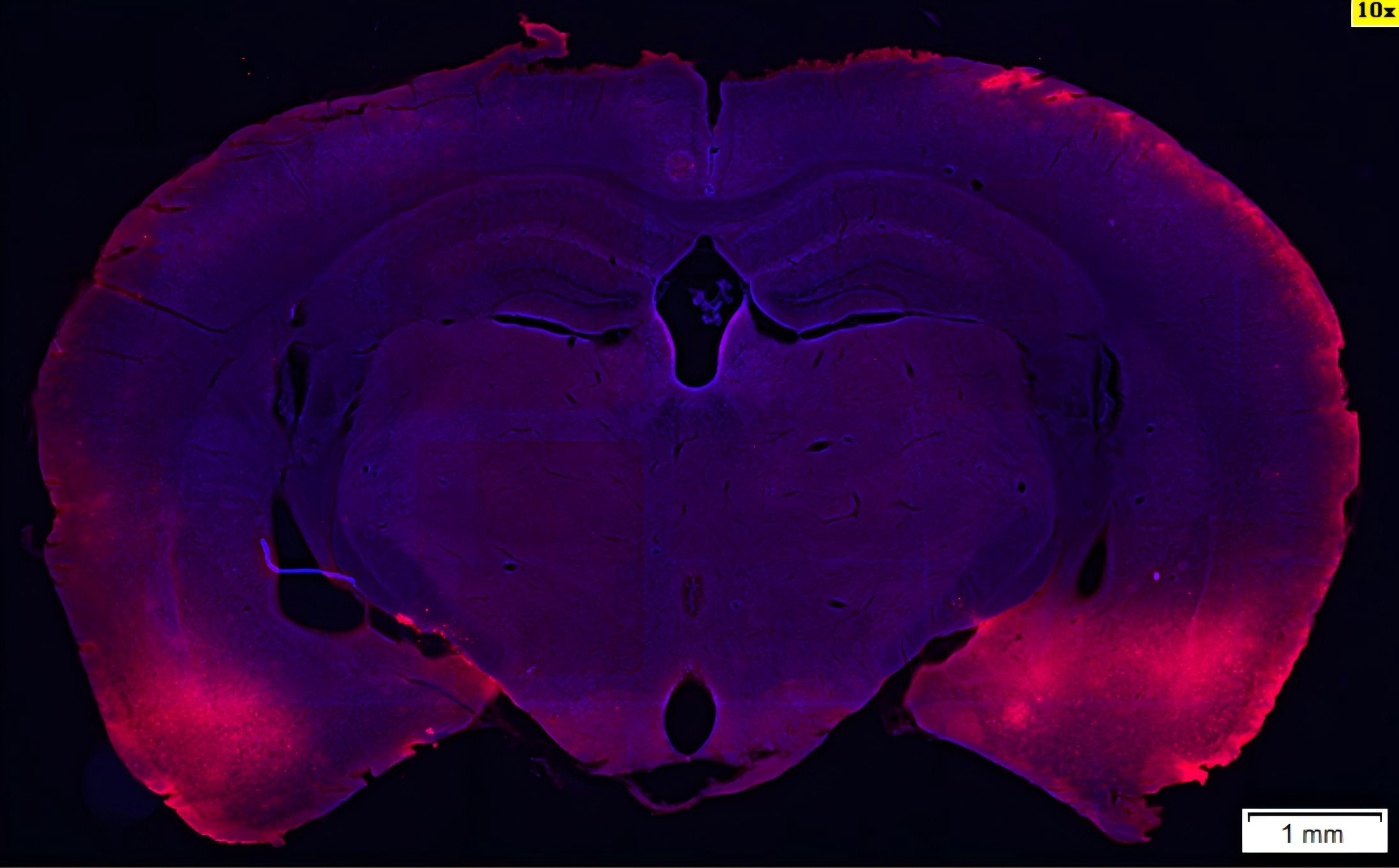

Supplement: Supplementary file 6 — Supporting File 6: advs74789‐sup‐0006‐Data.zip. [file ADVS-13-e22572-s004.zip › WT_PINK1.tif]

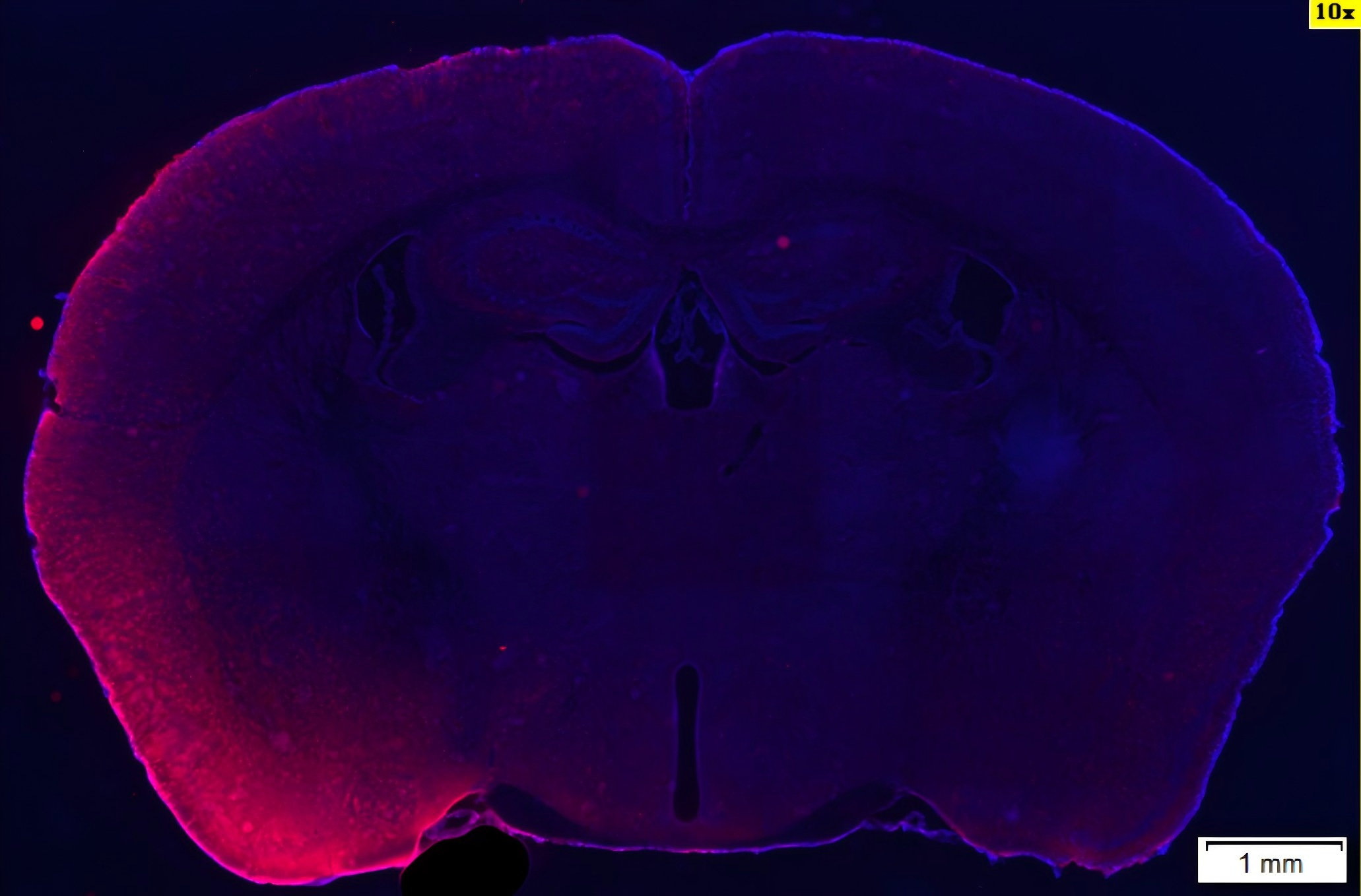

Supplement: Supplementary file 6 — Supporting File 6: advs74789‐sup‐0006‐Data.zip. [file ADVS-13-e22572-s004.zip › AAV-alk_PINK1.tif]

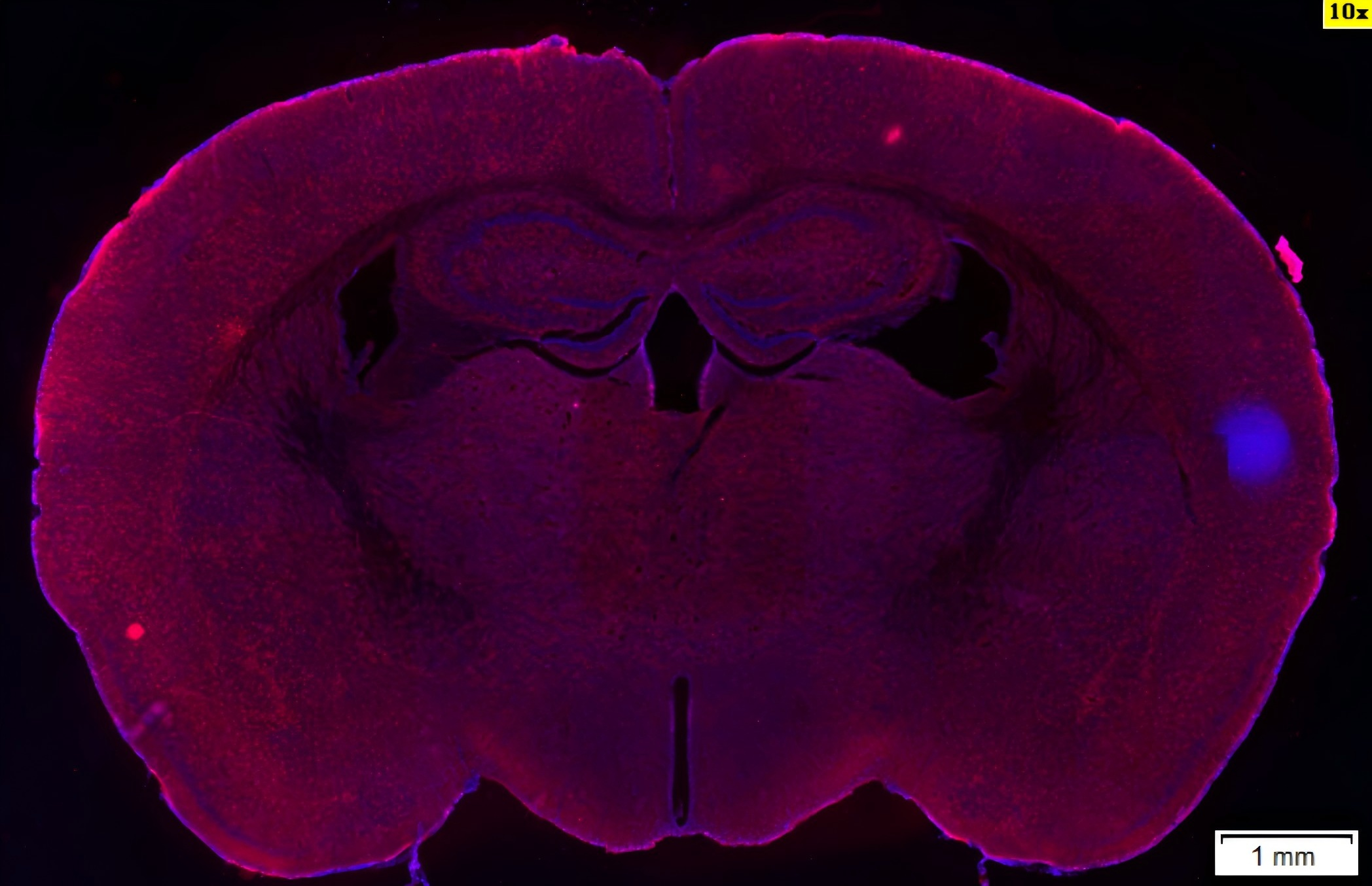

Supplement: Supplementary file 6 — Supporting File 6: advs74789‐sup‐0006‐Data.zip. [file ADVS-13-e22572-s004.zip › AAV-con_PINK1.tif]

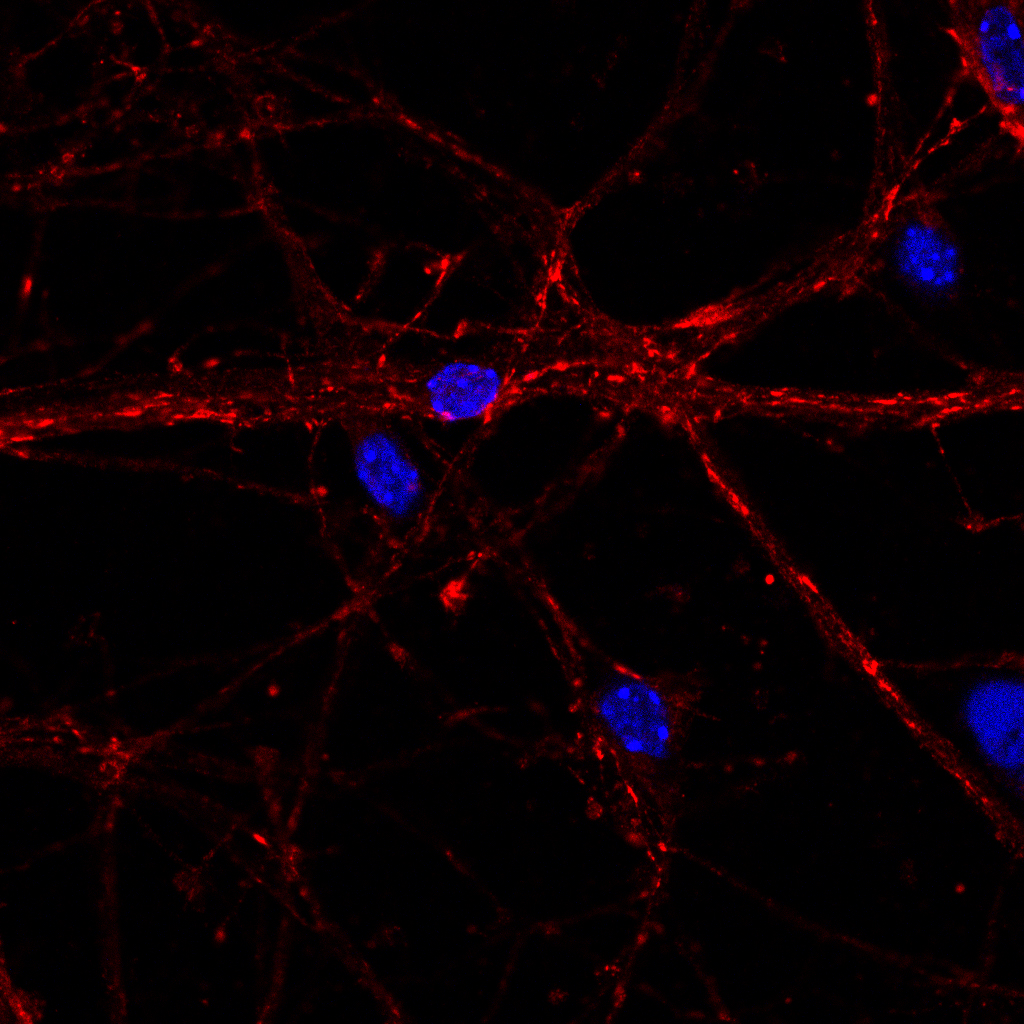

Supplement: Supplementary file 6 — Supporting File 6: advs74789‐sup‐0006‐Data.zip. [file ADVS-13-e22572-s004.zip › shcon_SYP_merge.tif]

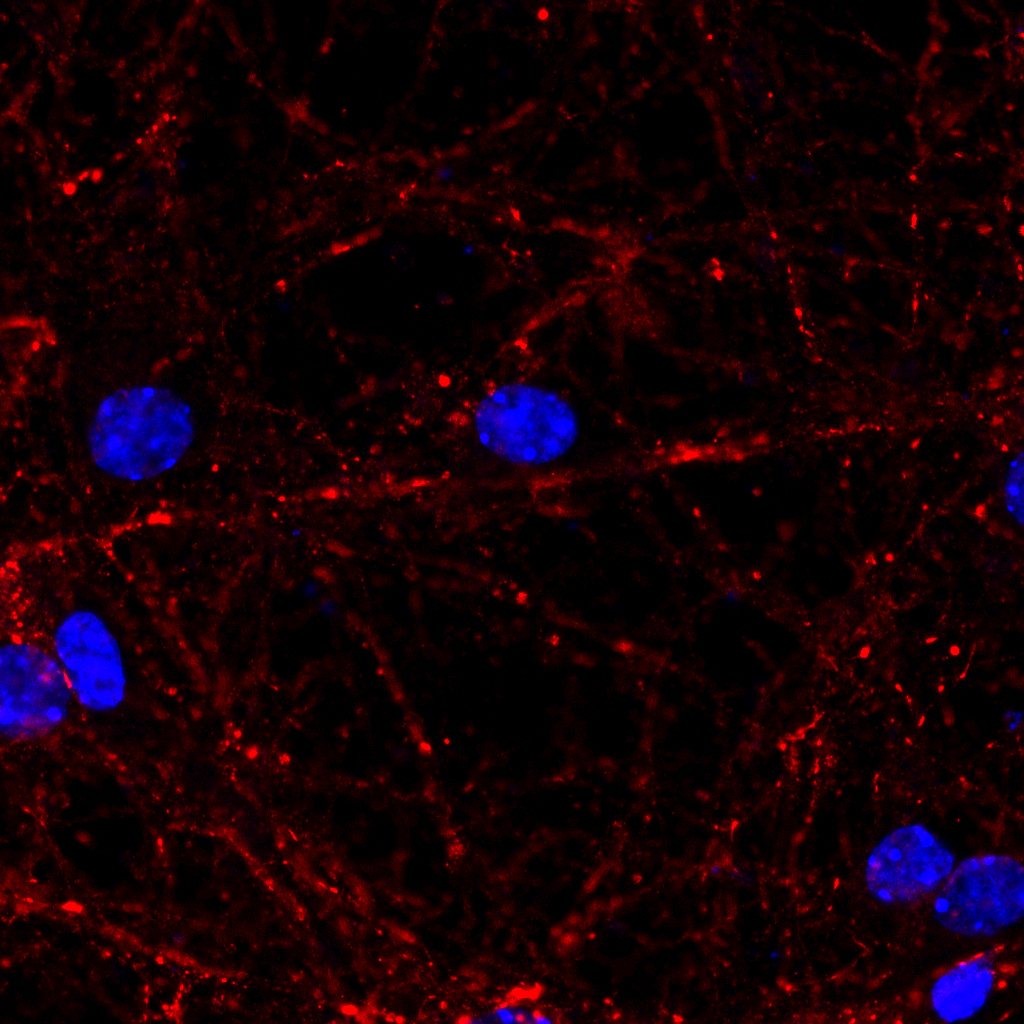

Supplement: Supplementary file 6 — Supporting File 6: advs74789‐sup‐0006‐Data.zip. [file ADVS-13-e22572-s004.zip › Vec_SYP_merge.tif]

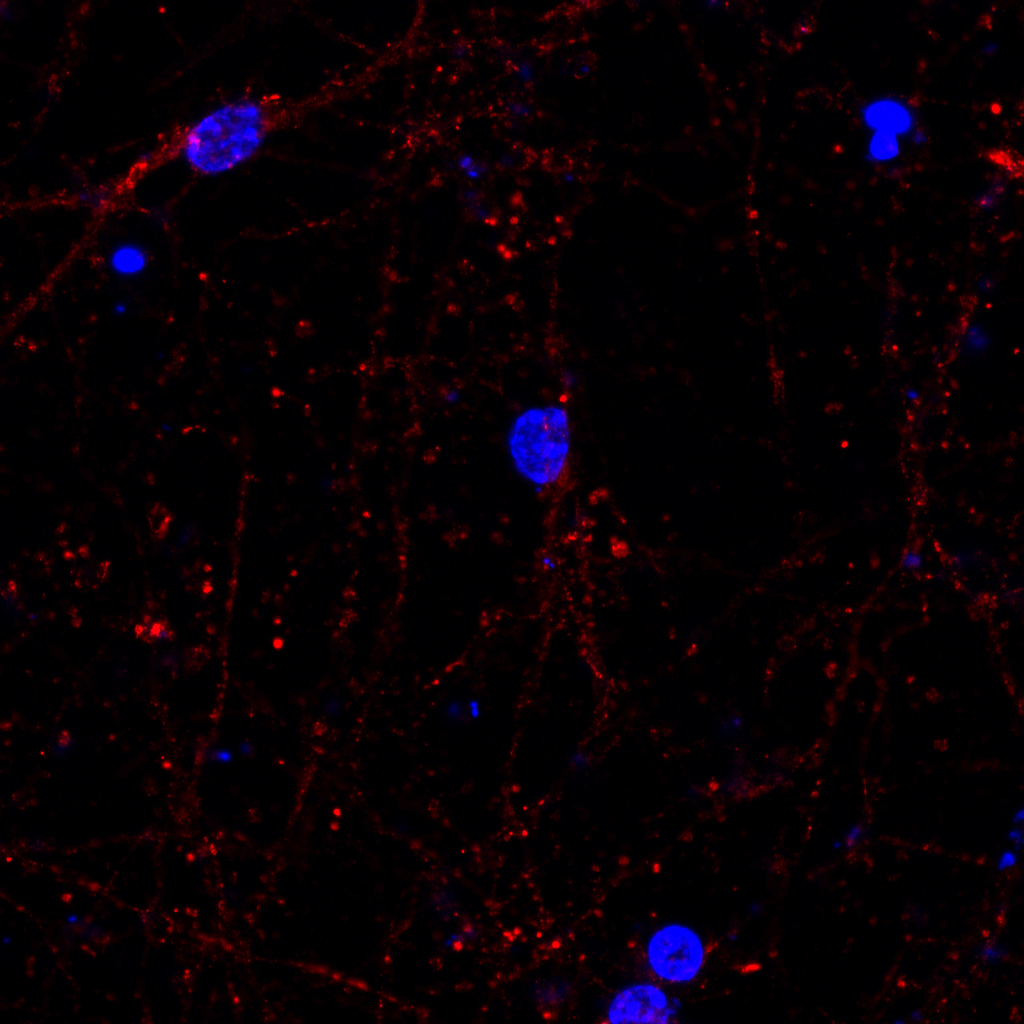

Supplement: Supplementary file 6 — Supporting File 6: advs74789‐sup‐0006‐Data.zip. [file ADVS-13-e22572-s004.zip › ALK_SYP_merge.tif]

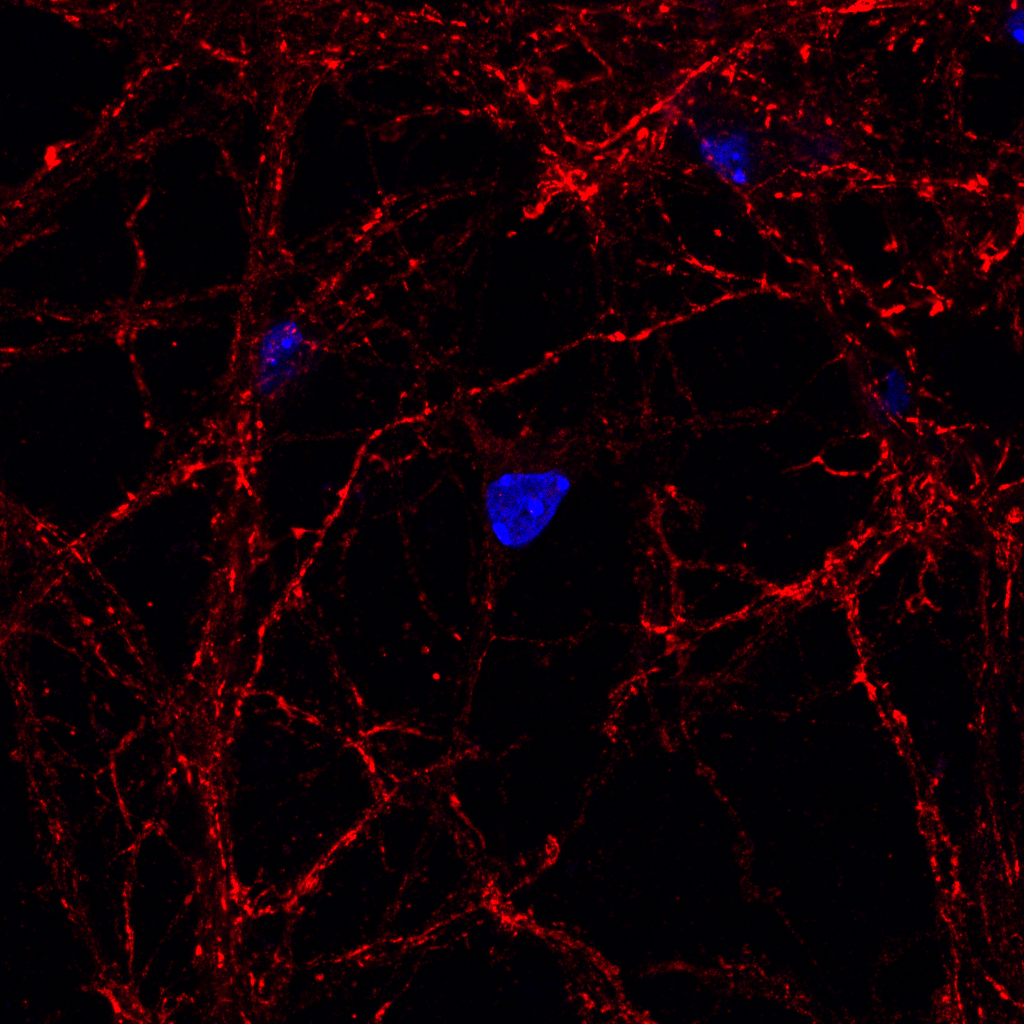

Supplement: Supplementary file 6 — Supporting File 6: advs74789‐sup‐0006‐Data.zip. [file ADVS-13-e22572-s004.zip › shALK_SYP_merge.tif]

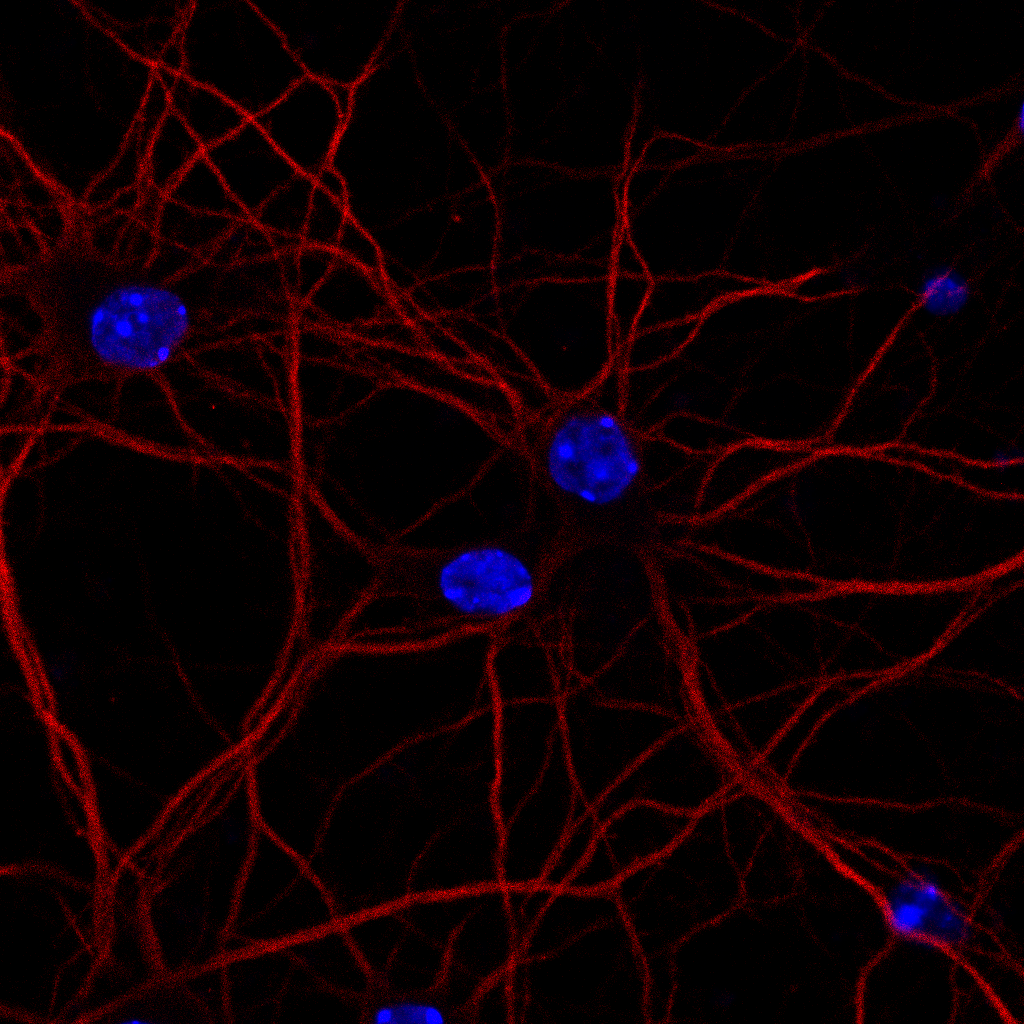

Supplement: Supplementary file 6 — Supporting File 6: advs74789‐sup‐0006‐Data.zip. [file ADVS-13-e22572-s004.zip › Vector_Map2_merge.tif]

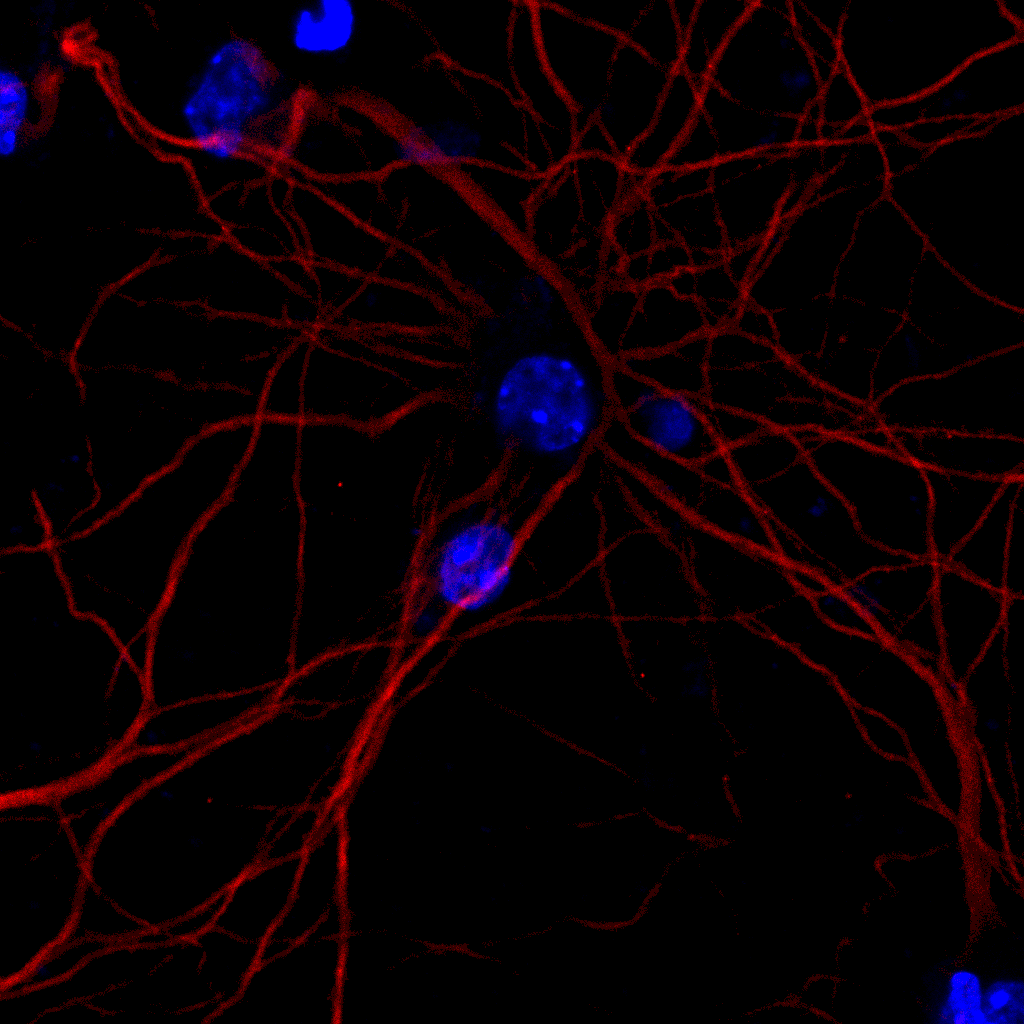

Supplement: Supplementary file 6 — Supporting File 6: advs74789‐sup‐0006‐Data.zip. [file ADVS-13-e22572-s004.zip › shcon_Map2_merge.tif]

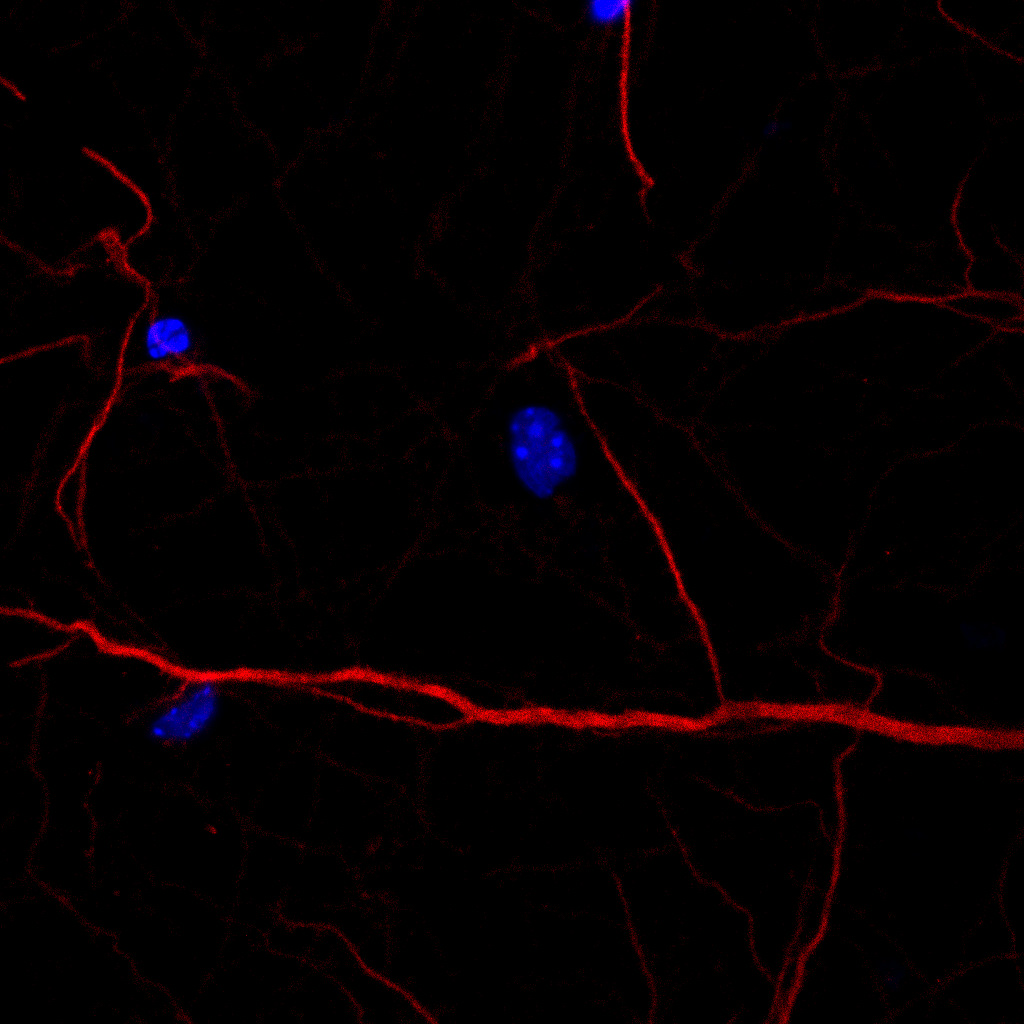

Supplement: Supplementary file 6 — Supporting File 6: advs74789‐sup‐0006‐Data.zip. [file ADVS-13-e22572-s004.zip › ALKBH3_Map2_merge.tif]

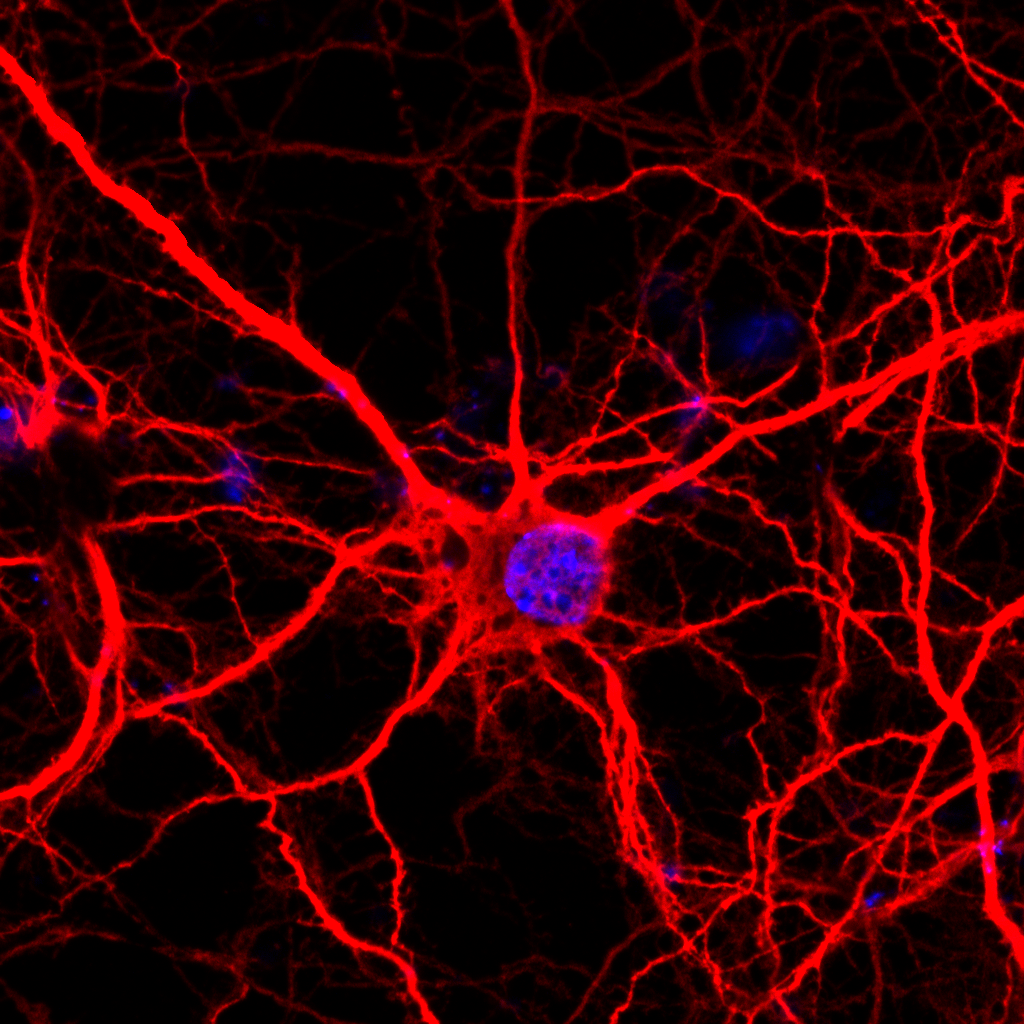

Supplement: Supplementary file 6 — Supporting File 6: advs74789‐sup‐0006‐Data.zip. [file ADVS-13-e22572-s004.zip › shALK_Map2_merge.tif]

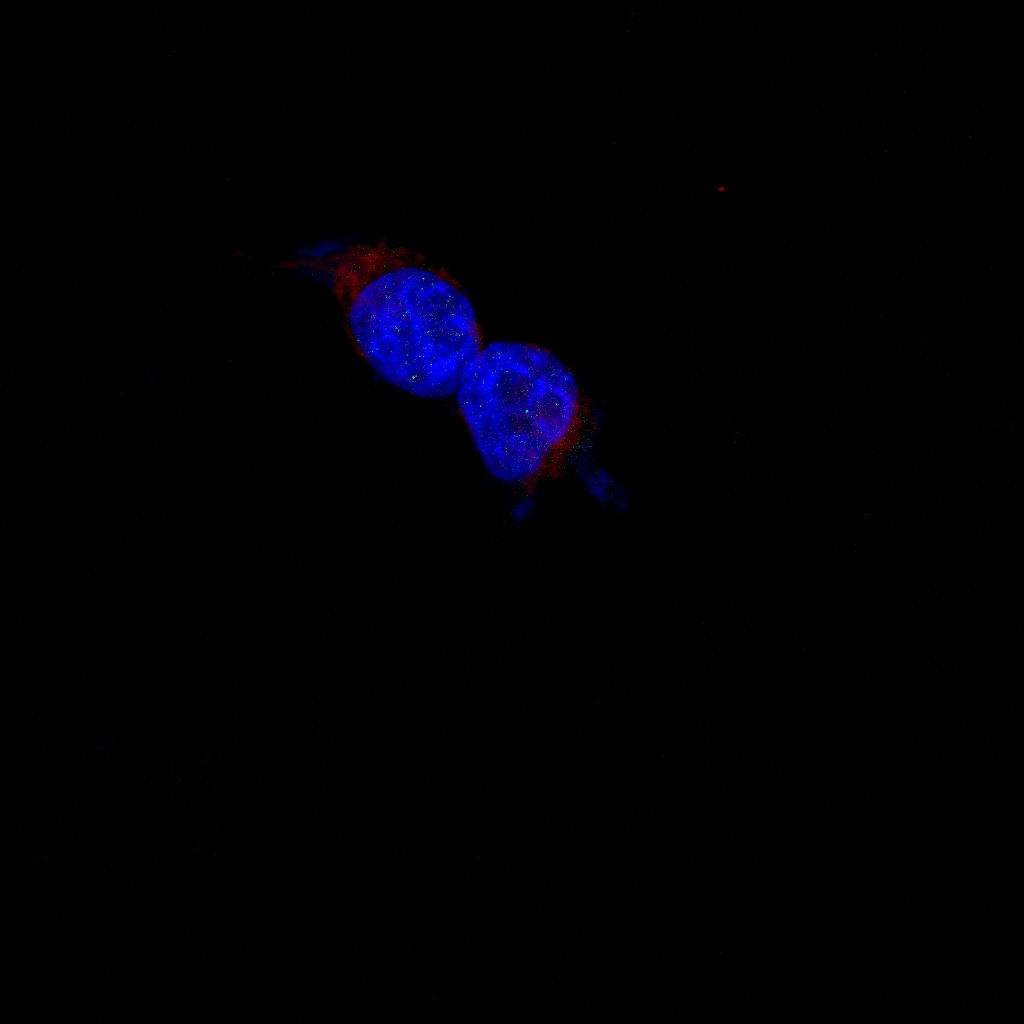

Supplement: Supplementary file 6 — Supporting File 6: advs74789‐sup‐0006‐Data.zip. [file ADVS-13-e22572-s004.zip › ALK_PINK1 no CCCP.tif]

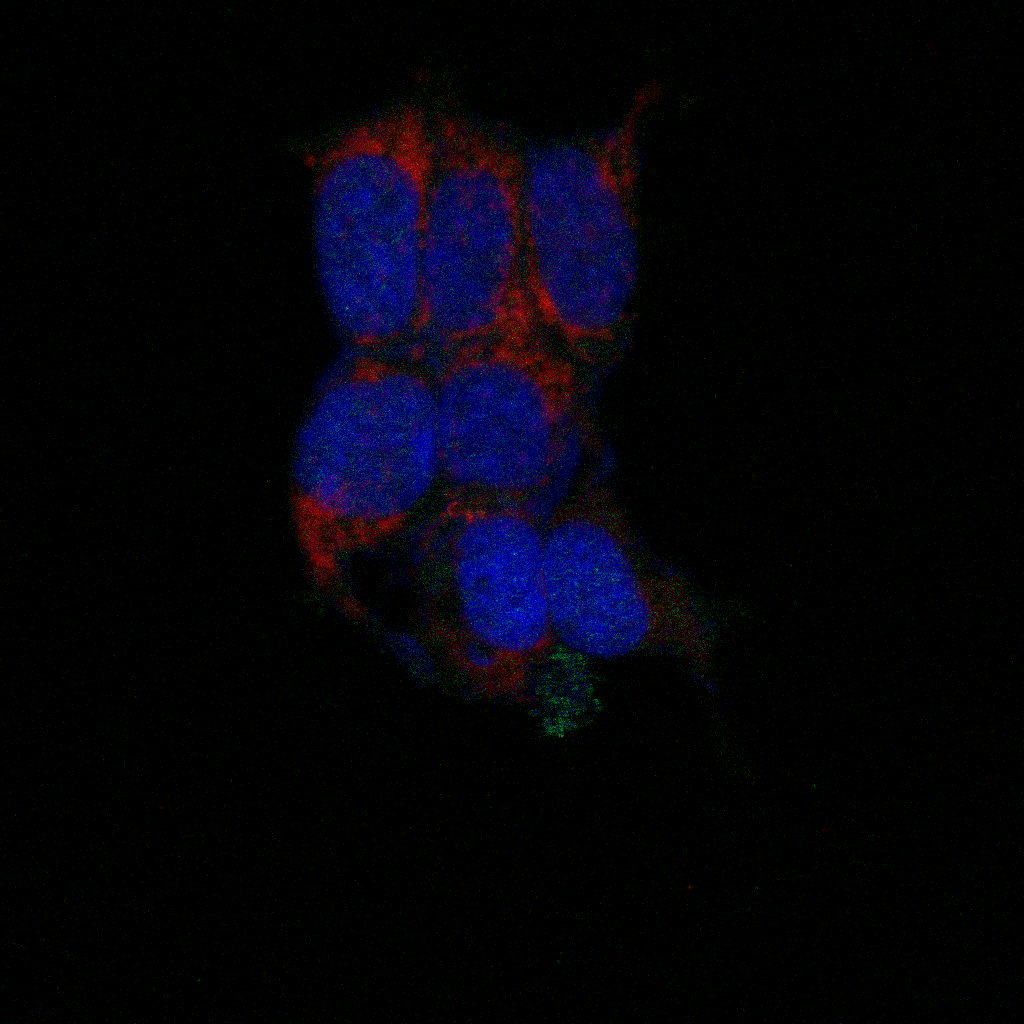

Supplement: Supplementary file 6 — Supporting File 6: advs74789‐sup‐0006‐Data.zip. [file ADVS-13-e22572-s004.zip › ALK_vec no CCCP.tif]

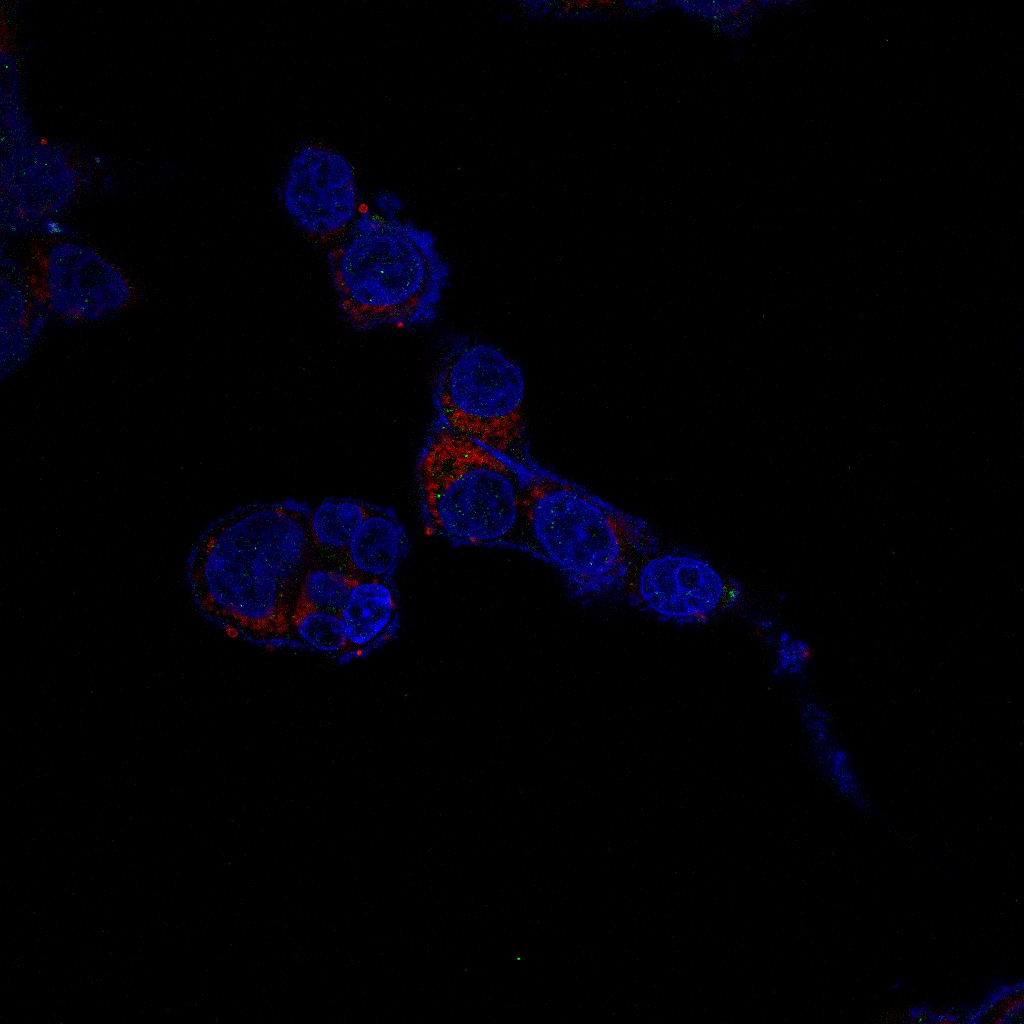

Supplement: Supplementary file 6 — Supporting File 6: advs74789‐sup‐0006‐Data.zip. [file ADVS-13-e22572-s004.zip › ALK_vec CCCP.tif]

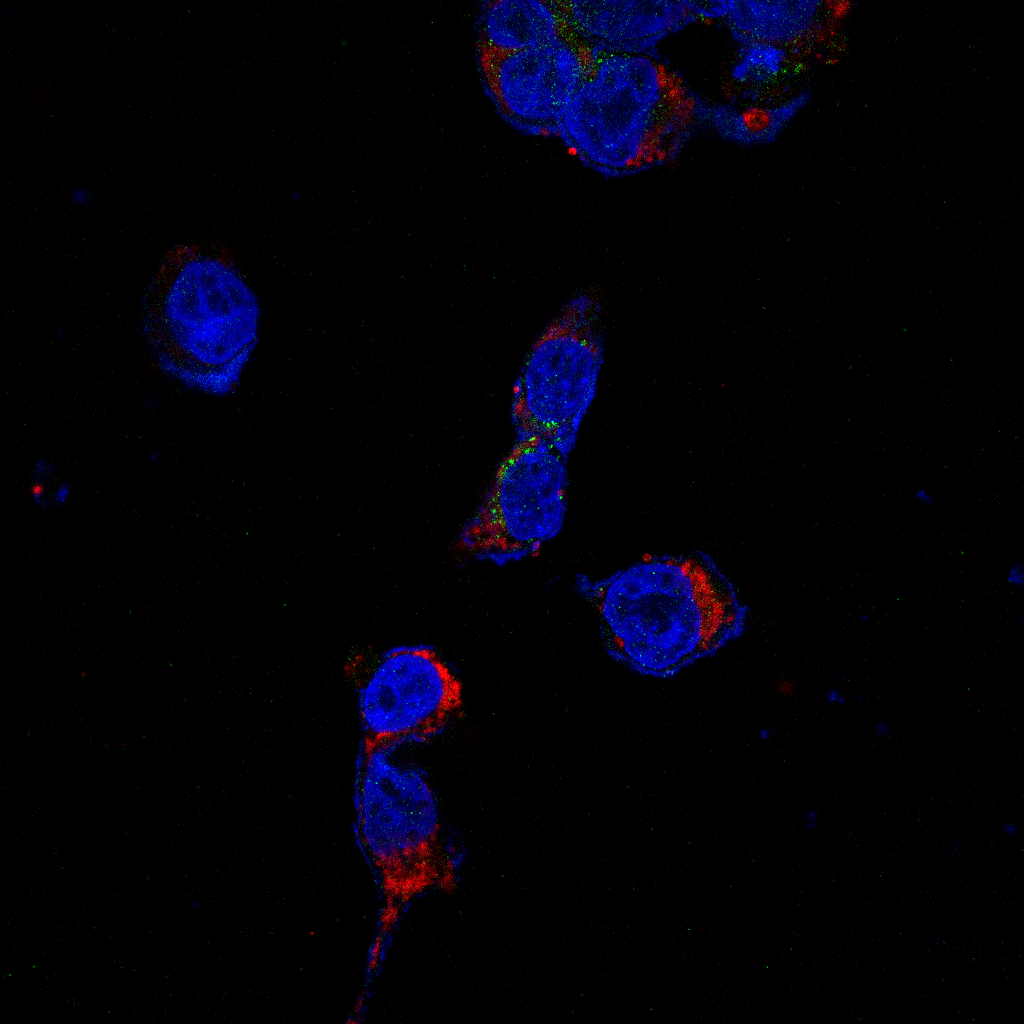

Supplement: Supplementary file 6 — Supporting File 6: advs74789‐sup‐0006‐Data.zip. [file ADVS-13-e22572-s004.zip › ALK_PINK1 CCCP.tif]

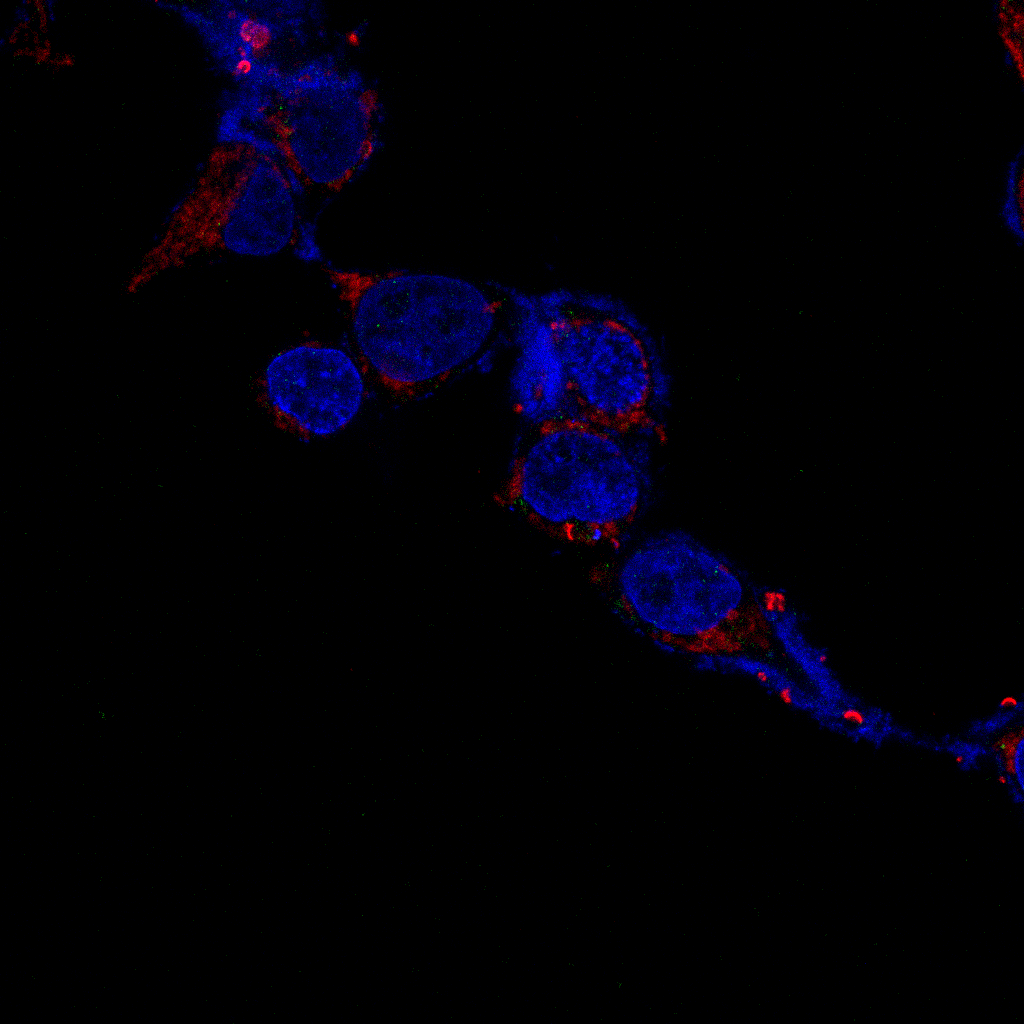

Supplement: Supplementary file 6 — Supporting File 6: advs74789‐sup‐0006‐Data.zip. [file ADVS-13-e22572-s004.zip › shALK_WT no CCCP.tif]

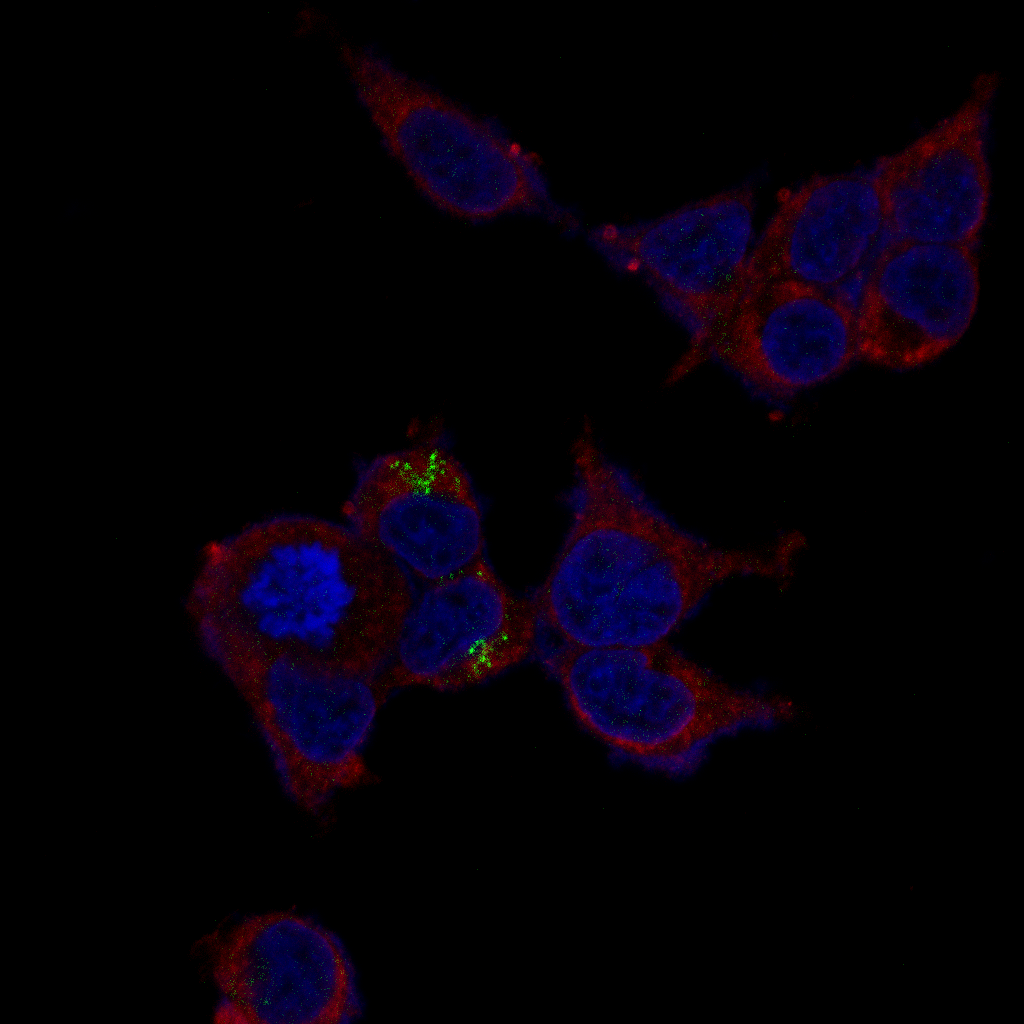

Supplement: Supplementary file 6 — Supporting File 6: advs74789‐sup‐0006‐Data.zip. [file ADVS-13-e22572-s004.zip › shALK_MT CCCP.tif]

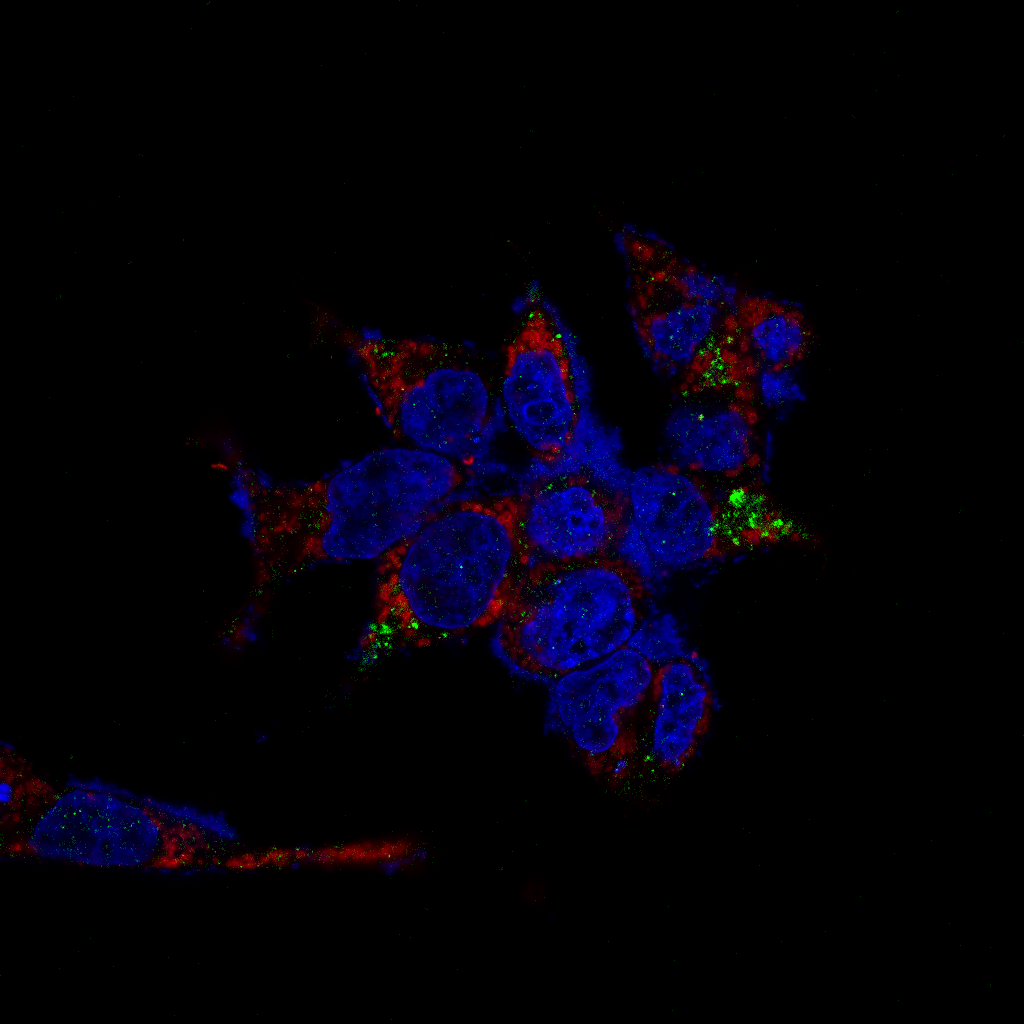

Supplement: Supplementary file 6 — Supporting File 6: advs74789‐sup‐0006‐Data.zip. [file ADVS-13-e22572-s004.zip › shALK_WT CCCP.tif]

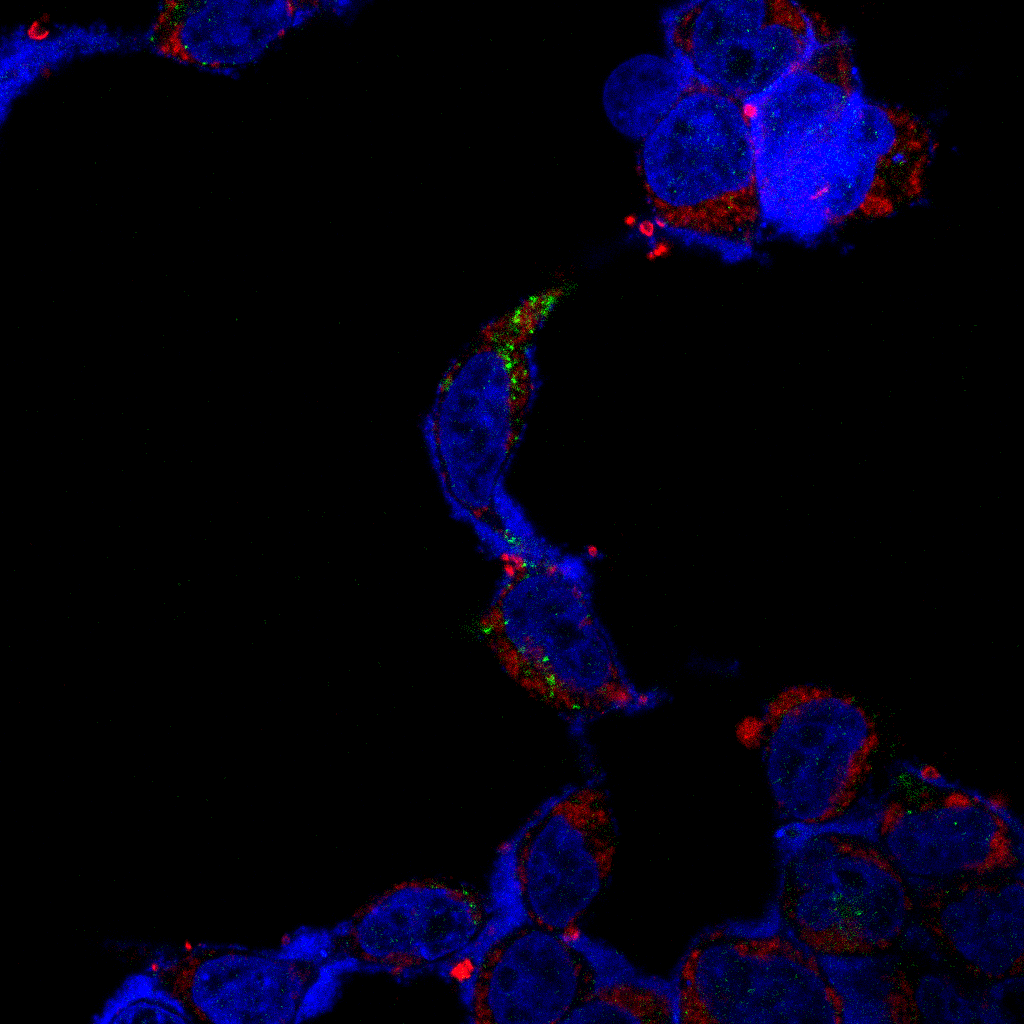

Supplement: Supplementary file 6 — Supporting File 6: advs74789‐sup‐0006‐Data.zip. [file ADVS-13-e22572-s004.zip › shALK_vec CCCP.tif]

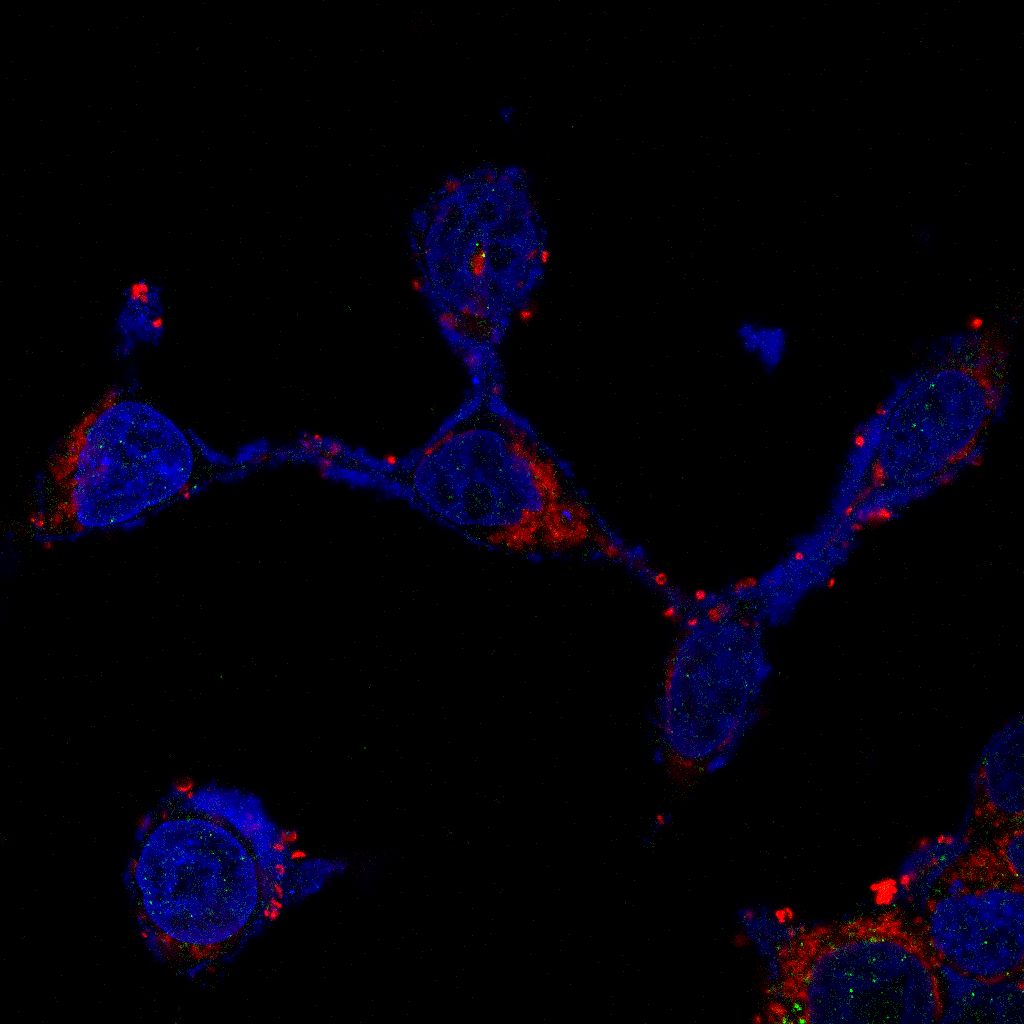

Supplement: Supplementary file 6 — Supporting File 6: advs74789‐sup‐0006‐Data.zip. [file ADVS-13-e22572-s004.zip › shALK_vec no CCCP.tif]

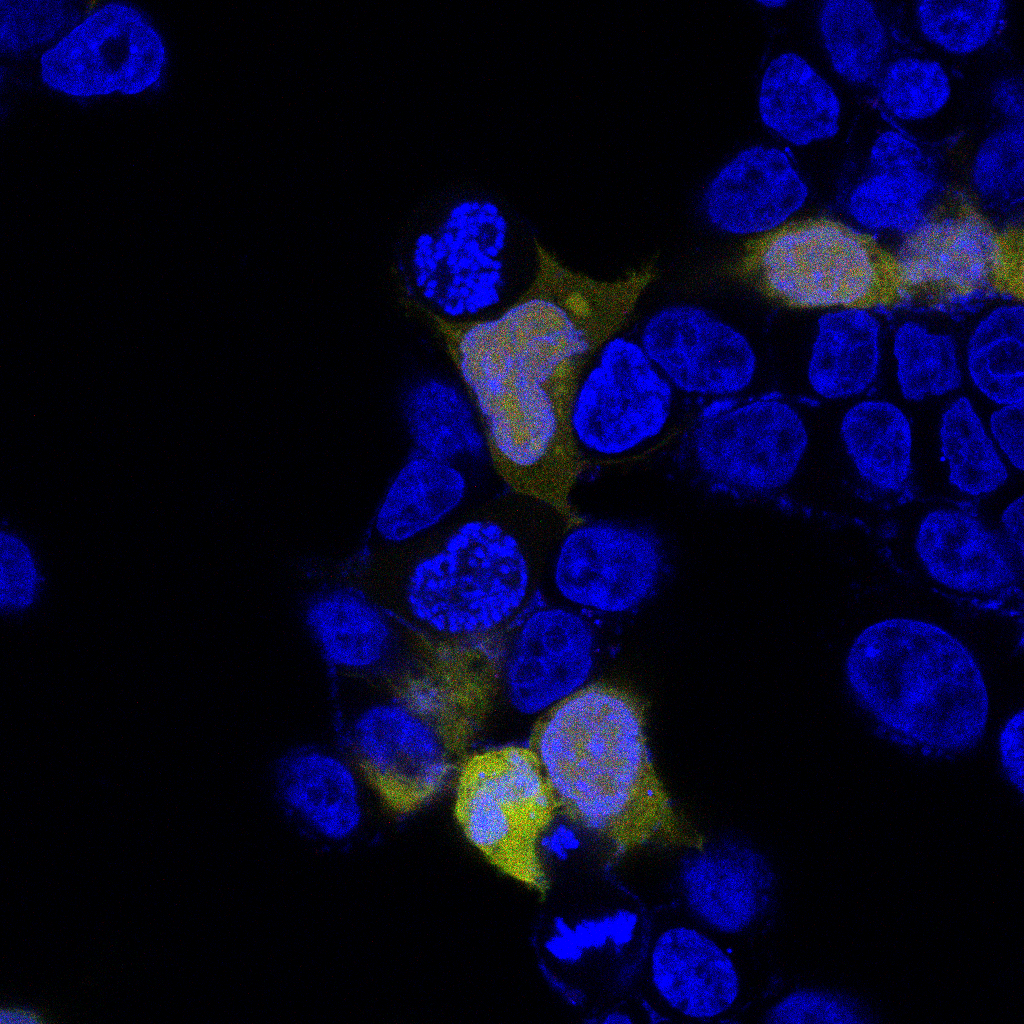

Supplement: Supplementary file 6 — Supporting File 6: advs74789‐sup‐0006‐Data.zip. [file ADVS-13-e22572-s004.zip › shALK no CCCP.tif]

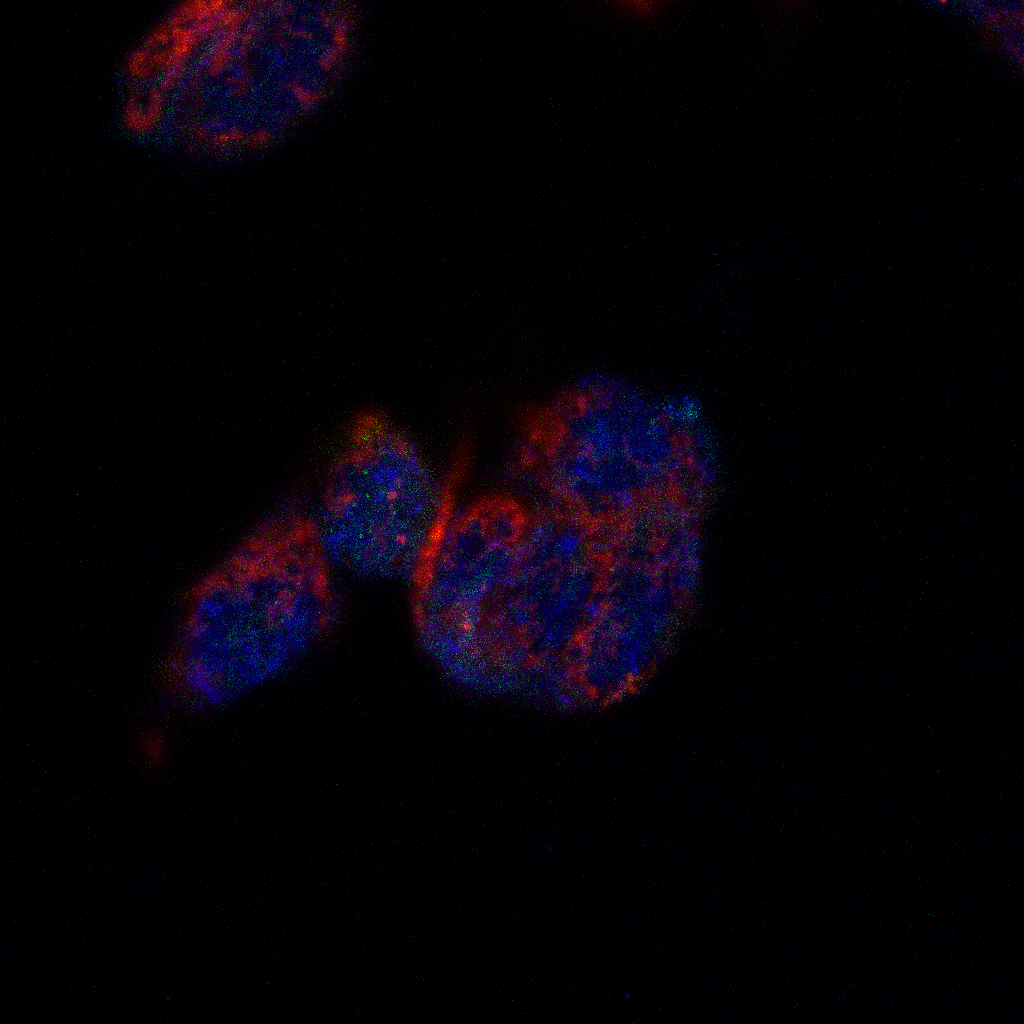

Supplement: Supplementary file 6 — Supporting File 6: advs74789‐sup‐0006‐Data.zip. [file ADVS-13-e22572-s004.zip › shcon no CCCP_293_1.tif]

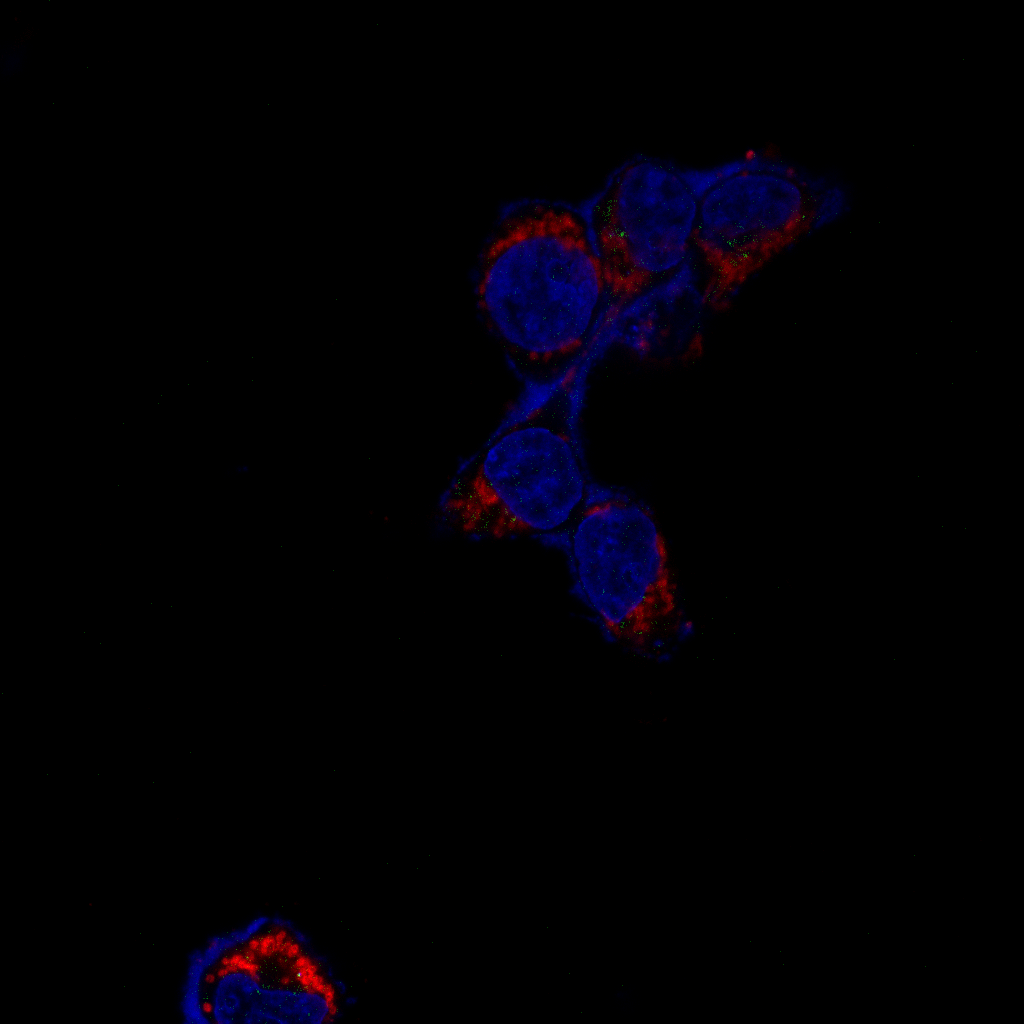

Supplement: Supplementary file 6 — Supporting File 6: advs74789‐sup‐0006‐Data.zip. [file ADVS-13-e22572-s004.zip › shALK_MT no CCCP.tif]

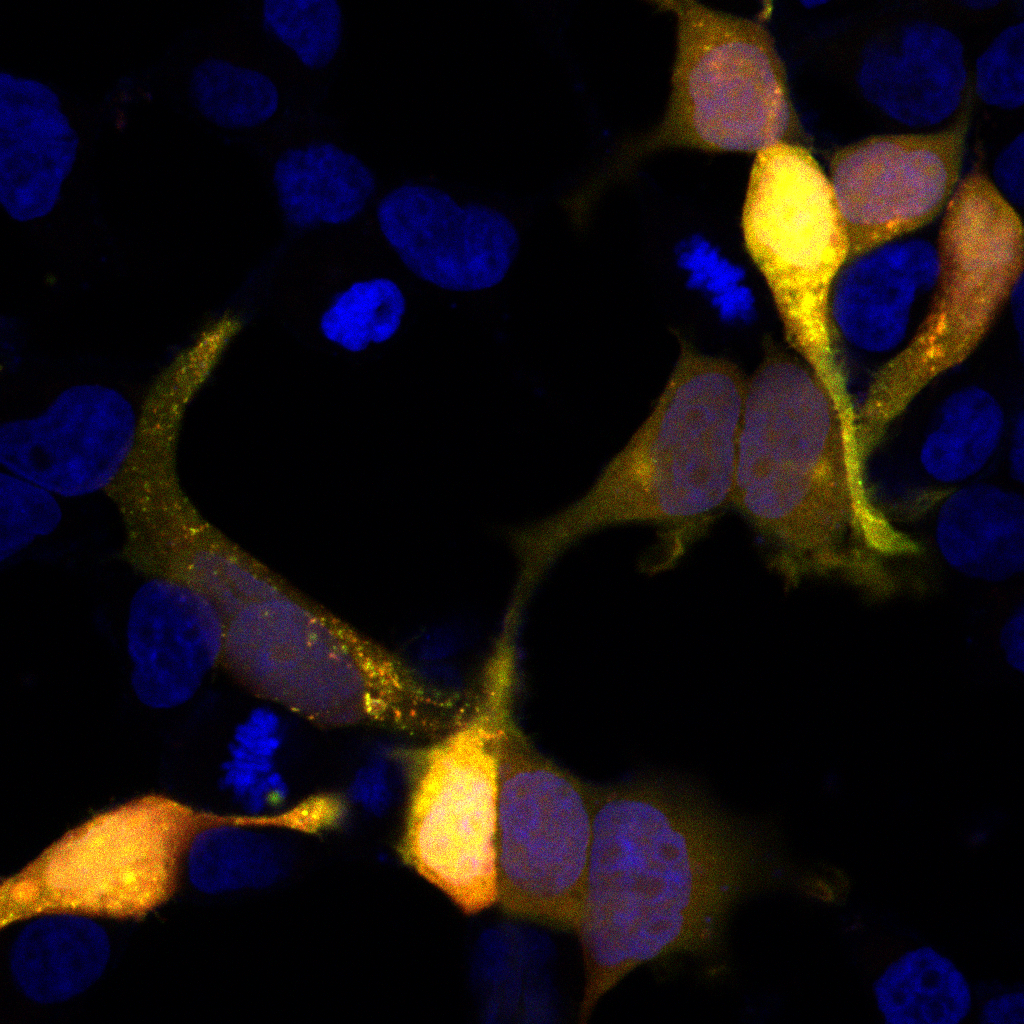

Supplement: Supplementary file 6 — Supporting File 6: advs74789‐sup‐0006‐Data.zip. [file ADVS-13-e22572-s004.zip › shALK CCCP.tif]

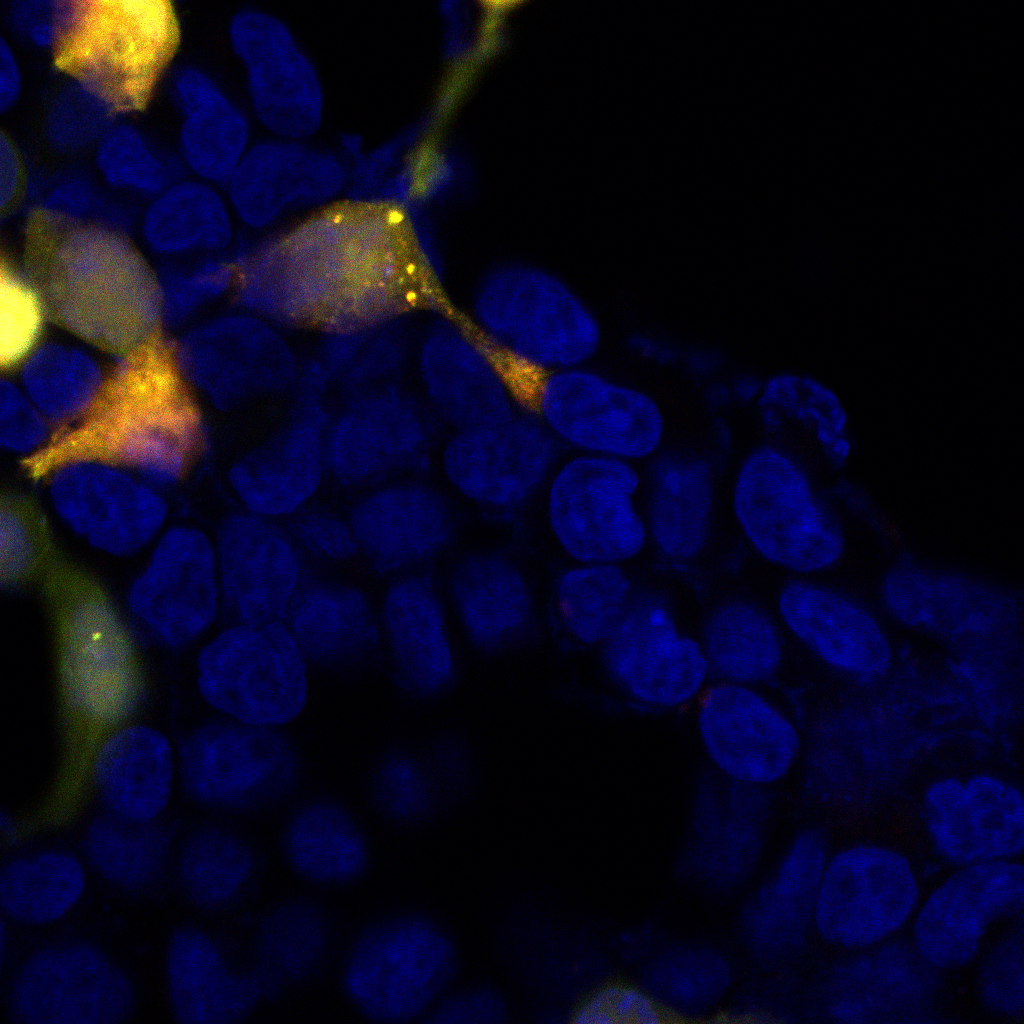

Supplement: Supplementary file 6 — Supporting File 6: advs74789‐sup‐0006‐Data.zip. [file ADVS-13-e22572-s004.zip › shcon CCCP.tif]

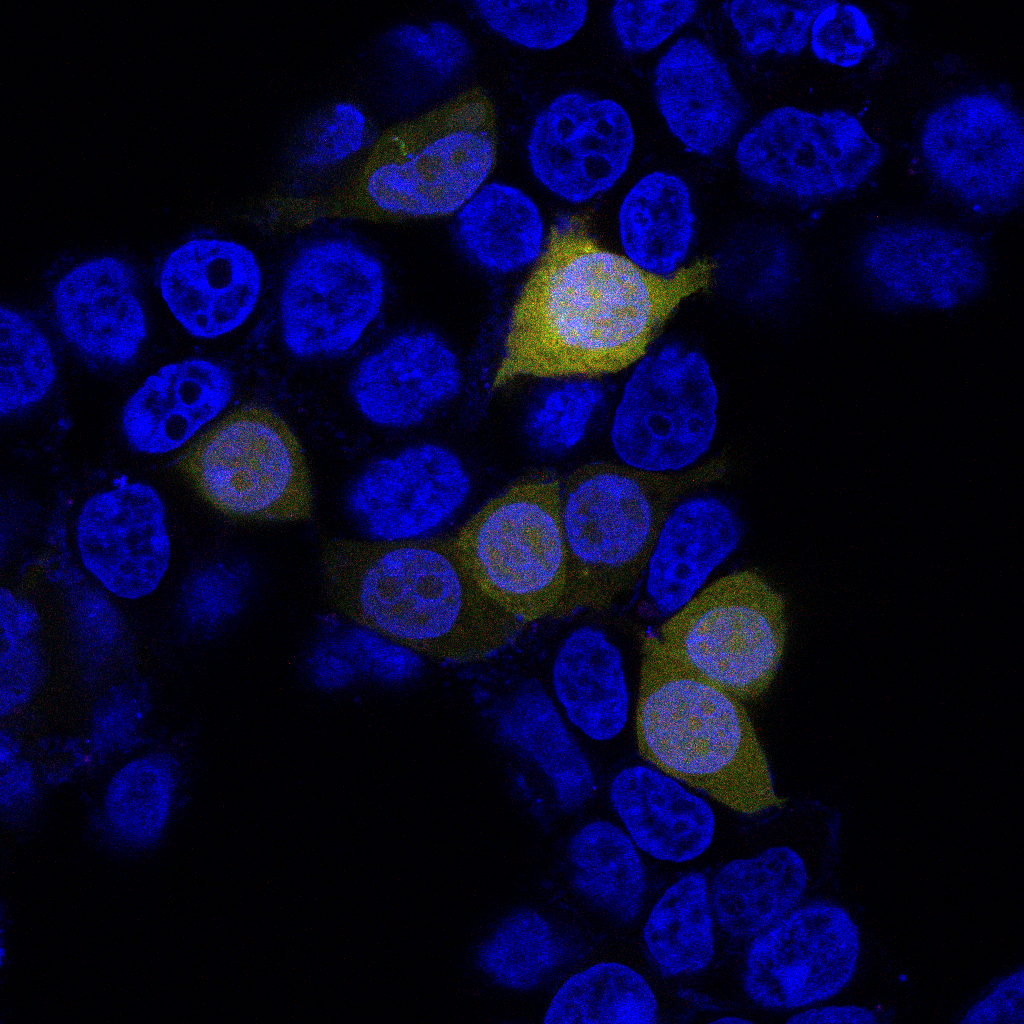

Supplement: Supplementary file 6 — Supporting File 6: advs74789‐sup‐0006‐Data.zip. [file ADVS-13-e22572-s004.zip › shcon no CCCP.tif]

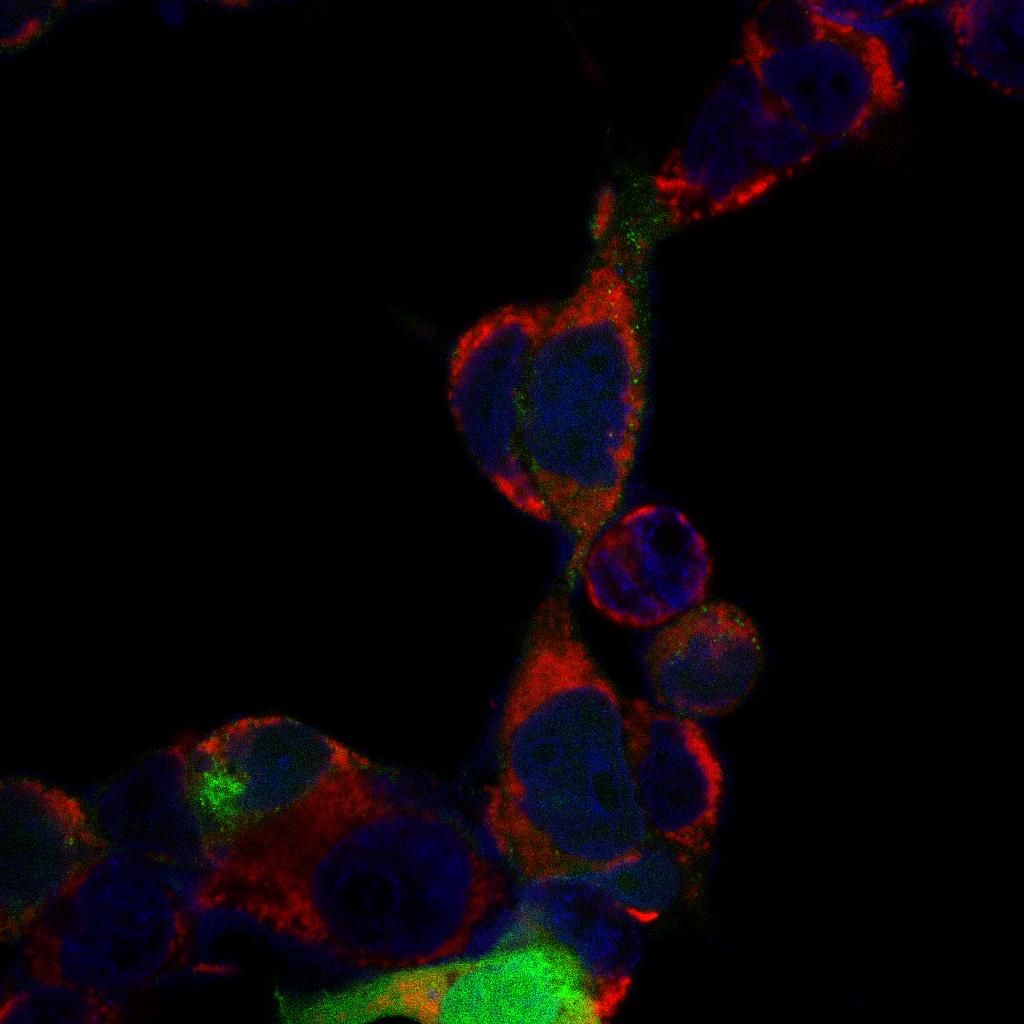

Supplement: Supplementary file 6 — Supporting File 6: advs74789‐sup‐0006‐Data.zip. [file ADVS-13-e22572-s004.zip › shcon CCCP_293_1.tif]

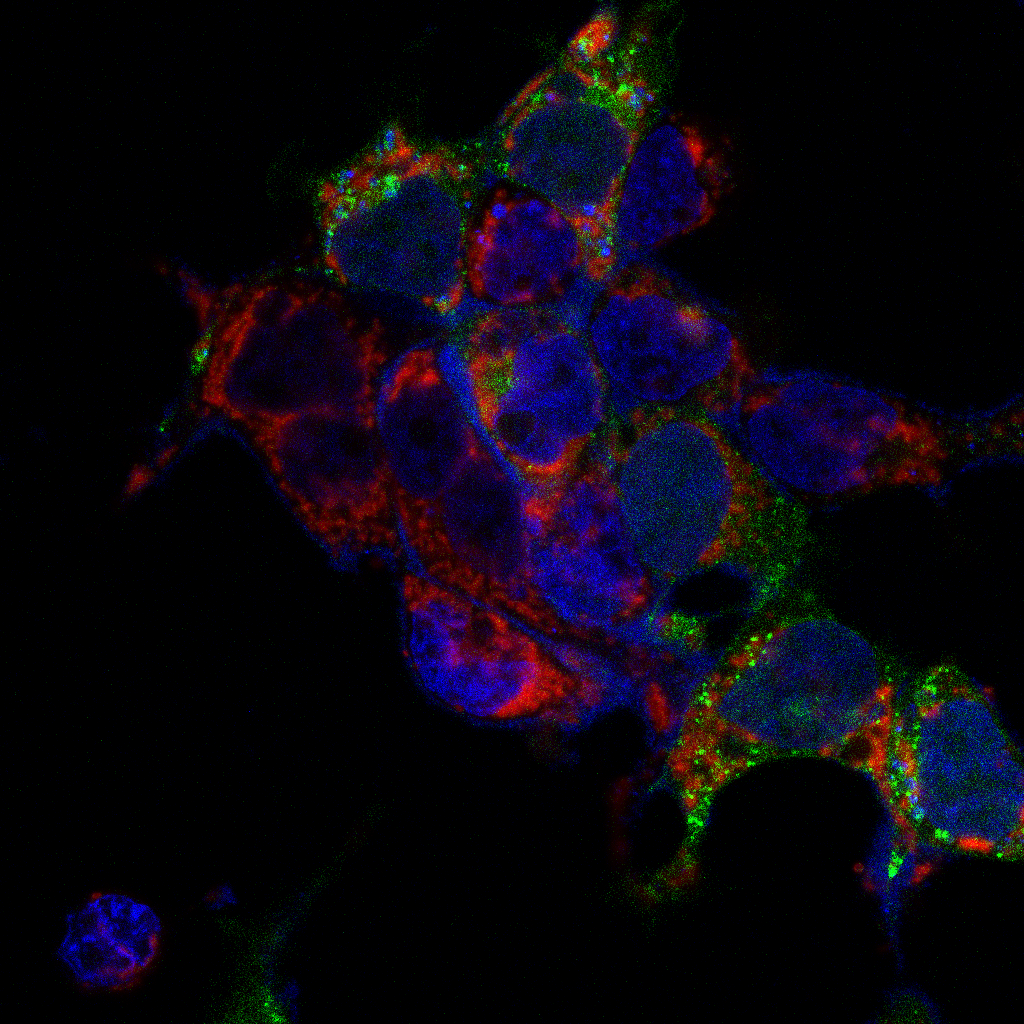

Supplement: Supplementary file 6 — Supporting File 6: advs74789‐sup‐0006‐Data.zip. [file ADVS-13-e22572-s004.zip › shalk CCCP_293_1.tif]

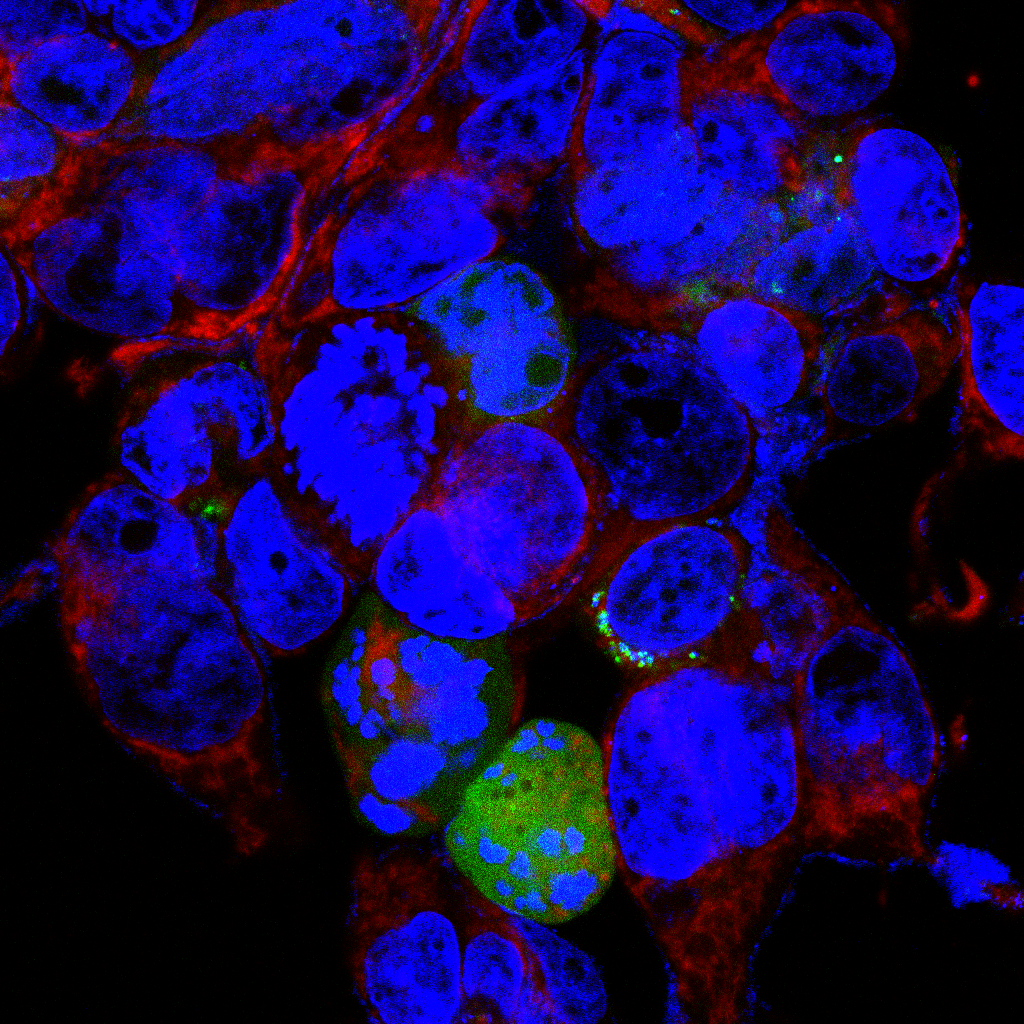

Supplement: Supplementary file 6 — Supporting File 6: advs74789‐sup‐0006‐Data.zip. [file ADVS-13-e22572-s004.zip › shalk no CCCP_293_1.tif]

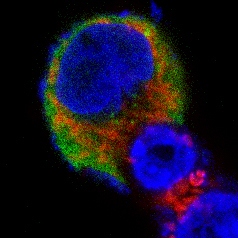

Supplement: Supplementary file 6 — Supporting File 6: advs74789‐sup‐0006‐Data.zip. [file ADVS-13-e22572-s004.zip › ALK-merge.tif]

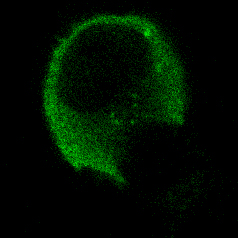

Supplement: Supplementary file 6 — Supporting File 6: advs74789‐sup‐0006‐Data.zip. [file ADVS-13-e22572-s004.zip › ALK-gfp.tif]

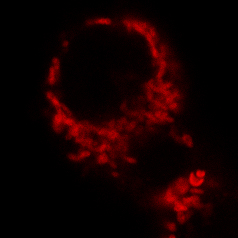

Supplement: Supplementary file 6 — Supporting File 6: advs74789‐sup‐0006‐Data.zip. [file ADVS-13-e22572-s004.zip › ALK-mito.tif]

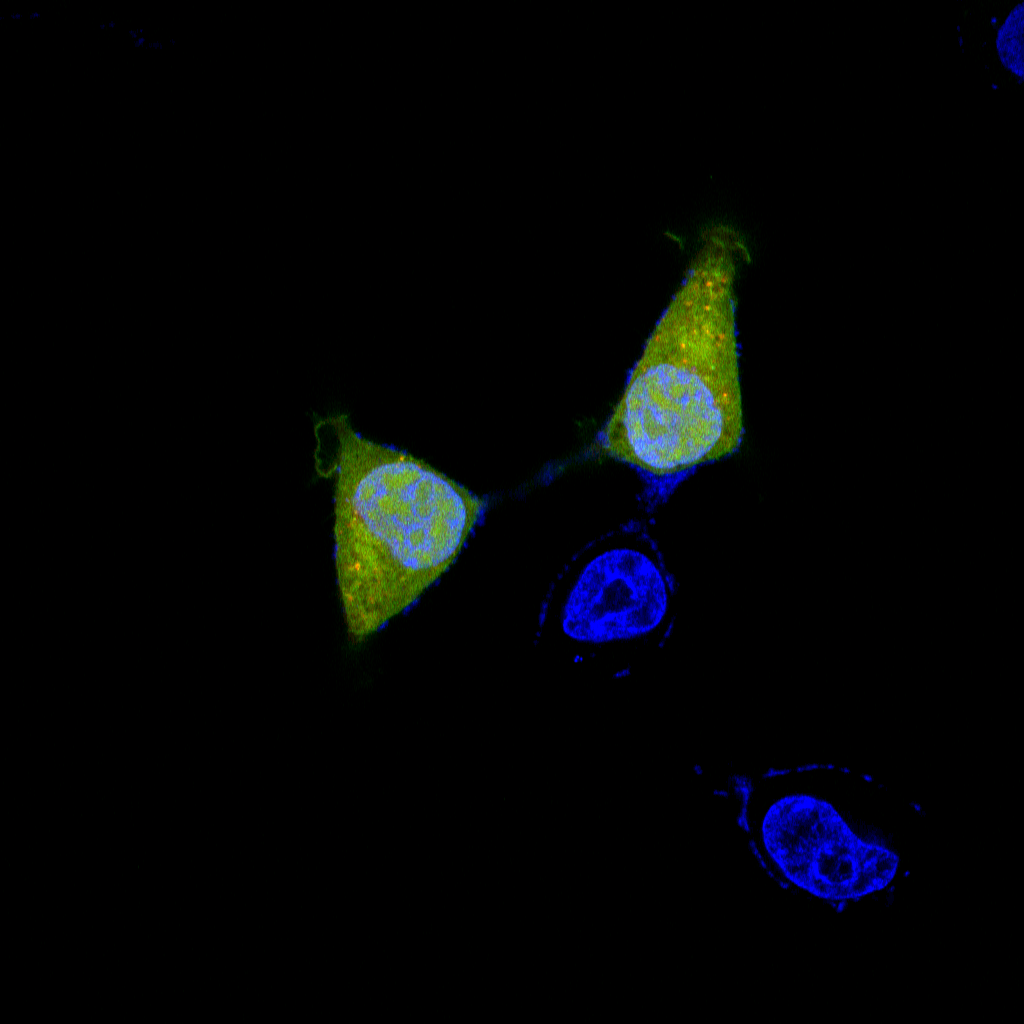

Supplement: Supplementary file 6 — Supporting File 6: advs74789‐sup‐0006‐Data.zip. [file ADVS-13-e22572-s004.zip › shALK_MT no CCCP_1.tif]

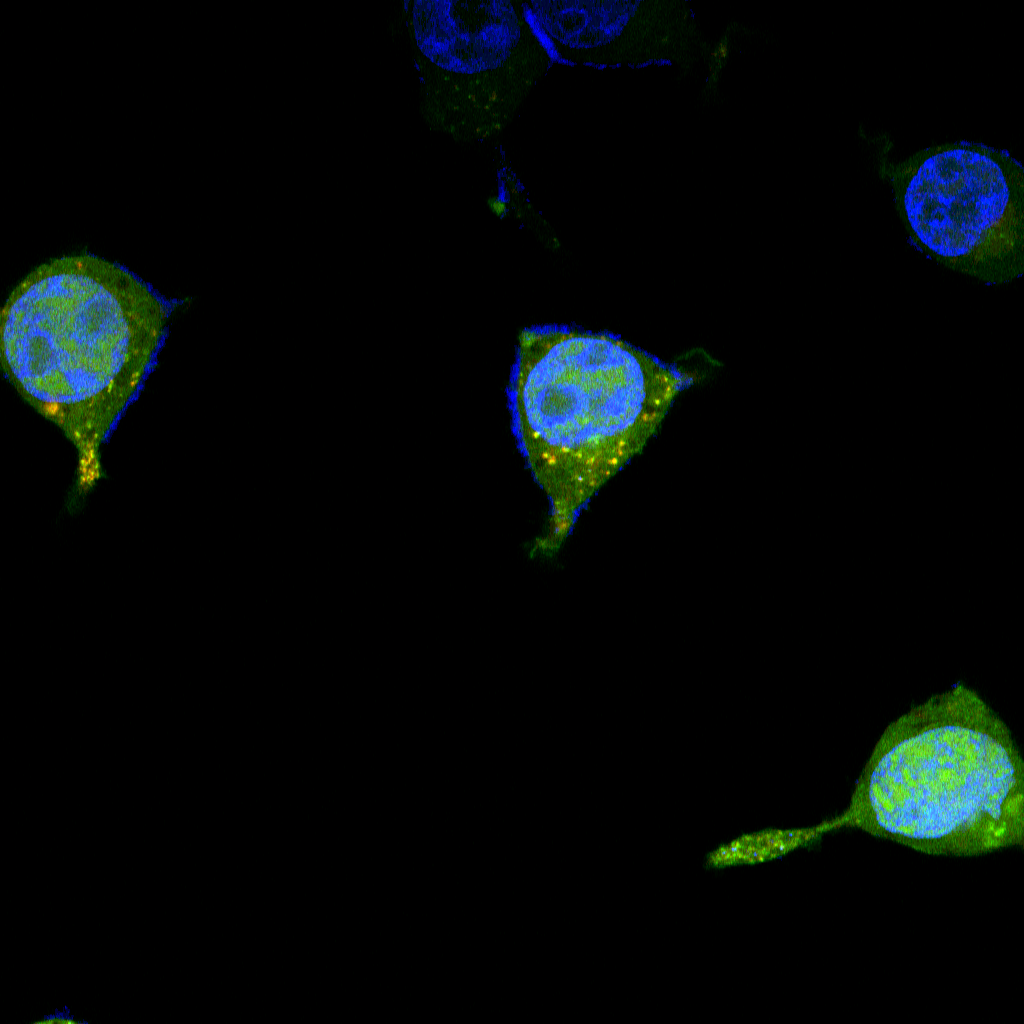

Supplement: Supplementary file 6 — Supporting File 6: advs74789‐sup‐0006‐Data.zip. [file ADVS-13-e22572-s004.zip › shALK_Vec CCCP_1.tif]

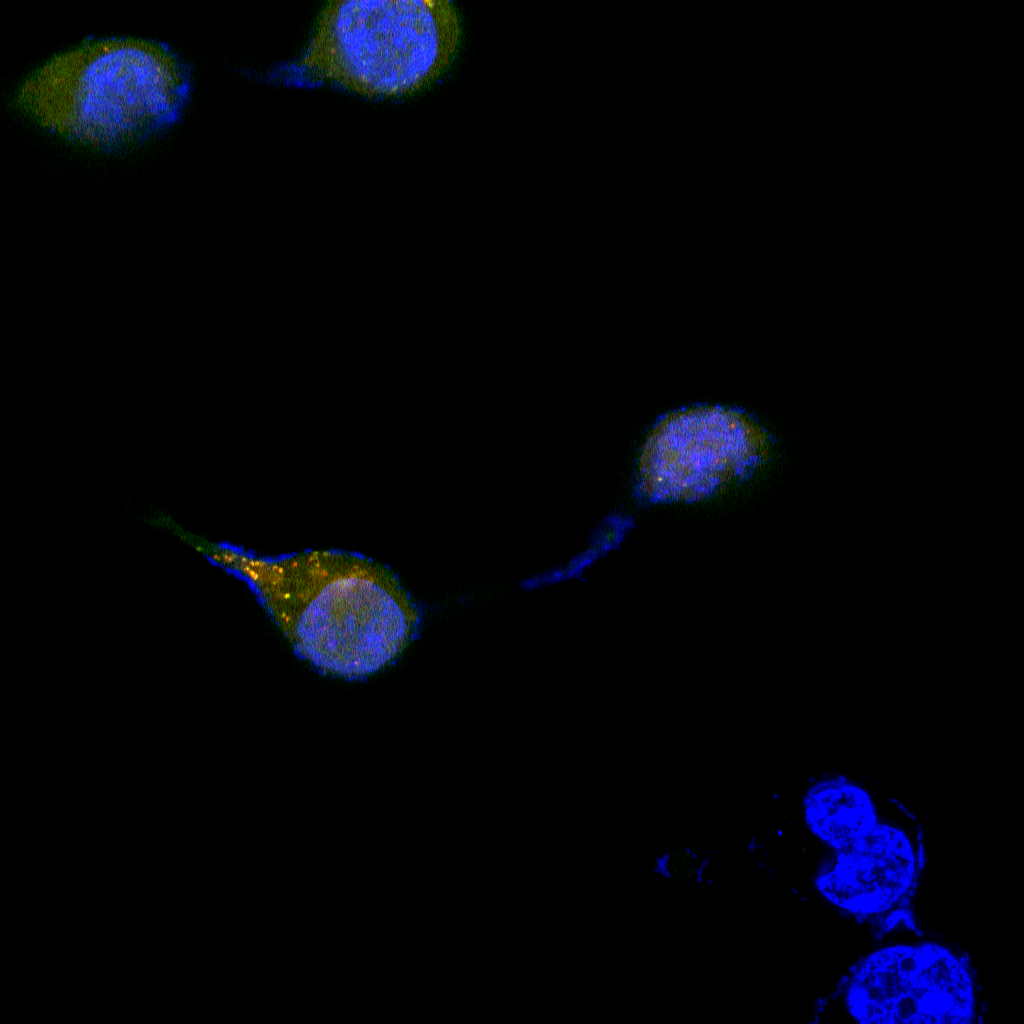

Supplement: Supplementary file 6 — Supporting File 6: advs74789‐sup‐0006‐Data.zip. [file ADVS-13-e22572-s004.zip › shALK_MT CCCP_1.tif]

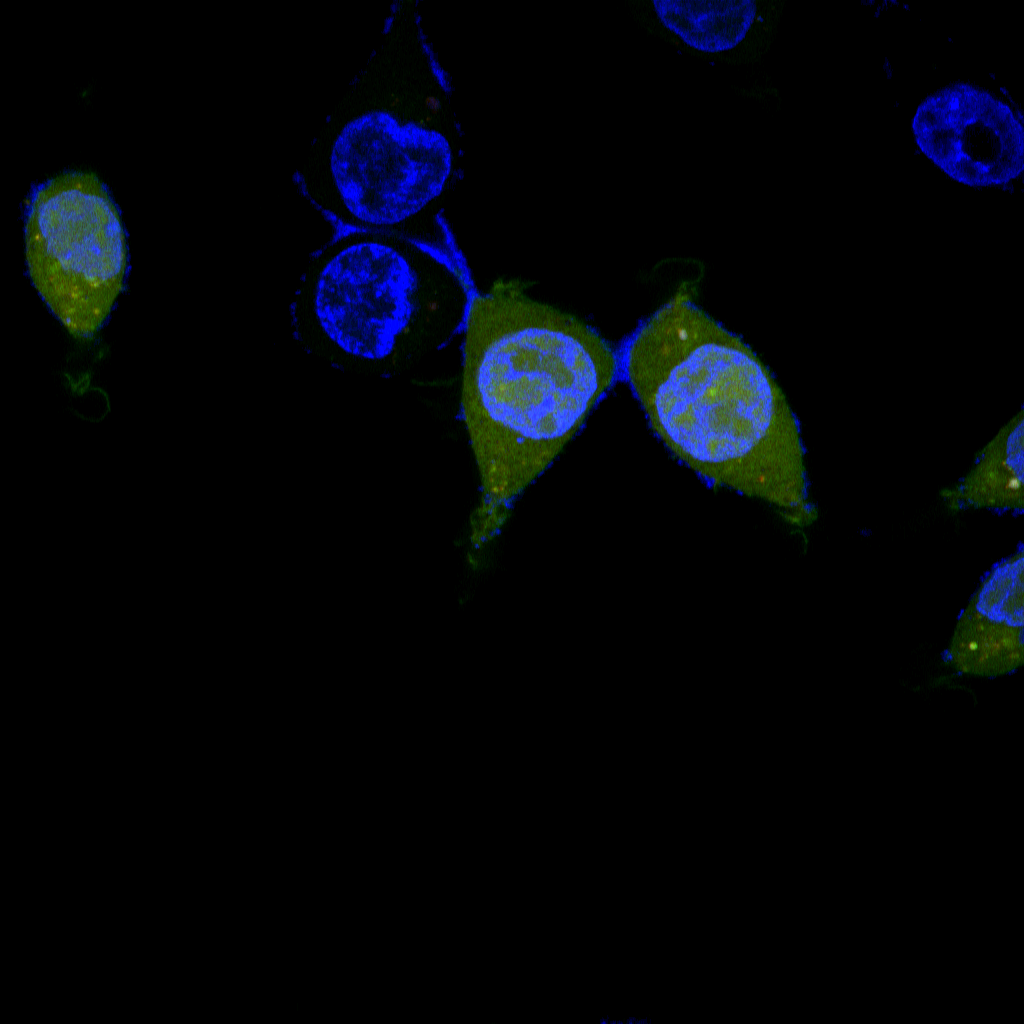

Supplement: Supplementary file 6 — Supporting File 6: advs74789‐sup‐0006‐Data.zip. [file ADVS-13-e22572-s004.zip › shALK_Vec no CCCP_1.tif]

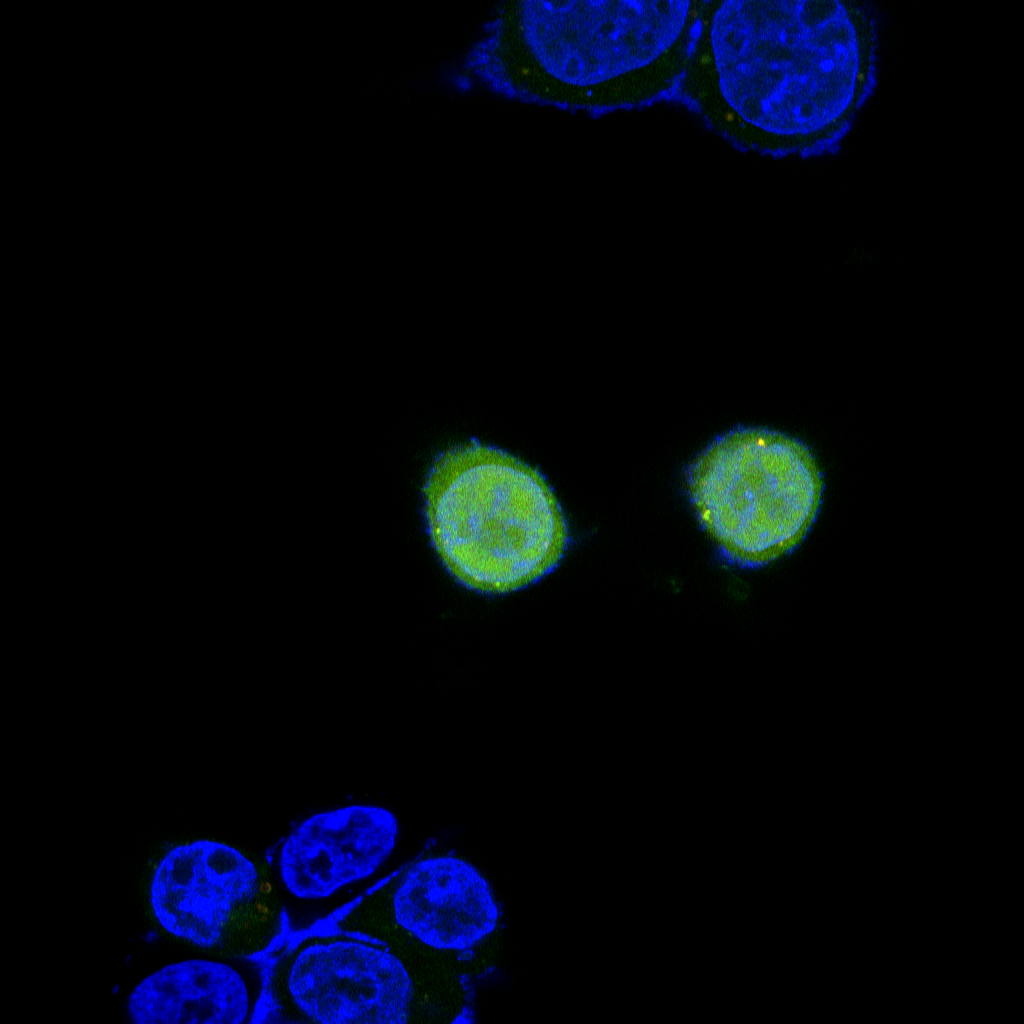

Supplement: Supplementary file 6 — Supporting File 6: advs74789‐sup‐0006‐Data.zip. [file ADVS-13-e22572-s004.zip › shALK_WT CCCP_1.tif]

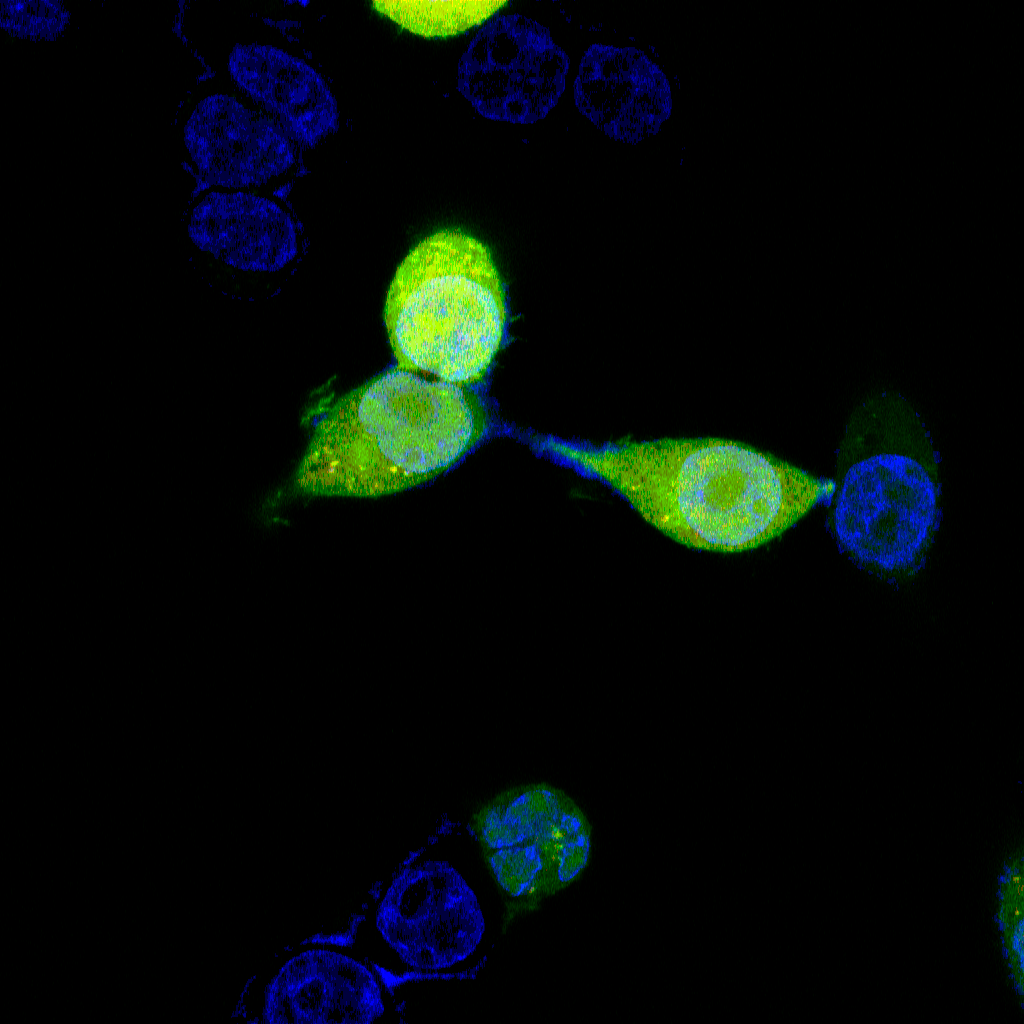

Supplement: Supplementary file 6 — Supporting File 6: advs74789‐sup‐0006‐Data.zip. [file ADVS-13-e22572-s004.zip › shALK_WT no CCCP_1.tif]

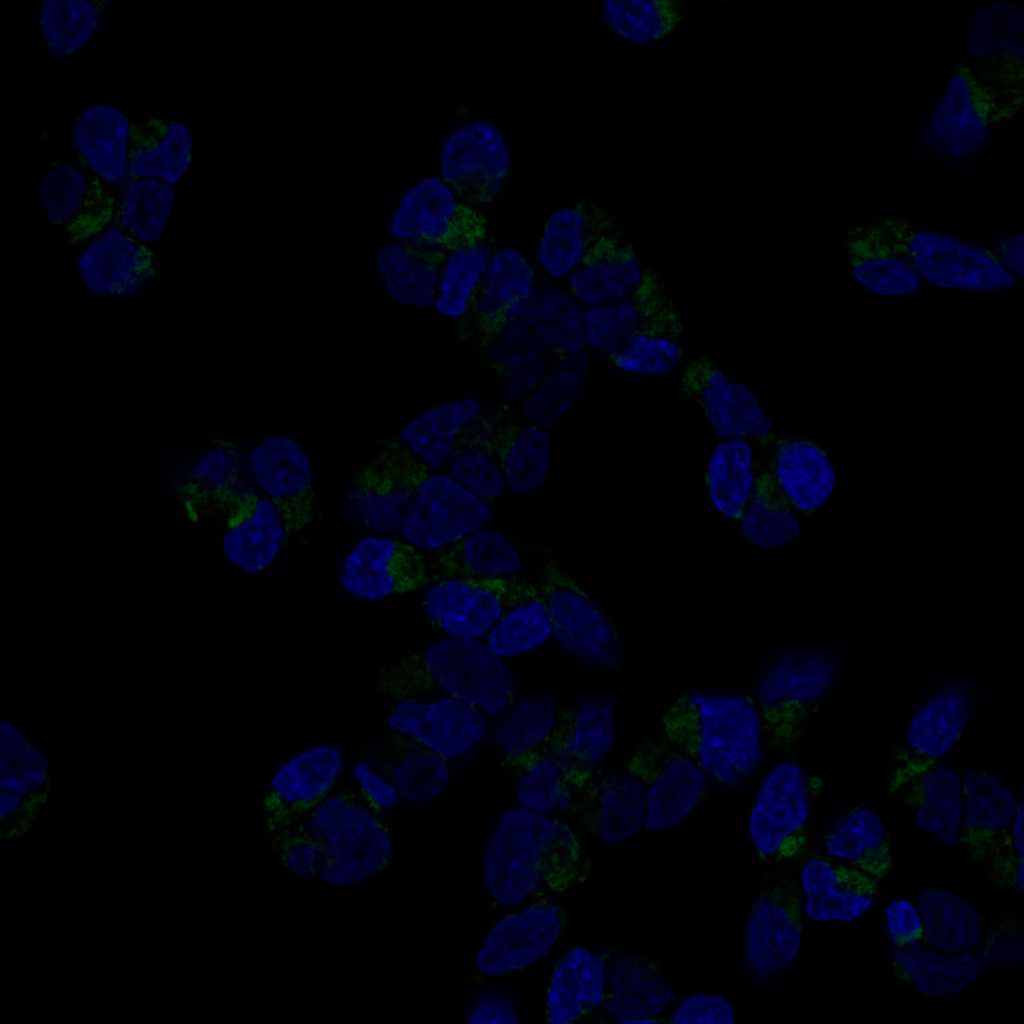

Supplement: Supplementary file 6 — Supporting File 6: advs74789‐sup‐0006‐Data.zip. [file ADVS-13-e22572-s004.zip › c_shalk tomm20_merge.tif]

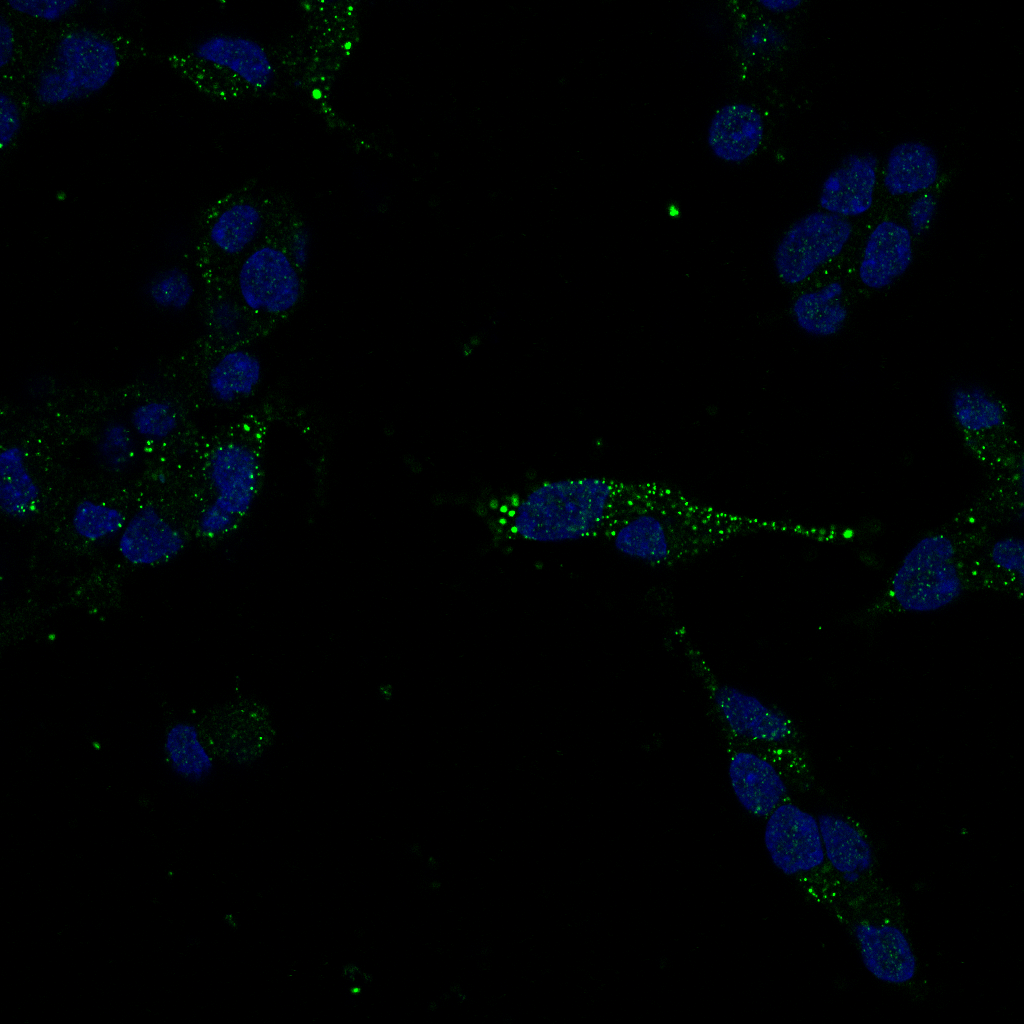

Supplement: Supplementary file 6 — Supporting File 6: advs74789‐sup‐0006‐Data.zip. [file ADVS-13-e22572-s004.zip › d_Vec pink1_merge.tif]

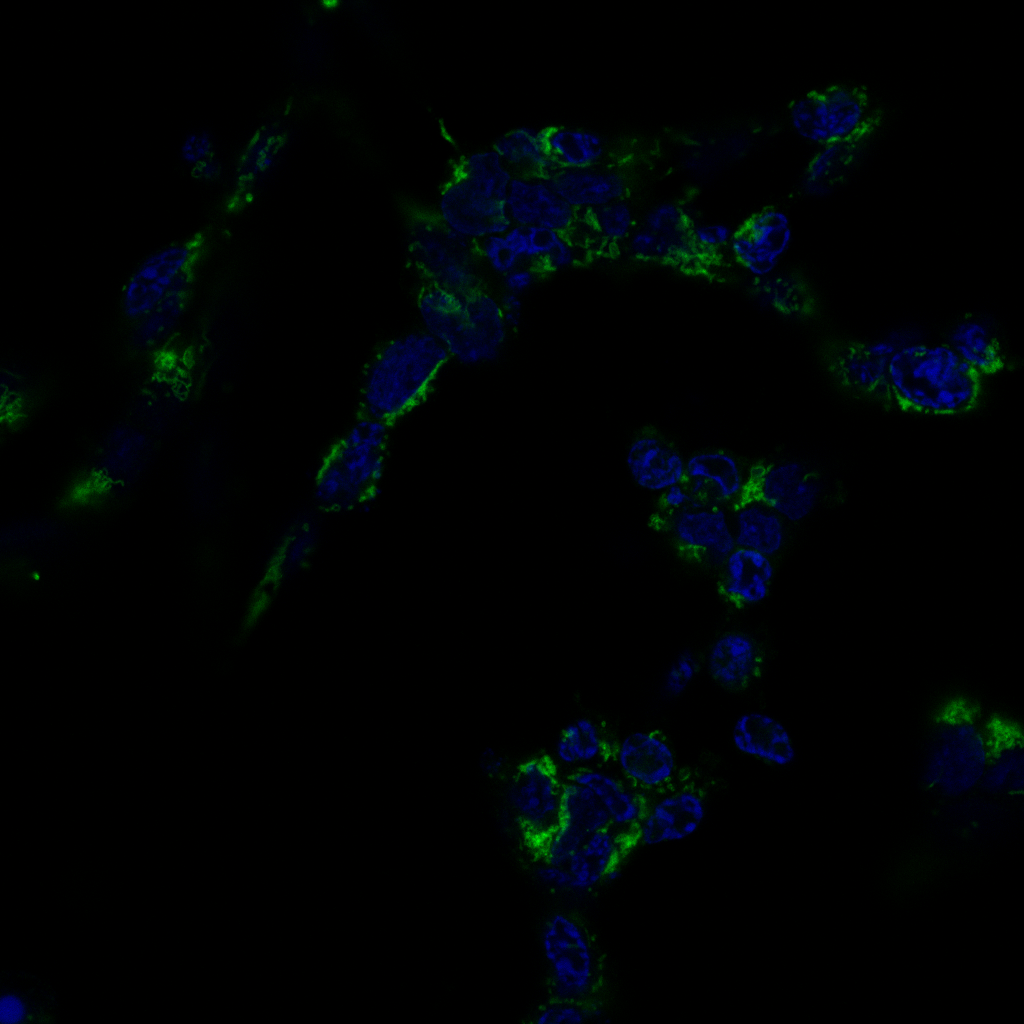

Supplement: Supplementary file 6 — Supporting File 6: advs74789‐sup‐0006‐Data.zip. [file ADVS-13-e22572-s004.zip › c_shcon tomm20_merge.tif]

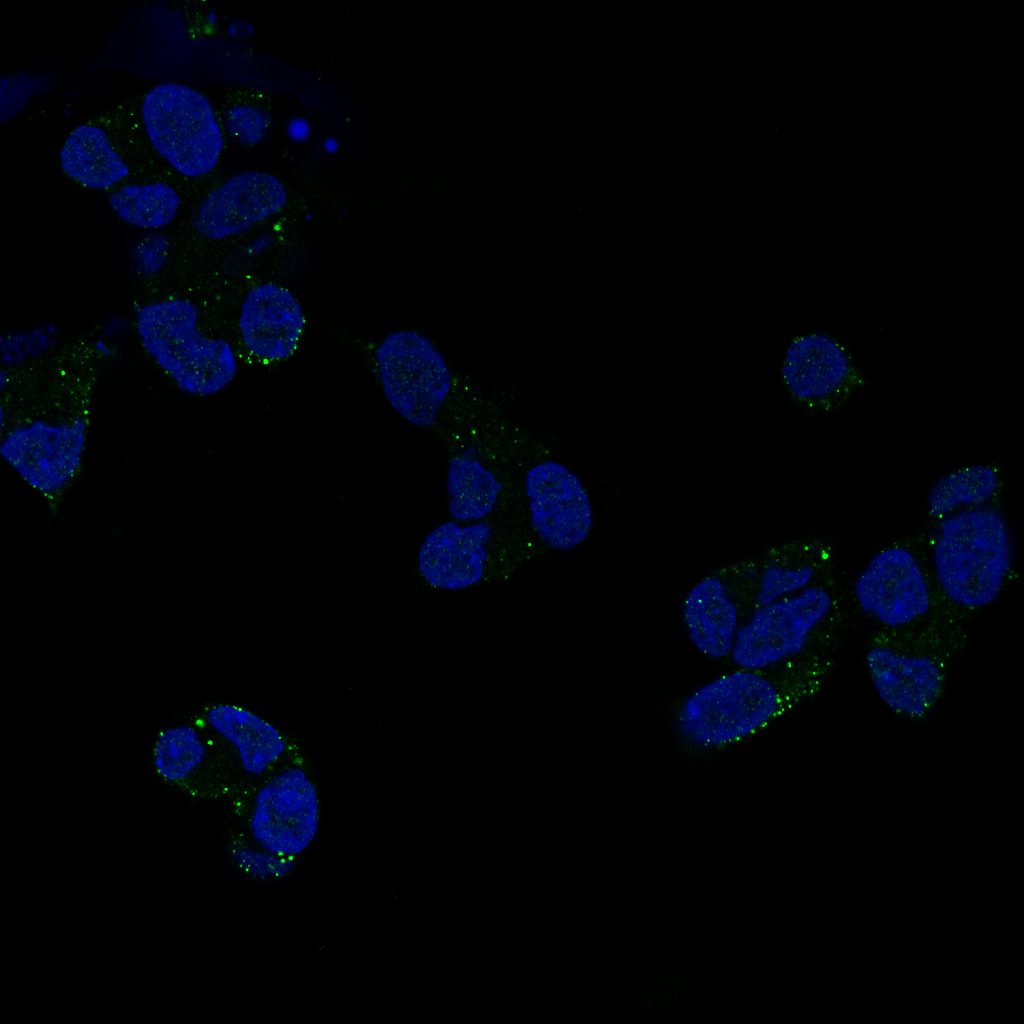

Supplement: Supplementary file 6 — Supporting File 6: advs74789‐sup‐0006‐Data.zip. [file ADVS-13-e22572-s004.zip › d_ALK pink1_merge.tif]

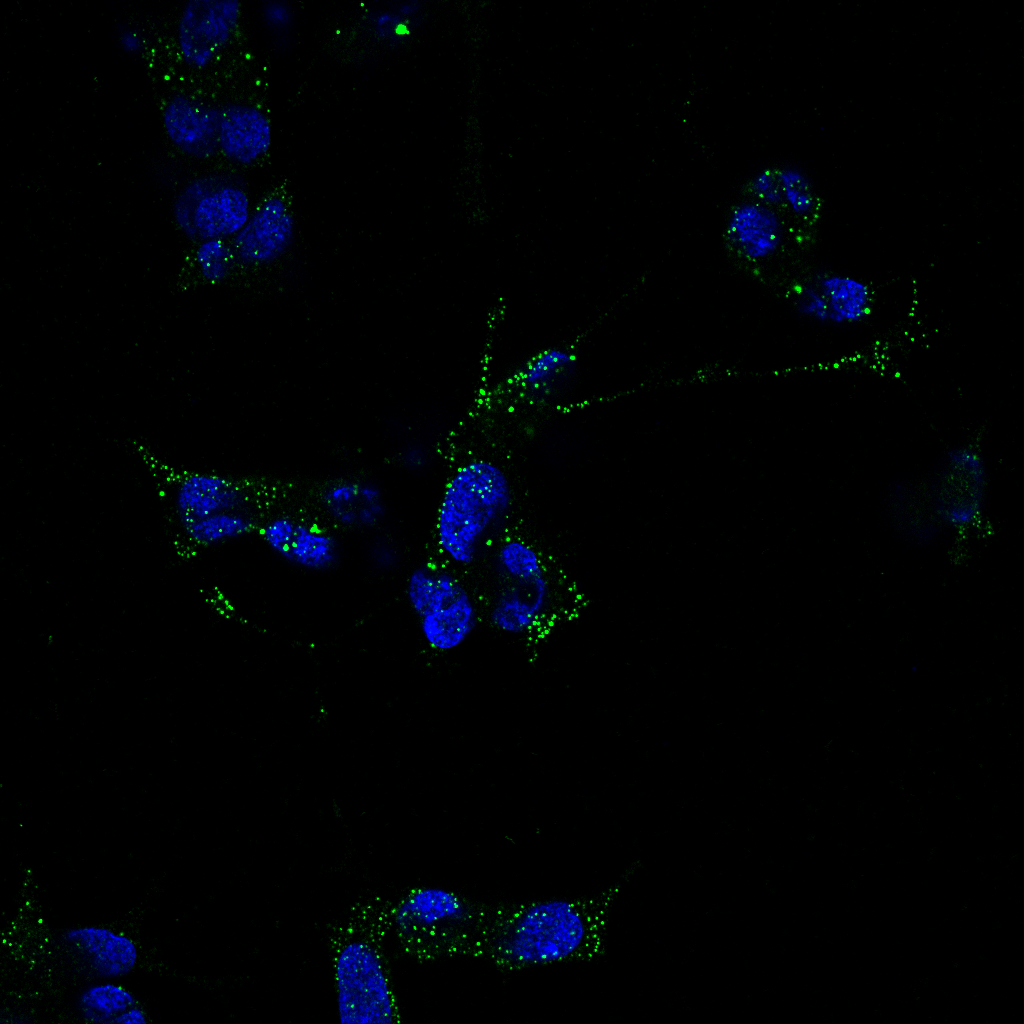

Supplement: Supplementary file 6 — Supporting File 6: advs74789‐sup‐0006‐Data.zip. [file ADVS-13-e22572-s004.zip › e_shALK pink1_merge.tif]

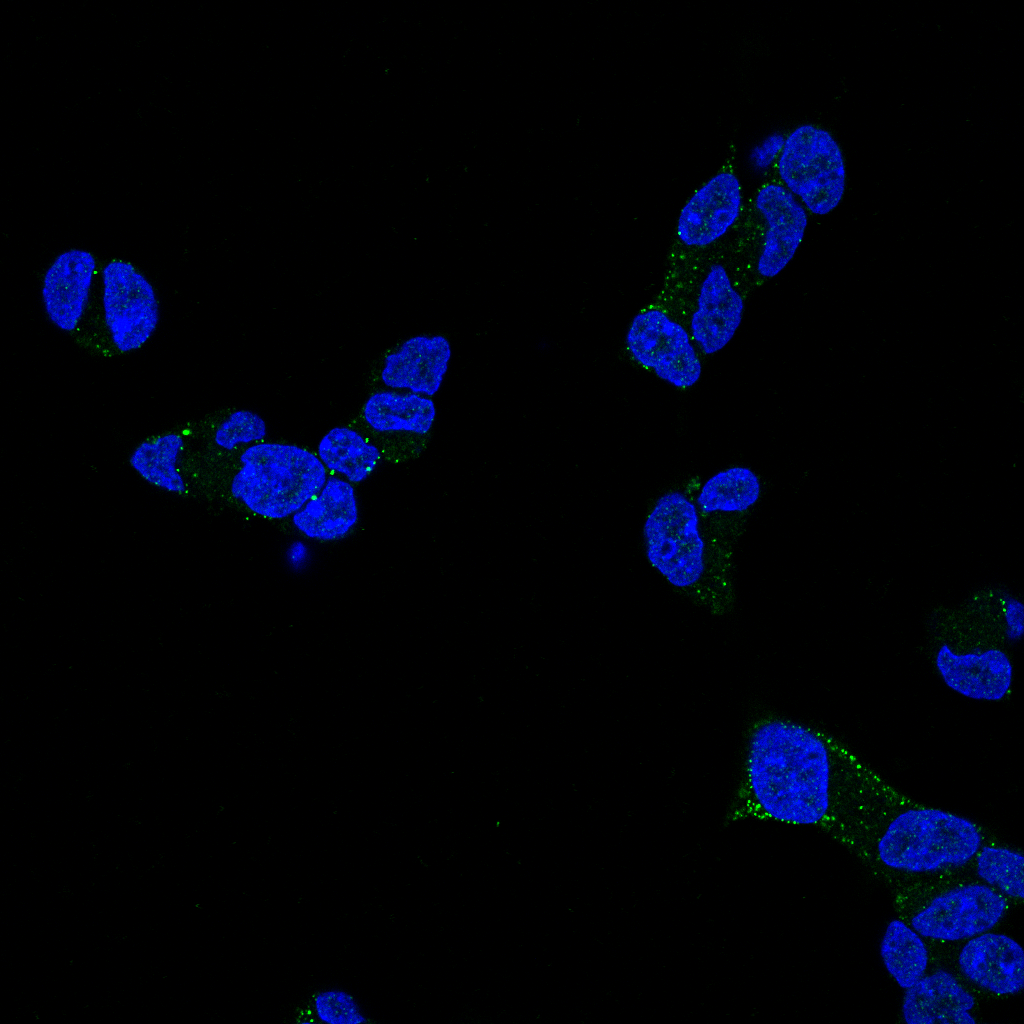

Supplement: Supplementary file 6 — Supporting File 6: advs74789‐sup‐0006‐Data.zip. [file ADVS-13-e22572-s004.zip › e_shcon pink1_merge.tif]

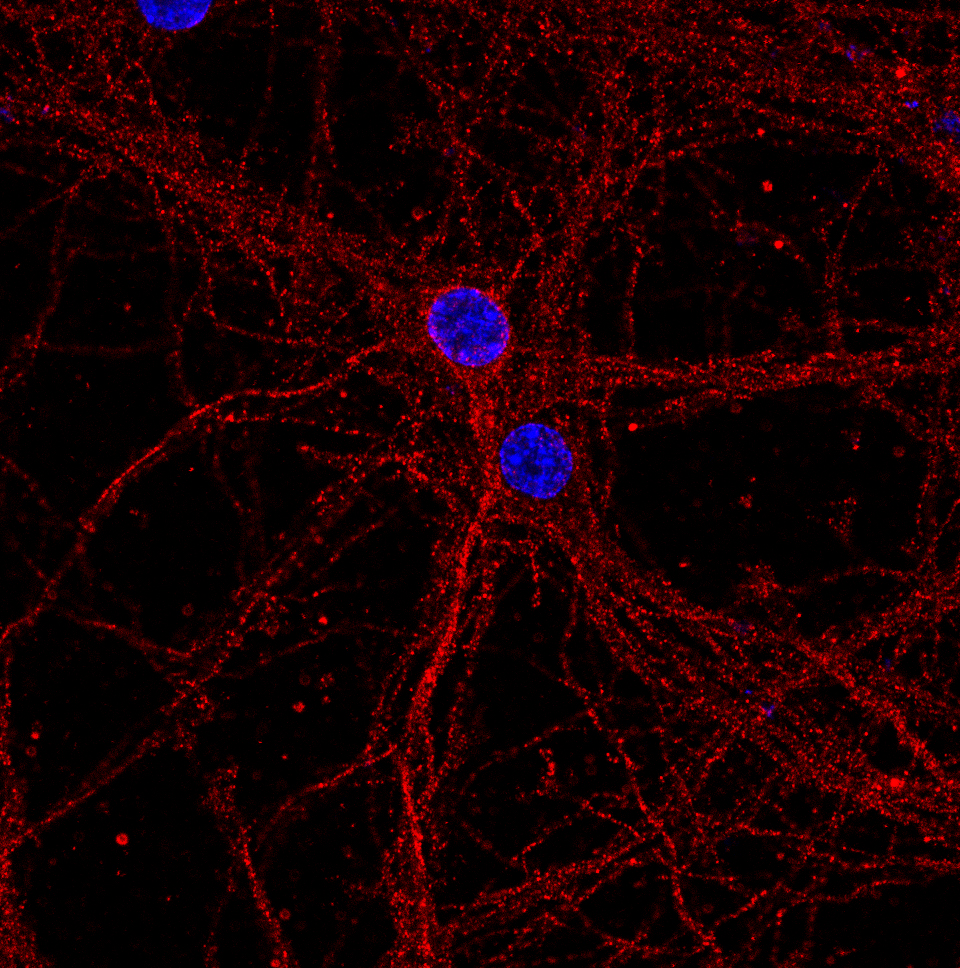

Supplement: Supplementary file 6 — Supporting File 6: advs74789‐sup‐0006‐Data.zip. [file ADVS-13-e22572-s004.zip › f_ALKBH3 tomm20 merge.tif]

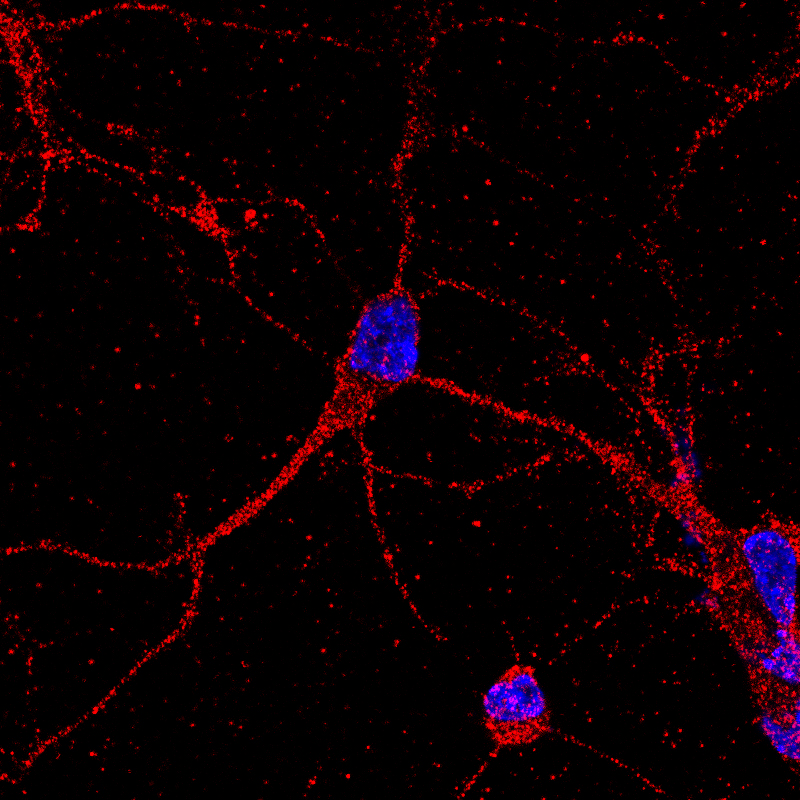

Supplement: Supplementary file 6 — Supporting File 6: advs74789‐sup‐0006‐Data.zip. [file ADVS-13-e22572-s004.zip › f_Vec tomm20 merge.tif]

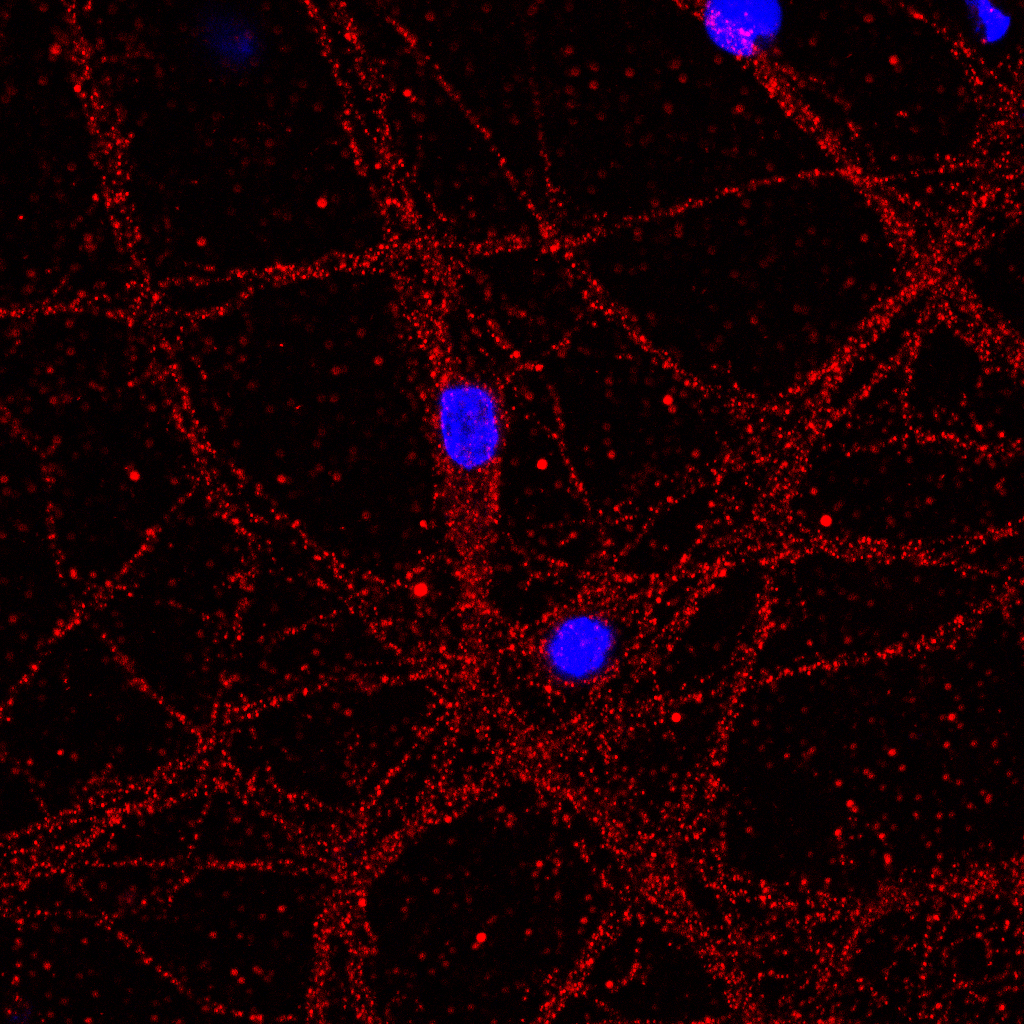

Supplement: Supplementary file 6 — Supporting File 6: advs74789‐sup‐0006‐Data.zip. [file ADVS-13-e22572-s004.zip › g_shalk tomm20_merge.tif]

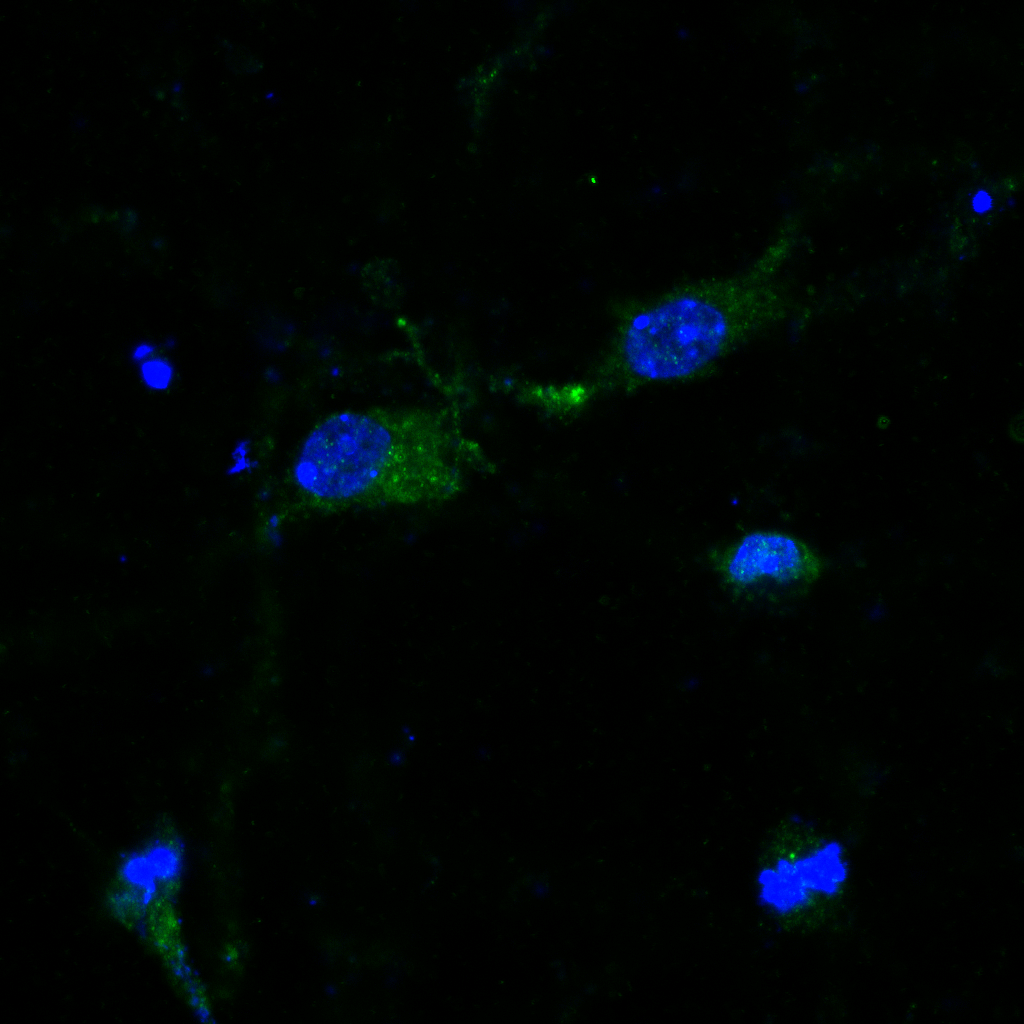

Supplement: Supplementary file 6 — Supporting File 6: advs74789‐sup‐0006‐Data.zip. [file ADVS-13-e22572-s004.zip › h_Vec Pink1 merge.tif]

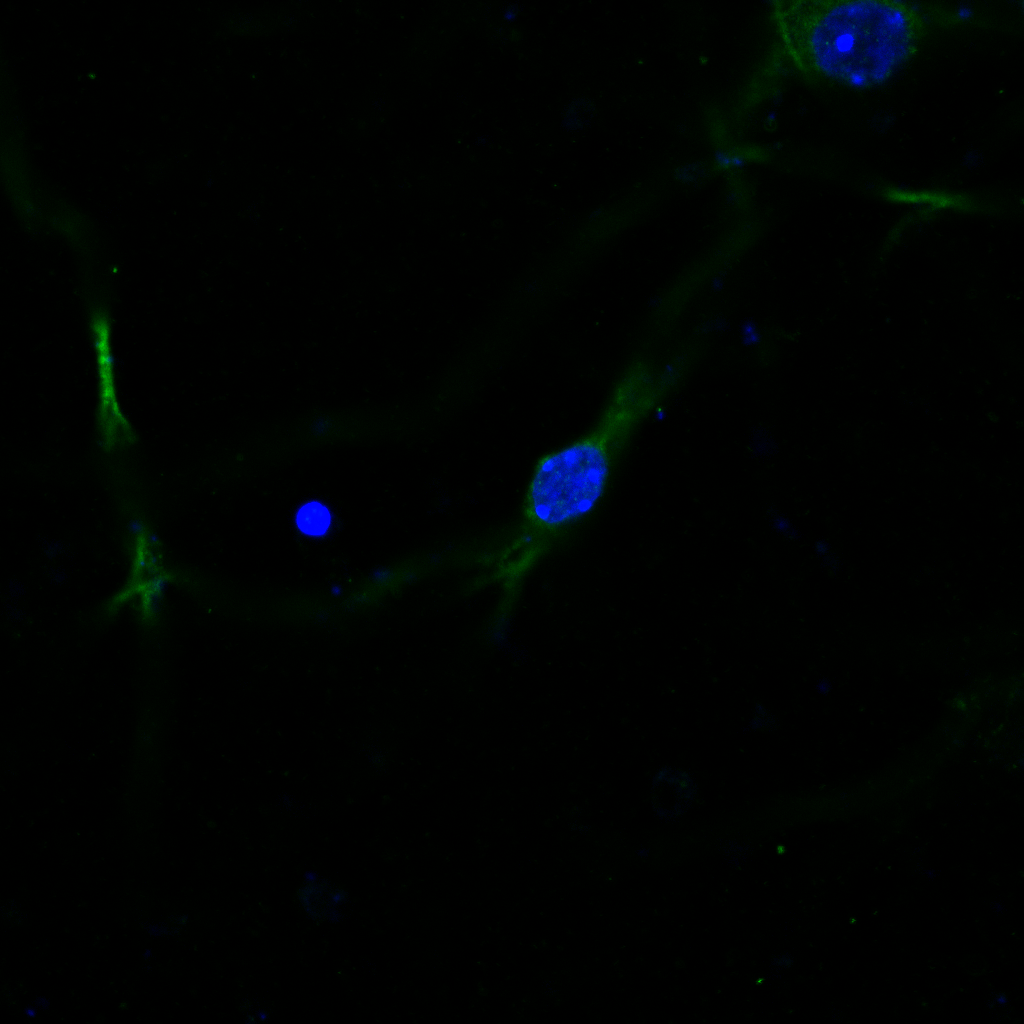

Supplement: Supplementary file 6 — Supporting File 6: advs74789‐sup‐0006‐Data.zip. [file ADVS-13-e22572-s004.zip › h_ALK Pink1 merge.tif]

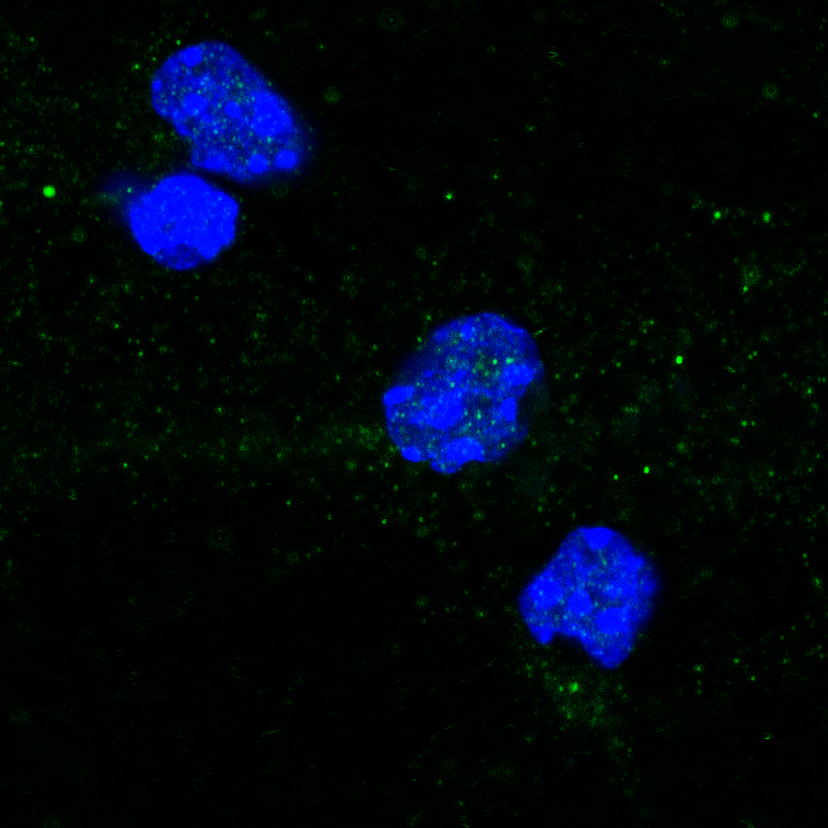

Supplement: Supplementary file 6 — Supporting File 6: advs74789‐sup‐0006‐Data.zip. [file ADVS-13-e22572-s004.zip › i_shcon pink1_merge.tif]

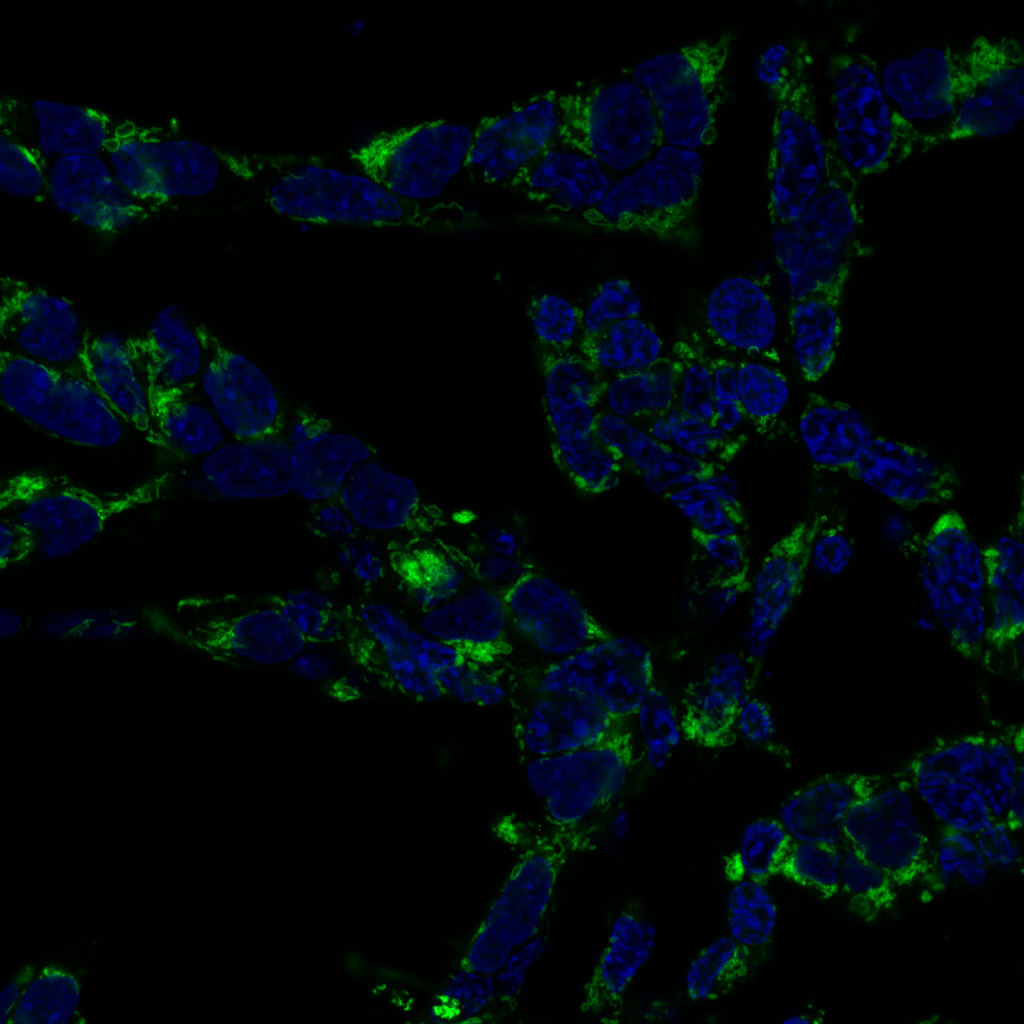

Supplement: Supplementary file 6 — Supporting File 6: advs74789‐sup‐0006‐Data.zip. [file ADVS-13-e22572-s004.zip › b_ALKBH3 tomm20 merge.tif]

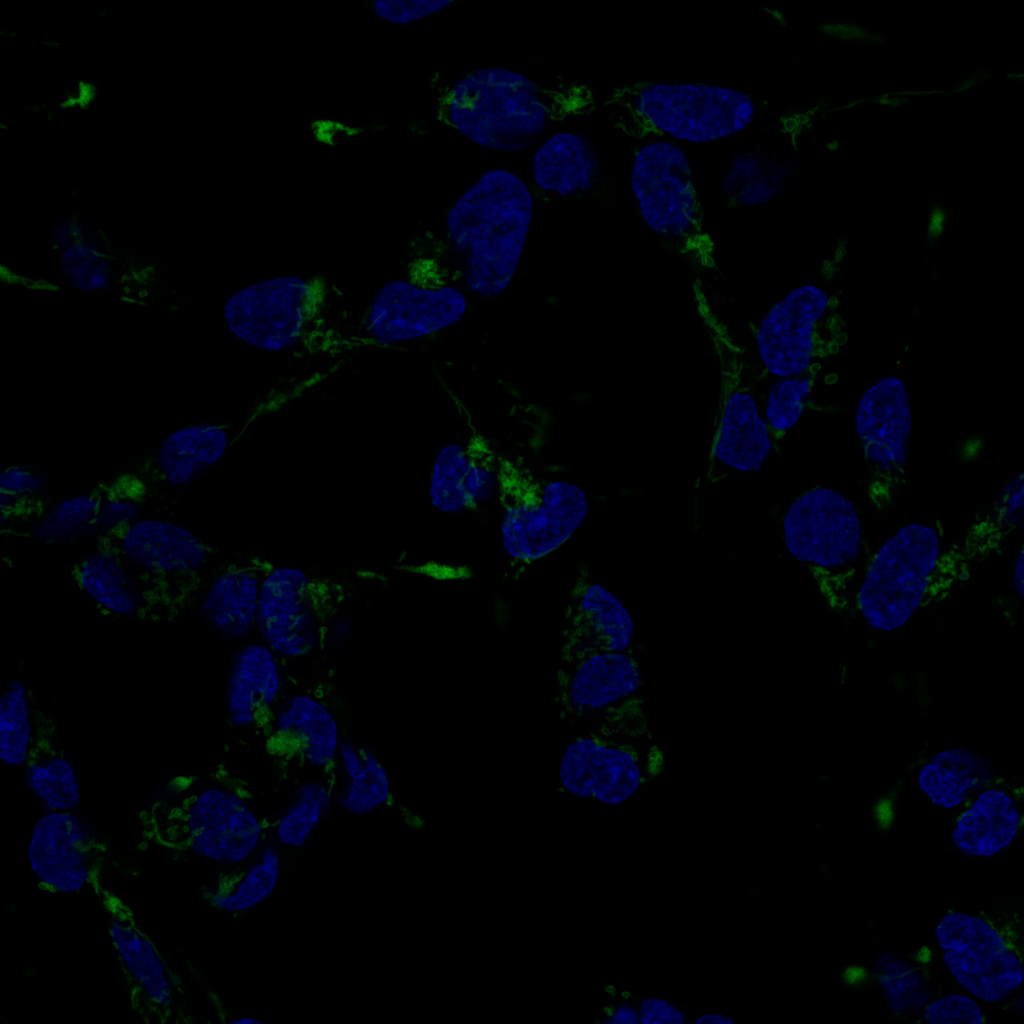

Supplement: Supplementary file 6 — Supporting File 6: advs74789‐sup‐0006‐Data.zip. [file ADVS-13-e22572-s004.zip › b_Vector_tomm20_merge.tif]

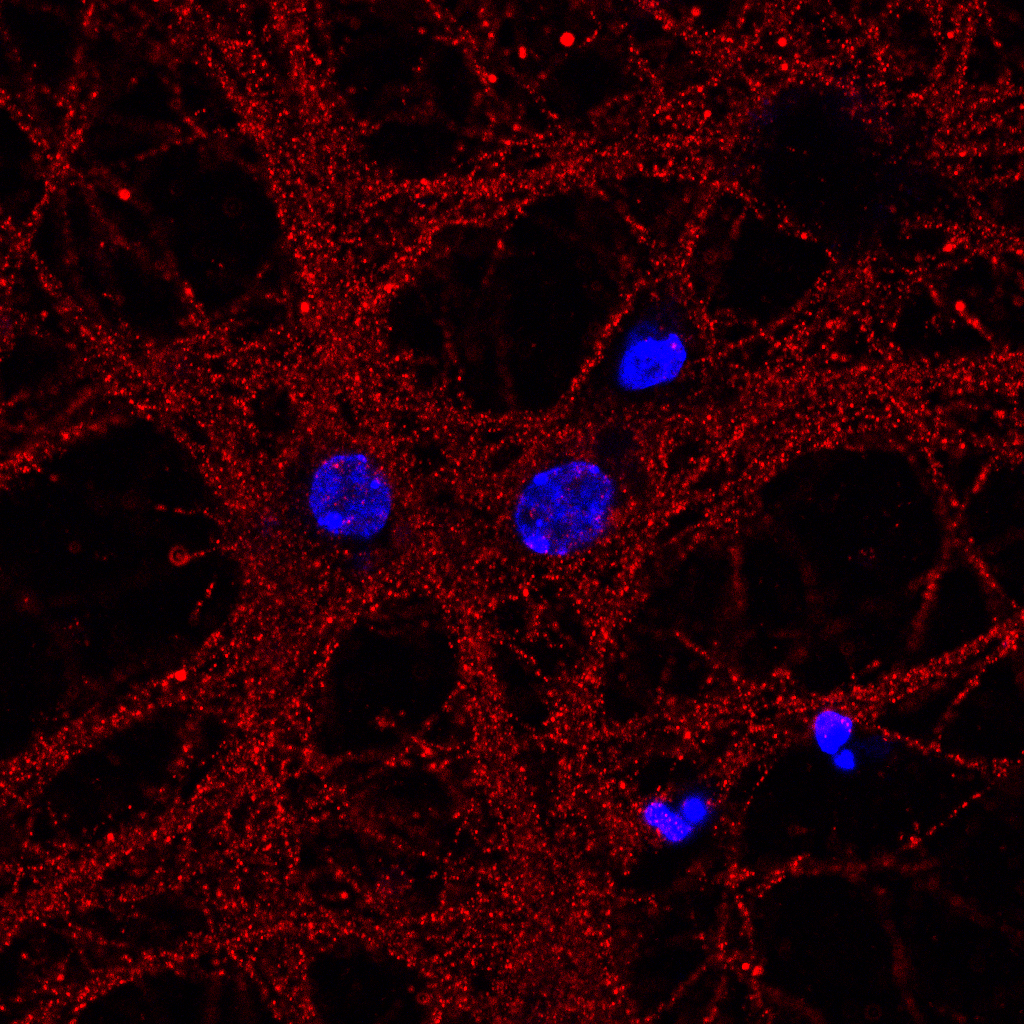

Supplement: Supplementary file 6 — Supporting File 6: advs74789‐sup‐0006‐Data.zip. [file ADVS-13-e22572-s004.zip › g_shcon tomm20_merge.tif]

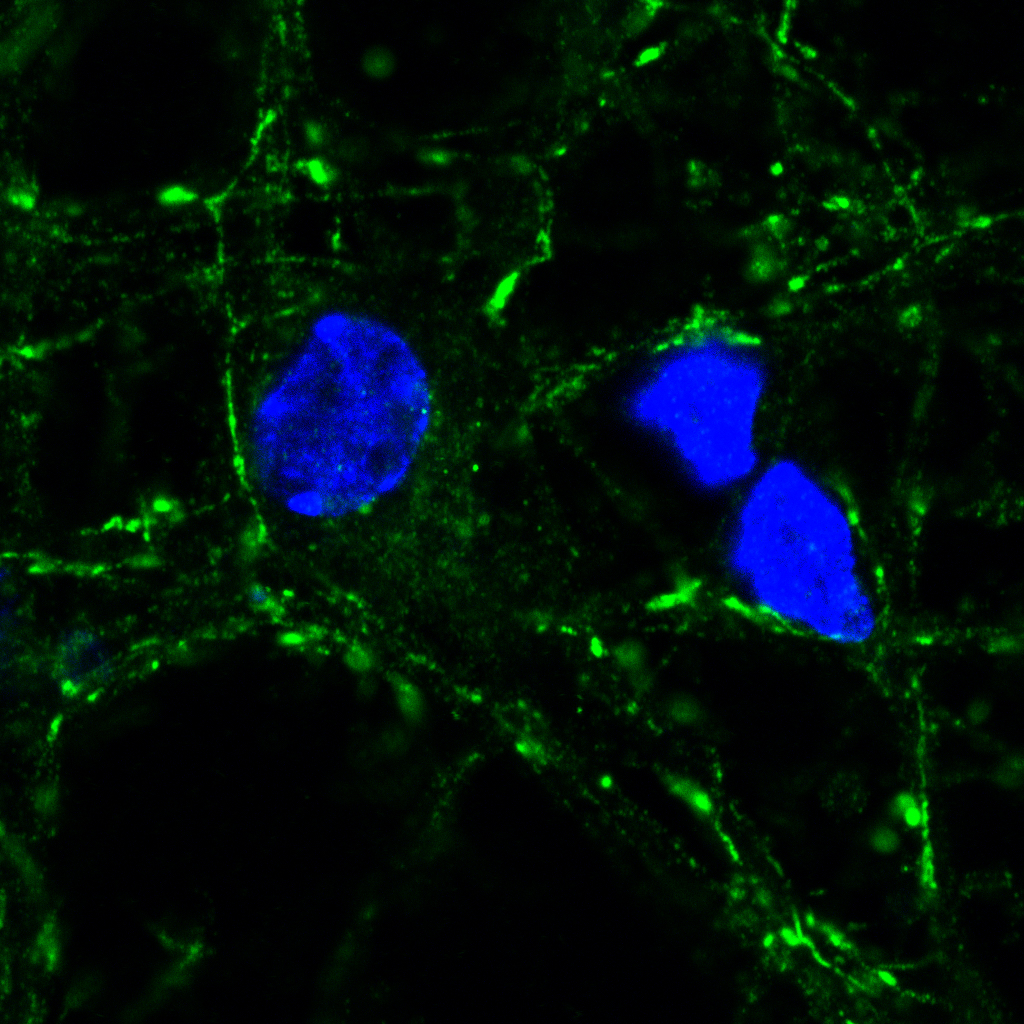

Supplement: Supplementary file 6 — Supporting File 6: advs74789‐sup‐0006‐Data.zip. [file ADVS-13-e22572-s004.zip › i_shalk pink1_merge.tif]

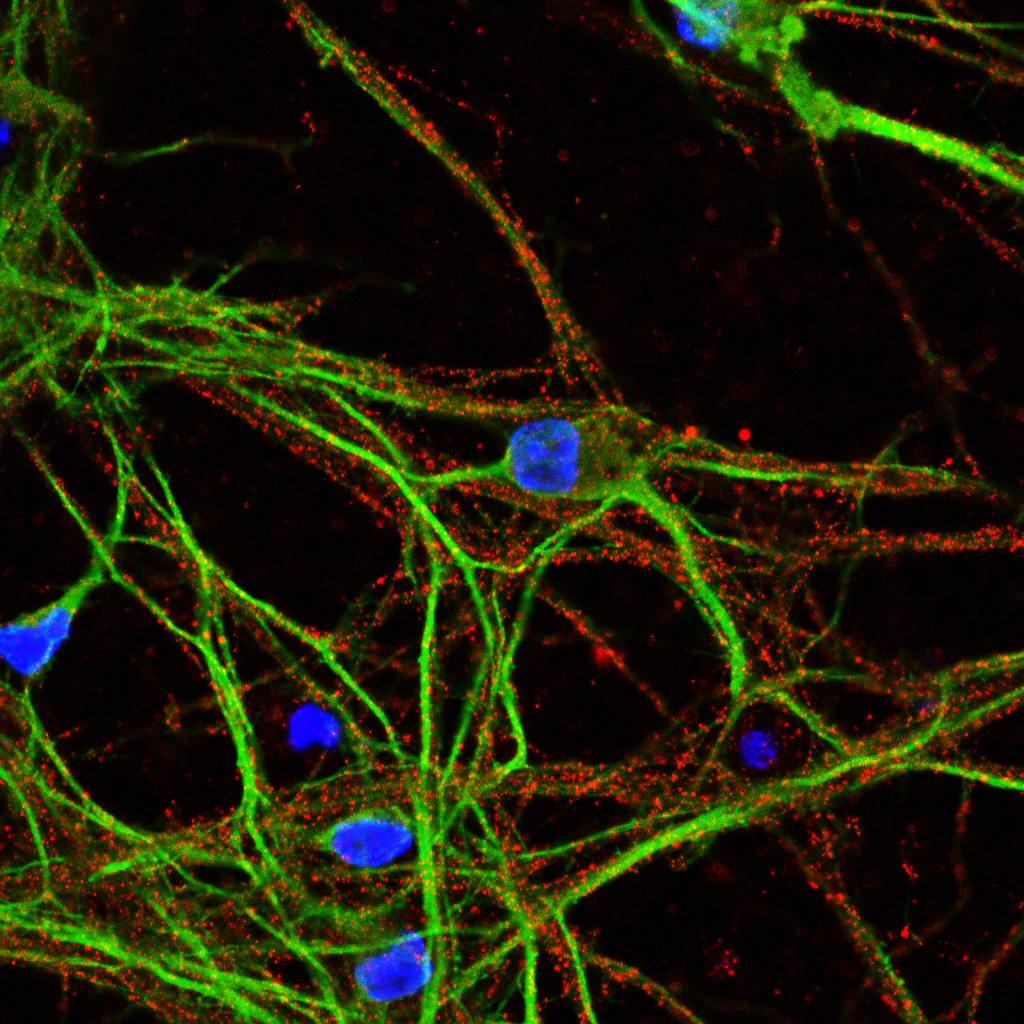

Supplement: Supplementary file 6 — Supporting File 6: advs74789‐sup‐0006‐Data.zip. [file ADVS-13-e22572-s004.zip › p_vec_merge.jpg]

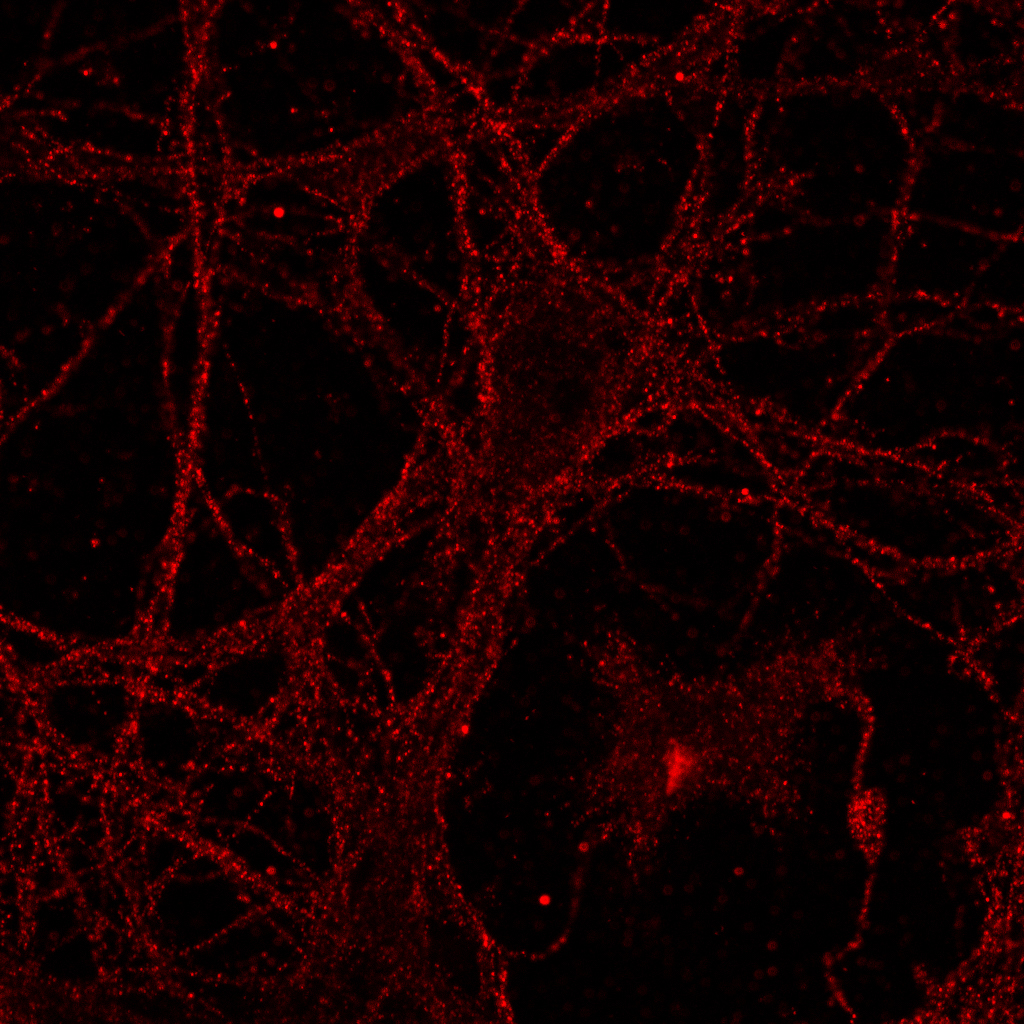

Supplement: Supplementary file 6 — Supporting File 6: advs74789‐sup‐0006‐Data.zip. [file ADVS-13-e22572-s004.zip › p_alk_tomm20-594.jpg]

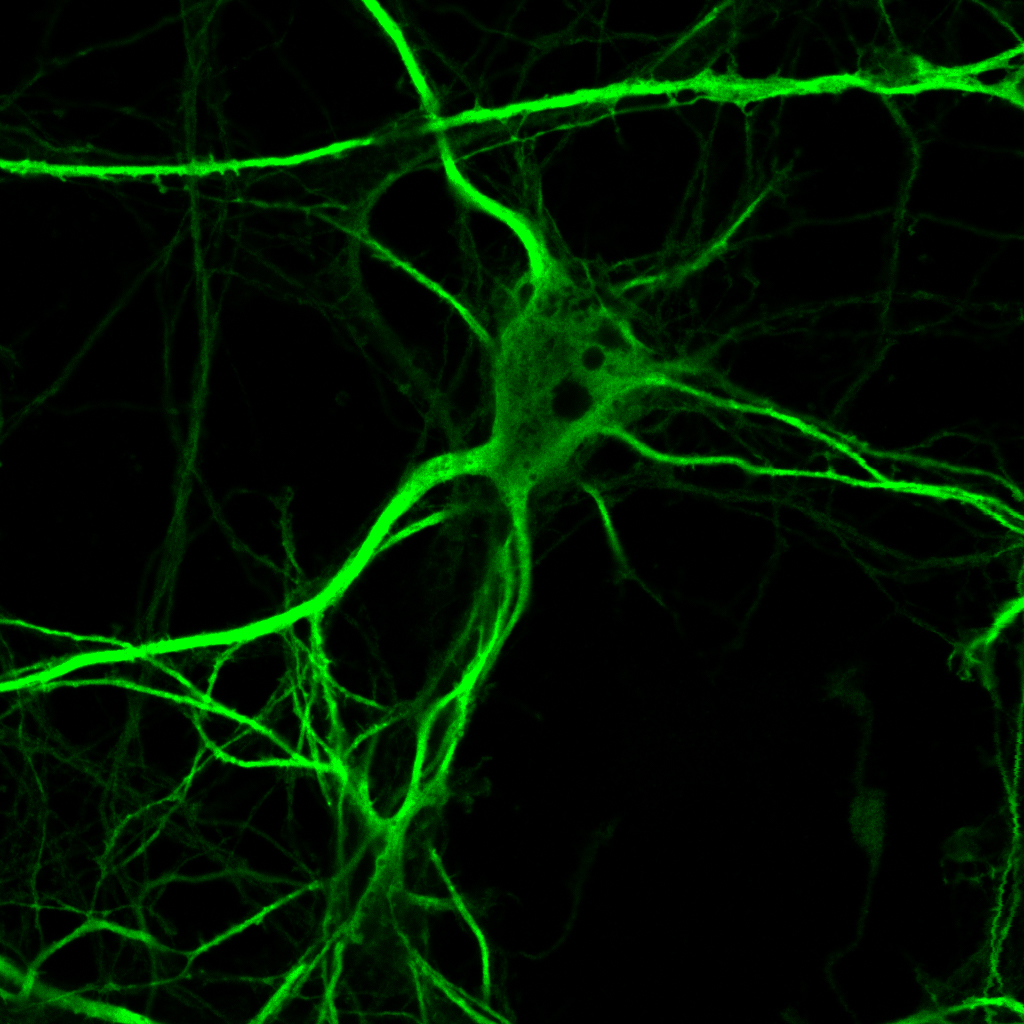

Supplement: Supplementary file 6 — Supporting File 6: advs74789‐sup‐0006‐Data.zip. [file ADVS-13-e22572-s004.zip › p_alk_map2-488.jpg]

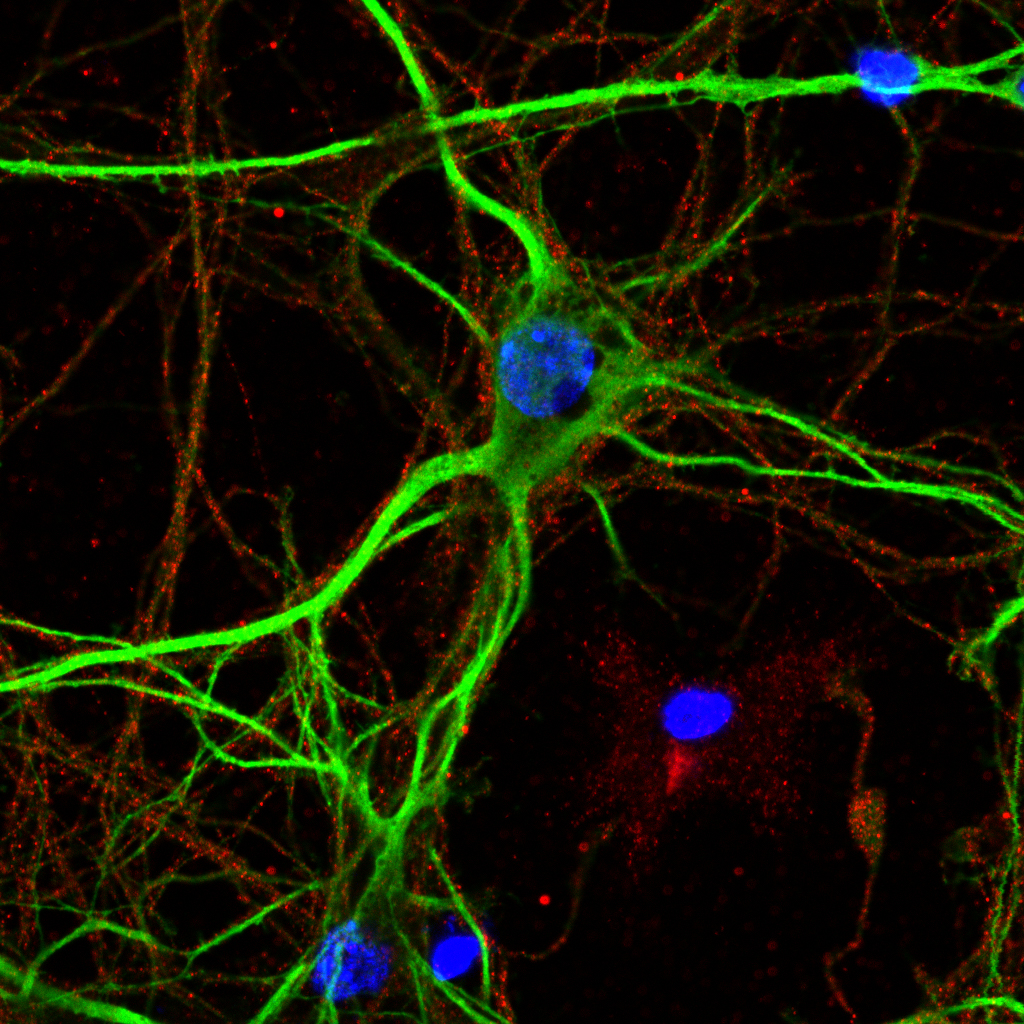

Supplement: Supplementary file 6 — Supporting File 6: advs74789‐sup‐0006‐Data.zip. [file ADVS-13-e22572-s004.zip › p_alk_merge.jpg]

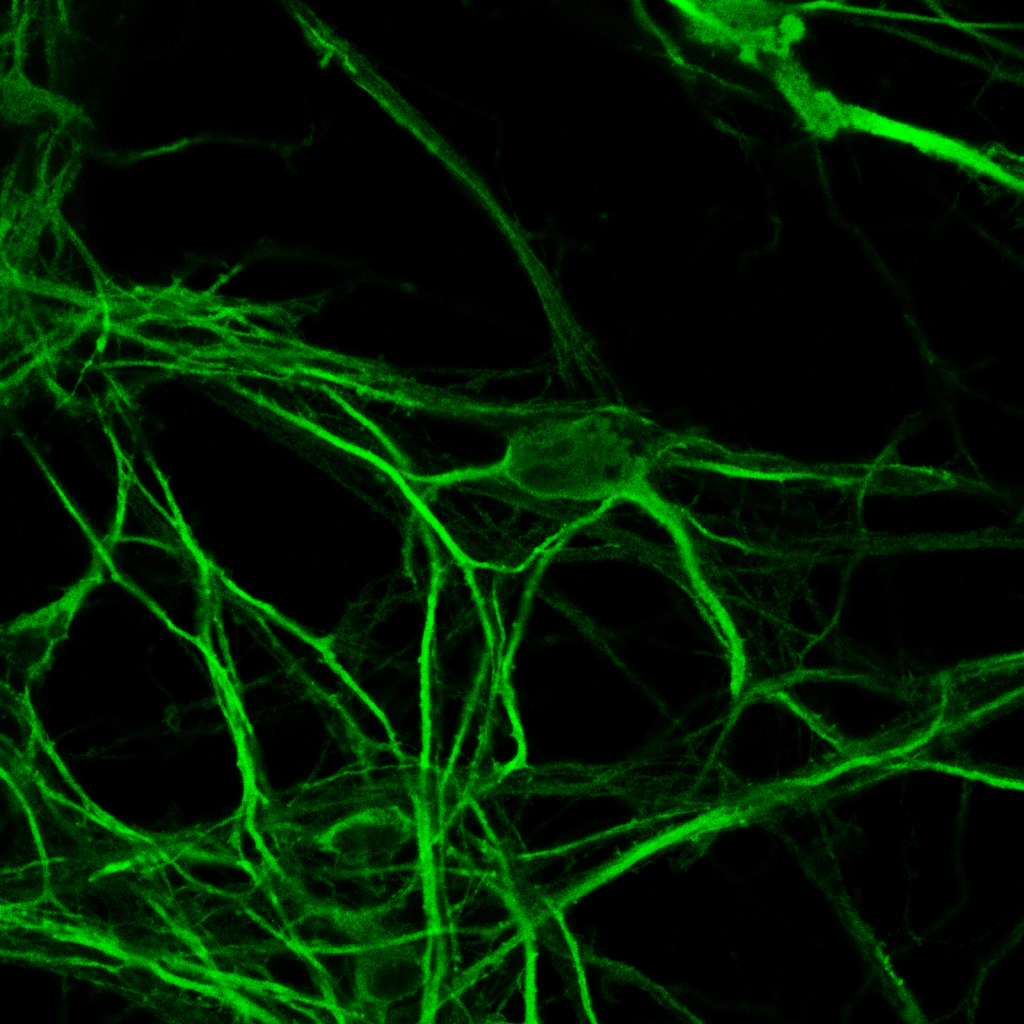

Supplement: Supplementary file 6 — Supporting File 6: advs74789‐sup‐0006‐Data.zip. [file ADVS-13-e22572-s004.zip › p_vec_map2-488.jpg]

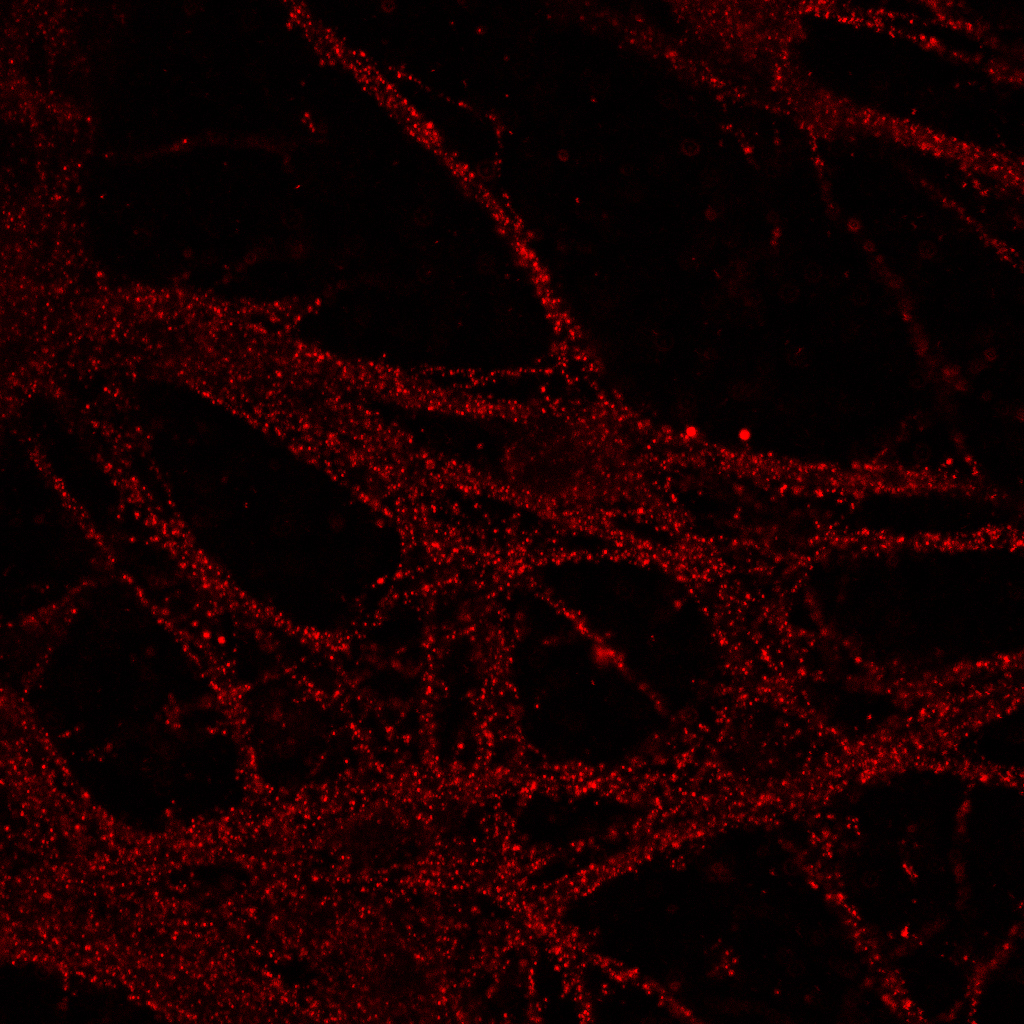

Supplement: Supplementary file 6 — Supporting File 6: advs74789‐sup‐0006‐Data.zip. [file ADVS-13-e22572-s004.zip › p_vec_tomm20-594.jpg]

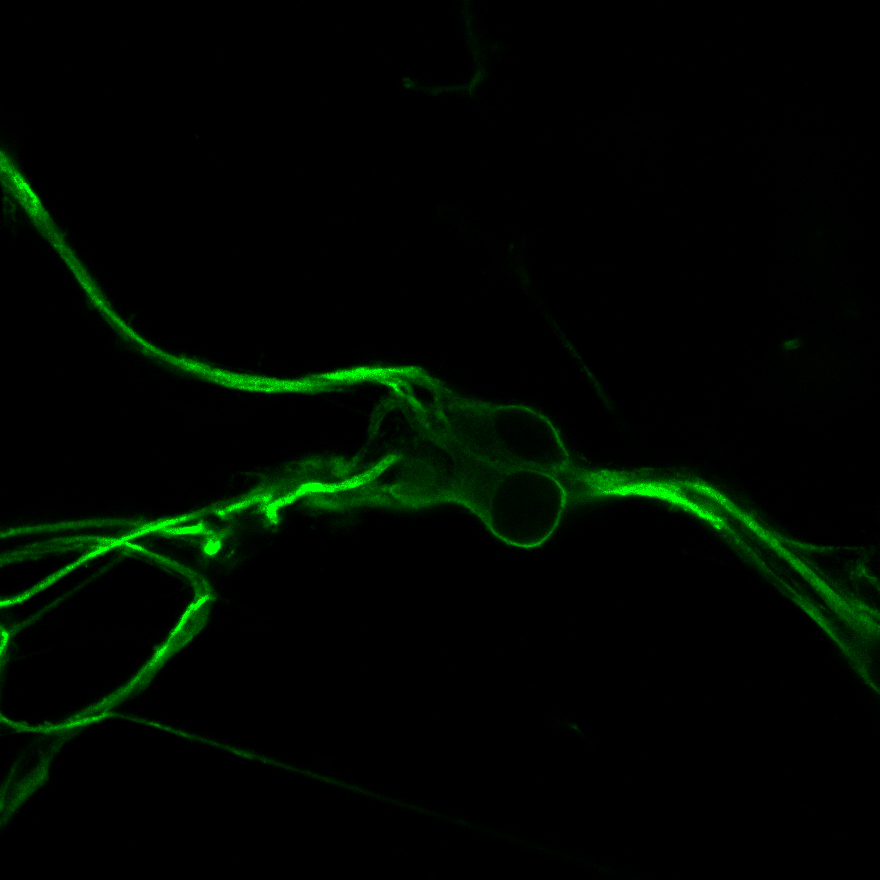

Supplement: Supplementary file 6 — Supporting File 6: advs74789‐sup‐0006‐Data.zip. [file ADVS-13-e22572-s004.zip › q_shAlk-map2.jpg]

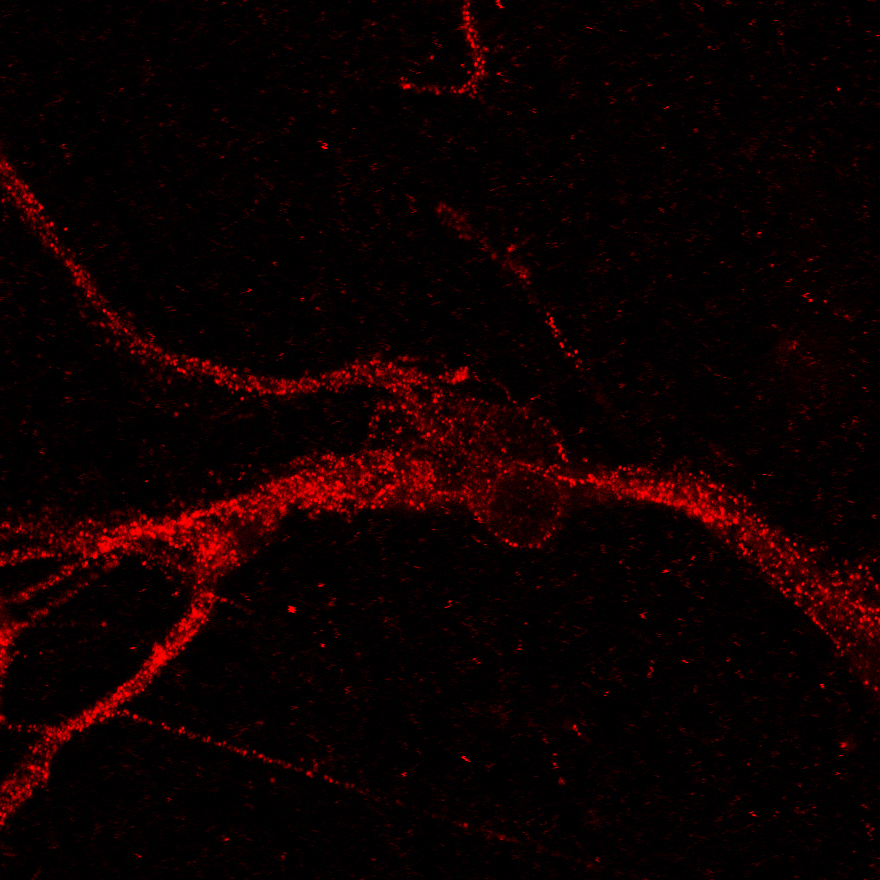

Supplement: Supplementary file 6 — Supporting File 6: advs74789‐sup‐0006‐Data.zip. [file ADVS-13-e22572-s004.zip › q_shAlk-tomm20.jpg]

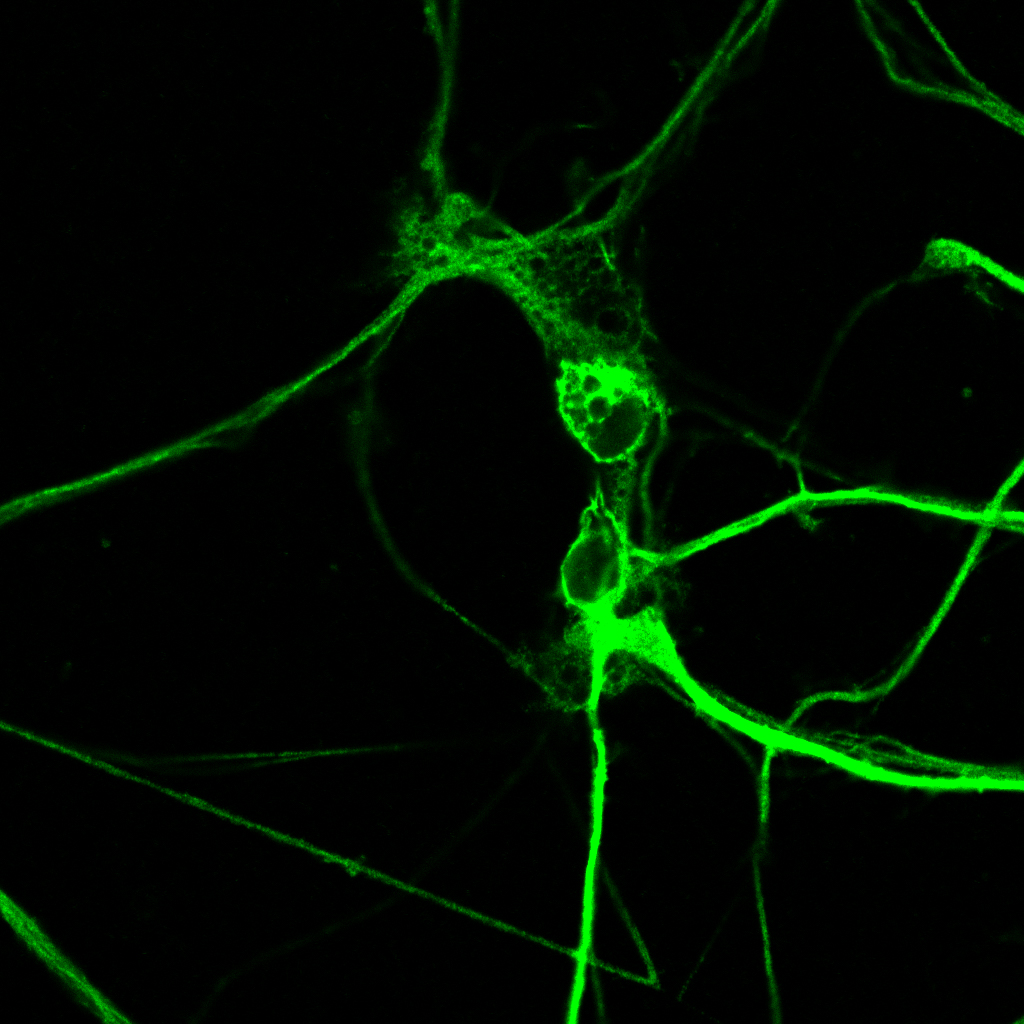

Supplement: Supplementary file 6 — Supporting File 6: advs74789‐sup‐0006‐Data.zip. [file ADVS-13-e22572-s004.zip › q_shcon-map2.jpg]

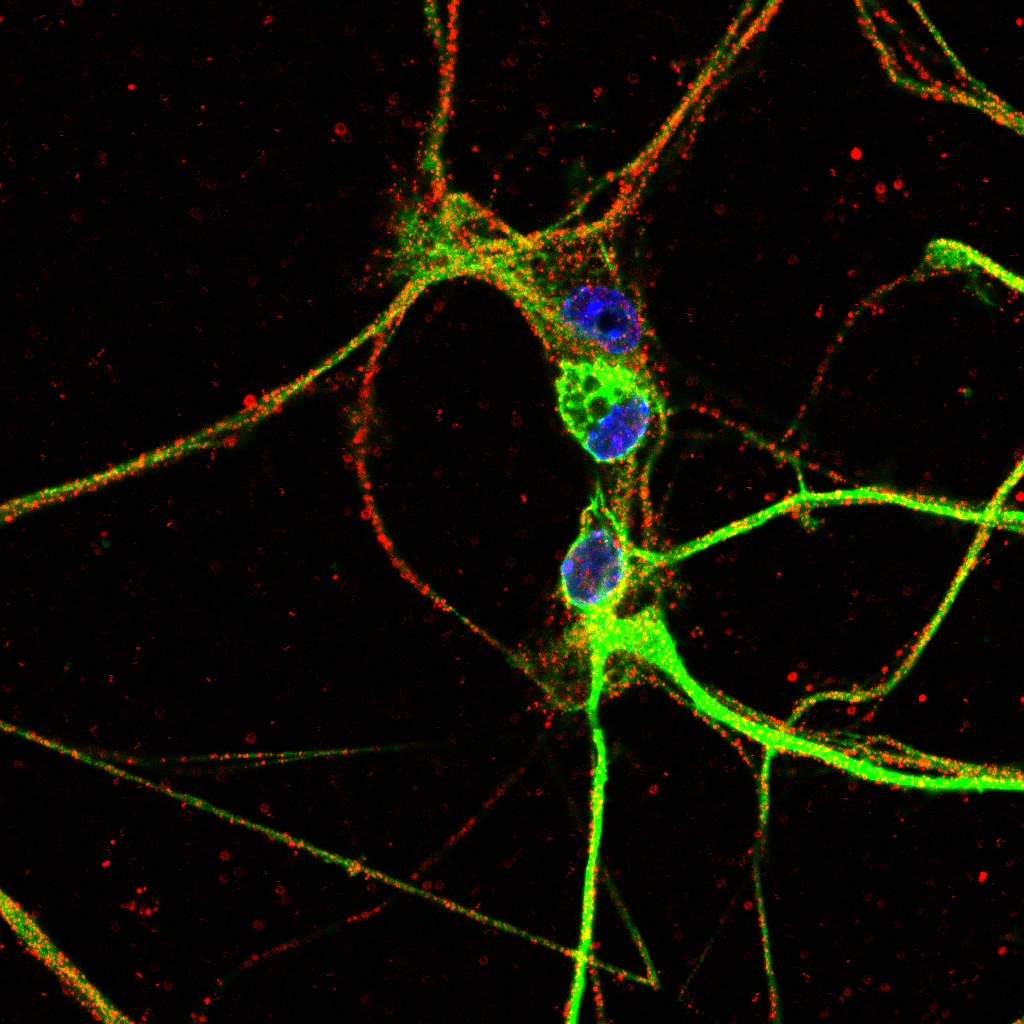

Supplement: Supplementary file 6 — Supporting File 6: advs74789‐sup‐0006‐Data.zip. [file ADVS-13-e22572-s004.zip › q_shcon-merge.jpg]

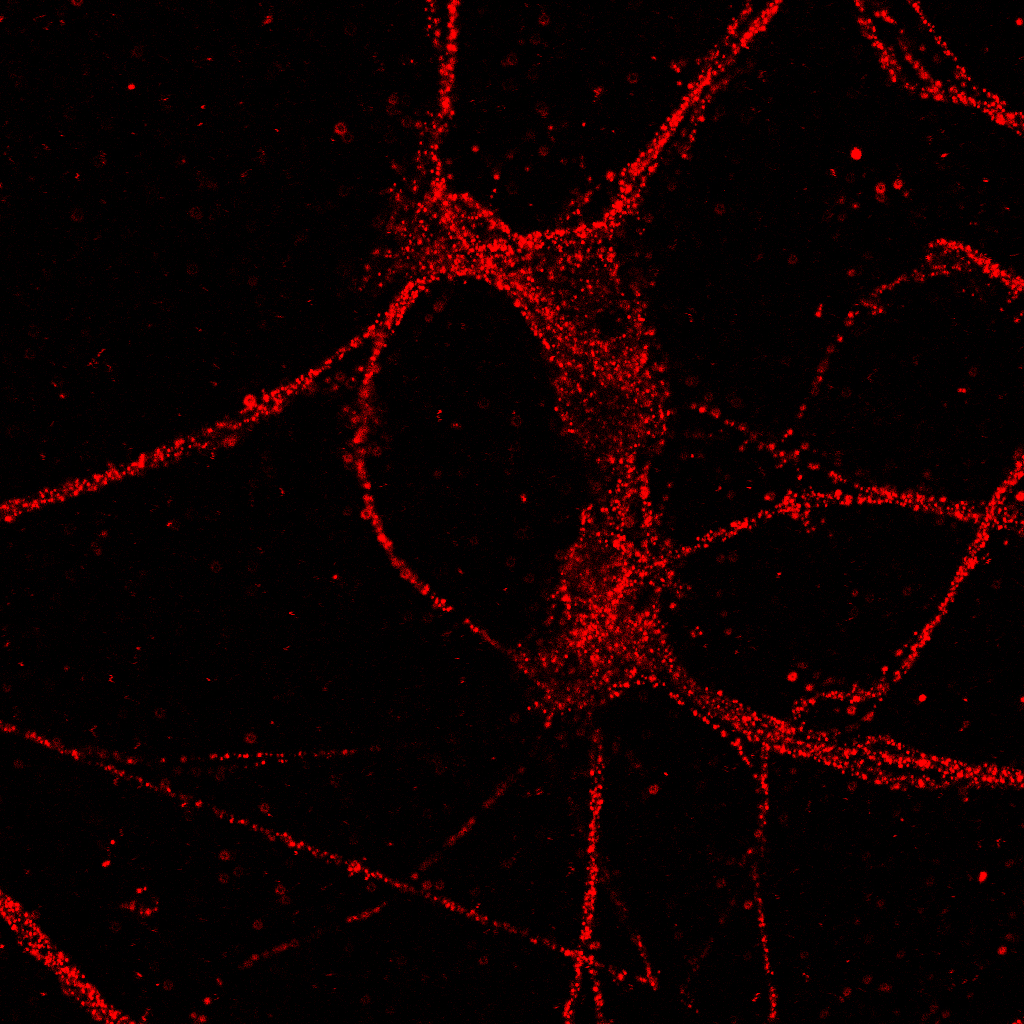

Supplement: Supplementary file 6 — Supporting File 6: advs74789‐sup‐0006‐Data.zip. [file ADVS-13-e22572-s004.zip › q_shcon-tomm20.jpg]

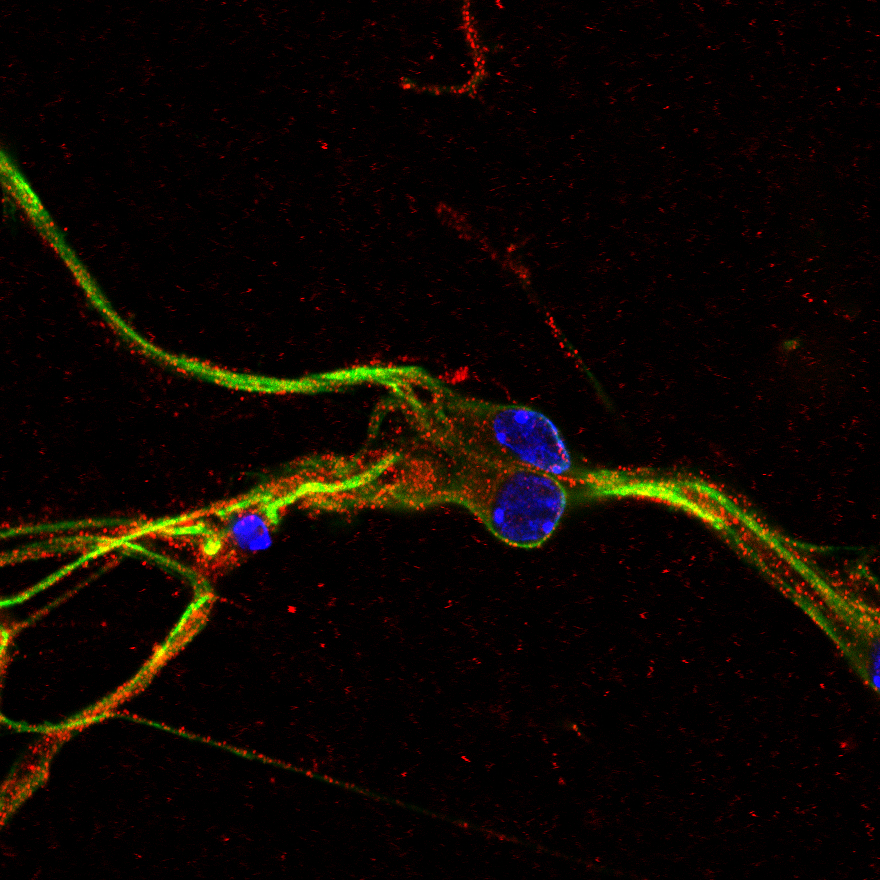

Supplement: Supplementary file 6 — Supporting File 6: advs74789‐sup‐0006‐Data.zip. [file ADVS-13-e22572-s004.zip › q_shAlk-merge.jpg]

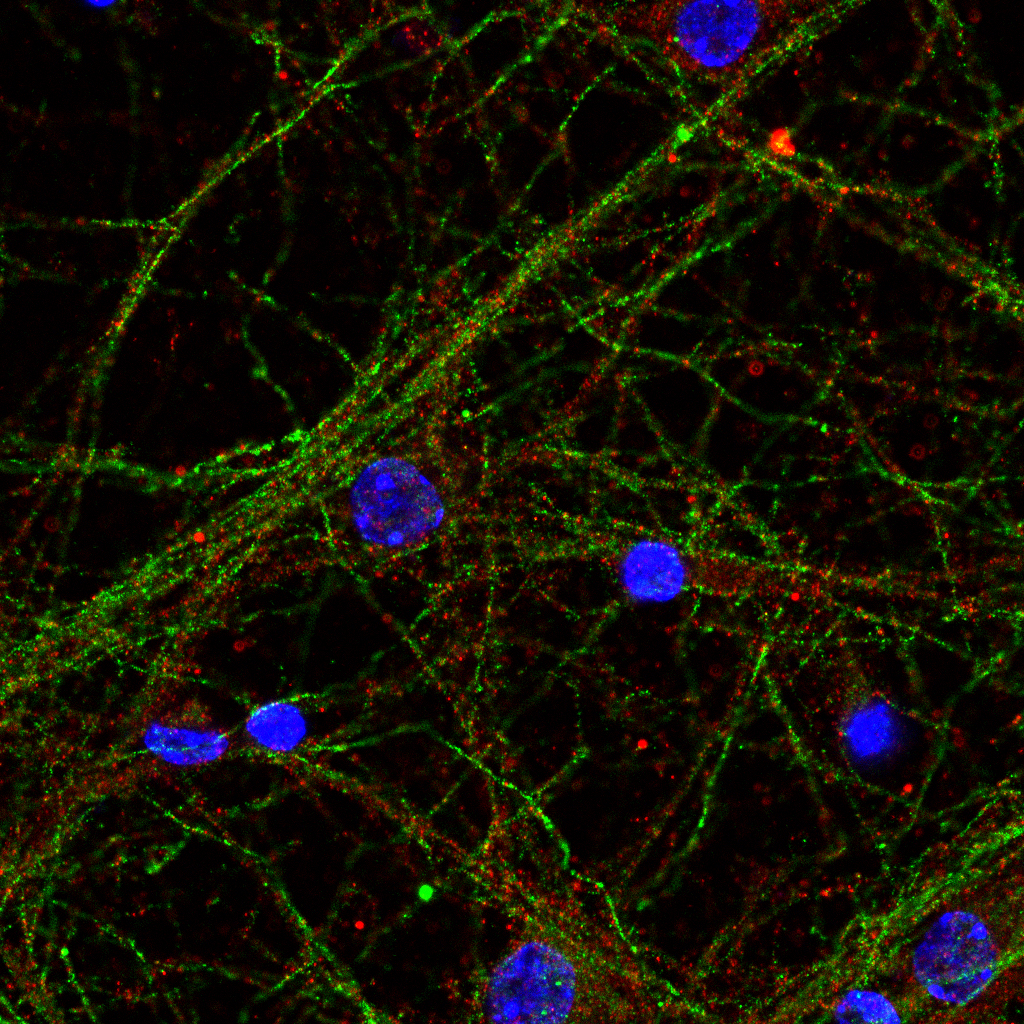

Supplement: Supplementary file 6 — Supporting File 6: advs74789‐sup‐0006‐Data.zip. [file ADVS-13-e22572-s004.zip › r_Alk_merge.jpg]

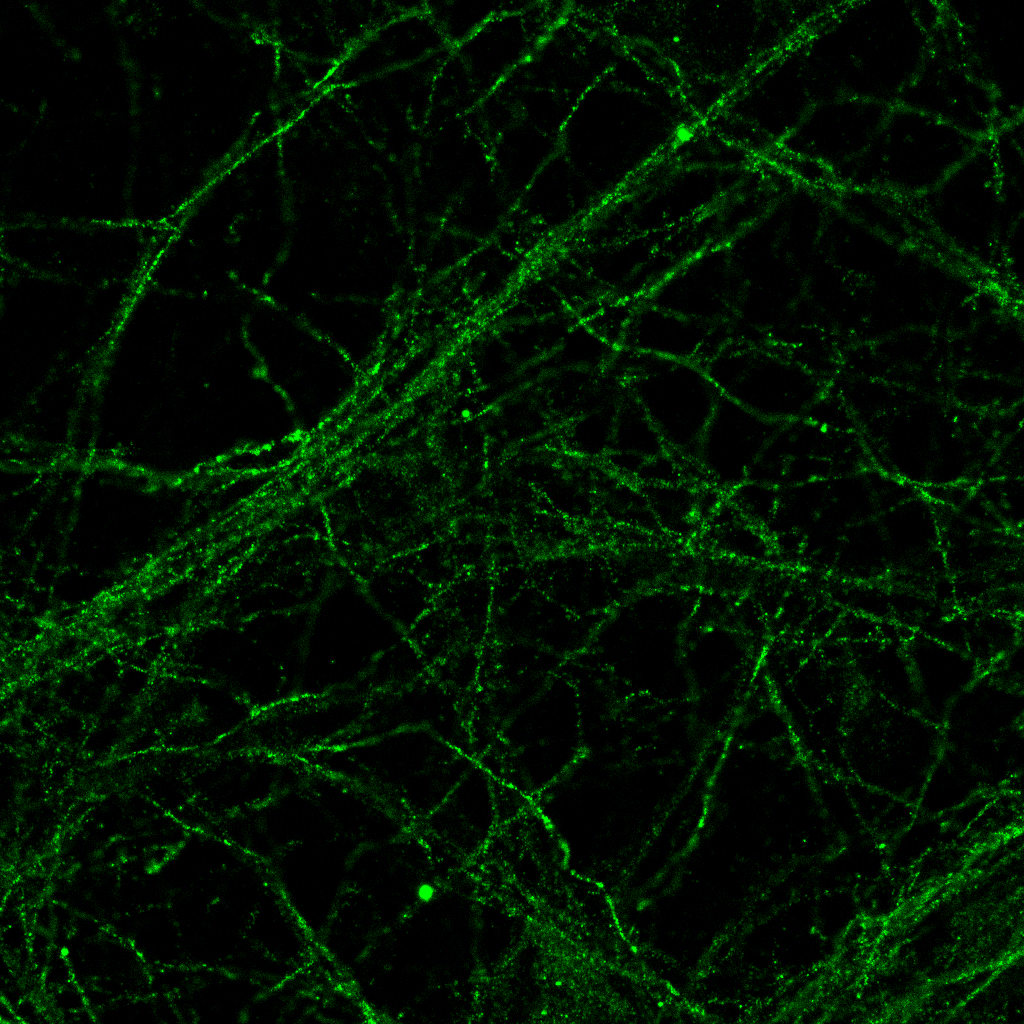

Supplement: Supplementary file 6 — Supporting File 6: advs74789‐sup‐0006‐Data.zip. [file ADVS-13-e22572-s004.zip › r_Alk_Tau_488.jpg]

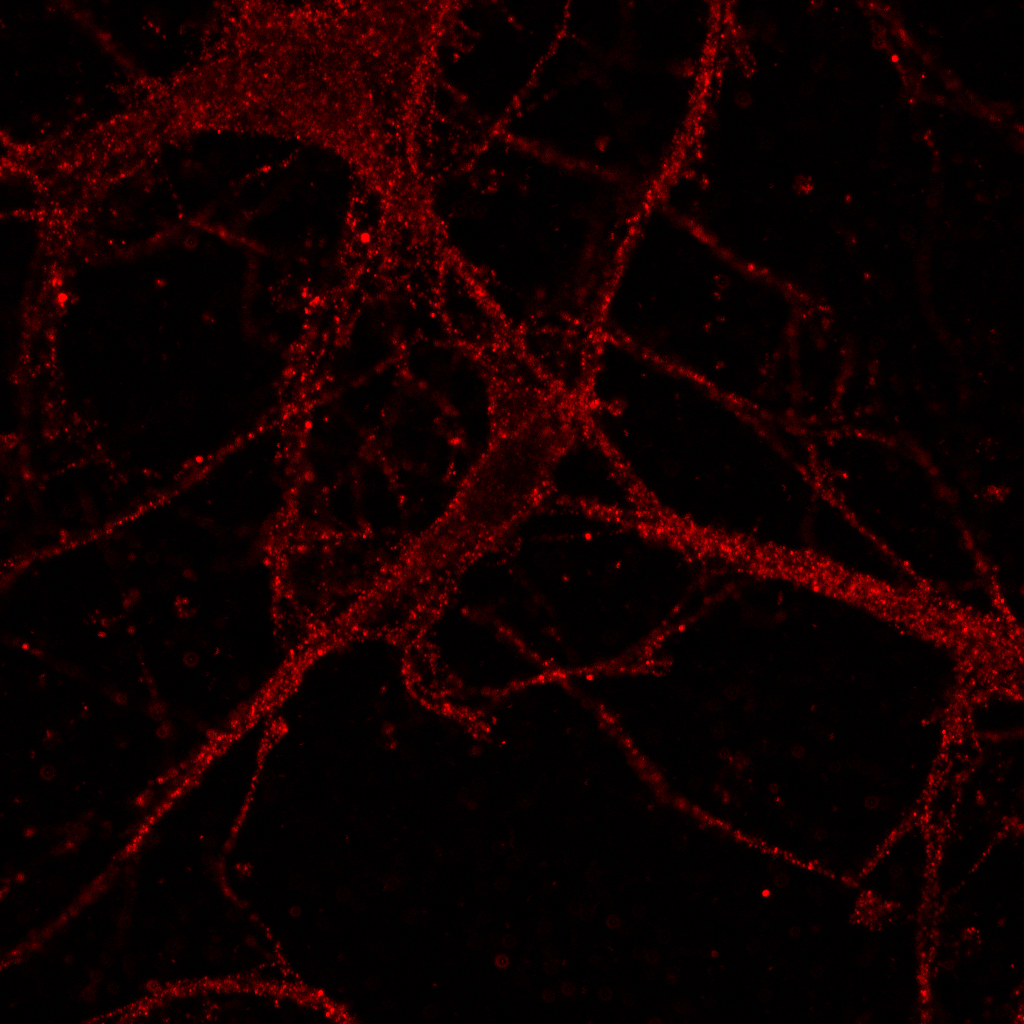

Supplement: Supplementary file 6 — Supporting File 6: advs74789‐sup‐0006‐Data.zip. [file ADVS-13-e22572-s004.zip › r_vec_Tomm20_594.jpg]

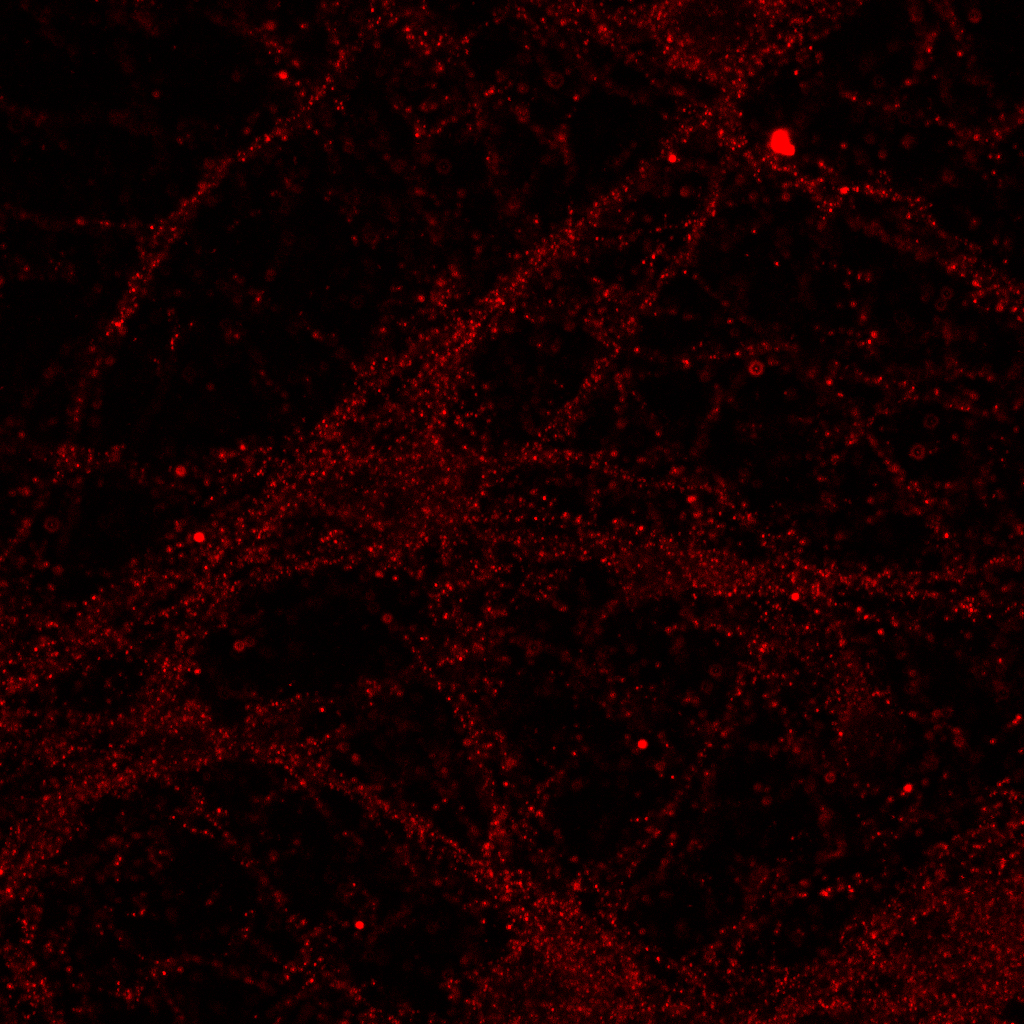

Supplement: Supplementary file 6 — Supporting File 6: advs74789‐sup‐0006‐Data.zip. [file ADVS-13-e22572-s004.zip › r_Alk_Tomm20.jpg]

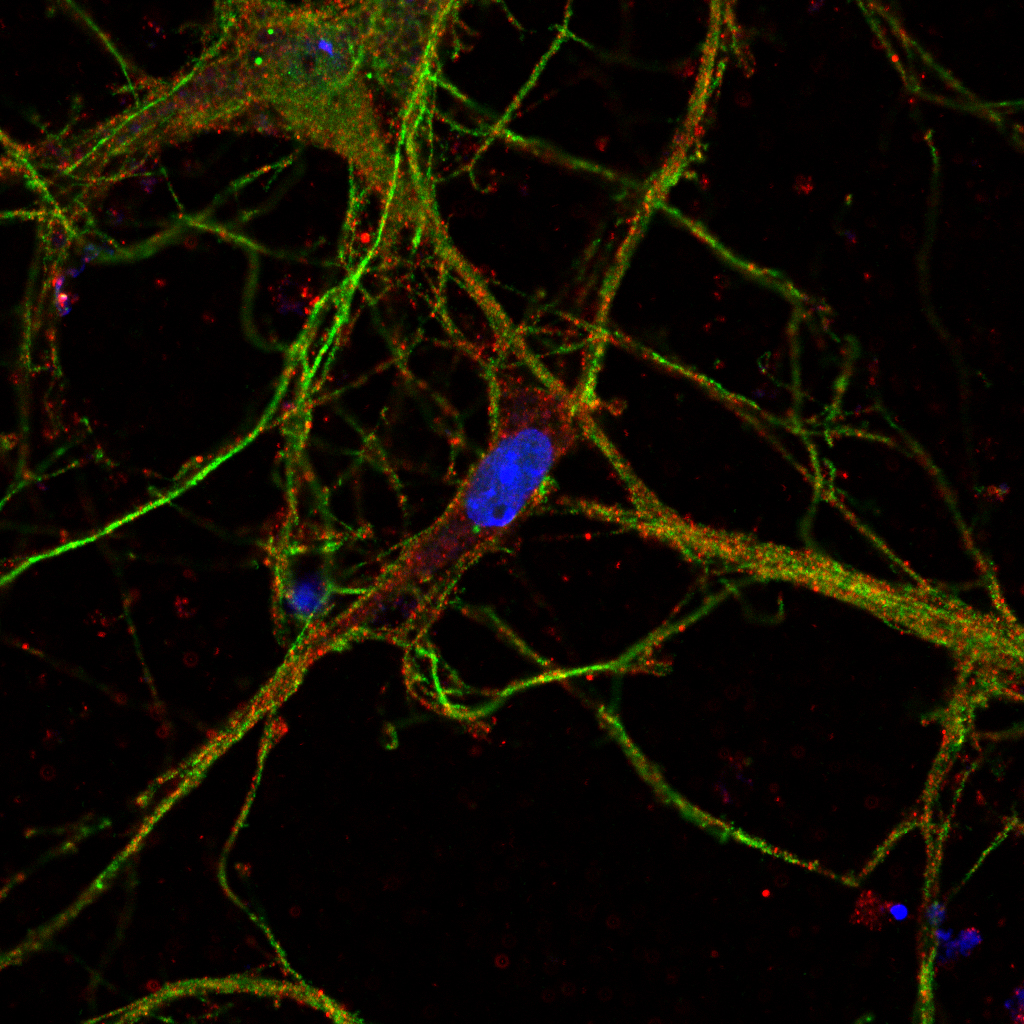

Supplement: Supplementary file 6 — Supporting File 6: advs74789‐sup‐0006‐Data.zip. [file ADVS-13-e22572-s004.zip › r_vec_merge.jpg]

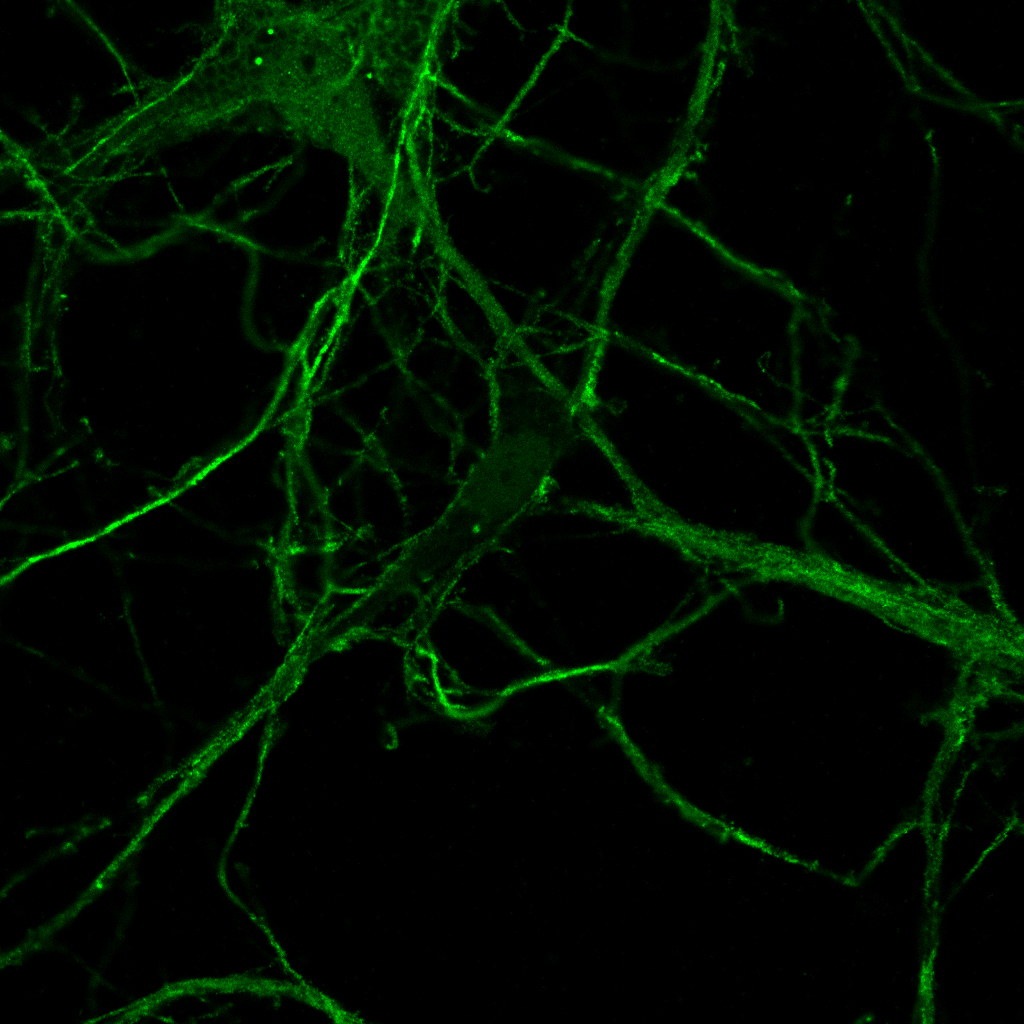

Supplement: Supplementary file 6 — Supporting File 6: advs74789‐sup‐0006‐Data.zip. [file ADVS-13-e22572-s004.zip › r_vec_Tau_488.jpg]

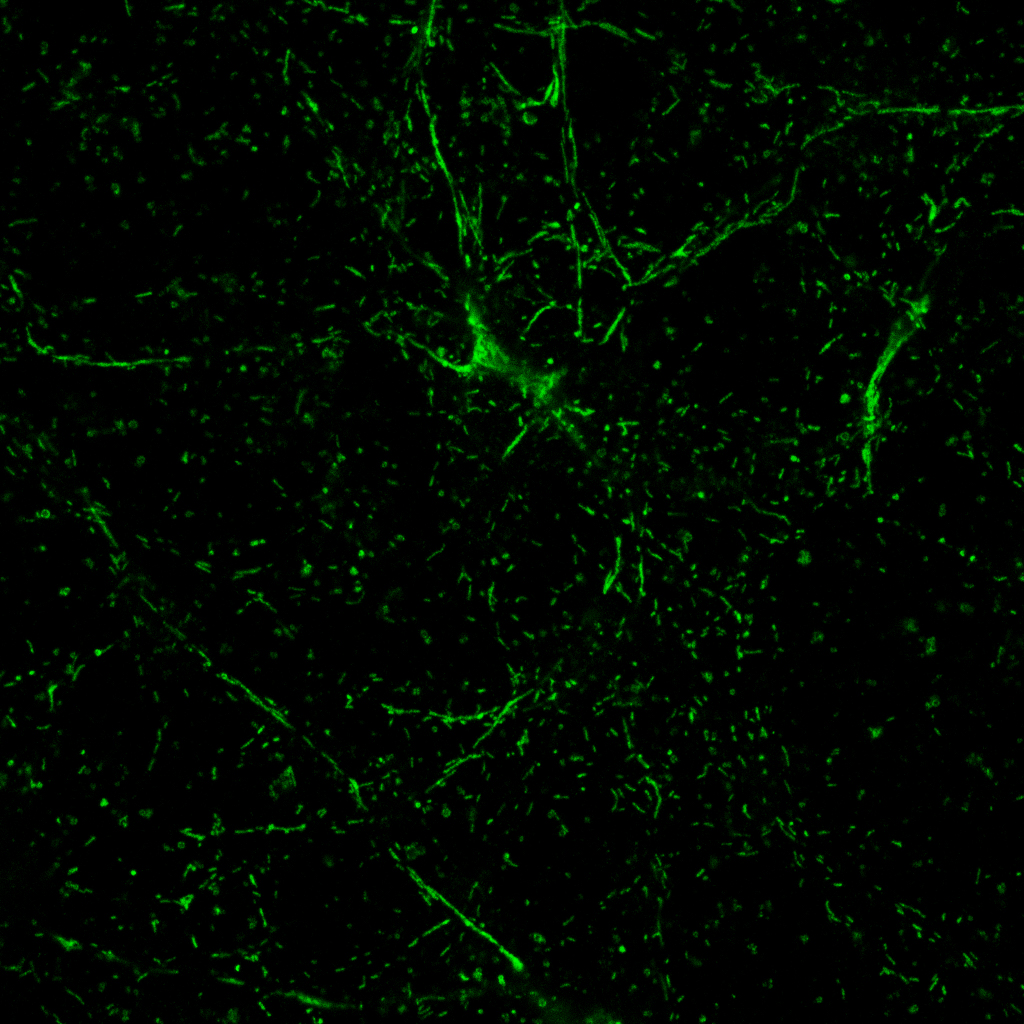

Supplement: Supplementary file 6 — Supporting File 6: advs74789‐sup‐0006‐Data.zip. [file ADVS-13-e22572-s004.zip › s_shcon-tomm20-5.jpg]

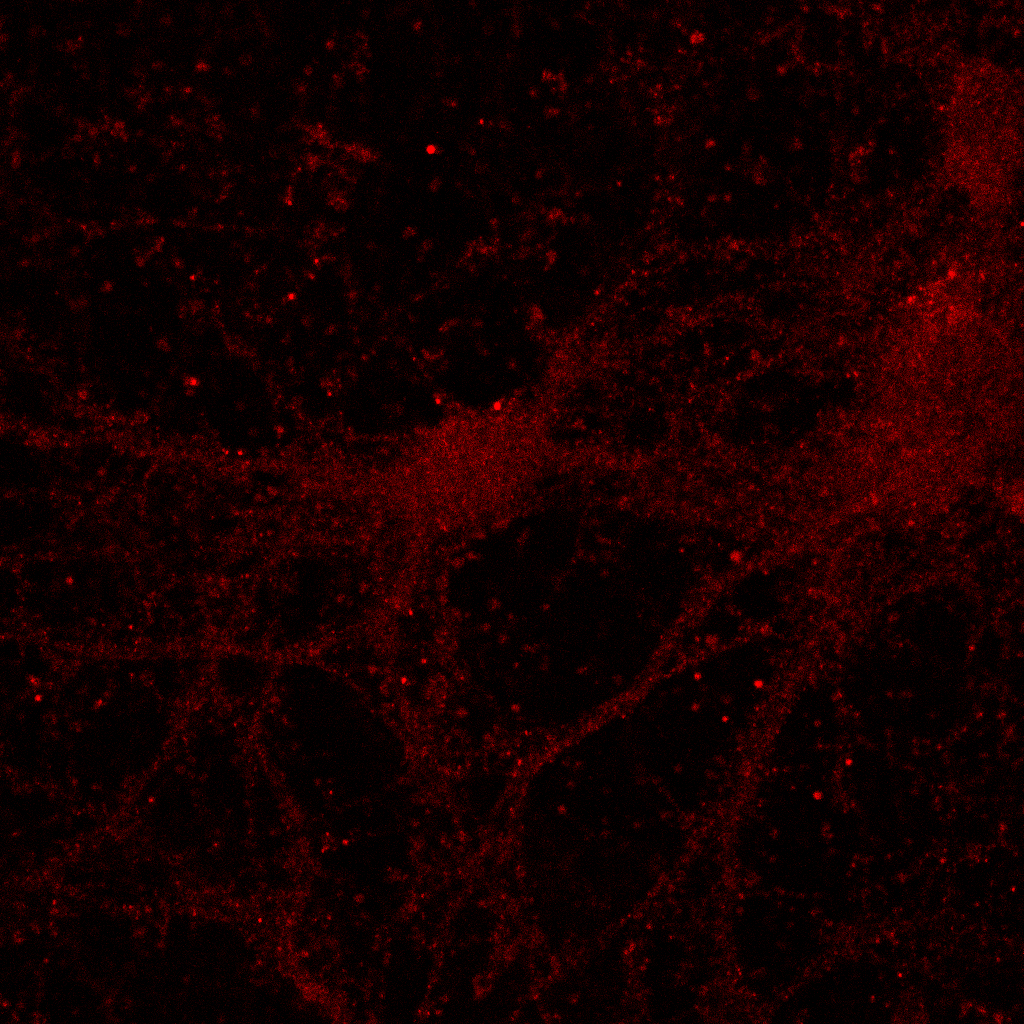

Supplement: Supplementary file 6 — Supporting File 6: advs74789‐sup‐0006‐Data.zip. [file ADVS-13-e22572-s004.zip › s_shAlk_Tau.jpg]

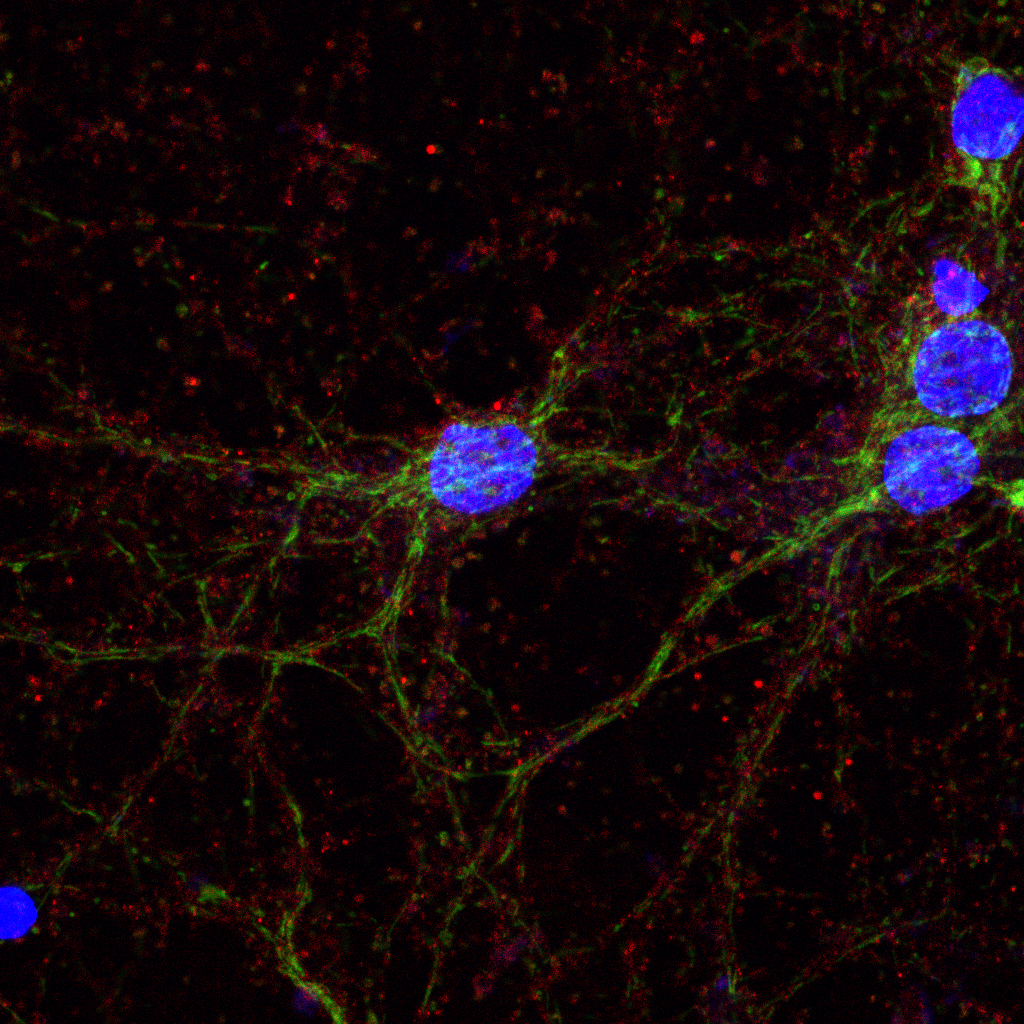

Supplement: Supplementary file 6 — Supporting File 6: advs74789‐sup‐0006‐Data.zip. [file ADVS-13-e22572-s004.zip › s_shAlk_Merge.jpg]

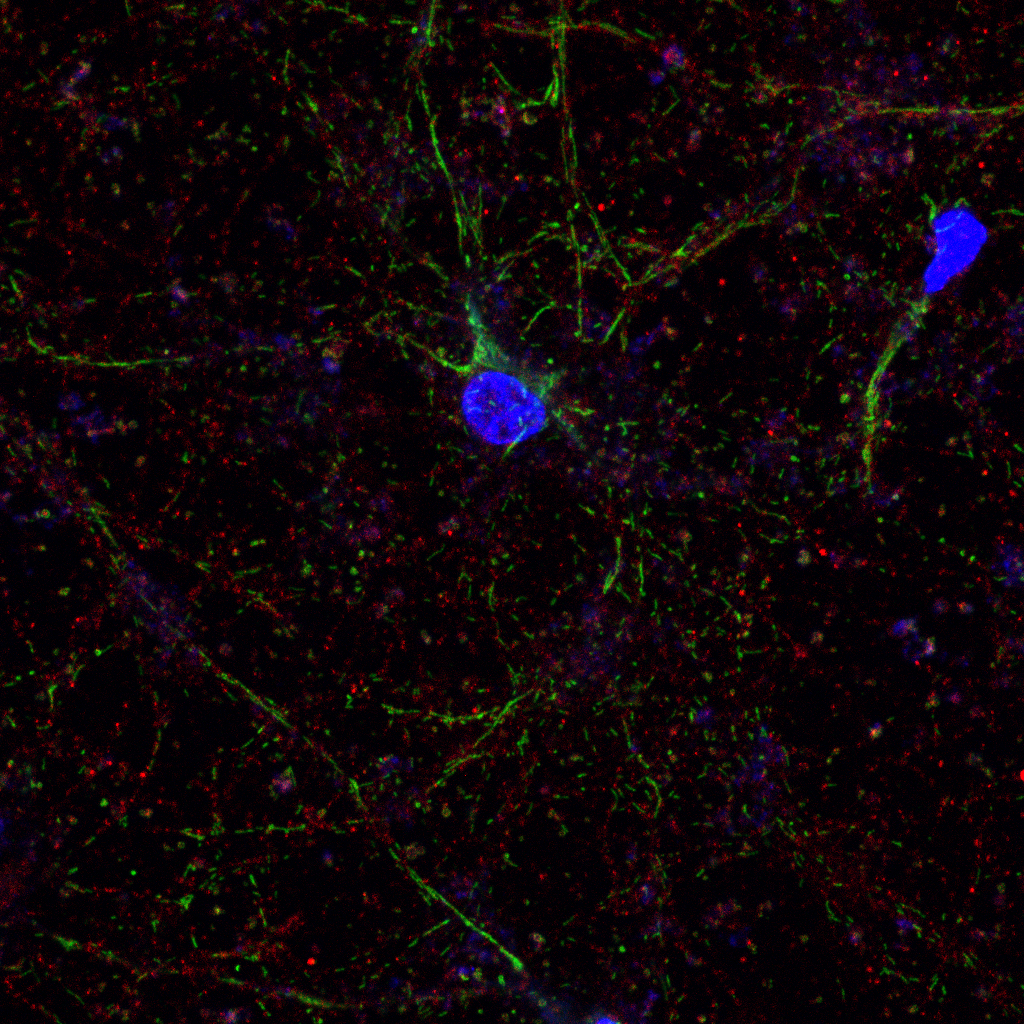

Supplement: Supplementary file 6 — Supporting File 6: advs74789‐sup‐0006‐Data.zip. [file ADVS-13-e22572-s004.zip › s_shcon_merge.jpg]

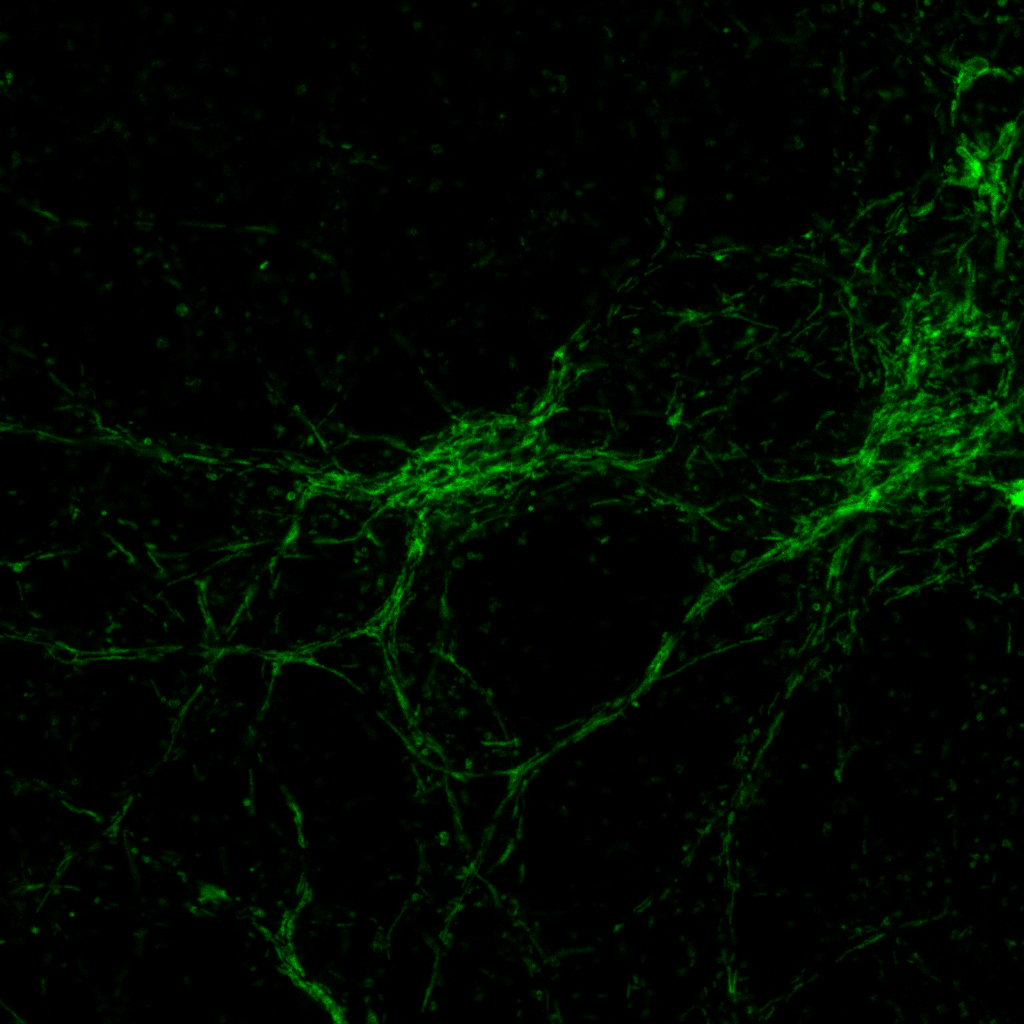

Supplement: Supplementary file 6 — Supporting File 6: advs74789‐sup‐0006‐Data.zip. [file ADVS-13-e22572-s004.zip › s_shAlk_Tomm20.jpg]

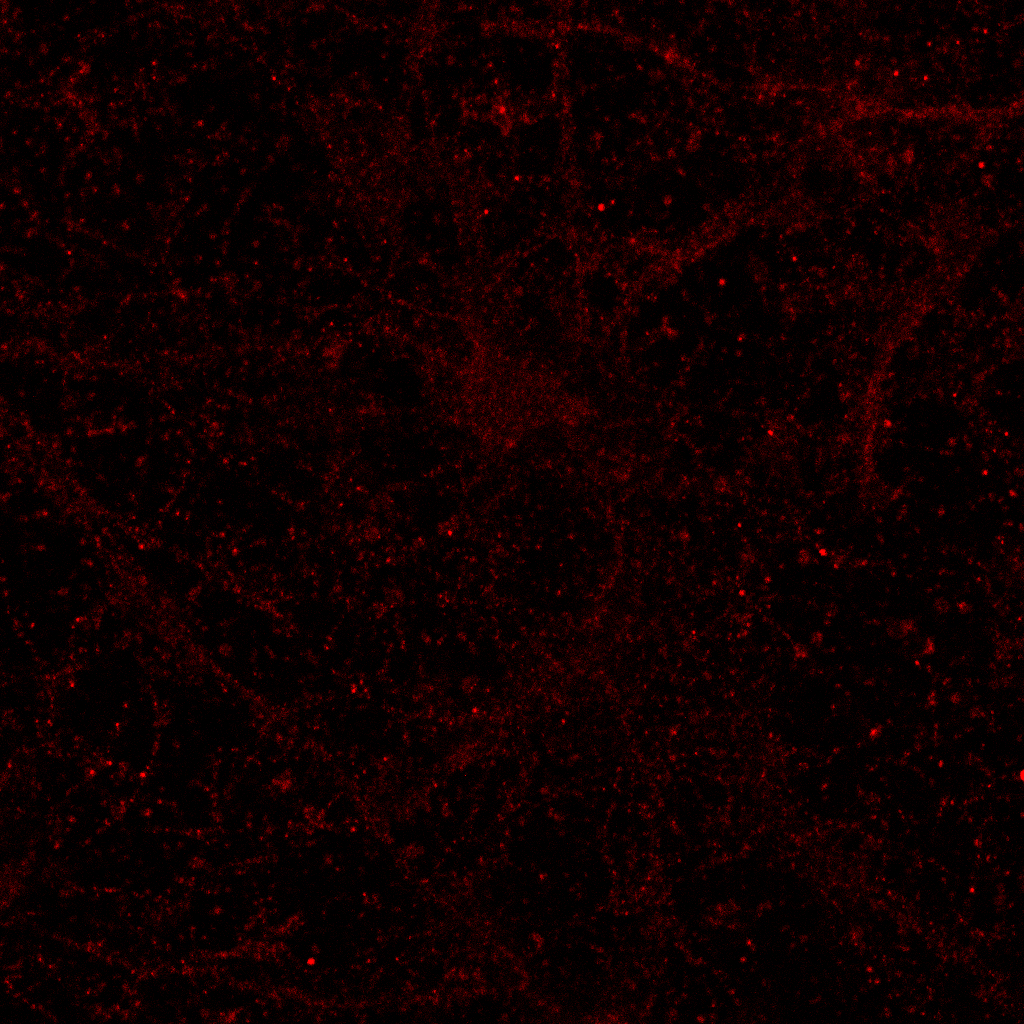

Supplement: Supplementary file 6 — Supporting File 6: advs74789‐sup‐0006‐Data.zip. [file ADVS-13-e22572-s004.zip › s_shcon_tau.jpg]
